# Effect of Antihypertensive Treatment on Hypotension, Mortality and Length of Stay in Orthopedic Trauma and First Detected High Blood Pressure Adults in a Large Urban Hospital: A Retrospective Cohort Study

**DOI:** 10.1111/jch.70210

**Published:** 2026-01-30

**Authors:** Carlos José Atencia, Fabian Jaimes

**Affiliations:** ^1^ Department of Internal Medicine School of Medicine Universidad De Antioquia Hospital San Vicente Fundacion Medellin Colombia

## Abstract

Trauma is a frequent cause of hospital admission in adults, and while arterial hypertension is common in the general population, reactive hypertension associated with trauma remains poorly studied from a therapeutic perspective. This retrospective cohort study aimed to estimate the impact of antihypertensive treatment on hypotension occurrence, in‐hospital mortality, and length of stay in patients with musculoskeletal trauma. We analyzed data from a high‐complexity center in Colombia between 2020 and 2024, including adults over 18 years with musculoskeletal trauma who had no previously known hypertension but presented two blood pressure readings >140/90 mm Hg during hospitalization. Patients were categorized into those receiving antihypertensive treatment versus no prescription. Primary outcomes were assessed using logistic, linear, and time‐to‐event regression models for hypotension requiring medical intervention, in‐hospital death, and length of stay. Among 712 patients analyzed, most were young men (77% male, mean age 35 years) with few comorbidities (obesity 6.2%, diabetes 4.6%). Sixty percent had open fractures requiring surgical management. Antihypertensive drug exposure was significantly associated with hypotension (OR 11.9, 95% CI 5.69–26.4) but showed no significant association with in‐hospital death (OR 5.18, 95% CI 0.79–39.6), length of stay (1.5 days, 95% CI −0.1 to 3.1), or time‐to‐discharge alive (HR 0.79, 95% CI 0.59–1.06). Our findings suggest that treating reactive hypertension in hospitalized musculoskeletal trauma patients may increase hypotension risk without improving mortality or length of stay outcomes.

## 1 Introduction

Young adults are especially prone to musculoskeletal trauma [1]. In 2019, the World Health Organization (WHO) reported that more than 90% of car accidents occurred in developing countries [1], and in Colombia, assaults and transport accidents caused 13 302 deaths in men aged 15 to 44 years, representing 54.7% of the causes of death in this age group [2]. Preventive strategies [2], Advanced Trauma Life Support (ATLS) [3], damage control and early fluid resuscitation [4] have improved initial survival in trauma [5, 6]. After their acute phase, these survivors face risks of death, complications such as sepsis [5, 7] and the need for various medical and surgical interventions [7, 8]. Also during hospitalization, some patients are detected with increasing in blood pressure, which could correspond to previously undiagnosed arterial hypertension or to a reactive phenomenon related to the stress of trauma, pain, anemia or anxiety [4, 8, 9].

On the other hand, hypertension is an everywhere public‐health problem. A meta‐analysis showed a prevalence of 24% of hypertension in adults in our country [10], and other reports indicate that—in the young population—undiagnosed and untreated hypertension is more frequent [11]. Consequently, hospital‐care physicians tend to overestimate the effect of blood pressure values ​​and, therefore, are prone to treat moderately high levels of hypertension in hospitalized patients, even without symptoms [12, 13]. Antihypertensive drug therapy in these acutely ill patients may be associated with episodes of hypotension and prolonged hospital stays [14, 15, 16], so its prescription and dosing must be balanced, as it may cause more harm than benefit when its pharmacological effect is combined with the effects of anesthesia, blood loss, and the use of other medications [17, 18].

The practice guidelines of the European Society of Cardiology, the American Heart Association (AHA), and the International Society of Hypertension do not include specific recommendations for the treatment of hypertension in trauma patients [19, 20, 21]. The ATLS guidelines also have no recommendations on the management of blood pressure in trauma patients [3]. A systematic search of some biomedical databases (Appendix 1) only found studies on the behavior and prognosis of blood pressure in cranioencephalic, spinal and closed abdominal trauma, and use of intravenous antihypertensive medications in elderly patients admitted for noncardiac medical causes [22, 23, 24, 25]. Given this lack of specific recommendations, the care of these patients is usually determined exclusively by the criteria of the attending physician, and there is no clear perspective of the balance between the risk and benefit of such decisions.

The objective of this study was to estimate the impact of antihypertensive treatment on the occurrence of hypotension, in‐hospital mortality, and length‐of‐stay in patients hospitalized for musculoskeletal trauma in a high‐complexity hospital in Medellín, Colombia.

## 2 Methods

### 2.1 Study Design

A retrospective cohort that includes adult patients with musculoskeletal trauma hospitalized at the San Vicente Fundación University Hospital (Medellín, Colombia) between January 2020 and April 2024, with International Classification of Diseases (ICD)‐10 diagnostic codes from Chapter XIX Trauma and some other consequences of external causes (S00–T98) (Appendix 2). The hospital is a high‐complexity center with 229 general‐hospital beds and 130 critical‐care beds for adults. Almost 20 000 surgeries are performed annually, as well as 55 000 triage events and more than 22 000 emergency‐care visits. The patients in the cohort were followed during their hospital stay until discharge or death.

### 2.2 Selection and Description of Participants

Patients aged 18 years or older with musculoskeletal trauma, who required at least 3 days of hospitalization and had systolic blood pressure (SBP) >140 mm Hg or diastolic blood pressure (DBP) >90 mm Hg on at least two independent measurements after the first 48 h of hospitalization were included. SBP and DBP values ​​were chosen according to standard definitions of hypertension from current clinical practice guidelines, and the first 48 h of care is the estimated time, according to ATLS guidelines, for the patient to be stabilized, resuscitated, and primary and secondary trauma injuries to be ruled out. Patients were excluded if they had a known prior diagnosis of hypertension, hospitalization for head trauma or abdominal trauma with renal involvement, hypertensive emergencies including stimulant intoxication, or patients admitted to the Intensive Care Unit (ICU) due to neurological, ventilatory, or hemodynamic impairment secondary to trauma. For these excluded conditions, there is scientific literature and various recommendations on the management of high blood pressure.

### 2.3 Variables (Appendix 3)

The exposure variable was defined as the prescription and administration of an antihypertensive medication for more than 48 h. The prescription frequency, duration, and type of medication were recorded. The outcomes of interest were the presence of an episode of hypotension, defined as mean arterial pressure (MAP) < 65 mm Hg, requiring some type of medical intervention (any of: requirement for intravenous fluids, elevation of extremities, suspension of medications, need for vasopressor, or transfer to the ICU), in‐hospital mortality, and length of hospital stay. Potential confounding variables collected at admission, selected based on a review of the literature and the opinion of two expert clinicians, were: age, sex, race, fractured bone, fracture type according to the Gustillo–Anderson (GA) classification [26], Revised Trauma Score (RTS, which measures trauma severity) [27] and history of smoking, obesity, alcoholism, diabetes, chronic kidney disease, myocardial infarct or stroke. During hospitalization, potential confounders were collected if they occurred before the hypertensive episode: amount of intravenous fluids administered, the need for a surgical procedure, prescription of nonsteroidal anti‐inflammatory analgesics and opioids, infection or sepsis, pain measured by visual analog scale, insomnia reported by the patient, constipation or prescription of laxatives, anxiety or stress, hemoglobin and creatinine values.

### 2.4 Data Collection and Measurements

The drug prescription variables are recorded in the electronic medical record in the Systems, Applications, Products in Data Processing (SAP) prescription module (Version 7.0 [28]). The outcome variables were obtained from the same electronic records where vital signs, the days of stay and the patient's vital state at discharge are recorded by the trained nursing staff. A database was designed using Access (Windows 11), standardized and validated according to the previous variables. Research assistants in charge of searching and collecting the information received specific training before the start of the project and two retraining sessions during the research. The database was audited every 3 months by the principal investigator, and errors were reported to the collection personnel for verification and correction.

### 2.5 Bias Control

Selection and measurement biases were controlled by strictly defined inclusion and exclusion criteria and standardized data collection, respectively. Additionally, the procedures mentioned above were implemented to minimize errors and ensure the accuracy of the information. The potential confounding variables for each outcome of interest, identified and selected as previously described, were corroborated and verified by means of causal diagrams (directed acyclic graph, DAG) [29] (Appendix 5, 6 and 7).

### 2.6 Sample Size

The sample size calculation was estimated using the percentages of hypotension, death, and length of stay from the studies available in the literature [22, 23, 24, 30]. A 1:3 ratio between exposed:unexposed groups was expected, with a Type 1 error of 5% and a Type 2 error of 20% for all outcomes [31]. For the mortality outcome, one study [22] reported an absolute difference of 5% (from 2% to 7%) with the use of antihypertensive drugs. For this difference in proportions, the necessary sample size would be 682 subjects. For the hypotension outcome, one study [23] reported a 2.5 mm Hg reduction in blood pressure values, but did not report results in terms of proportions of hypotension in the groups. For the outcome of length‐of‐hospital stay, the studies reviewed [22, 23] presented a difference of 3 days with a standard deviation of 1 day with the use of antihypertensive therapy, for which a sample size of 340 subjects in total would be needed. Accordingly, considering the potential confounding variables, the estimated sample size for the current research was 700 participants (Appendix 4).

## 3 Statistical Methods

### 3.1 Descriptive Analysis

Qualitative variables are presented as absolute and relative frequencies, and quantitative variables as medians and interquartile ranges or means and standard deviation, depending on their distribution.

### 3.2 Multivariate Association Analysis and Confounding Control

To evaluate the association between the prescription of antihypertensive drugs and the outcomes, adjustments were made with the specific set of previously defined confounding variables [22, 23, 24, 25] and presented in the causal structure of a DAG (Appendices 5, 6 and 7).

For the outcomes of hypotension requiring medical intervention and in‐hospital mortality, logistic regression models adjusted for the confounding variables were used. Odds ratios (OR) were calculated with their respective 95% confidence intervals (CI). Extreme data or leverage points in the models were evaluated, as well as multicollinearity by means of correlation coefficients and the variance inflation factor (VIF). Multivariate logistic models were performed for each of the hypotension outcomes requiring different types of medical interventions (Appendices 5 and 6).

For length‐of‐stay, two models were used: 1. Multiple linear regression for the continuous outcome of length‐of‐stay in days, and 2. Cox regression for the hazard of being discharged alive from the hospital, with in‐hospital death as a competing risk. There were 16 hospital stays longer than 60 days, so the length of stay (linear) and time‐to‐discharge alive models (competing risks) were also analyzed, excluding these extreme values (Appendix 7). In the linear model and in the Cox model, all the assumptions were checked, and the proportionality of the risks for the Cox model was evaluated by means of Schoenfeld residuals and Log‐Log curves.

We had missing data only on the variable hemoglobin and creatinine, with 9.9% and 19%, respectively. Considering the distribution of these variables and the possible mechanism of missing at random, simple imputation techniques were used based on the median of the available data. Logistic models were adjusted with the imputed variables and with the complete cases. A sensitivity analysis was performed for the loss of 13 eligible patients, assuming that all or none of them had episodes of hypotension and death (Appendix 8).

All analyses were performed using software R version 4.2.2 and the integrated development environment RStudio [32]. The final report was developed according to the recommendations of STROBE [33].

### 3.3 Ethical Aspects

The protocol was approved by the Bioethics Committee of the University of Antioquia Faculty of Medicine and the San Vicente Fundación Hospital. The research was conducted in accordance with the Declaration of Helsinki 2008 and Colombian regulations for research [34].

## 4 Results

**FIGURE 1:**
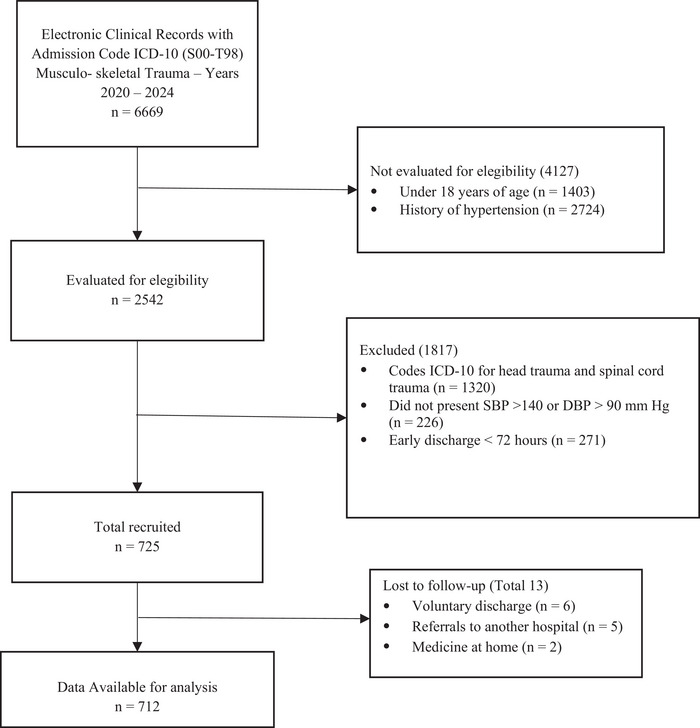
Patient collection flowchart.

Of 6669 clinical records of trauma admissions requiring hospitalization from 2020 to 2024, 2542 (38.1 %) were patients over 18 years of age and without a history of high blood pressure, of which 725 (28.5%) were eligible and 712 (28%) participants were analyzed (Figure 1).

The study population consisted primarily of men (77%, *n* = 547) with a mean age of 35 years and with few comorbidities (obesity: 6.2%, *n* = 44; diabetes mellitus: 4.6%, *n* = 33; consumption of alcohol: 57%, *n* = 408; and smoking: 46%, *n* = 330). Most patients (59%, *n* = 427) had open fractures that required surgical management and corresponded to Group 3 (21%, *n* = 153) of the Gustillo–Anderson classification [26] (Table 1). The antihypertensive medications most frequently prescribed de novo during hospitalization were Hydrochlorothiazide (33%) and Clonidine (34%). Infections (23%, *n* = 160) and the presence of insomnia (37%, *n* = 264), constipation (25%, *n* = 179) and anxiety (16%, *n* = 117) were most common in patients medicated with antihypertensives (Table 2).

**TABLE 1:** Baseline demographic and clinical characteristics of the study patients.

Characteristics	Total, *n* = 712^a^	Not prescribed, *n* = 63^a^	Prescribed, *n* = 80^a^
Male sex	547 (77%)	488 (77%)	59 (74%)
Latino race	704 (99%)	625 (99%)	79 (99%)
Age in years	35 (26, 52)	36 (26, 52)	35 (28, 51)
Heart rate in beats per minute (bpm)	89 (81, 98)	89 (81, 87)	100 (86, 102)
Respiratory rate in respirations per minute (rpm)	22 (20, 24)	22.0 (20, 24)	25.0 (21, 28)
Glasgow Coma Scale	15 (15, 15)	15 (14, 15)	15 (12, 15)
SBP on admission in mm Hg	128 (120, 136)	128 (120, 135)	130 (118, 140)
Revised Trauma Score (RTS)	3.69 (3.69, 3.69)	3.69 (3.69, 3.69)	3.69 (3.20, 3.69)
Fractured bone			
Clavicle	13 (1.8%)	11 (1.7%)	2 (2.5%)
Shoulder	4 (0.6%)	4 (0.6%)	0 (0%)
Humerus	44 (6.2%)	40 (6.3%)	4 (5.0%)
Elbow	7 (1.0%)	6 (1.0%)	1 (1.3%)
Radium or ulna	50 (7.0%)	45 (7.1%)	5 (6.3%)
Wrist	5 (0.7%)	5 (0.8%)	0 (0%)
Hane	9 (1.3%)	9 (1.4%)	0 (0%)
Pelvis	38 (5.3%)	31 (4.9%)	7 (8.8%)
Hip	40 (5.6%)	36 (5.7%)	4 (5.0%)
Femur	181 (25%)	151 (24%)	30 (38%)
Knee	21 (3.0%)	18 (2.9%)	3 (3.8%)
Tibia–Fibula	243 (34%)	220 (35%)	23 (29%)
Ankle	23 (3.2%)	22 (3.5%)	1 (1.3%)
Foot	33 (4.6%)	33 (5.2%)	0 (0%)
GA classification			
Closed	285 (40%)	259 (41%)	26 (33%)
Grade 1	129 (18%)	117 (19%)	12 (15%)
Grade 2	145 (20%)	130 (21%)	15 (19%)
Grade 3	153 (21%)	126 (20%)	27 (34%)
Weight in kg	69 (62, 72)	68 (62, 72)	70 (66, 80)
Cigarette, tobacco, or vaper smoker	330 (46%)	296 (47%)	34 (43%)
Alcohol consumption	408 (57%)	361 (57%)	47 (59%)
Obesity	44 (6.2%)	33 (5.2%)	11 (14%)
Diabetes mellitus Type 1 or 2	33 (4.6%)	27 (4.3%)	6 (7.5%)
Myocardial infarction	3 (0.4%)	2 (0.3%)	1 (1.3%)
Stroke	2 (0.3%)	2 (0.3%)	0 (0%)

Abbreviation: RTS, revised trauma score.

^a^ Dichotomous and categorical variables are presented in absolute numbers and percentages (%); continuous quantitative variables are presented as median with interquartile range (IQR). GA, Gustillo–Anderson. Grade 1, Dermis opening <1 cm. Grade 2, Dermis opening of 1–10 cm. Grade 3, Opening > 10 cm with tissue damage.

**TABLE 2:** Characteristics of interventions, drug prescriptions and complications during hospitalization of the study patients.

Characteristics	Total, *n* = 712^a^	Not prescribed, *n* = 632^a^	Prescribed, *n* = 80^a^
Systolic blood pressure in mm Hg^b^	153 (144, 162)	151 (142, 159)	168 (161, 176)
Diastolic blood pressure in mm Hg^b^	92 (90, 97)	91 (90, 96)	98 (92, 102)
Antihypertensive medication			
No medications	632 (89%)	632 (100%)	0 (0%)
Losartan	9 (1.3%)	0 (0%)	9 (11%)
Enalapril	5 (0.7%)	0 (0%)	5 (6.3%)
Amlodipine	8 (1.1%)	0 (0%)	8 (10%)
Metoprolol	4 (0.6%)	0 (0%)	4 (5.0%)
Hydrochlorotiazide	26 (3.7%)	0 (0%)	26 (33%)
Prazosin	1 (0.1%)	0 (0%)	1 (1.3%)
Clonidine	27 (3.8%)	0 (0%)	27 (34%)
Administration schedule			
No medication	632 (89%)	632 (100%)	0 (0%)
Every 24 h	25 (3.5%)	0 (0%)	25 (31%)
Every 12 h	39 (5.5%)	0 (0%)	39 (49%)
Every 8 h	15 (2.1%)	0 (0%)	15 (19%)
Every 6 h	1 (0.1%)	0 (0%)	1 (1.3%)
Days of medication prescribed	0.00 (0.00, 0.00)	0.00 (0.00, 0.00)	4.00 (2.00, 7.00)
Time to surgery (days)	3.0 (2.0, 5.0)	3.0 (2.0, 5.0)	5.0 (2.0, 13.0)
Intravenous fluids (IVF) during hospitalization (mL)	8575 (5500, 16 800)	7800 (5200, 13 650)	24 000 (13 725, 30 000)
Anti‐inflammatories	708 (99%)	629 (100%)	79 (99%)
Opioids	705 (99%)	626 (99%)	79 (99%)
Clinical infection	160 (23%)	113 (18%)	47 (59%)
Thromboembolic disease	7 (1.0%)	4 (0.6%)	3 (3.8%)
Pain by Visual Analogue Pain Scale (VAS)			
Mild	37 (5.2%)	36 (5.7%)	1 (1.3%)
Moderate	347 (49%)	333 (53%)	14 (18%)
Severe	328 (46%)	263 (42%)	65 (81%)
Insomnia	264 (37%)	207 (33%)	57 (71%)
Constipation	179 (25%)	131 (21%)	48 (60%)
Anxiety	117 (16%)	70 (11%)	47 (59%)
Hemoglobin(gr/dL)^c^	11.45 (9.80, 13.00)	11.70 (10.28, 13.10)	8.55 (7.30, 11.58)
Creatinine (mg/dL)^c^	0.89 (0.68, 0.96)	0.88 (0.67, 0.92)	1.02 (0.81, 1.53)

^a^ Dichotomous and categorical variables are presented in absolute numbers and percentages (%); continuous quantitative variables are presented as median with interquartile range (IQR). VAS: Visual Analogue Scale: Mild: 1–3, Moderate: 4–6, and Severe: 7–10.

^b^ Average of the two elevated SBP and DPB measurements at study entry.

^c^ Measured before detection of elevated blood pressure levels.

### 4.1 Outcomes

Table 3 shows the outcomes of hypotension, mortality, length of stay and time to discharge alive in trauma patients according to their exposure to antihypertensives after multiple regression adjustments.

**TABLE 3:** Main outcomes of hypotension, mortality, length of stay and time to discharge alive in trauma patients according to their exposure to antihypertensives after multiple regression adjustments (Appendices 5, 6, and 7).

Outcomes	Estimate		95% CI^a^	*p* value^b^
Hypotension (mean arterial pressure [MAP] < 65 mm Hg)	OR^c^	11.9	5.69 to 26.4	<0.001
Death	OR^c^	5.18	0.79 to 36.6	0.094
Hospital stay (days)	Mean difference	1.5	−0.1 to 3.1	0.066
Time to discharge alive	sHR^d^	0.79	0.59 to 1.06	0.12

^a^ CI, confidence interval.

^b^ Wald estimator for coefficient in regression.

^c^ OR Odds Ratio.

^d^ sHR, subdistribution hazard ratio.

### 4.2 Odds of Hypotension

Patients prescribed antihypertensives had a significantly higher incidence of hypotension than those not prescribed them (OR = 11.9; 95% CI: 5.69–26.4). The association was consistent between antihypertensive use and hypotension classified according to the type of interventions received, and similar results were obtained with the analysis of the model with imputed hemoglobin and creatinine data (OR = 10.9, CI 95% = 5.22; 24.2) (Appendix 5).

### 4.3 Odds of In‐Hospital Death

The adjusted odds of death in patients prescribed antihypertensives were higher than in those who did not receive them (OR = 8.83, CI 95% = 1.05; 85.8). In the analysis performed with complete cases without imputation of hemoglobin and creatinine, the previous association lost magnitude and statistical significance (OR = 5.18, CI 95% = 0.79; 39.6) (Appendix 6).

### 4.4 Length‐of‐Stay

**FIGURE 2:**
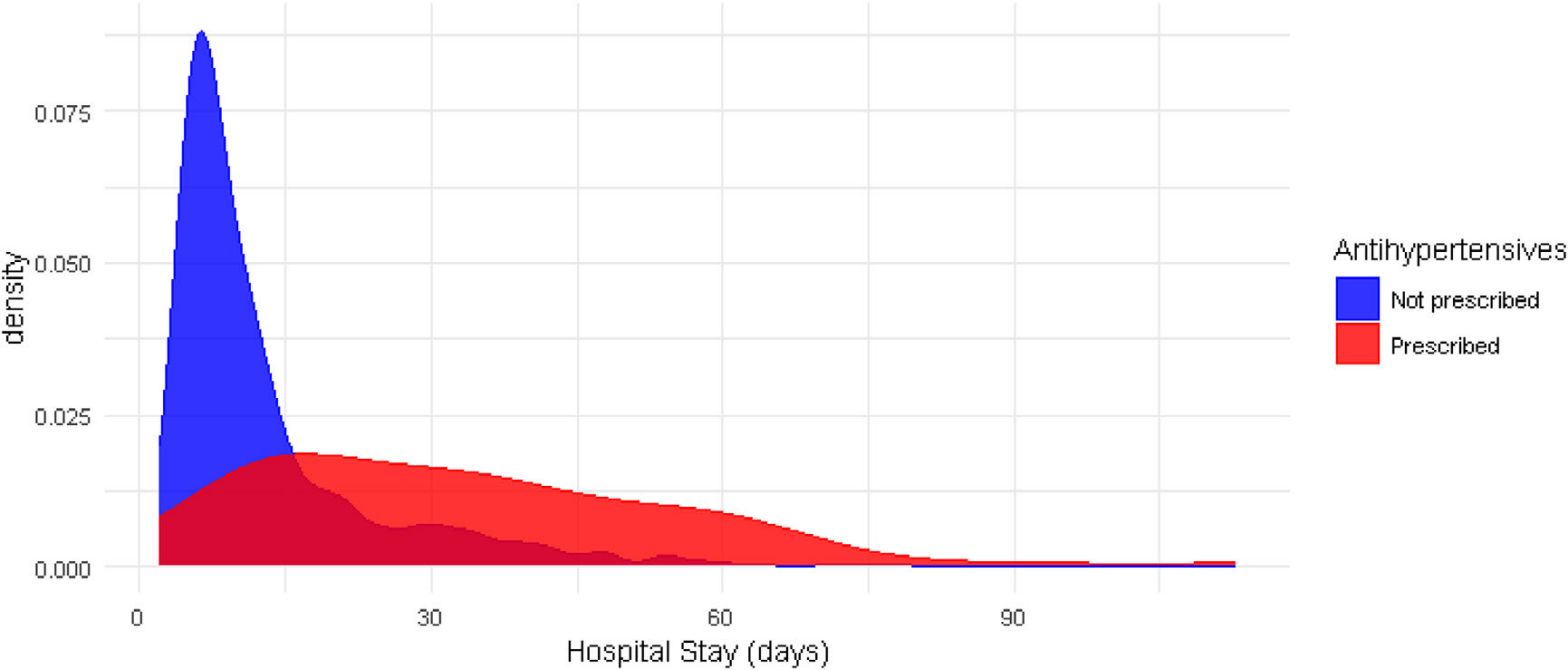
Cumulative distribution of days of stay in patients exposed and not exposed to antihypertensives.

According to the linear model, the prescription of antihypertensives was associated with an increase of 3.4 days (CI 95% = 1.4; 5.5) in hospital stay. Figure 2 shows the distribution of days in the groups without a prescription (median 9 days, interquartile range [IQR] 6–16) and with a prescription (median 31 days, IQR 16–46) of antihypertensives. Using only individuals with stays of less than 60 days, the prescription of antihypertensives was associated with a smaller and non‐statistically significant increase (1.5 days, CI 95%, −0.1 to 3.1) in length of stay (Appendix 7). Also, a Cox regression model was fitted for the time to the event of being discharged alive from the hospital, according to whether or not antihypertensives were prescribed, with in‐hospital death as a competing risk. The hazard of being discharged alive in patients prescribed antihypertensives was lower than in those not prescribed any antihypertensives (HR = 0.72, CI 95% = 0.55; 0.96). When performing the same analysis excluding those with hospital stays longer than 60 days, the estimate of the effect of antihypertensive use on time‐to‐discharge decreased in magnitude and precision (HR 0.79, CI 95%, 0.59–1.06) (Appendix 7).

Sensitivity analysis assuming the 13 patients lost to follow‐up had the extremes of all or none of the outcomes of hypotension and death, did not show significant differences from the previous results (Appendix 8).

## 5 Discussion

In the present study of 712 musculoskeletal trauma patients with elevated blood pressure detected at hospitalization and no history of hypertension, the prescription of oral antihypertensive therapy was associated with an increase in episodes of hypotension requiring medical management. For the outcomes of in‐hospital mortality and length of stay, the magnitude and statistical significance of the associations were reduced when sensitivity analyses were considered for data imputation and stays longer than 60 days, respectively.

Recent studies by Anderson [24, 30] and Rastogi [23] included older patients with a greater number of comorbidities, hospitalized for non‐cardiac medical causes and with a known history of arterial hypertension. In these studies, the effect of in‐hospital intensification of antihypertensive drugs, intravenous or oral, on the occurrence of hypotension, death, acute renal failure (ARF) and cardiovascular events (acute myocardial infarction [AMI] and cardiovascular disease [CVD]) were evaluated. The results of all the studies suggest that in hospitalized older adults with high blood pressure, intensive pharmacological therapy is associated with worse outcomes. The study by Portelli [22] was carried out in a young population with more severe trauma than those considered in our study, with spinal cord trauma and in previously hypertensive patients or with recently diagnosed hypertension (15%). In this study, however, the impact of antihypertensive pharmacological treatment was not evaluated, but rather the occurrence of complications, such as an ICU requirement and the need for mechanical ventilation (MV) in the groups of patients with previous hypertension, with a new diagnosis and without hypertension; showing a worse prognosis in those whose hypertension was diagnosed during hospitalization (Appendix 1).

Our study is the first to evaluate, specifically in patients hospitalized for trauma and without a known history of hypertension, the impact of prescribing antihypertensive medications guided by blood pressure figures. Our results are consistent in replicating the findings of studies found in the literature regarding the risk of arterial hypotension in known hypertensive patients with treatment intensification within the hospital. The effect of prescribing antihypertensives on this outcome was observed in the same direction, but to a substantially greater extent. This could be partially explained by the fact that our patients did not have a previously confirmed diagnosis of hypertension and, therefore, their elevated blood pressure figures could simply have been due to an adaptive and transient phenomenon in response to trauma. Additionally, certain types of frequently prescribed antihypertensive medications, such as Clonidine, Prazosin, or Metoprolol, are often associated with greater decreases in blood pressure levels [21].

The studies in the elderly population previously described failed to confirm an association between antihypertensive treatment and mortality or prolonged hospital stay. Although in our study the effect of pharmacological antihypertensive therapy on these same outcomes did not reach statistical significance in the adjusted analyses, the magnitude of this association does not allow us to rule out a clinically relevant adverse effect of the intervention. Other studies, such as that of Rastogi [23], which included patients with a previous diagnosis of hypertension and evaluated other outcomes such as the development of ARF and the incidence of cardio‐cerebrovascular events (AMI and CVD), also do not seem to favor the indication of exposing hospitalized or acutely ill patients with high blood pressure to antihypertensives for intensive pharmacological therapy purposes.

Our study expands and specifies the population in which the use of in‐hospital antihypertensives could be associated with adverse effects, particularly when patients do not have a previous diagnosis of hypertension. Reactive arterial hypertension to trauma is frequent and may be confused with a diagnosis of hypertension if the definitions of the practice guidelines are applied indiscriminately [21]. These results suggest the need to re‐evaluate local practices related to the decision of whether or not to start antihypertensive medications, as well as the selection of those medications that, according to clinical practice guidelines, have the best evidence profile and an adequate risk/benefit balance for their use [20].

This study has the strength of having included patients with two or more blood pressure readings above 140/90 mm Hg, which, according to the current definition of clinical practice guidelines, could correspond to those patients considered to have Stage 2 hypertension [20, 21]. In this group of patients, where the attending clinician has a reasonable doubt about whether it is a trauma‐reactive hypertension or a previously undiagnosed arterial hypertension, the prescription of antihypertensives occurred in 11.2% (*n* = 80) of the study population, which is approximate equivalent to the prevalence reported for arterial hypertension in the general population [10]. Our study evaluated clinically important outcomes for the patient and the clinician during hospitalization, such as symptomatic hypotension requiring medical intervention. Finally, the multivariate models demonstrated estimates of the robust effect to the imputed data and follow‐up losses.

The limitations of our study are its retrospective nature, with the consequent difficulty inherent in data quality. Patient selection was based on their diagnostic codes, which were recorded by the physician upon admission and by the orthopedic traumatologist after surgery and upon discharge of the patient, and also based on the recording of the participants' blood pressure figures after the initial fluid resuscitation of the trauma. Both selection criteria were applied prior to the evaluation and measurement of exposure and outcome, so the differences between the eligible patients included and those who were not included would be minimal and completely random, so this is not an important source of selection bias. However, the institution where the study was conducted, due to its high complexity, may be a filter that selects patients with a more complex musculoskeletal trauma than those treated in routine practice.

On the other hand, since 2023, the hospital where the study was carried out has made the drug prescription policy more flexible, allowing private patients' medications outside the institutional formulary to be administered with a doctor's order in the medical record. Although we exhaustively searched the daily clinical evolutions, we do not have a reliable record of all the medications potentially administered to the patients included. Since this inaccuracy in the exposure record, although minor, may have occurred before the occurrence of the evaluated outcomes, we cannot rule out a non‐differential measurement bias, which would generate a dilution of the effect of antihypertensives on the outcomes of interest. However, since patients with a previous diagnosis of arterial hypertension were excluded, it seems unlikely that oral medications were administered outside the electronic medical record.

Another limitation, as in all non‐experimental research, is the residual confounding caused by variables that cannot be measured or those that are measured inaccurately. Although DAGs were constructed to identify and adjust for confounding variables in regression models, the accurate measurement of certain variables, such as smoking history and alcohol intake, is impossible to determine from electronic medical records alone.

Finally, we do not have electronic records of outpatient appointments or new hospital admissions that would allow us to measure the outcomes of patients who were counter‐referred, those who were discharged voluntarily, or those treated in home‐health programs (13 in total, 1.8% of the study cohort). These losses to follow‐up, however, have similar baseline characteristics to those of the cohort analyzed, and sensitivity analyses suggest that they do not affect the results obtained (Appendix 8).

Despite having extensive literature on the benefits of controlling blood pressure levels in outpatients, there is uncertainty about the best approach for hypertension in hospitalized patients, and much more about the diagnostic and therapeutic approach to reactive hypertension associated with trauma. A systematic review analyzed 14 clinical practice guidelines showed that there are no recommendations in this regard [35]. The present results support the scientific statement of the AHA, which recognizes that, with the current state of evidence, treating asymptomatic hypertension in hospitalized patients should be the exception rather than the rule and that studies are needed to clarify whether there is clinical benefit in this practice [36]. Consequently, the prescription of de novo antihypertensives in the hospital setting should be very cautious and guided by the individual needs of each patient [37].

## Author Contributions

Carlos José Atencia made substantial contributions to: The conception and design of the study, or acquisition of data, or analysis and interpretation of data, drafting the article or revising it critically for important intellectual content and final approval of the version to be submitted. Fabian Jaimes made substantial contributions to: The conception and design of the study, analysis and interpretation of data, drafting the article or revising it critically for important intellectual content and final approval of the version to be submitted.

## Ethics Statement

The protocol was approved by the Bioethics Committee of the University of Antioquia Faculty of Medicine and the San Vicente Fundación Hospital (Approval Act No. 051 from June 22, 2023, and Approval Act No. 30–2023 from October 13, 2023). The research was conducted in accordance with the Declaration of Helsinki 2008 and Colombian regulations for research. Consent to participate and Consent for publication: According to National and International regulations, laws, and due to the nature of this study, informed consent to participate and publish was not required.

## Conflicts of Interest

The authors declare no conflicts of interest.

## Disclaimers

The author's state that the views expressed in the submitted article are their own and not an official position of the institution or funder.

## Permission to Reproduce Material

All material is originally produced by authors.

## R Code Availability

Statistical analysis was sent (Supporting file 2).

## Supporting information




**Supporting file 1**: DAGs in Appendix based on ref [38‐52].

**Supporting file 2**: jch70210‐sup‐0002‐SuppMat.docx

## Data Availability

The data that support the findings of this study are openly available in Mendeley at https://data.mendeley.com/datasets/k6pjfjn4vv/1, reference number DOI: 10.17632/k6pjfjn4vv.1.
