# Supplementary material for: Effect of Antihypertensive Treatment on Hypotension, Mortality and Length of Stay in Orthopedic Trauma and First Detected High Blood Pressure Adults in a Large Urban Hospital: A Retrospective Cohort Study
Source: J Clin Hypertens (Greenwich). 2026 Jan 30;28(2):e70210. doi: 10.1111/jch.70210 (PMC12856957; doi:10.1111/jch.70210)
Supplement: Supplementary file 1 — Supporting file 1: DAGs in Appendix based on ref [38‐52]. [file JCH-28-e70210-s001.docx]

**Appendix 1**

A search of the last 10 years (2012 – 2022) was performed in Medline through Pubmed, using filters for publication type (“clinical study”) and using the MESH and truncated terms “Hypertension,” “Hypertens,” AND “Trauma” that were included in the title of the article or in its structured abstract. A total of 415 results were detected, of which 205 articles were not related to the topic. Of the remaining 210 articles, 57 were about the treatment of hypertension in acute settings (emergencies and hypertensive emergencies); 45 were related to arterial hypertension secondary to renal trauma; 32 were related to hypertension secondary to intra-abdominal trauma; 28 were related to arterial hypertension secondary to encephalocranial, spinal cord and ocular trauma; 27 articles referred to the treatment of hypertension in pregnant women; 19 evaluated sequelae of arterial hypertension secondary to traumatic amputation of limbs; and 10 were related to arterial hypertension secondary to trauma with aortic involvement. Of the related articles, six evaluated the prevalence of arterial hypertension in hospitalized trauma patients and four of these were chosen for discussion (22,23,37–40).

A search over the past 10 years of EMBASE conducted using filters for publication type to randomized clinical trials and controlled clinical trials and using truncated descriptors of “Hypertens” AND “Injury” yielded 870 results, of which an individual review of the structured abstract and title was performed. Of all the articles, 339 did not contain the search keywords or were not research on living humans, and 265 articles were not related to the topic. Of the 266 related articles, 91 were about the treatment of hypertension in acute settings (emergencies and hypertensive emergencies); 41 were related to arterial hypertension secondary to renal trauma; 39 were related to hypertension secondary to renal trauma; 35 corresponded to arterial hypertension secondary to encephalocranial, spinal cord and ocular trauma; 24 articles referred to the treatment of hypertension in pregnant women; 21 evaluated sequelae of arterial hypertension secondary to traumatic amputation of limbs; and 18 related to arterial hypertension secondary to trauma with aortic involvement. Of the 17 articles selected, nine of them were duplicated in the Medline search (22,23) and eight evaluated the phenomenon of arterial hypertension in hospitalized patients. Five were observational studies analyzing the treatment of hypertension in hospitalized subjects and seven of them specifically in the context of musculoskeletal trauma, so these four were chosen for analysis and discussion.

In the Cochrane library, clinical trials, randomized trials, and meta-analyses were searched using “Antihypertensive drugs/Antihypertensive medications,” “inpatients,” and “trauma”; 53 results were obtained. When reviewed individually, none of them were related to the topic or objectives of this research.

Finally, in a search in Scielo that used the health sciences descriptors (DECS) in Spanish “Hypertension” and “Trauma,” only one article was obtained where the point prevalence of arterial hypertension in hospitalized patients was measured (9).

**Comparison of population characteristics, interventions, outcomes, designs and analysis of the four studies reported in the literature**

| **Characteristic** | **Atencia (pending, 2024)** | **Anderson (J.Int.Med, 2018)** | **Anderson (J.Int.Med 2023)** | **Rastogi (JAMA, 2022)** | **Portelli (JSR,**  **2022)** |
| --- | --- | --- | --- | --- | --- |
| Population | n = 700;  age: 37 years old; men: 77%; mixed race: 99% | n = 4056; age: 77 years old; men: 97.7% | n = 66140; age: 74.4 years old; men: 97.5%;  white race: 75.9% | n = 9040; age: 65 years old; men: 59%; white race: 69.7% | n = 1906; age: 34 years old; men: 81%; white race: 53% |
| Comorbidity | Musculo-skeletal trauma, obesity, alcohol | Non-cardiac medical or surgical disease | Non-cardiac medical or surgical disease | Non-cardiac medical or surgical disease | Trauma to the thorax and abdomen included |
| Intervention/ Exposure | Oral medications | Oral medications and IV | Oral medications and IV | Oral medications and IV | No medications |
| Hospital stay | 9 (6-16) vs 31(16-46) | 6.7 vs 7.1 | - | 3.56 vs 3.66 | 3 vs 6 |
| Hypotension | OR 11.9 (5.6-26.4) | AES* Hazard Ratio  (HR) 1.41 (1.06-1.88) | OR 1.22 (1.15-1.30) | - | - |
| Death | 1.1% vs 8.0%  Subdistribution Hazard Ratio (sHR) 0.79 (0.59-1.06) | HR 0.84 (0.62-1.12) | OR 1.11 (0.91-1.37) | - | 3% vs 5% |
| Other outcomes | - | Readmission  HR 1.23 (1.07-1.42)  AMI  HR 1.18 (0.99-1.40) | Acute Renal Failure (ARF)  OR 1.43 (1.29-1.58)  Cerebrovascular Disease (CVD) 0.2% vs 0.1% | ARF 7.9% VS 10.3%  (CVD 0.1% vs 0.1%  AMI 0.6% vs 1.2% | MV** 38% vs 17% |
| Design | Retrospec. Cohort | Retrospec. Cohort | Retrospec. Cohort | Retrospec. Cohort | Retrospec. Cohort |
| Adjustment method | Regression | Propensity Score Matching (PSM) | PSM | PSM | PSM |

*Adverse effect survival ** Mechanical ventilation

**Appendix 2**

Chapter XIX: Injuries, poisoning and certain other consequences of external causes (S00–T98).

**Injuries of shoulder and arm (S40–S49):** S40: Superficial injury of shoulder and arm; S41: Wound of shoulder and arm; S42: Fracture of shoulder and arm; S43: Dislocation, sprain and strain of joints and ligaments of shoulder girdle; S44: Injury of nerves at shoulder and arm; S45: Injury of blood vessels at shoulder and arm; S46: Injury of tendon and muscle at shoulder and arm; S47: Crush injury of shoulder and arm; S48: Traumatic amputation of shoulder and arm; S49: Other and unspecified injuries of shoulder and arm.

**Injuries of forearm and elbow (S50–S59):** S50: Superficial injury of forearm and elbow; S51: Wound of forearm and elbow; S52: Fracture of forearm; S53: Dislocation, sprain and strain of joints and ligaments of elbow; S54: Injury of nerves at forearm level; S55: Injury of blood vessels at forearm level; S56: Injury of tendon and muscle at forearm level; S57: Crush injury of forearm; S58: Traumatic amputation of forearm; S59: .

**Other and unspecified injuries of forearm; Injuries of wrist and hand (S60–S69):** S60: Superficial injury of wrist and hand; S61: Wound of wrist and hand; S62: Fracture at wrist and hand; S63: Dislocation, sprain and strain of joints and ligaments at wrist and hand; S64: Injury of nerves at wrist and hand; S65: Injury of blood vessels at wrist and hand; S66: Injury of tendon and muscle at wrist and hand; S67: Crush injury of wrist and hand; S68: Traumatic amputation of wrist and hand; S69: Other and unspecified injuries of wrist and hand.

**Injuries of hip and thigh (S70–S79):** S70: Superficial injury of hip and thigh; S71: Wound of hip and thigh; S72: Fracture of femur; S73: Dislocation, sprain and strain of hip joint and ligaments; S74: Injury of nerves at hip and thigh; S75: Injury of blood vessels at hip and thigh; S76: Injury of tendon and muscle at hip and thigh; S77: Crush injury of hip and thigh; S78: Traumatic amputation of hip and thigh; S79: Other and unspecified injuries of hip and thigh.

**Injuries of knee and leg (S80–S89)** S80: Superficial injury of leg; S81: Wound of leg; S82: Fracture of leg, including ankle; S83: Dislocation, sprain and strain of joints and ligaments of knee; S84: Injury of nerves at leg level; S85: Injury of blood vessels at leg level; S86: Injury of tendon and muscle at leg level; S87: Crush injury of leg; S88: Traumatic amputation of leg; S89: Other and unspecified injuries of leg.

**Injuries of ankle and foot (S90–S99)**: S90: Superficial injury of ankle and foot; S91: Wound of ankle and foot; S92: Fracture of foot, except ankle; S93: Dislocation, sprain and strain of joints and ligaments of ankle and foot; S94: Injury of nerves at foot and ankle level; S95: Injury of blood vessels of foot and ankle level; S96: Injury of tendon and muscle of foot and ankle level; S97: Crush injury of foot and ankle; S98: Traumatic amputation of foot and ankle; S99: Other and unspecified injuries of foot and ankle.

**Injuries involving multiple body regions (T00–T07)**: T00: Superficial injuries involving multiple body regions; T01: Wounds involving multiple body regions; T02: Fractures involving multiple body regions; T03: Dislocations, sprains and strains involving multiple body regions; T04: Crush injuries involving multiple body regions; T05: Traumatic amputations involving multiple body regions; T06: Other injuries involving multiple body regions, not elsewhere classified; T07: Multiple injuries, unspecified.

**Injuries of unspecified part of trunk, limb or body region (T08–T14)**: T08: Fracture of vertebral column, level unspecified; T09: Other injuries of vertebral column and trunk, level unspecified; T10: Fracture of upper limb, level unspecified; T11: Other injuries of upper limb, level unspecified; T12: Fracture of lower limb, level unspecified; T13: Other injuries of lower limb, level unspecified; T14: Injury of unspecified body regions.

**Appendix 3**

**Operational table of variables: Exposure**

| **Variable** | **Nature of variable** | **Level of Measurement** | **Operational Definition** | **Values** |
| --- | --- | --- | --- | --- |
| Antihypertensive treatment | Qualitative | Nominal | Patient who records any antihypertensive medication in the electronic Kardex for more than 48 hours. | 1: Yes  0: No |
| Type of antihypertensive treatment | Qualitative | Nominal | Distribution according to type of antihypertensive used, time and at any dose for more than 48 hours. | 1. Angiotensin converting enzyme inhibitors (ACEIs) 2. Beta blockers 3. Aldosterone receptor antagonists 4. Calcium channel blockers 5. Alpha 1 adrenergic blockers 6. Alpha 2 receptor agonist 7. Minoxidil 8. Diuretics |
| Prescribed daily dose (PDD) | Qualitative | Nominal | Calculated from the WHO formula (46) | Number of PDD of each medication |

**Operational table of variables: Outcomes**

| **Variable** | **Nature of variable** | **Level of Measurement** | **Operational Definition** | **Values** |
| --- | --- | --- | --- | --- |
| Low blood pressure | Quantitative | Continuous | Mean arterial pressure (MAP) during hypotension after initiation of antihypertensives or the lowest recorded during hospitalization | 0 – 100 mmHg |
| Hypotension (MAP < 65 or the lowest recorded) requiring medical intervention 48 hours after starting medication | Qualitative | Dichotomous | Intervention such as: suspension of antihypertensives, need for intravenous fluids or elevation of lower limbs (Trendelenburg position) and need for vasopressor or transfer to Special Care Unit SCU/ICU | Suspension of medication  1: Yes  0: No |
|  |  |  |  | Need for liquids or elevation of extremities  1: Yes  0: No |
|  |  |  |  | Start vasopressor or transfer to SCU/ICU  1: Yes  0: No |
| Time-to-discharge alive | Qualitative | Dichotomous with time to event | Patient who is discharged due to death or discharged alive. | 1: Yes  0: No |
| Hospital stay | Quantitative | Counting | Days of hospital stay, measured from the date of admission until discharge, transfer or death | # of days |

**Operational table of variables: Possible confounders**

| **Variable** | **Nature of the variable** | **Level of measurement of the variable** | **Operational definition** | **Values** |
| --- | --- | --- | --- | --- |
| Age | Quantitative | Continuous | Distribution according to patient age in years | Age in years recorded in the Clinical History |
| Sex | Qualitative | Dichotomous | Patient gender | 1: Male  0: Female |
| Race or ethnicity | Qualitative | Nominal, dichotomous | Distributed according to medical criterion | 1: Afro-Colombian  0: Non-Afro-Colombian |
| Fracture | Qualitative | Nominal | Anatomical site or bone where the fracture is located | Clavicle, shoulder, humerus, elbow, radius or ulna, wrist, hand, pelvis, hip, femur, knee, tibia - fibula, ankle, foot |
| Classification of the fracture (26) | Qualitative | Ordinal | According to the Gustillo-Anderson (GA) Classification of Fractures | 1. Closed  2. Open Grade 1 –Opening of the dermis <1cm  3. Open Grade 2 –  Opening of the dermis of 1-10 cm  4. Open Grade 3 –  Opening > 10 cm with tissue damage |
| Revised Trauma Score (RTS) (47) | Quantitative | Continuous | Distribution according to the RTS physiological score (uses the Glasgow Coma Scale (GCS), systolic blood pressure (SBP) and respiratory rate (RR)) | 0 to12  (RTS= 0,9368*GCS + 0.7326*PAS + 0.2908* RR) – 3.15 |

| Intravenous fluids (IVF) prior to detection of elevated blood pressure figures | Quantitative | Continuous | Amount of intravenous fluids administered after trauma and started prior to detection of elevated blood pressure levels in cubic centimeters | Cubic centimeters (cc) |
| --- | --- | --- | --- | --- |
| Surgical procedure prior to the detection of high blood pressure figures | Qualitative | Nominal, dichotomous | Distribution according to time from hospitalization to surgery | 1: Control surgery with braces, or definitive reduction surgery and internal or external fixation or skeletal traction.  0: No surgery (use of splints, casts, bandages) |
| Use of non-steroidal anti-inflammatory analgesics (NSAIDs) prior to detection of elevated blood pressure levels | Qualitative | Nominal, dichotomous | Distribution according to use of analgesics of any dose | 1: Yes  0: No |
| Use of opioid analgesics prior to detection of elevated blood pressure levels | Qualitative | Nominal, dichotomous | Distribution according to use of analgesics of any dose | 1: Yes  0: No |
| Infection/sepsis during hospitalization | Qualitative | Nominal, dichotomous | Defined as sepsis, surgical- site infection or other infection (phlebitis, urinary tract infection) | 1: Yes  0: No |
| Pain reported by the patient prior to the detection of elevated blood pressure levels | Qualitative | Ordinal | WHO Visual Analogue Pain (VAS) Scale | 1: Mild (VAS 1-3)  2: Moderate (VAS 4-6)  3: Severe (VAS 7-10) |
| Insomnia reported by the patient prior to the detection of elevated blood pressure levels | Qualitative | Nominal, dichotomous | Inability to fall asleep or stay asleep reported to nursing or the doctor | 1: Yes  0: No |

| Constipation (or laxative prescription medication record) reported by the patient prior to detection of elevated blood pressure levels | Qualitative | Nominal, dichotomous | Any change in bowel habits referred to the nurse or doctor | 1: Yes  0: No |
| --- | --- | --- | --- | --- |
| Anxiety or stress prior to detecting high blood pressure levels | Qualitative | Nominal, dichotomous | Consultation with Psychiatry or Psychology during hospitalization with diagnosis or symptoms of anxiety or stress | 1: Yes  0: No |
| Hemoglobin prior to detection of high blood pressure figures | Quantitative | Continuous | Hemoglobin figure | 3 gr/dl to 18gr/dl |
| Creatinine prior to detection of elevated blood pressure figures | Quantitative | Continuous | Creatinine figure | Greater than 0.2 mg/dl |
| Smoking History | Qualitative | Nominal, dichotomous | Distribution by smoking or non-smoking status | 1: Yes  0: No |
| Obesity | Quantitative | Interval | Body mass index (BMI) > 25 kg/m2 or by any anthropometric index reported by nursing or nutrition | 1: Yes  0: No |
| Alcoholism | Qualitative | Nominal, dichotomous | History or no history of alcohol consumption at the time of hospitalization greater than 80 gr per day in men and 40 gr per day in women | 1: Yes  0: No |

| Diabetes Mellitus | Qualitative | Nominal, dichotomous | History or no history of Diabetes Mellitus at the time of hospitalization; glycated hemoglobin greater than 6.5% | 1: Yes  0: No |
| --- | --- | --- | --- | --- |
| Chronic Kidney Failure (CKF) | Qualitative | Nominal, dichotomous | History or no history of CKF at the time of hospitalization or creatinine greater than 1.5 g/dl or filtration rate less than 60 cc/min | 1: Yes  0: No |
| History of acute myocardial infarction | Qualitative | Nominal, dichotomous | History or no history of acute myocardial infarction, elevated troponins, coronary angiography or Q waves in inferior leads on ECG | 1: Yes  0: No |
| Cerebrovascular disease | Qualitative | Nominal, dichotomous | History or no history of cerebrovascular disease at the time of hospitalization; previous CT scan with encephalomalacia or acute hemiparesis | 1: Yes  0: No |

**Appendix 4**

**Sample size calculation**

For the calculation of the sample size, a ratio of 1:3 is expected between hypertensive patients with antihypertensive treatment and without treatment, according to the studies available in the literature (22,23,41), so, for this study, we stuck to this ratio for the exposure.

For the outcome of days of hospital stay, the studies reviewed (22,23) suggested that a difference of three days with a standard deviation of one day could occur with the use of antihypertensive therapy. For a difference in means, the following formula was used to calculate the sample size:

$$2N = \frac{{4\left( Z\alpha+Z\beta\right)}^{2} \sigma^{2}}{\delta^{2}}$$

Where:

Zα is 1.96, the critical region for a significance of 5%;

Zβ is 1.282, which is the critical region for a power of 80%;

Sigma (σ) is the standard deviation (SD) of the mean;

Delta (δ) is the mean of the change expected to be detected; in this case, three days.

According to the above, the necessary sample size would be 340 subjects in total.

For the mortality outcome, only one study (22) reported an absolute difference of 5% (from 2% to 7%), according to the use of antihypertensive drugs. For a difference in proportions, the calculation was based on the following formula:

$$2N=\frac{2\left[ Z\alpha\sqrt[2]{p\left( 1-p \right)}+Z\beta\sqrt[2]{\mathrm{Pc}\left( 1-Pc \right)+Pi\left( 1-Pi \right)} \right]^{2}}{\left( Pc-Pi \right)^{2}}$$

Zα is 1.96, the critical region for a significance of 5%;

Zβ is 1.282, which is the critical region for a power of 80%;

Pc is the proportion in the unexposed group; in this case, 2%

Pi is the proportion in the exposed group; in this case, 7%

P is the average of the proportions (Pc + Pi)/2; in this case, 3.5%

According to the above, the necessary sample size would be 700 subjects.

For the outcome of hypotension, only one study (23) reported a reduction in both groups of 2.5 mm Hg of the blood pressure figures, but did not report results in terms of proportions of arterial hypotension in the groups. Therefore, we will use the calculation made for mortality. The estimated sample size of 700 subjects in total was chosen, with a ratio of 2:1 of those exposed to antihypertensive drugs and those not exposed.

**Appendix 5**

**DAG 1: Hypotensive outcome requiring medical treatment** (4,18,36–43,48,49)


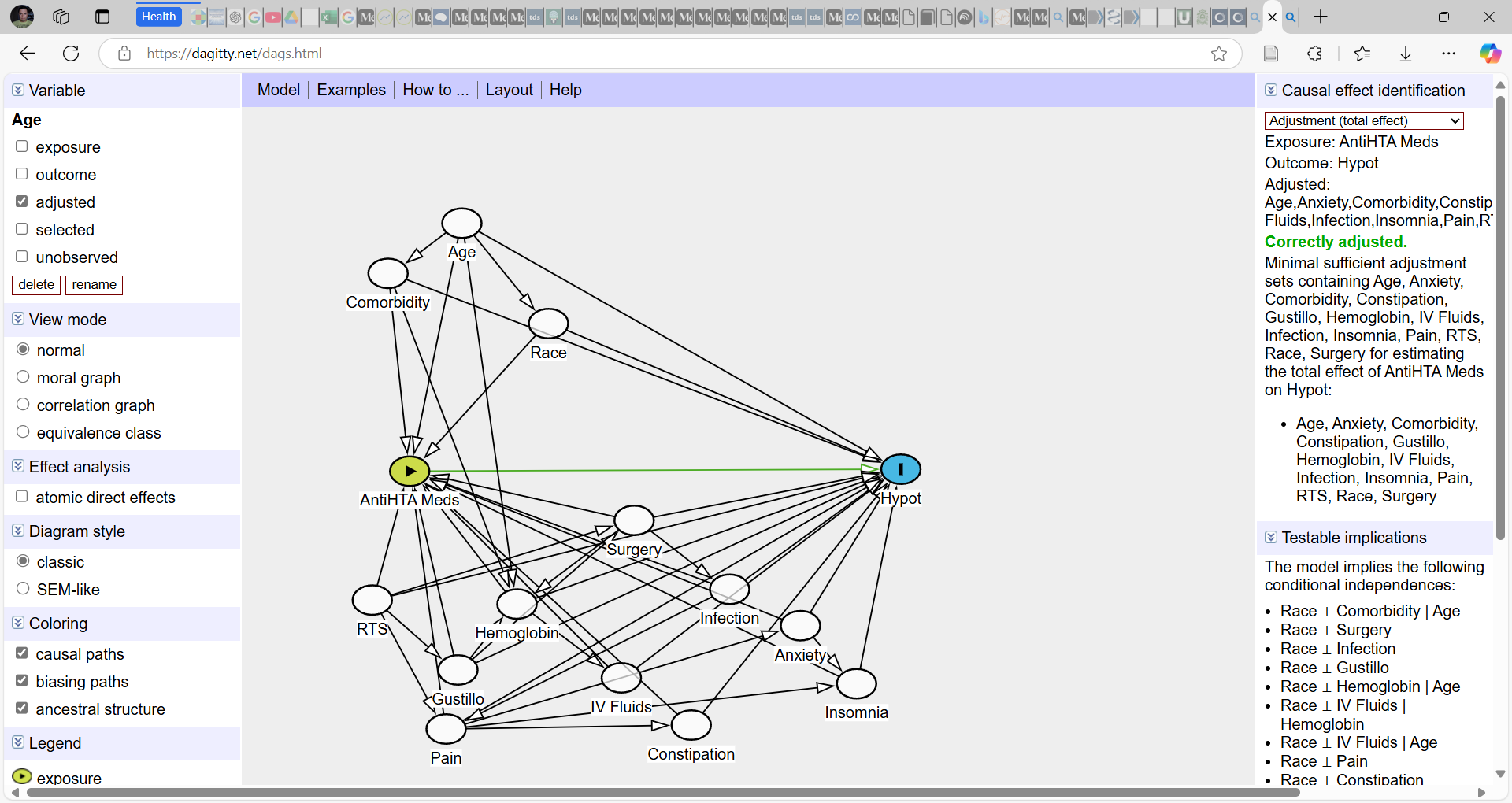


**DAG 1 Table: Multiple logistic regression model for the outcome of**

**arterial hypotension (imputed hemoglobin and creatinine)**

| **Variables** | **OR**^1^ | **CI 95%**^1^ | **p-value** |
| --- | --- | --- | --- |
| Antihypertensives |  |  |  |
| Not prescribed | 1.00 | — |  |
| Prescribed | 11.9 | 5.69, 26.4 | <0.001 |
| Race |  |  |  |
| Mixed Race | 1.00 | — |  |
| Black | 0.19 | 0.01, 1.44 | 0.2 |
| Age (years) | 1.01 | 1.00, 1.02 | 0.2 |
| Myocardial infarction (AMI) |  |  |  |
| No AMI | 1.00 | — |  |
| AMI | 1.20 | 0.02, 32.9 | >0.9 |
| Liquor consumption |  |  |  |
| No consumption | 1.00 | — |  |
| Consumption | 0.67 | 0.39, 1.14 | 0.14 |
| History of obesity |  |  |  |
| Absent | 1.00 | — |  |
| Present | 2.40 | 0.83, 6.88 | 0.10 |
| Diabetes Mellitus Type 1 or 2 |  |  |  |
| No Diabetes | 1.00 | — |  |
| Diabetes | 1.48 | 0.54, 3.81 | 0.4 |
| Cerebrovascular Disease (CVD) |  |  |  |
| No CVD | 1.00 | — |  |
| CVD | 1.44 | 0.03, 58.8 | 0.8 |
| Pain by VAS | 1.13 | 0.74, 1.72 | 0.6 |
| Intravenous fluids (mL) | 1.00 | 1.00, 1.00 | 0.036 |
| Hemoglobin (gr/dL) | 0.84 | 0.76, 0.93 | <0.001 |
| Anxiety |  |  |  |
| Absent | 1.00 | — |  |
| Present | 1.05 | 0.56, 1.92 | 0.9 |
| Time to surgery (days0 | 0.99 | 0.95, 1.01 | 0.5 |
| Trauma Score (RTS) | 0.99 | 0.97, 1.00 | 0.13 |
| Weight (Kg) | 0.97 | 0.94, 1.00 | 0.026 |
| Cigarette, tobacco or vaper smoker |  |  |  |
| No | 1.00 | — |  |
| Yes | 1.21 | 0.73, 2.04 | 0.5 |
| Insomnia |  |  |  |
| Absent | 1.00 | — |  |
| Present | 1.43 | 0.83, 2.43 | 0.2 |
| Clinical infection |  |  |  |
| Absent | 1.00 | — |  |
| Present | 2.19 | 1.22, 3.92 | 0.008 |
| Constipation |  |  |  |
| Absent | 1.00 | — |  |
| Present | 0.52 | 0.28, 0.93 | 0.030 |
| Gustillo-Anderson (GA) Classification |  |  |  |
| Closed | 1.00 | — |  |
| GA1 | 1.85 | 0.99, 3.44 | 0.052 |
| GA2 | 1.28 | 0.66, 2.43 | 0.5 |
| GA3 | 1.59 | 0.84, 3.00 | 0.15 |
| ^1^OR = Odds Ratio, CI = Confidence Interval, GA= Gustillo-Anderson. | | | |

**DAG 1 Table: Multiple logistic regression model for the outcome of**

**hypotension without imputation in creatinine and hemoglobin**

**variables**

| **Variables** | **OR**^1^ | **CI 95%**^1^ | **p-value** |
| --- | --- | --- | --- |
| Antihypertensives |  |  |  |
| Not prescribed | 1.00 | — |  |
| Prescribed | 10.9 | 5.22, 24.2 | <0.001 |
| Race |  |  |  |
| Mixed Race | 1.00 | — |  |
| Black | 0.20 | 0.01, 1.51 | 0.2 |
| Age (years) | 1.01 | 0.99, 1.02 | 0.2 |
| Myocardial infarction (AMI) |  |  |  |
| No AMI | 1.00 | — |  |
| AMI | 1.23 | 0.03, 31.0 | >0.9 |
| Liquor consumption |  |  |  |
| No consumption | 1.00 | — |  |
| Consumption | 0.57 | 0.32, 1.00 | 0.053 |
| History of obesity |  |  |  |
| Absent | 1.00 | — |  |
| Present | 2.14 | 0.74, 6.16 | 0.2 |
| Diabetes Mellitus Type 1 or 2 |  |  |  |
| No Diabetes | 1.00 | — |  |
| Diabetes | 1.69 | 0.59, 4.62 | 0.3 |
| Cerebrovascular Disease (CVD) |  |  |  |
| No CVD | 1.00 | — |  |
| CVD | 1.15 | 0.03, 40.2 | >0.9 |
| Pain by VAS | 1.06 | 0.68, 1.65 | 0.8 |
| Intravenous fluids (mL) | 1.00 | 1.00, 1.00 | 0.079 |
| Hemoglobin (gr/dL) | 0.83 | 0.75, 0.92 | <0.001 |
| Creatinine | 1.31 | 0.90, 1.89 | 0.12 |
| Anxiety |  |  |  |
| Absent | 1.00 | — |  |
| Present | 1.14 | 0.61, 2.10 | 0.7 |
| Time to surgery (days0 | 0.99 | 0.95, 1.03 | 0.5 |
| Trauma Score (RTS) | 0.98 | 0.97, 1.00 | 0.10 |
| Weight (Kg) | 0.97 | 0.94, 1.00 | 0.030 |
| Cigarette, tobacco or vaper smoker |  |  |  |
| No | 1.00 | — |  |
| Yes | 1.36 | 0.79, 2.35 | 0.3 |
| Insomnia |  |  |  |
| Absent | 1.00 | — |  |
| Present | 1.21 | 0.69, 2.11 | 0.5 |
| Clinical infection |  |  |  |
| Absent | 1.00 | — |  |
| Present | 1.91 | 1.05, 3.48 | 0.035 |
| Constipation |  |  |  |
| Absent | 1.00 | — |  |
| Present | 0.57 | 0.31, 1.04 | 0.070 |
| Gustillo-Anderson (GA) Classification |  |  |  |
| Closed | 1.00 | — |  |
| GA1 | 2.17 | 1.12, 4.22 | 0.022 |
| GA2 | 1.22 | 0.62, 2.38 | 0.6 |
| GA3 | 1.48 | 0.76, 2.89 | 0.2 |
| ^1^OR = Odds Ratio, CI = Confidence Interval | | | |

**DAG 1 Table: Multiple logistic regression model for the outcome of**

**hypotension with outcome subtypes (only the estimator**

**of the effect of exposure is included, but is adjusted for all**

**confounding variables from DAG 1)**

**For hypotension requiring intravenous fluids**

| **Variable** | **OR**^1^ | **CI 95%**^1^ | **p-value** |
| --- | --- | --- | --- |
| Antihypertensives |  |  |  |
| Not prescribed | — | — |  |
| Prescribed | 31.5 | 13.2, 80.9 | <0.001 |

**For hypotension with the need to stop medication**

| **Variable** | **OR**^1^ | **CI 95%**^1^ | **p-value** |
| --- | --- | --- | --- |
| Antihypertensives |  |  |  |
| Not prescribed | — | — |  |
| Prescribed | 28.8 | 11.1, 81.9 | <0.001 |

**For hypotension requiring vasopressors**

| **Variable** | **OR**^1^ | **CI 95%**^1^ | **p-value** |
| --- | --- | --- | --- |
| Antihypertensives |  |  |  |
| Not prescribed | — | — |  |
| Prescribed | 3.13 | 1.08, 9.39 | 0.037 |

**APPENDIX 6**

**DAG 2: Outcome probability of in-hospital death (4,18,36,40,41,50–54)**


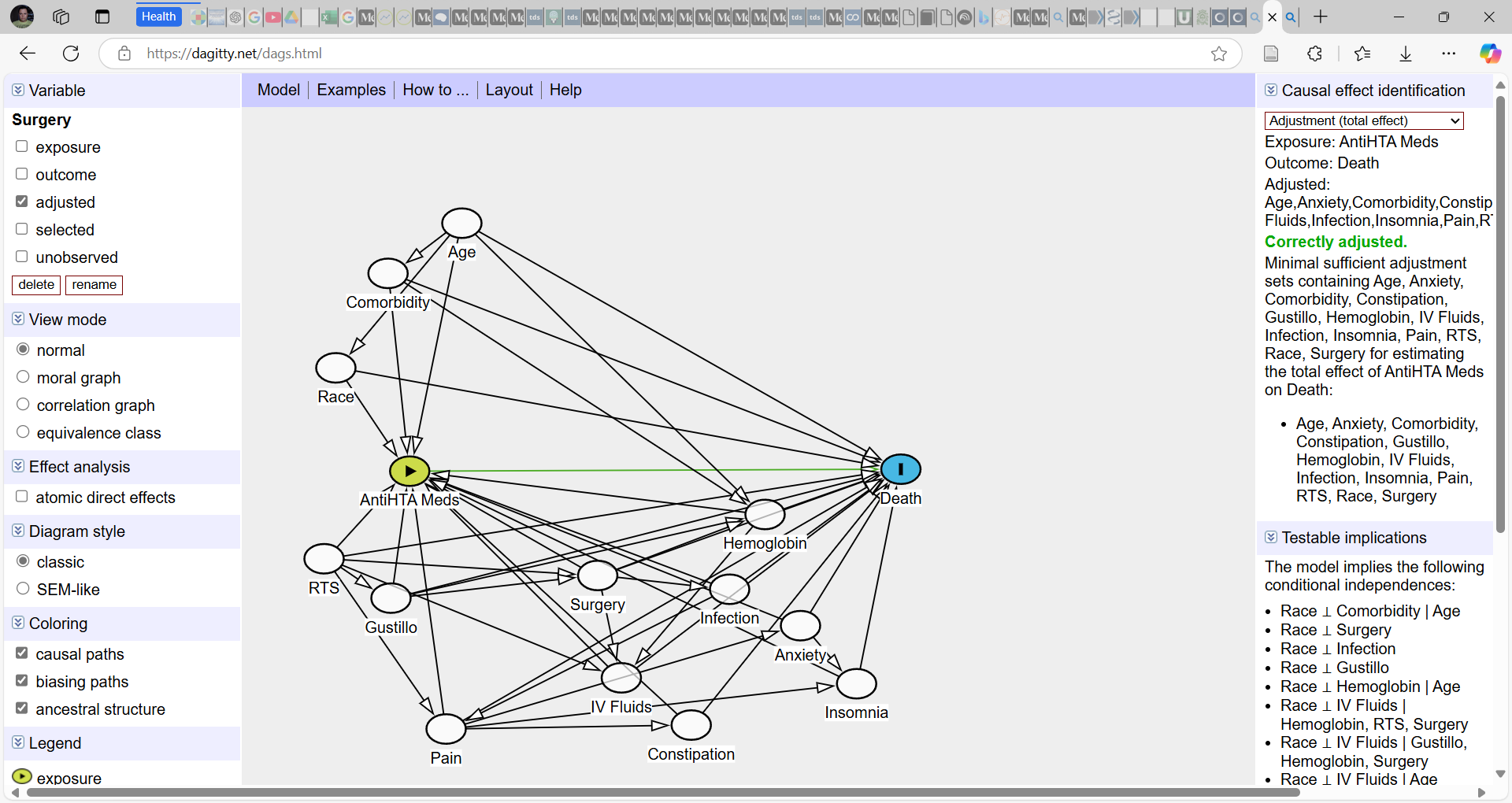


**DAG 2 Table: Logistic regression model for probability of in-hospital death (imputed**

**hemoglobin and creatinine)**

| **Model Variables** | **OR**^1^ | **CI 95%**^1^ | **p-value** |
| --- | --- | --- | --- |
| Antihypertensives |  |  |  |
| Not prescribed | 1.00 | — |  |
| Prescribed | 8.83 | 1.05, 85.8 | 0.049 |
| Race |  |  |  |
| Mixed Race | 1.00 | — |  |
| Black | 0.00 |  | >0.9 |
| Age (years) | 1.06 | 1.01, 1.13 | 0.045 |
| Myocardial infarction (AMI) |  |  |  |
| No AMI | 1.00 | — |  |
| AMI | 0.00 |  | >0.9 |
| Liquor consumption |  |  |  |
| No consumption | 1.00 | — |  |
| Consumption | 1.31 | 0.21, 9.48 | 0.8 |
| History of obesity |  |  |  |
| Absent | 1.00 | — |  |
| Present | 1.98 | 0.06, 60.7 | 0.7 |
| Diabetes Mellitus Type 1 or 2 |  |  |  |
| No Diabetes | 1.00 | — |  |
| Diabetes | 1.63 | 0.10, 16.0 | 0.7 |
| Cerebrovascular Disease (CVD) |  |  |  |
| No CVD | 1.00 | — |  |
| CVD | 0.00 |  | >0.9 |
| Pain by VAS | 0.55 | 0.11, 2.72 | 0.5 |
| Intravenous fluids (mL) | 1.00 | 1.00, 1.00 | 0.12 |
| Hemoglobin (gr/dL) | 0.87 | 0.57, 1.31 | 0.5 |
| Anxiety |  |  |  |
| Absent | 1.00 | — |  |
| Present | 10.2 | 1.09, 138 | 0.055 |
| Time to surgery (days0 | 0.87 | 0.64, 1.01 | 0.3 |
| Trauma Score (RTS) | 0.89 | 0.81, 0.95 | 0.002 |
| Weight (Kg) | 1.01 | 0.92, 1.12 | 0.8 |
| Cigarette, tobacco or vaper smoker |  |  |  |
| No | 1.00 | — |  |
| Yes | 2.88 | 0.47, 22.9 | 0.3 |
| Insomnia |  |  |  |
| Absent | 1.00 | — |  |
| Present | 0.42 | 0.02, 4.84 | 0.5 |
| Clinical infection |  |  |  |
| Absent | 1.00 | — |  |
| Present | 0.53 | 0.03, 6.83 | 0.6 |
| Constipation |  |  |  |
| Absent | 1.00 | — |  |
| Present | 3.77 | 0.39, 40.4 | 0.2 |
| Gustillo-Anderson (GA) Classification |  |  |  |
| Closed | 1.00 | — |  |
| GA1 | 0.00 |  | >0.9 |
| GA2 | 3.99 | 0.32, 49.0 | 0.3 |
| GA3 | 24.6 | 2.13, 467 | 0.017 |
| ^1^OR = Odds Ratio, CI = Confidence Interval | | | |

**DAG 2 Table: Logistic regression model for probability of in-hospital death (without imputation of creatinine and hemoglobin data)**

| **Model Variables** | **OR**^1^ | **CI 95%**^1^ | **p-value** |
| --- | --- | --- | --- |
| Antihypertensives |  |  |  |
| Not prescribed | 1.00 | — |  |
| Prescribed | 5.18 | 0.79, 39.6 | 0.094 |
| Race |  |  |  |
| Mixed Race | 1.00 | — |  |
| Black | 0.00 |  | >0.9 |
| Age (years) | 1.05 | 1.00, 1.11 | 0.069 |
| Myocardial infarction (AMI) |  |  |  |
| No AMI | 1.00 | — |  |
| AMI | 0.00 |  | >0.9 |
| Liquor consumption |  |  |  |
| No consumption | 1.00 | — |  |
| Consumption | 2.01 | 0.21, 19.0 | 0.5 |
| History of obesity |  |  |  |
| Absent | 1.00 | — |  |
| Present | 3.72 | 0.18, 67.4 | 0.4 |
| Diabetes Mellitus Type 1 or 2 |  |  |  |
| No Diabetes | 1.00 | — |  |
| Diabetes | 1.73 | 0.17, 13.3 | 0.6 |
| Cerebrovascular Disease (CVD) |  |  |  |
| No CVD | 1.00 | — |  |
| CVD | 0.00 |  | >0.9 |
| Pain by VAS | 0.58 | 0.13, 2.64 | 0.5 |
| Intravenous fluids (mL) | 1.00 | 1.00, 1.00 | 0.3 |
| Hemoglobin (gr/dL) | 0.84 | 0.58, 1.19 | 0.3 |
| Creatinine | 1.81 | 0.77, 3.15 | 0.046 |
| Anxiety |  |  |  |
| Absent | 1.00 | — |  |
| Present | 6.10 | 0.90, 47.8 | 0.069 |
| Time to surgery (days0 | 0.90 | 0.72, 1.05 | 0.3 |
| Trauma Score (RTS) | 1.95 | 0.45, 17.6 | 0.5 |
| Weight (Kg) | 0.98 | 0.90, 1.06 | 0.6 |
| Cigarette, tobacco or vaper smoker |  |  |  |
| No | 1.00 | — |  |
| Yes | 2.75 | 0.51, 18.4 | 0.3 |
| Insomnia |  |  |  |
| Absent | 1.00 | — |  |
| Present | 0.62 | 0.06, 5.51 | 0.7 |
| Clinical infection |  |  |  |
| Absent | 1.00 | — |  |
| Present | 1.00 | 0.09, 10.0 | >0.9 |
| Constipation |  |  |  |
| Absent | 1.00 | — |  |
| Present |  |  |  |
| Gustillo-Anderson (GA) Classification |  |  |  |
| Closed | 1.00 | — |  |
| GA1 | 0.00 |  | >0.9 |
| GA2 | 5.38 | 0.59, 56.5 | 0.13 |
| GA3 | 10.0 | 1.03, 136 | 0.059 |
| ^1^OR = Odds Ratio, CI = Confidence Interval | | | |

**APPENDIX 7**

**DAG 3: Outcome of days of hospital stay (4,8,18,36,41,50,51,55,56)**


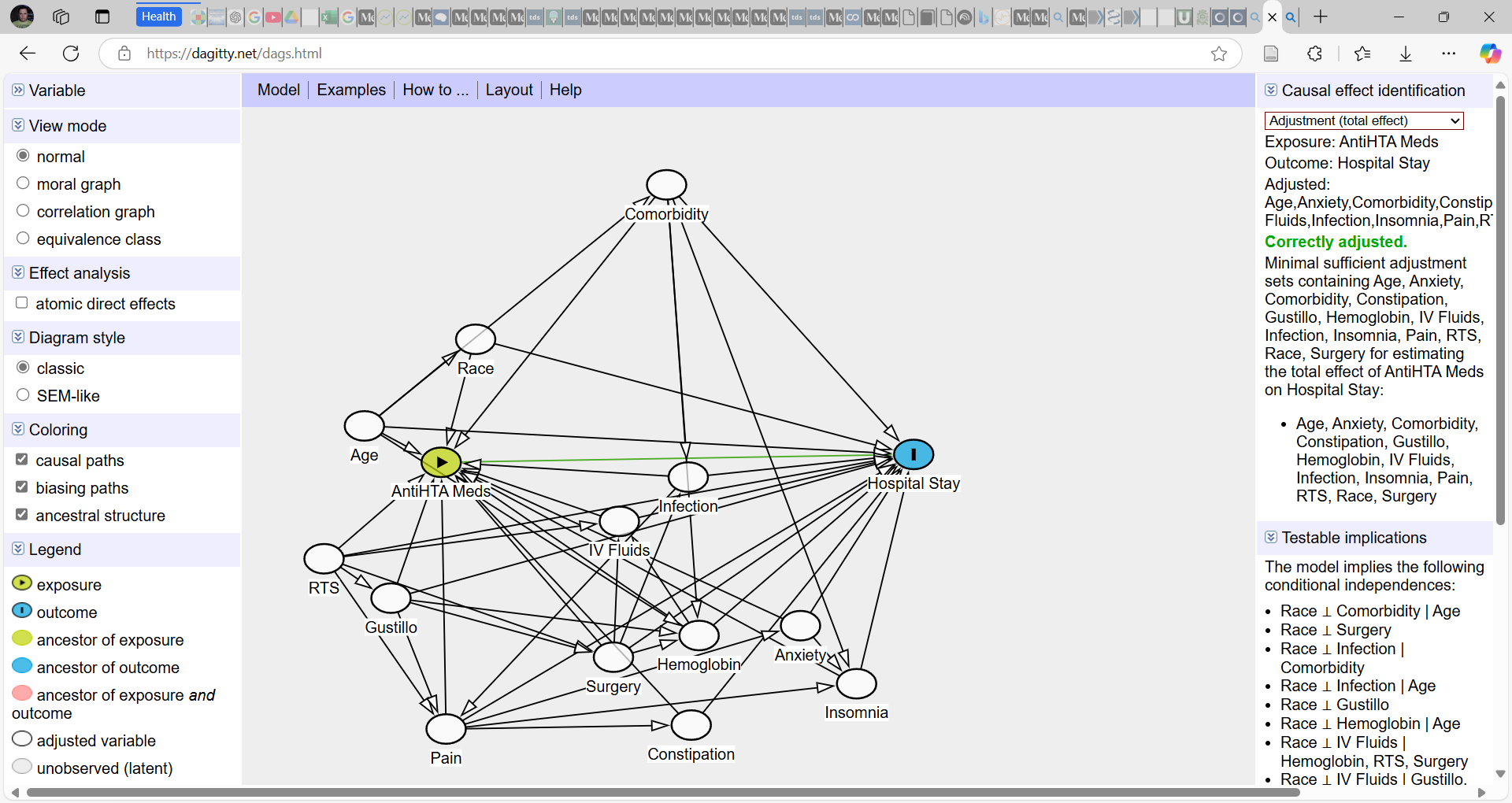


**DAG 3 Table: Multiple linear regression model for the outcome of hospital stay (includes stays longer than 60 days)**

| **Model Variables** | **Beta** | **CI 95%**^1^ | **p-value** |
| --- | --- | --- | --- |
| Antihypertensives |  |  |  |
| Not prescribed | — | — |  |
| Prescribed | 3.4 | 1.4, 5.5 | 0.001 |
| Time to surgery (days) | 0.27 | 0.16, 0.37 | <0.001 |
| Age (years) | -0.01 | -0.04, 0.02 | 0.6 |
| Race |  |  |  |
| Mixed Race | — | — |  |
| Black | -3.1 | -8.3, 2.1 | 0.2 |
| Weight (Kg) | -0.09 | -0.16, -0.01 | 0.028 |
| Cigarette, tobacco or vaper smoker |  |  |  |
| No | — | — |  |
| Yes | -0.65 | -2.0, 0.72 | 0.4 |
| Liquor consumption |  |  |  |
| No consumption | — | — |  |
| Consumption | 0.18 | -1.2, 1.6 | 0.8 |
| History of obesity |  |  |  |
| Absent | — | — |  |
| Present | 3.2 | 0.14, 6.4 | 0.041 |
| Diabetes Mellitus Type 1 or 2 |  |  |  |
| No Diabetes | — | — |  |
| Diabetes | 3.0 | 0.20, 5.8 | 0.036 |
| Myocardial infarction (AMI) |  |  |  |
| No AMI | — | — |  |
| AMI | -1.1 | -9.6, 7.3 | 0.8 |
| Cerebrovascular Disease (CVD) |  |  |  |
| No CVD | — | — |  |
| CVD | 2.5 | -7.9, 13 | 0.6 |
| Pain by VAS | -0.22 | -1.3, 0.89 | 0.7 |
| Insomnia |  |  |  |
| Absent | — | — |  |
| Present | 0.17 | -1.3, 1.7 | 0.8 |
| Constipation |  |  |  |
| Absent | — | — |  |
| Present | -0.30 | -1.9, 1.3 | 0.7 |
| Anxiety |  |  |  |
| Absent | — | — |  |
| Present | 1.2 | -0.67, 3.0 | 0.2 |
| Intravenous fluids (mL) | 0.00 | 0.00, 0.00 | <0.001 |
| Trauma Score (RTS) | -0.01 | -0.06, 0.04 | 0.6 |
| Clinical infection |  |  |  |
| Absent | — | — |  |
| Present | 3.6 | 1.8, 5.4 | <0.001 |
| Hemoglobin (gr/dL) | -0.35 | -0.63, -0.07 | 0.013 |
| ^1^CI = Confidence Interval | | | |

**DAG 3 Table: Multiple linear regression model for the outcome of hospital stay (includes only stays less than 60 days)**

| **Model Variables** | **Beta** | **CI 95%**^1^ | **p-value** |
| --- | --- | --- | --- |
| Antihypertensives |  |  |  |
| Not prescribed | — | — |  |
| Prescribed | 1.5 | -0.10, 3.1 | 0.066 |
| Time to surgery (days) | 0.04 | 0.01, 0.07 | 0.016 |
| Age (years) | 0.01 | -0.01, 0.04 | 0.3 |
| Race |  |  |  |
| Mixed Race | — | — |  |
| Black | -1.1 | -5.1, 2.9 | 0.6 |
| Weight (Kg) | -0.02 | -0.08, 0.03 | 0.4 |
| Cigarette, tobacco or vaper smoker |  |  |  |
| No | — | — |  |
| Yes | -0.54 | -1.6, 0.54 | 0.3 |
| Liquor consumption |  |  |  |
| No consumption | — | — |  |
| Consumption | 0.12 | -1.0, 1.2 | 0.8 |
| History of obesity |  |  |  |
| Absent | — | — |  |
| Present | -0.05 | -2.3, 2.2 | >0.9 |
| Diabetes Mellitus Type 1 or 2 |  |  |  |
| No Diabetes | — | — |  |
| Diabetes | 1.3 | -0.94, 3.6 | 0.3 |
| Myocardial infarction (AMI) |  |  |  |
| No AMI | — | — |  |
| AMI | -0.77 | -7.3, 5.7 | 0.8 |
| Cerebrovascular Disease (CVD) |  |  |  |
| No CVD | — | — |  |
| CVD | 3.4 | -4.6, 11 | 0.4 |
| Pain by VAS | -0.12 | -0.97, 0.73 | 0.8 |
| Insomnia |  |  |  |
| Absent | — | — |  |
| Present | -0.23 | -1.4, 0.92 | 0.7 |
| Constipation |  |  |  |
| Absent | — | — |  |
| Present | -0.55 | -1.8, 0.70 | 0.4 |
| Anxiety |  |  |  |
| Absent | — | — |  |
| Present | 1.1 | -0.26, 2.5 | 0.11 |
| Intravenous fluids (mL) | 0.00 | 0.00, 0.00 | <0.001 |
| Trauma Score (RTS) | -0.02 | -0.06, 0.02 | 0.3 |
| Clinical infection |  |  |  |
| Absent | — | — |  |
| Present | 2.0 | 0.68, 3.4 | 0.003 |
| Hemoglobin (gr/dL) | -0.22 | -0.43, -0.01 | 0.044 |
| ^1^CI = Confidence Interval | | | |

**Cox regression, with in-hospital mortality as a competing risk, for the outcome of risk of being discharged alive from the hospital (includes only stays longer than 60 days)**

| **Model Variables** | **sHR**^1^ | **CI 95%**^1^ | **p-value** |
| --- | --- | --- | --- |
| Antihypertensives |  |  |  |
| Not prescribed | 1.00 | — |  |
| Prescribed | 0.72 | 0.55, 0.96 | 0.025 |
| Age (years) | 1.00 | 1.00, 1.01 | 0.4 |
| Sex |  |  |  |
| Female | 1.00 | — |  |
| Male | 1.55 | 1.25, 1.94 | <0.001 |
| Race |  |  |  |
| Mixed Race | 1.00 | — |  |
| Black | 1.94 | 0.96, 3.94 | 0.067 |
| Trauma Score (RTS) | 1.00 | 0.99, 1.00 | 0.5 |
| Time to surgery (days) | 0.95 | 0.93, 0.96 | <0.001 |
| Hemoglobin (gr/dL) | 1.06 | 1.02, 1.10 | 0.005 |
| Clinical infection |  |  |  |
| Absent | 1.00 | — |  |
| Present | 0.48 | 0.38, 0.61 | <0.001 |
| Non-Steroidal Anti-Inflammatory Drugs (NSAIDs) |  |  |  |
| Not prescribed | 1.00 | — |  |
| Prescribed | 0.85 | 0.31, 2.28 | 0.7 |
| Opioid |  |  |  |
| Not prescribed | 1.00 | — |  |
| Prescribed | 1.37 | 0.56, 3.35 | 0.5 |
| Venous thromboembolic disease (VTD) |  |  |  |
| Not prescribed | 1.00 | — |  |
| Prescribed | 0.64 | 0.28, 1.43 | 0.3 |
| Intravenous fluids (mL) | 1.00 | 1.00, 1.00 | <0.001 |
| Cigarette, tobacco or vaper smoker |  |  |  |
| No | 1.00 | — |  |
| Yes | 1.42 | 1.17, 1.71 | <0.001 |
| Liquor consumption |  |  |  |
| No consumption | 1.00 | — |  |
| Consumption | 0.83 | 0.69, 1.01 | 0.060 |
| History of obesity |  |  |  |
| Absent | 1.00 | — |  |
| Present | 1.09 | 0.76, 1.57 | 0.6 |
| Diabetes Mellitus Type 1 or 2 |  |  |  |
| No Diabetes | 1.00 | — |  |
| Diabetes | 0.43 | 0.29, 0.65 | <0.001 |
| Myocardial infarction (AMI) |  |  |  |
| No AMI | 1.00 | — |  |
| AMI | 2.51 | 0.79, 7.93 | 0.12 |
| Cerebrovascular Disease (CVD) |  |  |  |
| No CVD | 1.00 | — |  |
| CVD | 1.42 | 0.35, 5.82 | 0.6 |
| Pain by VAS | 0.88 | 0.76, 1.02 | 0.10 |
| ^1^ scHR = specific cause Hazard Ratio , CI = Confidence Interval | | | |

**Cox regression, with in-hospital mortality as a competing risk, for the outcome of risk of being discharged alive from the hospital (includes only stays less than 60 days)**

| **Model Variables** | **sHR**^1^ | **CI 95%**^1^ | **p-value** |
| --- | --- | --- | --- |
| Antihypertensives |  |  |  |
| Not prescribed | 1.00 | — |  |
| Prescribed | 0.79 | 0.59, 1.06 | 0.12 |
| Age (years) | 1.00 | 1.00, 1.00 | 0.9 |
| Sex |  |  |  |
| Female | 1.00 | — |  |
| Male | 1.31 | 1.07, 1.62 | 0.010 |
| Race |  |  |  |
| Mixed Race | 1.00 | — |  |
| Black | 1.88 | 0.92, 3.81 | 0.082 |
| Trauma Score (RTS) | 1.00 | 1.00, 1.01 | 0.5 |
| Time to surgery (days) | 0.96 | 0.94, 0.97 | <0.001 |
| Hemoglobin (gr/dL) | 1.03 | 0.99, 1.07 | 0.10 |
| Clinical infection |  |  |  |
| Absent | 1.00 | — |  |
| Present | 0.57 | 0.44, 0.72 | <0.001 |
| Non-Steroidal Anti-Inflammatory Drugs (NSAIDs) |  |  |  |
| Not prescribed | 1.00 | — |  |
| Prescribed | 0.28 | 0.07, 1.14 | 0.076 |
| Opioid |  |  |  |
| Not prescribed | 1.00 | — |  |
| Prescribed | 1.29 | 0.57, 2.93 | 0.5 |
| Venous thromboembolic disease (VTD) |  |  |  |
| Not prescribed | 1.00 | — |  |
| Prescribed | 0.50 | 0.19, 1.26 | 0.14 |
| Intravenous fluids (mL) | 1.00 | 1.00, 1.00 | <0.001 |
| Cigarette, tobacco or vaper smoker |  |  |  |
| No | 1.00 | — |  |
| Yes | 1.31 | 1.10, 1.55 | 0.002 |
| Liquor consumption |  |  |  |
| No consumption | 1.00 | — |  |
| Consumption | 1.15 | 0.93, 1.41 | 0.2 |
| History of obesity |  |  |  |
| Absent | 1.00 | — |  |
| Present | 0.98 | 0.70, 1.37 | >0.9 |
| Diabetes Mellitus Type 1 or 2 |  |  |  |
| No Diabetes | 1.00 | — |  |
| Diabetes | 0.31 | 0.20, 0.49 | <0.001 |
| Myocardial infarction (AMI) |  |  |  |
| No AMI | 1.00 | — |  |
| AMI | 2.76 | 0.87, 8.75 | 0.084 |
| Cerebrovascular Disease (CVD) |  |  |  |
| No CVD | 1.00 | — |  |
| CVD | 1.42 | 0.34, 5.85 | 0.6 |
| Pain by VAS | 0.83 | 0.71, 0.97 | 0.018 |
| ^1^ scHR = specific cause Hazard Ratio, CI = Confidence Interval | | | |

**APPENDIX 8 - Sensitivity analysis**

**Characteristics of eligible subjects who were lost to follow-up (it is not known whether they had hypotension and in-hospital death outcomes)**

| **Basal Characteristics** | **Total**,  n = 13^1^ | **Not prescribed**,  n = 13^1^ | **Prescribed**,  n = 0^1^ |
| --- | --- | --- | --- |
| Male sex | 10 (77%) | 10 (77%) | 0 (NA%) |
| Unknown | 0 | 0 | 0 |
| Mixed race | 13 (100%) | 13 (100%) | 0 (NA%) |
| Lost data | 0 | 0 | 0 |
| Age (years) | 28 (24, 36) | 28 (24, 36) | NA (NA, NA) |
| Lost data | 0 | 0 | 0 |
| Respiratory rate (rpm) on admission |  |  |  |
| 18 | 1 (7.7%) | 1 (7.7%) | 0 (NA%) |
| 19 | 1 (7.7%) | 1 (7.7%) | 0 (NA%) |
| 19 | 1 (7.7%) | 1 (7.7%) | 0 (NA%) |
| 20 | 1 (7.7%) | 1 (7.7%) | 0 (NA%) |
| 22 | 1 (7.7%) | 1 (7.7%) | 0 (NA%) |
| 22 | 2 (15%) | 2 (15%) | 0 (NA%) |
| 22 | 2 (15%) | 2 (15%) | 0 (NA%) |
| 23 | 3 (23%) | 3 (23%) | 0 (NA%) |
| 26 | 1 (7.7%) | 1 (7.7%) | 0 (NA%) |
| Lost data | 0 | 0 | 0 |
| Glasgow Coma Scale (GCS) (pts.) |  |  |  |
| 15 | 13 (100%) | 13 (100%) | 0 (NA%) |
| Lost data | 0 | 0 | 0 |
| Systolic Blood Pressure (SBP) on admission (mmHg) | 119.0 (118.0, 122.0) | 119.0 (118.0, 122.0) | NA (NA, NA) |
| Lost data | 0 | 0 | 0 |
| Trauma Score (RTS) |  |  |  |
| 3.68 | 13 (100%) | 13 (100%) | 0 (NA%) |
| Lost data | 0 | 0 | 0 |
| Broken bone |  |  |  |
| Clavicle | 0 (0%) | 0 (0%) | 0 (NA%) |
| Shoulder | 0 (0%) | 0 (0%) | 0 (NA%) |
| Humerus | 0 (0%) | 0 (0%) | 0 (NA%) |
| Elbow | 0 (0%) | 0 (0%) | 0 (NA%) |
| Radium or ulna | 5 (38%) | 5 (38%) | 0 (NA%) |
| Wrist | 0 (0%) | 0 (0%) | 0 (NA%) |
| Hane | 0 (0%) | 0 (0%) | 0 (NA%) |
| Pelvis | 0 (0%) | 0 (0%) | 0 (NA%) |
| Hip | 1 (7.7%) | 1 (7.7%) | 0 (NA%) |
| Femur | 2 (15%) | 2 (15%) | 0 (NA%) |
| Knee | 0 (0%) | 0 (0%) | 0 (NA%) |
| Tibia – Fibula | 3 (23%) | 3 (23%) | 0 (NA%) |
| Ankle | 1 (7.7%) | 1 (7.7%) | 0 (NA%) |
| Foot | 1 (7.7%) | 1 (7.7%) | 0 (NA%) |
| Lost data | 0 | 0 | 0 |
| Gustillo Anderson (GA) Classification |  |  |  |
| Closed | 10 (77%) | 10 (77%) | 0 (NA%) |
| GA1 | 2 (15%) | 2 (15%) | 0 (NA%) |
| GA2 | 1 (7.7%) | 1 (7.7%) | 0 (NA%) |
| GA3 | 0 (0%) | 0 (0%) | 0 (NA%) |
| Lost data | 0 | 0 | 0 |
| Weight (Kg) |  |  |  |
| 57.0 | 1 (7.7%) | 1 (7.7%) | 0 (NA%) |
| 60.0 | 1 (7.7%) | 1 (7.7%) | 0 (NA%) |
| 61.0 | 1 (7.7%) | 1 (7.7%) | 0 (NA%) |
| 67.0 | 1 (7.7%) | 1 (7.7%) | 0 (NA%) |
| 69.0 | 1 (7.7%) | 1 (7.7%) | 0 (NA%) |
| 69.1 | 7 (54%) | 7 (54%) | 0 (NA%) |
| 78.0 | 1 (7.7%) | 1 (7.7%) | 0 (NA%) |
| Lost data | 0 | 0 | 0 |
| Cigarette, tobacco or vaper smoker | 6 (46%) | 6 (46%) | 0 (NA%) |
| Lost data | 0 | 0 | 0 |
| Liquor consumption | 0 (0%) | 0 (0%) | 0 (NA%) |
| Lost data | 0 | 0 | 0 |
| History of obesity | 0 (0%) | 0 (0%) | 0 (NA%) |
| Lost data | 0 | 0 | 0 |
| Diabetes Mellitus, Type 1 or 2 | 0 (0%) | 0 (0%) | 0 (NA%) |
| Lost data | 0 | 0 | 0 |
| Myocardial infarction (AMI) | 0 (0%) | 0 (0%) | 0 (NA%) |
| Lost data | 0 | 0 | 0 |
| Cerebrovascular Disease (CVD) | 0 (0%) | 0 (0%) | 0 (NA%) |
| Lost data | 0 | 0 | 0 |
| ^1^n (%); Median (Interquartile Range, IQR) | | | |

| **Characteristics during**  **Hospitalization** | **Total**,  n = 13^1^ | **Not prescribed**,  n = 13^1^ | **Prescribed**,  n = 0^1^ |
| --- | --- | --- | --- |
| Systolic blood pressure (SBP) (mmHg) |  |  |  |
| 142 | 1 (7.7%) | 1 (7.7%) | 0 (NA%) |
| 146 | 1 (7.7%) | 1 (7.7%) | 0 (NA%) |
| 148 | 4 (31%) | 4 (31%) | 0 (NA%) |
| 149 | 1 (7.7%) | 1 (7.7%) | 0 (NA%) |
| 150 | 2 (15%) | 2 (15%) | 0 (NA%) |
| 152 | 2 (15%) | 2 (15%) | 0 (NA%) |
| 158 | 1 (7.7%) | 1 (7.7%) | 0 (NA%) |
| 159 | 1 (7.7%) | 1 (7.7%) | 0 (NA%) |
| Lost data | 0 | 0 | 0 |
| Diastolic blood pressure (DBP) (mmHg) |  |  |  |
| 89 | 5 (38%) | 5 (38%) | 0 (NA%) |
| 90 | 1 (7.7%) | 1 (7.7%) | 0 (NA%) |
| 93 | 2 (15%) | 2 (15%) | 0 (NA%) |
| 97 | 1 (7.7%) | 1 (7.7%) | 0 (NA%) |
| 98 | 1 (7.7%) | 1 (7.7%) | 0 (NA%) |
| 98 | 2 (15%) | 2 (15%) | 0 (NA%) |
| 98 | 1 (7.7%) | 1 (7.7%) | 0 (NA%) |
| Lost data | 0 | 0 | 0 |
| Antihypertensive medication |  |  |  |
| No medication | 13 (100%) | 13 (100%) | 0 (NA%) |
| Losartan | 0 (0%) | 0 (0%) | 0 (NA%) |
| Enalapril | 0 (0%) | 0 (0%) | 0 (NA%) |
| Amlodipine | 0 (0%) | 0 (0%) | 0 (NA%) |
| Metoprolol | 0 (0%) | 0 (0%) | 0 (NA%) |
| Hydrochlorothiazide | 0 (0%) | 0 (0%) | 0 (NA%) |
| Prazosin | 0 (0%) | 0 (0%) | 0 (NA%) |
| Clonidine | 0 (0%) | 0 (0%) | 0 (NA%) |
| Unknown | 0 | 0 | 0 |
| Administration schedule |  |  |  |
| No medication | 13 (100%) | 13 (100%) | 0 (NA%) |
| Every 24 hrs | 0 (0%) | 0 (0%) | 0 (NA%) |
| Every 12 hrs | 0 (0%) | 0 (0%) | 0 (NA%) |
| Every 8 hrs | 0 (0%) | 0 (0%) | 0 (NA%) |
| Every 6 hrs | 0 (0%) | 0 (0%) | 0 (NA%) |
| Lost data | 0 | 0 | 0 |
| Days of prescribed medication | 0 (0%) | 0 (0%) | 0 (NA%) |
| Lost data | 0 | 0 | 0 |
| Time to surgery (days) | NA (NA, NA) | NA (NA, NA) | NA (NA, NA) |
| Lost data | 13 | 13 | 0 |
| Intravenous fluids during  hospitalization (mL) | 1,100 (900, 2,000) | 1,100 (900, 2,000) | NA (NA, NA) |
| Lost data | 0 | 0 | 0 |
| Anti-inflammatories | 13 (100%) | 13 (100%) | 0 (NA%) |
| Lost data | 0 | 0 | 0 |
| Opioid | 13 (100%) | 13 (100%) | 0 (NA%) |
| Lost data | 0 | 0 | 0 |
| Clinical infection | 0 (0%) | 0 (0%) | 0 (NA%) |
| Lost data | 0 | 0 | 0 |
| Thromboembolic disease | 0 (0%) | 0 (0%) | 0 (NA%) |
| Lost data | 0 | 0 | 0 |
| Pain by VAS |  |  |  |
| 1 | 5 (38%) | 5 (38%) | 0 (NA%) |
| 2 | 7 (54%) | 7 (54%) | 0 (NA%) |
| 3 | 1 (7.7%) | 1 (7.7%) | 0 (NA%) |
| Lost data | 0 | 0 | 0 |
| Insomnia | 1 (7.7%) | 1 (7.7%) | 0 (NA%) |
| Lost data | 0 | 0 | 0 |
| Constipation | 0 (0%) | 0 (0%) | 0 (NA%) |
| Lost data | 0 | 0 | 0 |
| Anxiety | 0 (0%) | 0 (0%) | 0 (NA%) |
| Lost data | 0 | 0 | 0 |
| Hemoglobin (gr/dL) |  |  |  |
| 12.8 | 1 (50%) | 1 (50%) | 0 (NA%) |
| 15.0 | 1 (50%) | 1 (50%) | 0 (NA%) |
| Lost data | 11 | 11 | 0 |
| Creatinine (mg/dL) | NA (NA, NA) | NA (NA, NA) | NA (NA, NA) |
| Lost data | 13 | 13 | 0 |
| Losses |  |  |  |
| 0 | 0 (0%) | 0 (0%) | 0 (NA%) |
| 1 | 13 (100%) | 13 (100%) | 0 (NA%) |
| Lost data | 0 | 0 | 0 |
| Hospital stay <60 | 13 (100%) | 13 (100%) | 0 (NA%) |
| Lost data | 0 | 0 | 0 |
| Hospital stay |  |  |  |
| 2 | 4 (31%) | 4 (31%) | 0 (NA%) |
| 3 | 5 (38%) | 5 (38%) | 0 (NA%) |
| 4 | 2 (15%) | 2 (15%) | 0 (NA%) |
| 5 | 1 (7.7%) | 1 (7.7%) | 0 (NA%) |
| 6 | 1 (7.7%) | 1 (7.7%) | 0 (NA%) |
| Lost data | 0 | 0 | 0 |
| Prescribed daily dose (PDD) | NA (NA, NA) | NA (NA, NA) | NA (NA, NA) |
| Lost data | 13 | 13 | 0 |
| ^1^n (%); Median (IQR) | | | |

**Logistic regression model of probability of hypotension (worst-case scenario sensitivity analysis: subjects lost to follow-up have the outcome)**

| **Model Variables** | **OR**^1^ | **CI 95%**^1^ | **p-value** |
| --- | --- | --- | --- |
| Antihypertensives |  |  |  |
| Not prescribed | 1.00 | — |  |
| Prescribed | 10.7 | 5.15, 23.6 | <0.001 |
| Race |  |  |  |
| Mixed Race | 1.00 | — |  |
| Black | 0.24 | 0.01, 1.74 | 0.2 |
| Age (years) | 1.01 | 1.00, 1.02 | 0.10 |
| Myocardial infarction (AMI)) |  |  |  |
| No AMI | 1.00 | — |  |
| AMI | 1.26 | 0.03, 31.1 | 0.9 |
| Systolic blood pressure (SBP) | 1.01 | 0.99, 1.03 | 0.2 |
| Liquor Consumption |  |  |  |
| No consumption | 1.00 | — |  |
| Consumption | 0.65 | 0.36, 1.15 | 0.15 |
| History of obesity |  |  |  |
| Absent | 1.00 | — |  |
| Present | 2.06 | 0.72, 5.84 | 0.2 |
| Diabetes Mellitus Type 1 or 2 |  |  |  |
| No Diabetes | 1.00 | — |  |
| Diabetes | 1.69 | 0.60, 4.54 | 0.3 |
| Cerebrovascular Disease (CVD) |  |  |  |
| No CVD | 1.00 | — |  |
| CVD | 1.09 | 0.03, 37.2 | >0.9 |
| Pain by VAS | 1.07 | 0.70, 1.66 | 0.7 |
| Intravenous fluids (mL) | 1.00 | 1.00, 1.00 | 0.057 |
| Hemoglobin (gr/dL) | 0.83 | 0.75, 0.92 | <0.001 |
| Creatinine | 1.25 | 0.86, 1.79 | 0.2 |
| Anxiety |  |  |  |
| Absent | 1.00 | — |  |
| Present | 1.09 | 0.59, 1.99 | 0.8 |
| Time to surgery (days) | 0.99 | 0.95, 1.00 | 0.4 |
| Trauma Score (RTS) | 0.98 | 0.97, 1.00 | 0.069 |
| Weight (Kg) | 0.97 | 0.94, 1.00 | 0.023 |
| Cigarette, tobacco or vaper smoker |  |  |  |
| No | 1.00 | — |  |
| Yes | 0.99 | 0.62, 1.57 | >0.9 |
| Insomnia |  |  |  |
| Absent | 1.00 | — |  |
| Present | 1.13 | 0.65, 1.95 | 0.7 |
| Clinical infection |  |  |  |
| Absent | 1.00 | — |  |
| Present | 1.79 | 0.99, 3.24 | 0.054 |
| Constipation |  |  |  |
| Absent | 1.00 | — |  |
| Present |  |  |  |
| Gustillo-Anderson (GA) Classification |  |  |  |
| Closed | 1.00 | — |  |
| GA1 | 2.03 | 1.05, 3.92 | 0.035 |
| GA2 | 1.21 | 0.62, 2.34 | 0.6 |
| GA3 | 1.48 | 0.77, 2.85 | 0.2 |
| ^1^OR = Odds Ratio, CI = Confidence Interval | | | |

**Logistic regression model of probability of death (Worst-case scenario sensitivity analysis: subjects with loss to follow-up have the outcome)**

| **Characteristic** | **OR**^1^ | **CI 95%**^1^ | **p-value** |
| --- | --- | --- | --- |
| Antihypertensives |  |  |  |
| Not prescribed | 1.00 | — |  |
| Prescribed | 4.69 | 0.74, 35.1 | 0.11 |
| Race |  |  |  |
| Mixed Race | 1.00 | — |  |
| Black | 0.00 |  | >0.9 |
| Age (years) | 1.05 | 1.00, 1.10 | 0.082 |
| Myocardial infarction (AMI)) |  |  |  |
| No AMI | 1.00 | — |  |
| AMI | 0.00 |  | >0.9 |
| Systolic blood pressure (SBP) | 1.01 | 0.96, 1.06 | 0.7 |
| Liquor Consumption |  |  |  |
| No consumption | 1.00 | — |  |
| Consumption | 2.43 | 0.28, 21.6 | 0.4 |
| History of obesity |  |  |  |
| Absent | 1.00 | — |  |
| Present | 4.00 | 0.19, 74.5 | 0.4 |
| Diabetes Mellitus Type 1 or 2 |  |  |  |
| No Diabetes | 1.00 | — |  |
| Diabetes | 1.34 | 0.14, 10.3 | 0.8 |
| Cerebrovascular Disease (CVD) |  |  |  |
| No CVD | 1.00 | — |  |
| CVD | 0.00 |  | >0.9 |
| Pain by VAS | 0.63 | 0.14, 2.86 | 0.5 |
| Intravenous fluids (mL) | 1.00 | 1.00, 1.00 | 0.2 |
| Hemoglobin (gr/dL) | 0.88 | 0.62, 1.23 | 0.5 |
| Creatinine | 1.83 | 0.81, 3.17 | 0.038 |
| Anxiety |  |  |  |
| Absent | 1.00 | — |  |
| Present | 6.26 | 1.00, 45.5 | 0.054 |
| Time to surgery (days) | 0.91 | 0.73, 1.05 | 0.3 |
| Trauma Score (RTS) | 1.87 | 0.45, 15.9 | 0.5 |
| Weight (Kg) | 0.97 | 0.89, 1.06 | 0.5 |
| Cigarette, tobacco or vaper smoker |  |  |  |
| No | 1.00 | — |  |
| Yes | 2.66 | 0.48, 18.2 | 0.3 |
| Insomnia |  |  |  |
| Absent | 1.00 | — |  |
| Present | 0.56 | 0.05, 4.89 | 0.6 |
| Clinical infection |  |  |  |
| Absent | 1.00 | — |  |
| Present | 1.46 | 0.16, 12.9 | 0.7 |
|  |  |  |  |
| Constipation |  |  |  |
| Absent | 1.00 | — |  |
| Present |  |  |  |
| Gustillo-Anderson (GA) Classification |  |  |  |
| Closed | 1.00 | — |  |
| GA1 | 0.00 |  | >0.9 |
| GA2 | 4.39 | 0.49, 43.3 | 0.2 |
| GA3 | 10.8 | 1.16, 143 | 0.049 |
| ^1^OR = Odds Ratio, CI = Confidence Interval | | | |

**Competing risks model for time to discharge (Worst-case scenario sensitivity analysis: subjects lost to follow-up have the outcome)**

| **Characteristic** | **sHR**^1^ | **CI 95%**^1^ | **p-value** |
| --- | --- | --- | --- |
| Antihypertensives |  |  |  |
| Not prescribed | 1.00 | — |  |
| Prescribed | 0.74 | 0.55, 0.99 | 0.043 |
| Age (years) | 1.00 | 1.00, 1.00 | >0.9 |
| Sex |  |  |  |
| Female | 1.00 | — |  |
| Male | 1.32 | 1.07, 1.62 | 0.009 |
| Antihypertensives |  |  |  |
| Not prescribed | 1.00 | — |  |
| Prescribed | 1.86 | 0.92, 3.78 | 0.085 |
| Trauma Score (RTS) | 1.00 | 1.00, 1.01 | 0.5 |
| Time to surgery (days) | 0.98 | 0.96, 0.99 | <0.001 |
| Hemoglobin (gr/dL) | 1.03 | 0.99, 1.07 | 0.13 |
| Clinical infection |  |  |  |
| Absent | 1.00 | — |  |
| Present | 0.56 | 0.44, 0.72 | <0.001 |
| Non-Steroidal Anti-Inflammatory Drugs (NSAIDs) |  |  |  |
| Not prescribed | 1.00 | — |  |
| Prescribed | 0.27 | 0.07, 1.10 | 0.068 |
| Opioid |  |  |  |
| Not prescribed | 1.00 | — |  |
| Prescribed | 1.28 | 0.57, 2.90 | 0.5 |
| Venous thromboembolic disease (VTD) |  |  |  |
| Not prescribed | 1.00 | — |  |
| Prescribed | 0.49 | 0.20, 1.24 | 0.13 |
| Intravenous fluids (mL) | 1.00 | 1.00, 1.00 | <0.001 |
| Cigarette, tobacco or vaper smoker |  |  |  |
| No | 1.00 | — |  |
| Yes | 1.35 | 1.14, 1.60 | <0.001 |
| Liquor Consumption |  |  |  |
| No consumption | 1.00 | — |  |
| Consumption | 1.10 | 0.90, 1.35 | 0.3 |
| History of obesity |  |  |  |
| Absent | 1.00 | — |  |
| Present | 0.95 | 0.68, 1.31 | 0.7 |
| Diabetes Mellitus Type 1 or 2 |  |  |  |
| No Diabetes | 1.00 | — |  |
| Diabetes | 0.34 | 0.22, 0.52 | <0.001 |
| Myocardial infarction (AMI) |  |  |  |
| No AMI | 1.00 | — |  |
| AMI | 2.92 | 0.92, 9.25 | 0.068 |
|  |  |  |  |
| Cerebrovascular Disease (CVD) |  |  |  |
| No CVD | 1.00 | — |  |
| CVD | 1.51 | 0.37, 6.23 | 0.6 |
| Pain by VAS | 0.82 | 0.70, 0.95 | 0.009 |
| ^1^ sHR = Subdistribution Hazard Ratio, CI = Confidence Interval | | | |

**References:**

38. A. Amirhekmat , A. Dinicu , L. Grimaud , et al., “ Diagnosis and Treatment of Chronic Medical Conditions among Trauma Patients at a Level 1

Trauma Center ,” American Surgeon 86 , no. 10 ( 2020 ): 1264 – 1268 , https://doi.org/10.1177/0003134820964212 .

39. S. S. A. Soliman , E. H. Guseman , Z. T. Haile , and G. Ice , “ Prevalence and Determinants of Hypertension Unawareness Among Egyptian

Adults: The 2015 EHIS ,” Journal of Human Hypertension 35 , no. 10 ( 2021 ): 927 – 934 , https://doi.org/10.1038/s41371-020-00431-1 .

40. L. Saliba , S. P. Stawicki , C. Thongrong , S. D. Bergese , T. J. Papadimos , and A. T. Gerlach , “ Association Between In-Hospital Acute

Hypertensive Episodes and Outcomes in Older Trauma Patients ,” Internal and Emergency Medicine 9 , no. 5 ( 2014 ): 553 – 558 ,

https://doi.org/10.1007/s11739-013-0984-0 .

41. K. Hu , Q. Zhou , Y. Jiang , et al., “ Association Between Frailty and Mortality, Falls, and Hospitalization Among Patients With Hypertension: A

Systematic Review and Meta-Analysis ,” BioMed Research International 2021 ( 2021 ): 2690296 , https://doi.org/10.1155/2021/2690296 .

42. W. H. Lai , S. C. Wu , C. S. Rau , et al., “ Systolic Blood Pressure Lower Than Heart Rate Upon Arrival at and Departure From the Emergency

Department Indicates a Poor Outcome for Adult Trauma Patients ,” International Journal of Environmental Research and Public Health 13 , no. 6

( 2016 ): 528 , https://doi.org/10.3390/ijerph13060528 .

43. R. D. Brook , A. B. Weder , and S. Rajagopalan , “ Environmental Hypertensionology: The Effects of Environmental Factors on Blood Pressure in

Clinical Practice and Research ,” Journal of Clinical Hypertension 13 , no. 11 ( 2022 ): 836 – 842 ,

https://onlinelibrary.wiley.com/doi/10.1111/j.1751-7176.2011.00543.x .

44. K. Kario , S. Hoshide , Y. C. Chia , et al., “ Guidance on Ambulatory Blood Pressure Monitoring: A Statement From the HOPE Asia Network ,”

Journal of Clinical Hypertension 23 , no. 3 ( 2021 ): 411 – 421 , https://doi.org/10.1111/jch.14128 .

45. P. Muntner , D. Shimbo , R. M. Carey , et al., “ Measurement of Blood Pressure in Humans: A Scientific Statement From the American Heart Association,” Hypertension 73 , no. 5 ( 2019 ): e35 – 66 , https://doi.org/10.1161/HYP.0000000000000087 .

**AQ**4**8**6. P. Castiglionia and G. Paratib , “ Present Trends and Future Directions in the Analysis of Cardiovascular Variability ,” Journal of Hypertension 29 (

2011 ): 1285 – 1288 , https://doi.org/10.1097/HJH.0b013e3283491d97 .

47. G. Mancia , “ Short- and Long-Term Blood Pressure Variability Present and Future ,” Hypertension 60 ( 2012 ): 512 – 517 ,

https://doi.org/10.1161/HYPERTENSIONAHA.112.194340 .

48. A. P. Carson , G. Howard , G. L. Burke , S. Shea , E. B. Levitan , and P. Muntner , “ Ethnic Differences in Hypertension Incidence Among

Middle-Aged and Older U. S. Adults: The Multi-Ethnic Study of ,” Atherosclerosis Hypertension 57 , no. 6 ( 2011 ): 1101 – 1107 ,

https://doi.org/10.1161/HYPERTENSIONAHA.110.168005 .

49. G. Hambright , V. Agrawal , P. L. Sladek , S. M. Slonim , and M. S. Truitt , “ Acute Care Surgery: Trauma, Critical Care, Emergency General

Surgery … and Preventative Health? ,” American Journal of Surgery 212 , no. 5 ( 2016 ): 803 – 806 ,

https://doi.org/10.1016/j.amjsurg.2016.07.006 .

50. {{Carlos José Atencia: Reference (50) is empty. This is <span style="color: rgb(27, 27, 27); font-family: &quot;Roboto Mono Web&quot;,

&quot;Bitstream Vera Sans Mono&quot;, Consolas, Courier, monospace; font-size: 16px; letter-spacing: normal;">Muntner P, Hardy ST, Fine LJ,

Jaeger BC, Wozniak G, Levitan EB, Colantonio LD. Trends in Blood Pressure Control Among US Adults With Hypertension, 1999-2000 to 2017-

2018. JAMA. 2020 Sep 22;324(12):1190-1200. doi: 10.1001/jama.2020.14545. PMID: 32902588; PMCID: PMC7489367.</span>}}PubMed

Central Link [Internet] ,Muntner P, Hardy ST, Fine LJ, Jaeger BC, Wozniak G, Levitan EB, Colantonio LD. Trends in Blood Pressure Control Among

US Adults With Hypertension, 1999-2000 to 2017-2018. JAMA. 2020 Sep 22;324(12):1190-1200. doi: 10.1001/jama.2020.14545. PMID:

32902588; PMCID: PMC7489367. accessed September 11, 2022, https://www.ncbi.nlm.nih.gov/pmc/articles/PMC7489367/ .

51. N. Kallioinen , A. Hill , M. S. Horswill , H. E. Ward , and M. O. Watson , “ Sources of Inaccuracy in the Measurement of Adult Patients′ resting

Blood Pressure in Clinical Settings: A Systematic Review ,” Journal of Hypertension 35 , no. 3 ( 2017 ): 421 – 441 ,

https://doi.org/10.1097/HJH.0000000000001197 .

52. H. Al Ghorani , F. Götzinger , M. Böhm , and F. Mahfoud , “ Arterial Hypertension—Clinical Trials Update 2021 ,” Nutrition, Metabolism and

Cardiovascular Diseases 32 , no. 1 ( 2022 ): 21 – 231 .
