# Supplementary material for: Effect of Antihypertensive Treatment on Hypotension, Mortality and Length of Stay in Orthopedic Trauma and First Detected High Blood Pressure Adults in a Large Urban Hospital: A Retrospective Cohort Study
Source: J Clin Hypertens (Greenwich). 2026 Jan 30;28(2):e70210. doi: 10.1111/jch.70210 (PMC12856957; doi:10.1111/jch.70210)
Supplement: Supplementary file 2 — Supporting file 2: jch70210‐sup‐0002‐SuppMat.docx [file JCH-28-e70210-s002.docx]

Análisis Final: con y sin imputación, subtipos de hipotensión, estancia mayor y menor 60 días, análisis sensibilidad y múltiples desenlaces.

Carlos Atencia

2024-07-24

# Protocolo R

# PREPARACION Y VISUALIZACION

#Llamamos la base de datos y abrimos su ubicacion--------------
library(readxl)
Base_de_datos_HTA <- read_excel("C:/Users/Usuario/Desktop/Maestria Epidemiologia Clinica/Investigacion/Codigos analisis R/Base de datos HTA.xlsx")
View(Base_de_datos_HTA)

# Echamos un vistazo y renombramos la base de datos

DBhtatx <- Base_de_datos_HTA

# Cargamos los paquetes------------
library(medicaldata)
library(summarytools)

##
## Attaching package: 'summarytools'

## The following object is masked from 'package:tibble':
##
## view

install.packages("tidyverse")

library(dplyr)
library(dslabs)
library(haven)
library(foreign)
library(ggplot2)
library(knitr)
library(tidyverse)
library(gtsummary)

#print(dfSummary(DBhtatx,valid.col = FALSE),method= "render")

# Estructura de la base de datos y nombres de columnas
str(DBhtatx)

## tibble [741 × 58] (S3: tbl_df/tbl/data.frame)
## $ nombre : chr [1:741] "Maria cano" "Luis hernandez" "Laura estefania ramirez rendon" "Paula andrea higuita taborda" ...
## $ id : chr [1:741] "21499858" "8295987" "1152462352" "1038926457" ...
## $ sexo : num [1:741] 0 1 0 0 1 1 1 1 1 0 ...
## $ raza : num [1:741] 2 2 2 2 2 2 2 2 2 2 ...
## $ edad : num [1:741] 68 73 26 19 33 57 39 57 41 41 ...
## $ ingreso : POSIXct[1:741], format: "2023-06-10" "2023-05-28" ...
## $ egreso : POSIXct[1:741], format: "2023-06-24" "2023-06-16" ...
## $ estancia : num [1:741] 14 19 9 30 5 4 14 15 15 18 ...
## $ muerte : num [1:741] 0 0 0 0 0 0 0 0 0 0 ...
## $ pas : num [1:741] 180 142 146 141 195 157 141 164 155 171 ...
## $ pad : num [1:741] 92 83 117 80 89 94 84 115 100 101 ...
## $ hipot : num [1:741] 1 1 1 1 1 0 0 0 0 0 ...
## $ hipotPAM : num [1:741] 61 61 57 61 62 89 66 79 80 69 ...
## $ hipot_lev : num [1:741] 0 0 0 0 0 0 0 0 0 0 ...
## $ hipot_md : num [1:741] 0 0 0 0 0 0 0 0 0 0 ...
## $ hipot_vaso : num [1:741] 0 0 0 0 0 0 0 0 0 0 ...
## $ antihta : num [1:741] 1 0 0 0 0 0 0 0 0 0 ...
## $ clase_med : num [1:741] 6 0 0 0 0 0 0 0 0 0 ...
## $ ddd : num [1:741] 5 1 1 1 1 1 1 1 1 1 ...
## $ horario_med: num [1:741] 2 0 0 0 0 0 0 0 0 0 ...
## $ mg_med : num [1:741] 10 0 0 0 0 0 0 0 0 0 ...
## $ dias_med : num [1:741] 12 NA NA NA NA NA NA NA NA NA ...
## $ ddp : num [1:741] 48 0 0 0 0 0 0 0 0 0 ...
## $ freccard : num [1:741] 89 60 113 110 86 100 98 77 116 144 ...
## $ glasgow : num [1:741] 15 15 15 15 15 15 15 15 15 15 ...
## $ pasrts : num [1:741] 110 90 118 101 124 135 114 143 100 99 ...
## $ rts : num [1:741] 120.5 97.4 133.4 120 129.9 ...
## $ fractura : num [1:741] 13 5 13 10 11 13 13 1 13 13 ...
## $ gustillo : num [1:741] 1 1 1 3 1 1 1 1 1 1 ...
## $ qxco : POSIXct[1:741], format: "2023-06-15" "2023-05-31" ...
## $ lev : num [1:741] 11601 7990 10360 20415 28900 ...
## $ aines : num [1:741] 1 1 1 1 1 1 1 1 1 1 ...
## $ opioid : num [1:741] 0 1 0 1 1 1 1 1 1 1 ...
## $ infx : num [1:741] 0 1 1 1 1 0 0 0 1 0 ...
## $ etev : num [1:741] 0 0 0 0 0 0 0 0 0 0 ...
## $ dolor : num [1:741] 2 1 2 3 3 2 1 2 3 2 ...
## $ insomnio : num [1:741] 1 0 0 1 1 0 0 1 1 0 ...
## $ constipa : num [1:741] 1 0 0 1 0 0 0 0 1 1 ...
## $ ansiedad : num [1:741] 1 0 0 1 0 0 0 0 1 0 ...
## $ hb : chr [1:741] "14.6" "9.7" "10.7" "8.4" ...
## $ creat : chr [1:741] "0.57" "0.78" "0.81" "0.42" ...
## $ peso : num [1:741] 73 69 62 80 66 85 60 75 75 78 ...
## $ fuma : num [1:741] 0 0 0 0 0 0 0 0 0 0 ...
## $ alcohol : num [1:741] 0 0 0 0 0 1 1 0 1 0 ...
## $ obeso : num [1:741] 0 0 1 0 0 0 0 0 0 1 ...
## $ diabetes : num [1:741] 0 1 0 0 0 0 0 0 0 0 ...
## $ iam : num [1:741] 0 0 0 0 0 0 0 0 0 0 ...
## $ ecv : num [1:741] 0 0 0 0 0 0 0 0 0 0 ...
## $ Columna43 : chr [1:741] "0" "0" "0" "0" ...
## $ Columna44 : logi [1:741] NA NA NA NA NA NA ...
## $ Columna45 : logi [1:741] NA NA NA NA NA NA ...
## $ Columna46 : logi [1:741] NA NA NA NA NA NA ...
## $ Columna47 : logi [1:741] NA NA NA NA NA NA ...
## $ Columna48 : logi [1:741] NA NA NA NA NA NA ...
## $ Columna49 : logi [1:741] NA NA NA NA NA NA ...
## $ Columna50 : logi [1:741] NA NA NA NA NA NA ...
## $ Columna51 : logi [1:741] NA NA NA NA NA NA ...
## $ Columna52 : logi [1:741] NA NA NA NA NA NA ...

colnames(DBhtatx)

## [1] "nombre" "id" "sexo" "raza" "edad"
## [6] "ingreso" "egreso" "estancia" "muerte" "pas"
## [11] "pad" "hipot" "hipotPAM" "hipot_lev" "hipot_md"
## [16] "hipot_vaso" "antihta" "clase_med" "ddd" "horario_med"
## [21] "mg_med" "dias_med" "ddp" "freccard" "glasgow"
## [26] "pasrts" "rts" "fractura" "gustillo" "qxco"
## [31] "lev" "aines" "opioid" "infx" "etev"
## [36] "dolor" "insomnio" "constipa" "ansiedad" "hb"
## [41] "creat" "peso" "fuma" "alcohol" "obeso"
## [46] "diabetes" "iam" "ecv" "Columna43" "Columna44"
## [51] "Columna45" "Columna46" "Columna47" "Columna48" "Columna49"
## [56] "Columna50" "Columna51" "Columna52"

# La variable de nombre es la que define los sujetos aun no ingresados
nchar(DBhtatx$nombre)

## [1] 10 14 30 28 23 24 25 26 21 25 20 31 19 30 29 29 29 24 26 31 31 33 21 34 21
## [26] 29 28 22 28 28 29 24 21 27 31 16 25 26 27 33 21 25 28 23 31 31 23 23 19 26
## [51] 27 24 31 24 40 29 25 31 20 30 25 30 25 18 26 28 28 25 23 28 24 32 24 20 22
## [76] 28 22 33 29 28 15 28 24 29 29 25 30 27 20 26 26 31 30 36 21 26 32 27 24 29
## [101] 36 32 29 20 28 25 25 29 24 30 27 28 25 36 25 34 21 29 25 27 23 31 25 28 27
## [126] 17 30 32 33 32 27 22 28 20 29 29 39 19 22 28 30 24 20 29 22 32 26 22 22 31
## [151] 22 24 26 31 26 26 23 22 30 31 21 26 27 27 33 26 29 23 29 26 18 27 23 29 26
## [176] 26 16 24 28 28 31 22 25 22 31 32 21 22 25 23 23 29 35 30 29 32 29 15 29 31
## [201] 32 28 27 18 29 25 29 30 32 26 28 24 29 29 26 34 27 26 25 22 28 26 28 32 32
## [226] 35 29 32 29 32 23 26 24 30 22 30 21 22 19 31 30 26 25 26 23 23 27 20 29 24
## [251] 27 30 27 30 34 31 28 29 25 30 27 17 36 31 21 23 37 24 26 25 27 22 17 24 24
## [276] 25 30 29 19 25 28 17 27 26 27 21 30 26 28 28 22 28 30 24 29 28 27 26 30 29
## [301] 24 28 34 28 32 27 24 22 23 32 27 20 34 27 29 20 30 10 31 28 26 27 28 20 20
## [326] 21 21 26 27 33 23 26 27 30 28 23 27 31 18 21 27 26 24 28 27 20 22 31 25 23
## [351] 25 26 25 24 28 31 23 29 29 21 26 26 26 22 25 31 27 30 30 28 27 35 24 23 31
## [376] 29 31 28 28 30 20 22 29 27 21 31 18 35 23 17 27 25 27 22 28 23 25 22 23 23
## [401] 28 27 18 33 24 24 21 27 23 24 27 29 27 22 23 25 29 22 28 27 30 29 39 32 28
## [426] 33 26 27 29 30 27 27 31 23 8 20 34 33 32 23 32 21 28 31 25 27 26 23 27 31
## [451] 26 35 30 27 31 26 29 28 31 26 27 22 21 28 28 30 23 27 36 29 29 21 28 33 29
## [476] 24 27 28 29 30 24 22 22 24 29 28 26 30 25 28 25 27 31 24 28 27 22 27 26 23
## [501] 34 30 29 18 31 28 27 31 29 30 30 24 29 23 28 29 29 13 24 31 30 29 21 24 28
## [526] 29 29 30 18 25 29 20 25 29 22 26 23 30 19 16 25 27 30 22 24 30 28 20 22 31
## [551] 25 28 26 28 19 29 29 29 24 27 29 19 29 25 31 34 27 28 32 30 31 23 26 34 34
## [576] 30 24 24 26 26 28 26 28 24 26 29 27 22 35 28 25 33 29 24 25 29 24 26 27 26
## [601] 26 25 23 23 25 24 26 27 21 25 15 23 26 22 29 24 31 22 27 28 23 22 25 26 23
## [626] 27 23 20 25 25 27 28 28 28 24 24 26 27 29 29 30 22 24 31 32 24 32 23 23 23
## [651] 29 29 27 28 17 25 27 21 28 24 25 24 29 26 26 30 26 34 31 29 30 26 20 28 25
## [676] 22 25 25 23 30 28 30 29 26 24 30 26 30 27 21 25 31 21 26 29 18 31 22 26 29
## [701] 26 20 24 25 26 22 23 28 28 21 28 22 27 23 34 31 19 31 27 21 28 26 31 19 20
## [726] 22 19 32 27 30 25 28 28 27 30 32 27 29 30 NA NA

#Los nombres tienen en promedio 26.3 letras


mean(nchar(DBhtatx$nombre), na.rm = T)

## [1] 26.41137

# % perdidos por columna (variable)
sum(is.na(DBhtatx))

## [1] 7717

porcentajeMiss <- function(x) {sum(is.na(x)) / length(x)*100}

apply(DBhtatx, 2, porcentajeMiss)

## nombre id sexo raza edad ingreso
## 0.2699055 0.2699055 0.2699055 0.4048583 0.4048583 0.2699055
## egreso estancia muerte pas pad hipot
## 0.2699055 0.2699055 0.2699055 0.2699055 0.2699055 0.2699055
## hipotPAM hipot_lev hipot_md hipot_vaso antihta clase_med
## 0.2699055 0.2699055 0.2699055 0.2699055 0.2699055 0.2699055
## ddd horario_med mg_med dias_med ddp freccard
## 0.2699055 0.4048583 0.4048583 88.7989204 0.5398111 0.2699055
## glasgow pasrts rts fractura gustillo qxco
## 0.2699055 0.4048583 0.1349528 0.2699055 3.1039136 2.0242915
## lev aines opioid infx etev dolor
## 0.2699055 0.2699055 0.2699055 0.4048583 0.2699055 0.2699055
## insomnio constipa ansiedad hb creat peso
## 0.2699055 0.2699055 0.2699055 9.9865047 19.0283401 5.9379217
## fuma alcohol obeso diabetes iam ecv
## 0.2699055 0.2699055 0.2699055 0.2699055 0.2699055 0.2699055
## Columna43 Columna44 Columna45 Columna46 Columna47 Columna48
## 0.2699055 100.0000000 100.0000000 100.0000000 100.0000000 100.0000000
## Columna49 Columna50 Columna51 Columna52
## 100.0000000 100.0000000 100.0000000 100.0000000

# Error de R con el dispositivo de graficos: resolvio al reiniciar

# El numero de missing es sustancial pero se debe a que se crearon filas que no se han diligenciado en essta BD
library(visdat)
sum(is.na(DBhtatx))

## [1] 7717

map_dbl(DBhtatx,.f=function(x) {sum(is.na(x))})

## nombre id sexo raza edad ingreso
## 2 2 2 3 3 2
## egreso estancia muerte pas pad hipot
## 2 2 2 2 2 2
## hipotPAM hipot_lev hipot_md hipot_vaso antihta clase_med
## 2 2 2 2 2 2
## ddd horario_med mg_med dias_med ddp freccard
## 2 3 3 658 4 2
## glasgow pasrts rts fractura gustillo qxco
## 2 3 1 2 23 15
## lev aines opioid infx etev dolor
## 2 2 2 3 2 2
## insomnio constipa ansiedad hb creat peso
## 2 2 2 74 141 44
## fuma alcohol obeso diabetes iam ecv
## 2 2 2 2 2 2
## Columna43 Columna44 Columna45 Columna46 Columna47 Columna48
## 2 741 741 741 741 741
## Columna49 Columna50 Columna51 Columna52
## 741 741 741 741

vis_miss(DBhtatx)


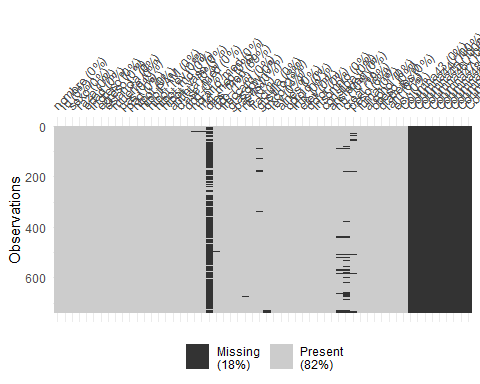


# Patron de perdida desde cual fila


# Por medio de la funcion select seleccionamos las variables solicitadas
DBhtatx <- DBhtatx %>%
 dplyr:::select(1:49)


colnames(DBhtatx)

## [1] "nombre" "id" "sexo" "raza" "edad"
## [6] "ingreso" "egreso" "estancia" "muerte" "pas"
## [11] "pad" "hipot" "hipotPAM" "hipot_lev" "hipot_md"
## [16] "hipot_vaso" "antihta" "clase_med" "ddd" "horario_med"
## [21] "mg_med" "dias_med" "ddp" "freccard" "glasgow"
## [26] "pasrts" "rts" "fractura" "gustillo" "qxco"
## [31] "lev" "aines" "opioid" "infx" "etev"
## [36] "dolor" "insomnio" "constipa" "ansiedad" "hb"
## [41] "creat" "peso" "fuma" "alcohol" "obeso"
## [46] "diabetes" "iam" "ecv" "Columna43"

# Por medio de la funcion slice seleccionamos las filas solicitadas
DBhtatx <- DBhtatx |> slice(1:739) # mientras obtenemos el total de 700

# Convert character vector to numeric de hemoglobina y creatinina
DBhtatx$hb <- as.numeric(DBhtatx$hb)
DBhtatx$creat <- as.numeric(DBhtatx$creat)


# Check the class of the converted vector
class(DBhtatx$hb)

## [1] "numeric"

typeof(DBhtatx$hb)

## [1] "double"

class(DBhtatx$creat)

## [1] "numeric"

typeof(DBhtatx$creat)

## [1] "double"

table(DBhtatx$alcohol)

##
## 0 1
## 310 429

DBhtatx$alcohol <- as.factor(DBhtatx$alcohol)
DBhtatx$alcohol <- as.factor(DBhtatx$constipa)


# Visualizacion de datos perdidos dias medicamento
print(vis_dat(DBhtatx))


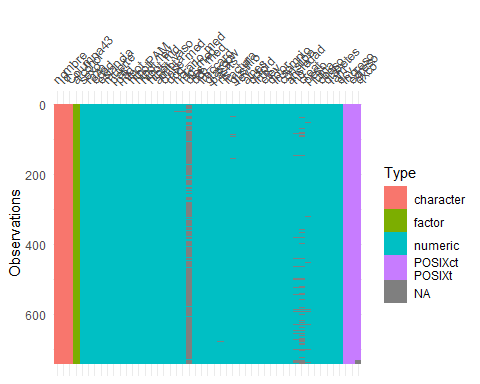


hist(DBhtatx$creat)


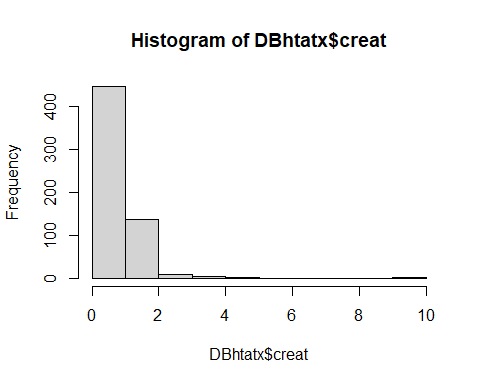


hist(DBhtatx$hb)
quantiles <- quantile(DBhtatx$hb, na.rm = T)
abline(v = quantiles[1], col = "green", lwd = 2, lty = 2)
abline(v = quantiles[2], col = "tomato", lwd = 2, lty = 2)
abline(v = quantiles[3], col = "tomato", lwd = 2, lty = 2)
abline(v = quantiles[4], col = "green", lwd = 2, lty = 2)
text(quantiles[1], par("usr")[4] - 1.5, labels = paste("25th Quantile:", round(quantiles[1], 2)), col = "cyan", pos = 4)


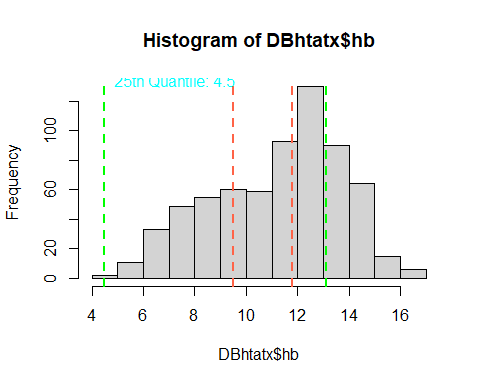


hist(DBhtatx$dias_med)


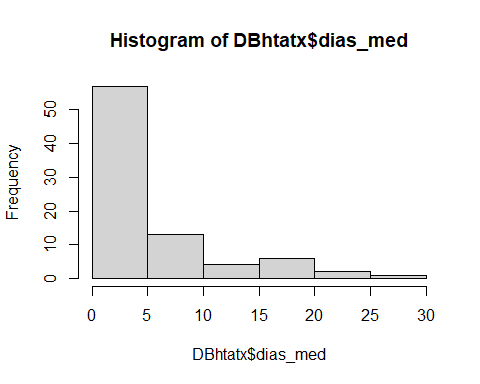


quantile(DBhtatx$dias_med, na.rm = T)

## 0% 25% 50% 75% 100%
## 1.0 2.5 4.0 7.0 29.0

#cor(DBhtatx$estancia,DBhtatx$dias_med)

# Identificamos este dato extremo para reportarlo a Marlyn y corregir
DBhtatx %>%filter(DBhtatx$dias_med>50) |>
 dplyr:::select("id", "nombre")

## # A tibble: 0 × 2
## # ℹ 2 variables: id <chr>, nombre <chr>

# Crear nueva variable de tiempo a cirugia en dias
DBhtatx <- DBhtatx |>
 mutate(tiempo_qxco = qxco - ingreso) # restando ell tiempo de ingreso al de la cirugia

# convertir segundos a dias
DBhtatx <- DBhtatx |>
 mutate(tiempo_qxco = tiempo_qxco/86400)

# Transformar a numerica
DBhtatx$tiempo_qxco <- as.numeric(DBhtatx$tiempo_qxco)

# Al ver histograma NO hay 11 datos negativos
hist(DBhtatx$tiempo_qxco)


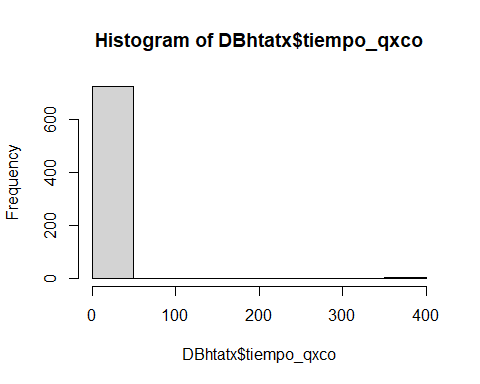


# Localicemoslos para informar a Marlyn y corregir
DBhtatx %>%
 filter(DBhtatx$tiempo_qxco<0) |>
 dplyr:::select("id", "nombre")

## # A tibble: 0 × 2
## # ℹ 2 variables: id <chr>, nombre <chr>

DBhtatx %>%
 filter(DBhtatx$tiempo_qxco>50) |>
 dplyr:::select("id", "nombre")

## # A tibble: 2 × 2
## id nombre
## <chr> <chr>
## 1 98491416 Robinson de jesus espinosa aguirre
## 2 71293234 Juan david gomez mazo

# la tabla lo confirma
table(DBhtatx$tiempo_qxco<0)

##
## FALSE
## 726

# la mediana y la media de tiempo quirurgico
median(DBhtatx$tiempo_qxco, na.rm = T)

## [1] 3

quantile(DBhtatx$tiempo_qxco, na.rm = T)

## 0% 25% 50% 75% 100%
## 0 2 3 5 368

# Reubicaremos la variable para presentar en tabla1
DBhtatx <- DBhtatx |>
 relocate(tiempo_qxco, .after = qxco)

# Ajuste para el RTS calcularemos FR con base en FC

DBhtatx <- DBhtatx |>
 mutate(frecresp = freccard/4)

# Visualizamos FC y FR
hist(DBhtatx$freccard)


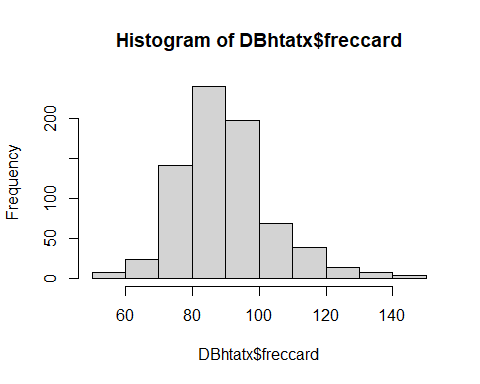


hist(DBhtatx$frecresp)


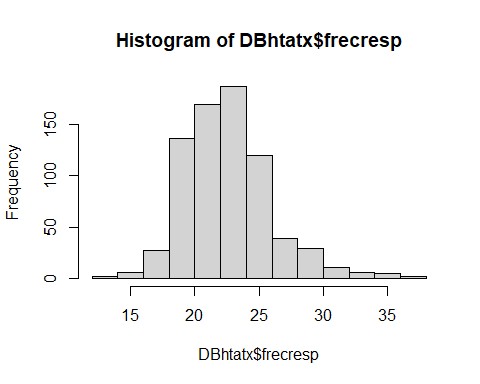


# Codificaremos la variables glasgow, pasrts y frecresp (@champion)
# GLASGOW
# Define the breaks for the bins
breaks_glasgow <- c(5, 8, 12, 15, Inf)

# Use cut() to create a variable numerica pero diferente
DBhtatx$glasgow_coded <- cut(DBhtatx$glasgow, breaks = breaks_glasgow, labels = FALSE, right = FALSE)

hist(DBhtatx$glasgow_coded)


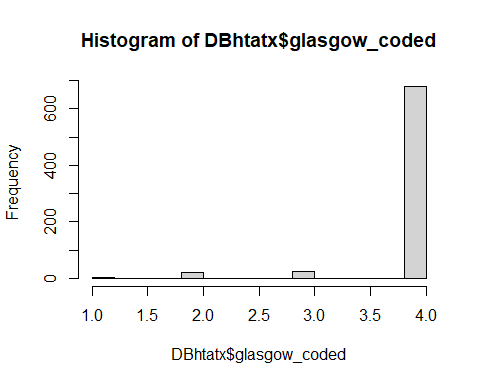


#PAS RTS
# Define the breaks for the bins
breaks_pasrts <- c(49, 75, 89, 90, Inf)

# Use cut() to create a variable numerica pero diferente
DBhtatx$pasrts_coded <- cut(DBhtatx$pasrts, breaks = breaks_pasrts, labels = FALSE, right = FALSE)

hist(DBhtatx$pasrts_coded)


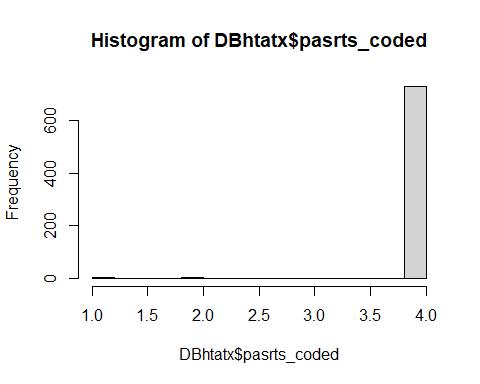


# frecresp
# Define the breaks for the bins
breaks_frecresp <- c(5, 9, 29, 30, Inf)

# Use cut() to create a variable numerica pero diferente
DBhtatx$frecresp_coded <- cut(DBhtatx$frecresp, breaks = breaks_frecresp, labels = FALSE, right = FALSE)

hist(DBhtatx$frecresp_coded)


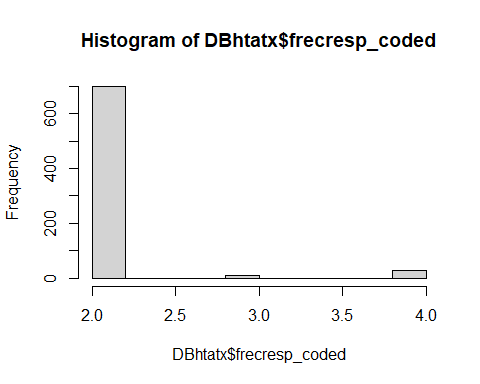


# reubicamos para prestentacion
DBhtatx <- DBhtatx |>
 relocate(frecresp, .after = freccard)

# Ahora un RTS calculado a partir de la GCS, PAS y FR (No de la FC) codificadas
DBhtatx <- DBhtatx |>
 mutate(cRTS = 0.9368*glasgow_coded + 0.7326*pasrts_coded + 0.2908* frecresp_coded -3.5718)

DBhtatx <- DBhtatx |>
 relocate(cRTS, .after = pasrts)

# Visualizamos RTS
hist(DBhtatx$cRTS)


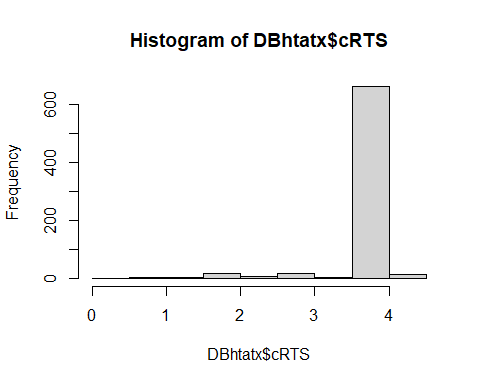


table(DBhtatx$constipa)

##
## 0 1
## 553 186

# Los dias de medicamentos en los que no estuvieron expuestos fue cero "0"
DBhtatx$dias_med <- ifelse(is.na(DBhtatx$dias_med), "0", DBhtatx$dias_med)

summary(DBhtatx$dias_med)

## Length Class Mode
## 739 character character

table(DBhtatx$dias_med)

##
## 0 1 10 12 14 17 2 20 21 25 29 3 4 5 6 7 8 9
## 656 16 2 1 3 3 5 3 1 1 1 13 15 8 2 6 2 1

DBhtatx$dias_med <- as.numeric(DBhtatx$dias_med)
summary(DBhtatx$dias_med)

## Min. 1st Qu. Median Mean 3rd Qu. Max.
## 0.000 0.000 0.000 0.682 0.000 29.000

DBhtatx %>%
 group_by(antihta) %>%
 reframe(DBhtatx$dias_med)

## # A tibble: 1,478 × 2
## antihta `DBhtatx$dias_med`
## <dbl> <dbl>
## 1 0 12
## 2 0 0
## 3 0 0
## 4 0 0
## 5 0 0
## 6 0 0
## 7 0 0
## 8 0 0
## 9 0 0
## 10 0 0
## # ℹ 1,468 more rows

DBhtatx$dias_med

## [1] 12 0 0 0 0 0 0 0 0 0 0 0 0 0 6 7 0 0 0 0 0 0 0 0 0
## [26] 29 0 0 14 1 0 1 7 0 0 0 0 0 0 0 0 2 1 3 0 21 0 9 1 0
## [51] 0 0 0 0 0 0 0 0 0 5 1 0 0 0 0 0 0 0 0 0 0 0 0 1 0
## [76] 0 0 0 1 0 0 1 0 0 0 0 0 0 0 0 0 0 0 0 0 0 0 4 0 0
## [101] 0 4 0 0 0 7 0 0 0 0 0 0 0 0 0 0 0 5 0 0 0 0 0 0 0
## [126] 0 0 0 0 0 0 0 0 0 0 0 0 0 0 0 0 0 0 0 0 0 0 0 0 0
## [151] 0 0 0 0 0 0 0 0 0 0 0 0 0 0 0 4 1 0 0 0 25 0 0 0 0
## [176] 1 0 0 0 0 0 0 0 0 0 0 0 0 0 0 0 0 0 0 1 0 0 0 0 1
## [201] 0 1 0 0 0 0 0 0 0 0 0 0 0 17 0 20 0 0 0 20 0 0 0 0 0
## [226] 14 0 0 0 0 3 0 0 7 0 0 0 0 0 0 4 3 0 0 0 5 0 4 0 4
## [251] 2 0 0 0 0 6 0 0 3 0 0 3 0 0 0 4 0 0 0 10 0 0 5 0 0
## [276] 10 0 0 0 0 0 0 5 0 0 0 3 0 0 0 0 8 0 20 0 0 0 0 0 0
## [301] 0 0 0 0 0 0 0 3 0 0 0 0 0 0 0 0 0 4 0 0 0 1 0 0 0
## [326] 0 17 0 0 0 0 0 0 0 0 0 0 0 0 0 0 0 0 0 0 1 4 0 0 0
## [351] 0 0 0 0 0 0 3 0 14 0 0 0 0 0 0 0 0 17 0 0 0 0 0 0 0
## [376] 4 0 0 0 0 0 0 0 3 0 0 5 0 0 0 0 0 0 0 4 0 0 0 0 1
## [401] 0 0 0 3 0 7 0 0 0 0 0 0 0 0 0 0 0 0 0 0 0 0 0 0 0
## [426] 0 0 0 0 0 0 0 0 0 4 0 0 0 0 0 0 0 0 8 0 4 0 0 0 0
## [451] 0 0 0 0 0 0 0 0 0 0 0 0 0 2 0 0 0 0 0 0 0 0 7 0 0
## [476] 0 5 0 0 0 0 0 0 0 0 0 0 0 0 0 0 0 0 0 0 0 0 0 0 0
## [501] 0 0 0 0 0 0 0 0 0 0 0 0 0 0 0 0 0 0 0 0 0 0 0 0 0
## [526] 0 0 0 0 0 0 0 0 0 0 0 0 0 0 0 0 0 0 0 0 0 0 0 0 0
## [551] 2 0 0 0 0 0 0 0 0 0 0 0 0 0 0 0 0 0 0 0 0 0 0 0 2
## [576] 0 0 0 0 0 0 0 0 4 0 0 0 0 0 0 0 0 0 0 0 0 0 0 0 0
## [601] 0 0 3 0 0 0 0 0 0 0 0 0 0 0 0 0 0 0 0 0 0 0 0 0 0
## [626] 0 0 0 0 0 0 0 0 0 0 0 0 0 0 0 0 0 0 0 0 0 0 0 0 0
## [651] 0 0 0 0 0 0 0 0 0 0 0 0 0 0 0 0 0 0 0 0 0 0 0 0 0
## [676] 0 0 0 0 0 0 0 0 0 0 0 0 0 0 0 0 0 0 0 0 0 0 0 0 0
## [701] 0 0 0 3 0 0 0 0 0 0 0 0 0 0 0 0 0 0 0 0 0 0 0 5 3
## [726] 4 0 0 0 0 0 0 0 0 0 0 0 0 0

hist(DBhtatx$dias_med)


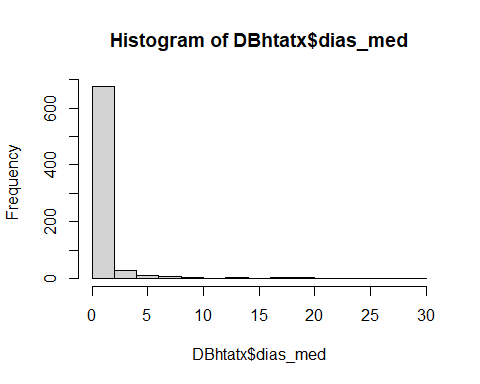


# Missing: manejo de datos perdidos de variables continuas. Impuitacion------
library(mice)

##
## Attaching package: 'mice'

## The following object is masked from 'package:stats':
##
## filter

## The following objects are masked from 'package:base':
##
## cbind, rbind

columns <- c("peso","raza","pasrts","cRTS")
imputed_DBhtatx <- mice(DBhtatx[,columns],m = 5,
 maxit = 5, method = "mean",seed = 2018,print=T)

##
## iter imp variable
## 1 1 peso raza pasrts cRTS
## 1 2 peso raza pasrts cRTS
## 1 3 peso raza pasrts cRTS
## 1 4 peso raza pasrts cRTS
## 1 5 peso raza pasrts cRTS
## 2 1 peso raza pasrts cRTS
## 2 2 peso raza pasrts cRTS
## 2 3 peso raza pasrts cRTS
## 2 4 peso raza pasrts cRTS
## 2 5 peso raza pasrts cRTS
## 3 1 peso raza pasrts cRTS
## 3 2 peso raza pasrts cRTS
## 3 3 peso raza pasrts cRTS
## 3 4 peso raza pasrts cRTS
## 3 5 peso raza pasrts cRTS
## 4 1 peso raza pasrts cRTS
## 4 2 peso raza pasrts cRTS
## 4 3 peso raza pasrts cRTS
## 4 4 peso raza pasrts cRTS
## 4 5 peso raza pasrts cRTS
## 5 1 peso raza pasrts cRTS
## 5 2 peso raza pasrts cRTS
## 5 3 peso raza pasrts cRTS
## 5 4 peso raza pasrts cRTS
## 5 5 peso raza pasrts cRTS

complete_DBhtatx <- mice::complete(imputed_DBhtatx)

DBhtatx[, columns] <- complete_DBhtatx[, columns]

print(vis_dat(DBhtatx))


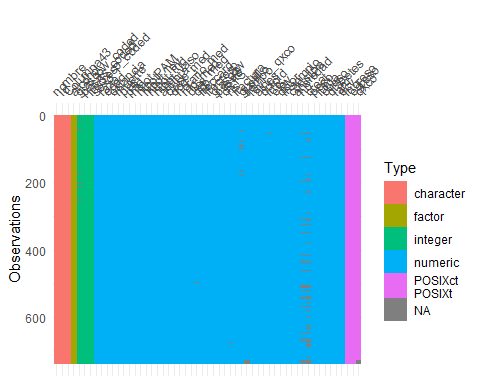


## Visualizacion-----------------------------------------------
#DBhtatx<-na.omit(DBhtatx)}
library(ggplot2)

# Check the levels of the factor variable
levels(DBhtatx$antihta)

## NULL

# If there are unwanted levels, we need to clean them
# DBhtatx$antihta <- droplevels(DBhtatx$antihta)

# Verify again to ensure there are no unintended levels

sum(is.na(DBhtatx$antihta))

## [1] 0

which(is.na(DBhtatx$antihta))

## integer(0)

print(which(is.na(DBhtatx$antihta)))

## integer(0)

library(corrplot)

## corrplot 0.92 loaded

DBhtatx %>% dplyr:::select(muerte,hipot,edad,estancia,pas,pad,hipotPAM,ddd,mg_med,dias_med,ddp,freccard,glasgow,pasrts,rts,lev,dolor,hb,creat,peso) |> na.omit()|>cor()|>corrplot(addCoef.col = T)


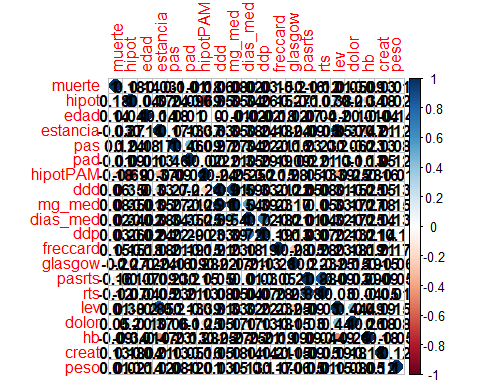


sum(is.na(DBhtatx$estancia))

## [1] 0

#DBhtatx <- na.omit(DBhtatx[, c("estancia", "antihta")])

# Plotting the density curves again
ggplot(DBhtatx, aes(x = estancia, fill = antihta, color = antihta)) +
 geom_density(alpha = 0.8)


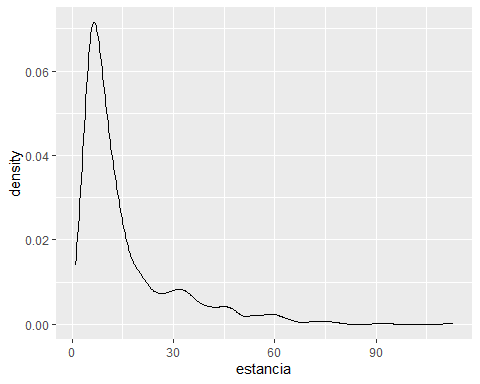


#
# ggplot(data= DBhtatx, mapping = aes(x= mg_med, y=estancia, color= "blue")) + geom_point(position = "jitter")
#
# ggplot(data= DBhtatx |> filter(hipotension == "con hipotension"), mapping = aes(x= dias_med, y=estancia, color= "blue", shape = hipotension)) + geom_point(position = "jitter")
#
# ggplot(data= DBhtatx, mapping = aes(x= ddp, y=estancia, color= "blue")) + geom_point(position = "jitter")
#
# ggplot(data= DBhtatx, mapping = aes(x= ddd, y=estancia, color= "blue")) + geom_point(position = "jitter")


# Especificos a cada variable------------------------------------


ggplot(DBhtatx, aes(x=estancia, color = antihta, group= antihta)) +
 geom_density(alpha = 0.5)


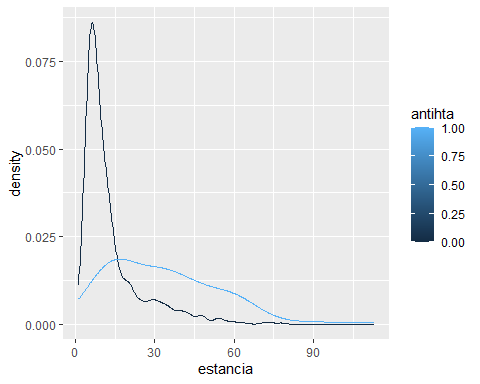


colnames(DBhtatx)

## [1] "nombre" "id" "sexo" "raza"
## [5] "edad" "ingreso" "egreso" "estancia"
## [9] "muerte" "pas" "pad" "hipot"
## [13] "hipotPAM" "hipot_lev" "hipot_md" "hipot_vaso"
## [17] "antihta" "clase_med" "ddd" "horario_med"
## [21] "mg_med" "dias_med" "ddp" "freccard"
## [25] "frecresp" "glasgow" "pasrts" "cRTS"
## [29] "rts" "fractura" "gustillo" "qxco"
## [33] "tiempo_qxco" "lev" "aines" "opioid"
## [37] "infx" "etev" "dolor" "insomnio"
## [41] "constipa" "ansiedad" "hb" "creat"
## [45] "peso" "fuma" "alcohol" "obeso"
## [49] "diabetes" "iam" "ecv" "Columna43"
## [53] "glasgow_coded" "pasrts_coded" "frecresp_coded"

# Variables dicotomicas: evaluar las frecuencias previas a la reccodificacion
table(DBhtatx$muerte)

##
## 0 1
## 728 11

table(DBhtatx$hipot)

##
## 0 1
## 557 182

table(DBhtatx$antihta)

##
## 0 1
## 656 83

table(DBhtatx$sexo)

##
## 0 1
## 170 569

table(DBhtatx$fuma)

##
## 0 1 2
## 392 346 1

table(DBhtatx$raza)

##
## 2 2.00948509485095 3
## 731 1 7

DBhtatx$raza <- gsub("2.00948509485095", "3", DBhtatx$raza)

table(DBhtatx$raza)

##
## 2 3
## 731 8

table(DBhtatx$gustillo)

##
## 0 1 2 3
## 277 136 150 155

table(DBhtatx$fractura)

##
## 1 2 3 4 5 6 7 8 9 10 11 12 13 14 15
## 13 4 45 7 57 1 5 9 38 41 186 23 252 24 34

table(DBhtatx$clase_med)

##
## 0 1 3 6 7 10 11 13 14
## 656 9 5 8 5 1 26 1 28

table(DBhtatx$horario_med)

##
## 0 1 2 3 4
## 655 26 41 15 1

# Variables a convertirlas en factor---------------

columnasAFactor <- c("sexo" , "raza" , "hipot_lev", "hipot_md" , "hipot_vaso","aines", "opioid", "infx","etev", "insomnio", "constipa", "ansiedad",
 "fuma" , "alcohol" , "obeso", "diabetes" , "iam" , "ecv", "gustillo", "fractura", "hipot", "muerte","antihta" , "clase_med", "horario_med")
# Agrupoandolas
DBhtatx[columnasAFactor] <- lapply(DBhtatx[columnasAFactor], factor)

glimpse(DBhtatx)

## Rows: 739
## Columns: 55
## $ nombre <chr> "Maria cano", "Luis hernandez", "Laura estefania ramire…
## $ id <chr> "21499858", "8295987", "1152462352", "1038926457", "103…
## $ sexo <fct> 0, 1, 0, 0, 1, 1, 1, 1, 1, 0, 0, 0, 1, 1, 1, 1, 1, 1, 1…
## $ raza <fct> 2, 2, 2, 2, 2, 2, 2, 2, 2, 2, 2, 2, 2, 2, 2, 2, 2, 2, 2…
## $ edad <dbl> 68, 73, 26, 19, 33, 57, 39, 57, 41, 41, 42, 43, 44, 44,…
## $ ingreso <dttm> 2023-06-10, 2023-05-28, 2023-06-01, 2023-12-14, 2023-1…
## $ egreso <dttm> 2023-06-24, 2023-06-16, 2023-06-10, 2024-01-13, 2024-0…
## $ estancia <dbl> 14, 19, 9, 30, 5, 4, 14, 15, 15, 18, 12, 37, 7, 28, 76,…
## $ muerte <fct> 0, 0, 0, 0, 0, 0, 0, 0, 0, 0, 0, 0, 0, 0, 0, 0, 0, 0, 0…
## $ pas <dbl> 180, 142, 146, 141, 195, 157, 141, 164, 155, 171, 159, …
## $ pad <dbl> 92, 83, 117, 80, 89, 94, 84, 115, 100, 101, 93, 95, 89,…
## $ hipot <fct> 1, 1, 1, 1, 1, 0, 0, 0, 0, 0, 1, 0, 1, 0, 1, 1, 0, 0, 0…
## $ hipotPAM <dbl> 61, 61, 57, 61, 62, 89, 66, 79, 80, 69, 64, 71, 64, 76,…
## $ hipot_lev <fct> 0, 0, 0, 0, 0, 0, 0, 0, 0, 0, 0, 0, 0, 0, 1, 1, 0, 0, 0…
## $ hipot_md <fct> 0, 0, 0, 0, 0, 0, 0, 0, 0, 0, 0, 0, 0, 0, 1, 1, 0, 0, 0…
## $ hipot_vaso <fct> 0, 0, 0, 0, 0, 0, 0, 0, 0, 0, 0, 0, 0, 0, 0, 1, 0, 0, 0…
## $ antihta <fct> 1, 0, 0, 0, 0, 0, 0, 0, 0, 0, 0, 0, 0, 0, 1, 1, 0, 0, 0…
## $ clase_med <fct> 6, 0, 0, 0, 0, 0, 0, 0, 0, 0, 0, 0, 0, 0, 1, 1, 0, 0, 0…
## $ ddd <dbl> 5.00, 1.00, 1.00, 1.00, 1.00, 1.00, 1.00, 1.00, 1.00, 1…
## $ horario_med <fct> 2, 0, 0, 0, 0, 0, 0, 0, 0, 0, 0, 0, 0, 0, 2, 2, 0, 0, 0…
## $ mg_med <dbl> 10.00, 0.00, 0.00, 0.00, 0.00, 0.00, 0.00, 0.00, 0.00, …
## $ dias_med <dbl> 12, 0, 0, 0, 0, 0, 0, 0, 0, 0, 0, 0, 0, 0, 6, 7, 0, 0, …
## $ ddp <dbl> 48, 0, 0, 0, 0, 0, 0, 0, 0, 0, 0, 0, 0, 0, 12, 14, 0, 0…
## $ freccard <dbl> 89, 60, 113, 110, 86, 100, 98, 77, 116, 144, 128, 117, …
## $ frecresp <dbl> 22.25, 15.00, 28.25, 27.50, 21.50, 25.00, 24.50, 19.25,…
## $ glasgow <dbl> 15, 15, 15, 15, 15, 15, 15, 15, 15, 15, 15, 15, 15, 15,…
## $ pasrts <dbl> 110.0000, 90.0000, 118.0000, 101.0000, 124.0000, 135.00…
## $ cRTS <dbl> 3.687400, 3.687400, 3.687400, 3.687400, 3.687400, 3.687…
## $ rts <dbl> 120.5192, 97.4340, 133.3592, 120.0326, 129.9032, 142.03…
## $ fractura <fct> 13, 5, 13, 10, 11, 13, 13, 1, 13, 13, 10, 11, 13, 13, 9…
## $ gustillo <fct> 1, 1, 1, 3, 1, 1, 1, 1, 1, 1, 2, 2, 1, 2, 1, 1, 1, 1, 1…
## $ qxco <dttm> 2023-06-15, 2023-05-31, 2023-06-06, 2023-12-17, 2023-1…
## $ tiempo_qxco <dbl> 5, 3, 5, 3, 4, 1, 14, 13, 14, 17, 9, 11, 5, 15, 24, 10,…
## $ lev <dbl> 11601, 7990, 10360, 20415, 28900, 3000, 4500, 15800, 98…
## $ aines <fct> 1, 1, 1, 1, 1, 1, 1, 1, 1, 1, 1, 1, 1, 1, 1, 1, 1, 1, 1…
## $ opioid <fct> 0, 1, 0, 1, 1, 1, 1, 1, 1, 1, 1, 1, 1, 1, 1, 1, 1, 1, 1…
## $ infx <fct> 0, 1, 1, 1, 1, 0, 0, 0, 1, 0, 0, 0, 0, 1, 1, 1, 1, 1, 1…
## $ etev <fct> 0, 0, 0, 0, 0, 0, 0, 0, 0, 0, 0, 0, 0, 0, 0, 0, 0, 0, 0…
## $ dolor <dbl> 2, 1, 2, 3, 3, 2, 1, 2, 3, 2, 3, 2, 2, 3, 3, 3, 3, 3, 3…
## $ insomnio <fct> 1, 0, 0, 1, 1, 0, 0, 1, 1, 0, 0, 0, 0, 1, 1, 1, 0, 1, 1…
## $ constipa <fct> 1, 0, 0, 1, 0, 0, 0, 0, 1, 1, 0, 0, 0, 1, 0, 1, 0, 1, 1…
## $ ansiedad <fct> 1, 0, 0, 1, 0, 0, 0, 0, 1, 0, 0, 0, 1, 1, 1, 1, 0, 1, 0…
## $ hb <dbl> 14.6, 9.7, 10.7, 8.4, 13.4, 14.5, NA, 13.5, 13.1, 12.3,…
## $ creat <dbl> 0.57, 0.78, 0.81, 0.42, 0.86, 0.77, NA, 0.87, 0.44, 0.8…
## $ peso <dbl> 73, 69, 62, 80, 66, 85, 60, 75, 75, 78, 60, 63, 72, 66,…
## $ fuma <fct> 0, 0, 0, 0, 0, 0, 0, 0, 0, 0, 0, 0, 0, 0, 2, 0, 0, 1, 1…
## $ alcohol <fct> 1, 0, 0, 1, 0, 0, 0, 0, 1, 1, 0, 0, 0, 1, 0, 1, 0, 1, 1…
## $ obeso <fct> 0, 0, 1, 0, 0, 0, 0, 0, 0, 1, 0, 0, 0, 0, 0, 0, 0, 1, 0…
## $ diabetes <fct> 0, 1, 0, 0, 0, 0, 0, 0, 0, 0, 0, 1, 0, 0, 0, 0, 0, 0, 0…
## $ iam <fct> 0, 0, 0, 0, 0, 0, 0, 0, 0, 0, 0, 0, 0, 0, 0, 0, 0, 0, 0…
## $ ecv <fct> 0, 0, 0, 0, 0, 0, 0, 0, 0, 0, 0, 0, 0, 0, 0, 0, 0, 0, 0…
## $ Columna43 <chr> "0", "0", "0", "0", "0", "0", "0", "0", "0", "0", "0", …
## $ glasgow_coded <int> 4, 4, 4, 4, 4, 4, 4, 4, 4, 4, 4, 4, 4, 4, NA, 4, 4, 4, …
## $ pasrts_coded <int> 4, 4, 4, 4, 4, 4, 4, 4, 4, 4, 4, 4, 4, 4, 4, 4, 4, 4, 4…
## $ frecresp_coded <int> 2, 2, 2, 2, 2, 2, 2, 2, 3, 4, 4, 3, 2, 2, 2, 4, 2, 2, 2…

DBhtatx$sexo <- factor(DBhtatx$sexo,
 levels = c(0,1),
 labels = c("Mujer", "Hombre"))

DBhtatx$raza <- factor(DBhtatx$raza,
 levels = c(2,3),
 labels = c("Mestizo", "Negro"))


DBhtatx$fuma <- factor(DBhtatx$fuma,
 levels = c(0,1,2),
 labels = c("No", "Si", "Si"))
table(DBhtatx$fuma)

##
## No Si
## 392 347

DBhtatx$gustillo <- factor(DBhtatx$gustillo,
 levels = c(0,1,2,3),
 labels = c("Cerrada", "GA1", "GA2", "GA3"))
# Gustillo tiene muchas categorias NAs que estamos seguros son Cerradas (En la HC gustillo es cerrada no se clasifica)
DBhtatx <- DBhtatx %>%
 mutate(gustillo = replace_na(gustillo, "Cerrada"))
# DBhtatx$gustillo[is.na(DBhtatx$gustillo)] <- "Cerrada" # otra forma

DBhtatx$fractura <- factor(DBhtatx$fractura,
 levels = c(1,2,3,4,5,7,8,9,10,11,12,13,14,15),
 labels = c("Clavicula" , "Hombro" , "Humero" , "Codo" , "Radio o cubito" , "Muneca"
 , "Mano" , "Pelvis" , "Cadera" , "Femur" , "Rodilla" , "Tibia - perone"
 , "Tobillo" , "Pie"))


# Visualizacion de fracturas
library(plotly)

##
## Attaching package: 'plotly'

## The following object is masked from 'package:ggplot2':
##
## last_plot

## The following object is masked from 'package:stats':
##
## filter

## The following object is masked from 'package:graphics':
##
## layout

H <- ggplot(data= DBhtatx, mapping= aes(x=fractura))+
 geom_bar() +
 labs(title = "Localizacion de Fractura", subtitle = "Hueso fracturado", caption = "Grafico 1", x= "Hueso", y = "Numero de casos")+
 theme(axis.text.x = element_text(angle = 45, hjust = 1))

ggplotly(H)

## PhantomJS not found. You can install it with webshot::install_phantomjs(). If it is installed, please make sure the phantomjs executable can be found via the PATH variable.


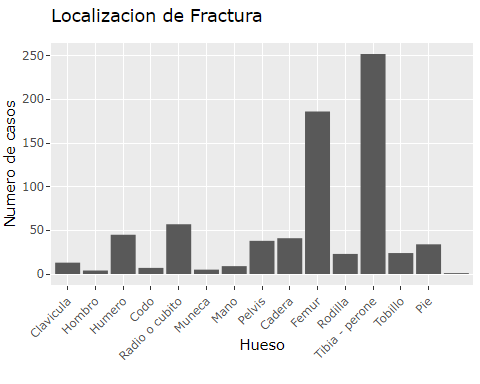


# Variables desenlace
table(DBhtatx$muerte)

##
## 0 1
## 728 11

table(DBhtatx$antihta)

##
## 0 1
## 656 83

table(DBhtatx$hipot)

##
## 0 1
## 557 182

# Codificacion de variables
table(DBhtatx$hipot_lev)

##
## 0 1
## 640 99

DBhtatx$hipot_lev <- factor(DBhtatx$hipot_lev,
 levels = c(1,0),
 labels = c("presente", "ausente"))


table(DBhtatx$hipot_lev)

##
## presente ausente
## 99 640

DBhtatx$hipot <- factor(DBhtatx$hipot,
 levels = c(1,0),
 labels = c("presente", "ausente"))

DBhtatx <- DBhtatx %>%
 mutate(Hipot = ifelse(DBhtatx$hipot=="presente",0,1))

table(DBhtatx$hipot)

##
## presente ausente
## 182 557

DBhtatx$hipot_md <- factor(DBhtatx$hipot_md,
 levels = c(1,0),
 labels = c("presente", "ausente"))


DBhtatx$hipot_vaso <- factor(DBhtatx$hipot_vaso,
 levels = c(1,0),
 labels = c("presente", "ausente"))


DBhtatx$muerte <- factor(DBhtatx$muerte,
 levels = c(0,1),
 labels = c("sobrevivio", "fallecio"))
table(DBhtatx$muerte)

##
## sobrevivio fallecio
## 728 11

DBhtatx$antihta <- factor(DBhtatx$antihta,
 levels = c(0,1),
 labels = c("No prescrito", "Prescrito"))
table(DBhtatx$antihta)

##
## No prescrito Prescrito
## 656 83

DBhtatx$horario_med <- factor(DBhtatx$horario_med,
 levels = c(0,1,2,3,4),
 labels = c("noMd", "c/24hr", "c/12hr", "c/8hr", "c/6hr"))

DBhtatx$clase_med <- factor(DBhtatx$clase_med,
 levels = c(0,1,3,6,7,11,13,14),
 labels = c("noMd", "Losartan", "Enalapril", "Amlodipino", "Metoprolol","Hidroclorotiazida","Prazosin","Clonidina"))

ggplot(data= DBhtatx, mapping= aes(x=clase_med, fill = clase_med))+
 geom_bar() +
 labs(title = "Medicamento Prescrito", subtitle = "Discriminado por dosis", x= "Sexo", y = "Numero de casos")+
 facet_wrap(.~DBhtatx$horario_med)+
 scale_fill_manual(values = c("Losartan"= "#FFC300", "Enalapril" = "#DAF7A6","Amlodipino"= "tomato", "Metoprolol"="cyan", "Hidroclorotiazida"="brown", "Prazosin"= "pink", "Clonidina"="green"))+
 theme_dark()+
 theme(axis.text.x = element_text(angle = 45, hjust = 1))


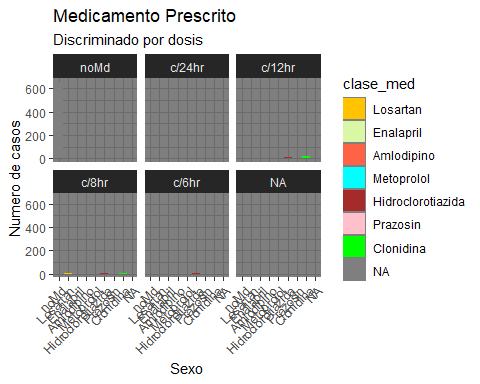


# Visualizar los medicamentos prescritos
library(plotly)

M <- ggplot(data= DBhtatx, mapping= aes(x=clase_med))+
 geom_bar() +
 labs(title = "Medicamentos prescritos", subtitle = "Medicamento", caption = "Grafico 2", x= "Antihipertensivo", y = "Numero de casos")+
 theme(axis.text.x = element_text(angle = 45, hjust = 1))

ggplotly(M)


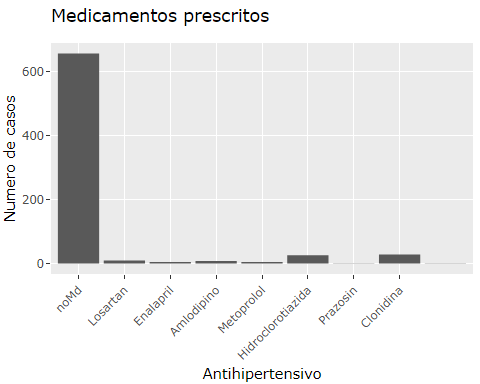


ggplot(data= DBhtatx, mapping= aes(x=fractura, fill = gustillo))+
 geom_bar(width = 0.5) +
 labs(title = "Hueso Fracturado", subtitle = "Discriminado Gustillo-Anderson", x= "Hueso", y = "Numero de fracturas")+
 theme_dark()+ theme(axis.text.x = element_text(angle = 45, hjust = 1))


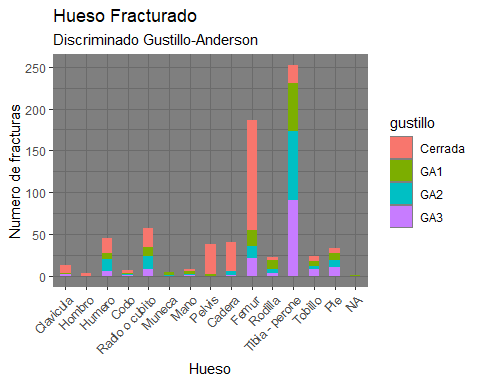


# Codificacion variables de confusion
DBhtatx$ecv <- factor(DBhtatx$ecv,
 levels = c(0,1),
 labels = c("no ECV", "ECV"))

DBhtatx$iam <- factor(DBhtatx$iam,
 levels = c(0,1),
 labels = c("no IAM", "IAM"))

DBhtatx$diabetes <- factor(DBhtatx$diabetes,
 levels = c(0,1),
 labels = c("no Diabetes", "Diabetes"))

DBhtatx$alcohol <- factor(DBhtatx$alcohol,
 levels = c(0,1),
 labels = c("No consumo", "Consumo"))

DBhtatx$ansiedad <- factor(DBhtatx$ansiedad,
 levels = c(0,1),
 labels = c("Ausente", "Presente"))

DBhtatx$constipa <- factor(DBhtatx$constipa,
 levels = c(0,1),
 labels = c("Ausente", "Presente"))
table(DBhtatx$constipa)

##
## Ausente Presente
## 553 186

DBhtatx$insomnio <- factor(DBhtatx$insomnio,
 levels = c(0,1),
 labels = c("Ausente", "Presente"))

DBhtatx$etev <- factor(DBhtatx$etev,
 levels = c(0,1),
 labels = c("Ausente", "Presente"))

DBhtatx$infx <- factor(DBhtatx$infx,
 levels = c(0,1),
 labels = c("Ausente", "Presente"))

DBhtatx$opioid <- factor(DBhtatx$opioid,
 levels = c(0,1),
 labels = c("No prescrito", "Prescrito"))

DBhtatx$aines <- factor(DBhtatx$aines,
 levels = c(0,1),
 labels = c("No prescrito", "Prescrito"))

DBhtatx$obeso <- factor(DBhtatx$obeso,
 levels = c(0,1),
 labels = c("Ausente", "Presente"))


# Visualizaxion general--------------------------------------
head(DBhtatx)

## # A tibble: 6 × 56
## nombre id sexo raza edad ingreso egreso
## <chr> <chr> <fct> <fct> <dbl> <dttm> <dttm>
## 1 Maria cano 2149… Mujer Mest… 68 2023-06-10 00:00:00 2023-06-24 00:00:00
## 2 Luis hernandez 8295… Homb… Mest… 73 2023-05-28 00:00:00 2023-06-16 00:00:00
## 3 Laura estefan… 1152… Mujer Mest… 26 2023-06-01 00:00:00 2023-06-10 00:00:00
## 4 Paula andrea … 1038… Mujer Mest… 19 2023-12-14 00:00:00 2024-01-13 00:00:00
## 5 Alvaro Jose B… 1038… Homb… Mest… 33 2023-12-19 00:00:00 2024-02-24 00:00:00
## 6 Ovidio de jes… 8151… Homb… Mest… 57 2022-07-28 00:00:00 2022-08-01 00:00:00
## # ℹ 49 more variables: estancia <dbl>, muerte <fct>, pas <dbl>, pad <dbl>,
## # hipot <fct>, hipotPAM <dbl>, hipot_lev <fct>, hipot_md <fct>,
## # hipot_vaso <fct>, antihta <fct>, clase_med <fct>, ddd <dbl>,
## # horario_med <fct>, mg_med <dbl>, dias_med <dbl>, ddp <dbl>, freccard <dbl>,
## # frecresp <dbl>, glasgow <dbl>, pasrts <dbl>, cRTS <dbl>, rts <dbl>,
## # fractura <fct>, gustillo <fct>, qxco <dttm>, tiempo_qxco <dbl>, lev <dbl>,
## # aines <fct>, opioid <fct>, infx <fct>, etev <fct>, dolor <dbl>, …

str(DBhtatx)

## tibble [739 × 56] (S3: tbl_df/tbl/data.frame)
## $ nombre : chr [1:739] "Maria cano" "Luis hernandez" "Laura estefania ramirez rendon" "Paula andrea higuita taborda" ...
## $ id : chr [1:739] "21499858" "8295987" "1152462352" "1038926457" ...
## $ sexo : Factor w/ 2 levels "Mujer","Hombre": 1 2 1 1 2 2 2 2 2 1 ...
## $ raza : Factor w/ 2 levels "Mestizo","Negro": 1 1 1 1 1 1 1 1 1 1 ...
## $ edad : num [1:739] 68 73 26 19 33 57 39 57 41 41 ...
## $ ingreso : POSIXct[1:739], format: "2023-06-10" "2023-05-28" ...
## $ egreso : POSIXct[1:739], format: "2023-06-24" "2023-06-16" ...
## $ estancia : num [1:739] 14 19 9 30 5 4 14 15 15 18 ...
## $ muerte : Factor w/ 2 levels "sobrevivio","fallecio": 1 1 1 1 1 1 1 1 1 1 ...
## $ pas : num [1:739] 180 142 146 141 195 157 141 164 155 171 ...
## $ pad : num [1:739] 92 83 117 80 89 94 84 115 100 101 ...
## $ hipot : Factor w/ 2 levels "presente","ausente": 1 1 1 1 1 2 2 2 2 2 ...
## $ hipotPAM : num [1:739] 61 61 57 61 62 89 66 79 80 69 ...
## $ hipot_lev : Factor w/ 2 levels "presente","ausente": 2 2 2 2 2 2 2 2 2 2 ...
## $ hipot_md : Factor w/ 2 levels "presente","ausente": 2 2 2 2 2 2 2 2 2 2 ...
## $ hipot_vaso : Factor w/ 2 levels "presente","ausente": 2 2 2 2 2 2 2 2 2 2 ...
## $ antihta : Factor w/ 2 levels "No prescrito",..: 2 1 1 1 1 1 1 1 1 1 ...
## $ clase_med : Factor w/ 8 levels "noMd","Losartan",..: 4 1 1 1 1 1 1 1 1 1 ...
## $ ddd : num [1:739] 5 1 1 1 1 1 1 1 1 1 ...
## $ horario_med : Factor w/ 5 levels "noMd","c/24hr",..: 3 1 1 1 1 1 1 1 1 1 ...
## $ mg_med : num [1:739] 10 0 0 0 0 0 0 0 0 0 ...
## $ dias_med : num [1:739] 12 0 0 0 0 0 0 0 0 0 ...
## $ ddp : num [1:739] 48 0 0 0 0 0 0 0 0 0 ...
## $ freccard : num [1:739] 89 60 113 110 86 100 98 77 116 144 ...
## $ frecresp : num [1:739] 22.2 15 28.2 27.5 21.5 ...
## $ glasgow : num [1:739] 15 15 15 15 15 15 15 15 15 15 ...
## $ pasrts : num [1:739] 110 90 118 101 124 135 114 143 100 99 ...
## $ cRTS : num [1:739] 3.69 3.69 3.69 3.69 3.69 ...
## $ rts : num [1:739] 120.5 97.4 133.4 120 129.9 ...
## $ fractura : Factor w/ 14 levels "Clavicula","Hombro",..: 12 5 12 9 10 12 12 1 12 12 ...
## $ gustillo : Factor w/ 4 levels "Cerrada","GA1",..: 2 2 2 4 2 2 2 2 2 2 ...
## $ qxco : POSIXct[1:739], format: "2023-06-15" "2023-05-31" ...
## $ tiempo_qxco : num [1:739] 5 3 5 3 4 1 14 13 14 17 ...
## $ lev : num [1:739] 11601 7990 10360 20415 28900 ...
## $ aines : Factor w/ 2 levels "No prescrito",..: 2 2 2 2 2 2 2 2 2 2 ...
## $ opioid : Factor w/ 2 levels "No prescrito",..: 1 2 1 2 2 2 2 2 2 2 ...
## $ infx : Factor w/ 2 levels "Ausente","Presente": 1 2 2 2 2 1 1 1 2 1 ...
## $ etev : Factor w/ 2 levels "Ausente","Presente": 1 1 1 1 1 1 1 1 1 1 ...
## $ dolor : num [1:739] 2 1 2 3 3 2 1 2 3 2 ...
## $ insomnio : Factor w/ 2 levels "Ausente","Presente": 2 1 1 2 2 1 1 2 2 1 ...
## $ constipa : Factor w/ 2 levels "Ausente","Presente": 2 1 1 2 1 1 1 1 2 2 ...
## $ ansiedad : Factor w/ 2 levels "Ausente","Presente": 2 1 1 2 1 1 1 1 2 1 ...
## $ hb : num [1:739] 14.6 9.7 10.7 8.4 13.4 14.5 NA 13.5 13.1 12.3 ...
## $ creat : num [1:739] 0.57 0.78 0.81 0.42 0.86 0.77 NA 0.87 0.44 0.81 ...
## $ peso : num [1:739] 73 69 62 80 66 85 60 75 75 78 ...
## $ fuma : Factor w/ 2 levels "No","Si": 1 1 1 1 1 1 1 1 1 1 ...
## $ alcohol : Factor w/ 2 levels "No consumo","Consumo": 2 1 1 2 1 1 1 1 2 2 ...
## $ obeso : Factor w/ 2 levels "Ausente","Presente": 1 1 2 1 1 1 1 1 1 2 ...
## $ diabetes : Factor w/ 2 levels "no Diabetes",..: 1 2 1 1 1 1 1 1 1 1 ...
## $ iam : Factor w/ 2 levels "no IAM","IAM": 1 1 1 1 1 1 1 1 1 1 ...
## $ ecv : Factor w/ 2 levels "no ECV","ECV": 1 1 1 1 1 1 1 1 1 1 ...
## $ Columna43 : chr [1:739] "0" "0" "0" "0" ...
## $ glasgow_coded : int [1:739] 4 4 4 4 4 4 4 4 4 4 ...
## $ pasrts_coded : int [1:739] 4 4 4 4 4 4 4 4 4 4 ...
## $ frecresp_coded: int [1:739] 2 2 2 2 2 2 2 2 3 4 ...
## $ Hipot : num [1:739] 0 0 0 0 0 1 1 1 1 1 ...

table(DBhtatx$antihta)

##
## No prescrito Prescrito
## 656 83

# Datos duplicados... no hay
duplicated(DBhtatx)

## [1] FALSE FALSE FALSE FALSE FALSE FALSE FALSE FALSE FALSE FALSE FALSE FALSE
## [13] FALSE FALSE FALSE FALSE FALSE FALSE FALSE FALSE FALSE FALSE FALSE FALSE
## [25] FALSE FALSE FALSE FALSE FALSE FALSE FALSE FALSE FALSE FALSE FALSE FALSE
## [37] FALSE FALSE FALSE FALSE FALSE FALSE FALSE FALSE FALSE FALSE FALSE FALSE
## [49] FALSE FALSE FALSE FALSE FALSE FALSE FALSE FALSE FALSE FALSE FALSE FALSE
## [61] FALSE FALSE FALSE FALSE FALSE FALSE FALSE FALSE FALSE FALSE FALSE FALSE
## [73] FALSE FALSE FALSE FALSE FALSE FALSE FALSE FALSE FALSE FALSE FALSE FALSE
## [85] FALSE FALSE FALSE FALSE FALSE FALSE FALSE FALSE FALSE FALSE FALSE FALSE
## [97] FALSE FALSE FALSE FALSE FALSE FALSE FALSE FALSE FALSE FALSE FALSE FALSE
## [109] FALSE FALSE FALSE FALSE FALSE FALSE FALSE FALSE FALSE FALSE FALSE FALSE
## [121] FALSE FALSE FALSE FALSE FALSE FALSE FALSE FALSE FALSE FALSE FALSE FALSE
## [133] FALSE FALSE FALSE FALSE FALSE FALSE FALSE FALSE FALSE FALSE FALSE FALSE
## [145] FALSE FALSE FALSE FALSE FALSE FALSE FALSE FALSE FALSE FALSE FALSE FALSE
## [157] FALSE FALSE FALSE FALSE FALSE FALSE FALSE FALSE FALSE FALSE FALSE FALSE
## [169] FALSE FALSE FALSE FALSE FALSE FALSE FALSE FALSE FALSE FALSE FALSE FALSE
## [181] FALSE FALSE FALSE FALSE FALSE FALSE FALSE FALSE FALSE FALSE FALSE FALSE
## [193] FALSE FALSE FALSE FALSE FALSE FALSE FALSE FALSE FALSE FALSE FALSE FALSE
## [205] FALSE FALSE FALSE FALSE FALSE FALSE FALSE FALSE FALSE FALSE FALSE FALSE
## [217] FALSE FALSE FALSE FALSE FALSE FALSE FALSE FALSE FALSE FALSE FALSE FALSE
## [229] FALSE FALSE FALSE FALSE FALSE FALSE FALSE FALSE FALSE FALSE FALSE FALSE
## [241] FALSE FALSE FALSE FALSE FALSE FALSE FALSE FALSE FALSE FALSE FALSE FALSE
## [253] FALSE FALSE FALSE FALSE FALSE FALSE FALSE FALSE FALSE FALSE FALSE FALSE
## [265] FALSE FALSE FALSE FALSE FALSE FALSE FALSE FALSE FALSE FALSE FALSE FALSE
## [277] FALSE FALSE FALSE FALSE FALSE FALSE FALSE FALSE FALSE FALSE FALSE FALSE
## [289] FALSE FALSE FALSE FALSE FALSE FALSE FALSE FALSE FALSE FALSE FALSE FALSE
## [301] FALSE FALSE FALSE FALSE FALSE FALSE FALSE FALSE FALSE FALSE FALSE FALSE
## [313] FALSE FALSE FALSE FALSE FALSE FALSE FALSE FALSE FALSE FALSE FALSE FALSE
## [325] FALSE FALSE FALSE FALSE FALSE FALSE FALSE FALSE FALSE FALSE FALSE FALSE
## [337] FALSE FALSE FALSE FALSE FALSE FALSE FALSE FALSE FALSE FALSE FALSE FALSE
## [349] FALSE FALSE FALSE FALSE FALSE FALSE FALSE FALSE FALSE FALSE FALSE FALSE
## [361] FALSE FALSE FALSE FALSE FALSE FALSE FALSE FALSE FALSE FALSE FALSE FALSE
## [373] FALSE FALSE FALSE FALSE FALSE FALSE FALSE FALSE FALSE FALSE FALSE FALSE
## [385] FALSE FALSE FALSE FALSE FALSE FALSE FALSE FALSE FALSE FALSE FALSE FALSE
## [397] FALSE FALSE FALSE FALSE FALSE FALSE FALSE FALSE FALSE FALSE FALSE FALSE
## [409] FALSE FALSE FALSE FALSE FALSE FALSE FALSE FALSE FALSE FALSE FALSE FALSE
## [421] FALSE FALSE FALSE FALSE FALSE FALSE FALSE FALSE FALSE FALSE FALSE FALSE
## [433] FALSE FALSE FALSE FALSE FALSE FALSE FALSE FALSE FALSE FALSE FALSE FALSE
## [445] FALSE FALSE FALSE FALSE FALSE FALSE FALSE FALSE FALSE FALSE FALSE FALSE
## [457] FALSE FALSE FALSE FALSE FALSE FALSE FALSE FALSE FALSE FALSE FALSE FALSE
## [469] FALSE FALSE FALSE FALSE FALSE FALSE FALSE FALSE FALSE FALSE FALSE FALSE
## [481] FALSE FALSE FALSE FALSE FALSE FALSE FALSE FALSE FALSE FALSE FALSE FALSE
## [493] FALSE FALSE FALSE FALSE FALSE FALSE FALSE FALSE FALSE FALSE FALSE FALSE
## [505] FALSE FALSE FALSE FALSE FALSE FALSE FALSE FALSE FALSE FALSE FALSE FALSE
## [517] FALSE FALSE FALSE FALSE FALSE FALSE FALSE FALSE FALSE FALSE FALSE FALSE
## [529] FALSE FALSE FALSE FALSE FALSE FALSE FALSE FALSE FALSE FALSE FALSE FALSE
## [541] FALSE FALSE FALSE FALSE FALSE FALSE FALSE FALSE FALSE FALSE FALSE FALSE
## [553] FALSE FALSE FALSE FALSE FALSE FALSE FALSE FALSE FALSE FALSE FALSE FALSE
## [565] FALSE FALSE FALSE FALSE FALSE FALSE FALSE FALSE FALSE FALSE FALSE FALSE
## [577] FALSE FALSE FALSE FALSE FALSE FALSE FALSE FALSE FALSE FALSE FALSE FALSE
## [589] FALSE FALSE FALSE FALSE FALSE FALSE FALSE FALSE FALSE FALSE FALSE FALSE
## [601] FALSE FALSE FALSE FALSE FALSE FALSE FALSE FALSE FALSE FALSE FALSE FALSE
## [613] FALSE FALSE FALSE FALSE FALSE FALSE FALSE FALSE FALSE FALSE FALSE FALSE
## [625] FALSE FALSE FALSE FALSE FALSE FALSE FALSE FALSE FALSE FALSE FALSE FALSE
## [637] FALSE FALSE FALSE FALSE FALSE FALSE FALSE FALSE FALSE FALSE FALSE FALSE
## [649] FALSE FALSE FALSE FALSE FALSE FALSE FALSE FALSE FALSE FALSE FALSE FALSE
## [661] FALSE FALSE FALSE FALSE FALSE FALSE FALSE FALSE FALSE FALSE FALSE FALSE
## [673] FALSE FALSE FALSE FALSE FALSE FALSE FALSE FALSE FALSE FALSE FALSE FALSE
## [685] FALSE FALSE FALSE FALSE FALSE FALSE FALSE FALSE FALSE FALSE FALSE FALSE
## [697] FALSE FALSE FALSE FALSE FALSE FALSE FALSE FALSE FALSE FALSE FALSE FALSE
## [709] FALSE FALSE FALSE FALSE FALSE FALSE FALSE FALSE FALSE FALSE FALSE FALSE
## [721] FALSE FALSE FALSE FALSE FALSE FALSE FALSE FALSE FALSE FALSE FALSE FALSE
## [733] FALSE FALSE FALSE FALSE FALSE FALSE FALSE

sum(duplicated(DBhtatx))

## [1] 0

which(duplicated(DBhtatx))

## integer(0)

print(which(duplicated(DBhtatx)))

## integer(0)

# Descriptivos generales
library(medicaldata)
library(summarytools)
library(psych)

##
## Attaching package: 'psych'

## The following objects are masked from 'package:ggplot2':
##
## %+%, alpha

library(pastecs)

##
## Attaching package: 'pastecs'

## The following objects are masked from 'package:dplyr':
##
## first, last

## The following object is masked from 'package:tidyr':
##
## extract

# describe(DBhtatx)
#
# summary(DBhtatx)
#
# describe.by(DBhtatx,DBhtatx$antihta)
#
# stat.desc(DBhtatx)

par(mfrow=c(1,2))
plot(density(DBhtatx$pas,na.rm = T),col=2,main="PAS")
plot(density(DBhtatx$pad, na.rm = T),col=2,main="PAD")


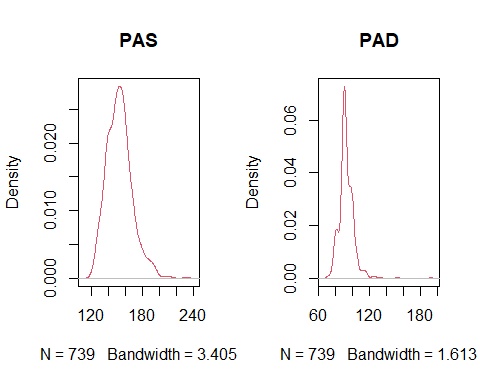


plot(density(DBhtatx$hipotPAM, na.rm = T),col=2,main="PAM")
plot(density(DBhtatx$pasrts, na.rm = T),col=2,main="PAStrauma")


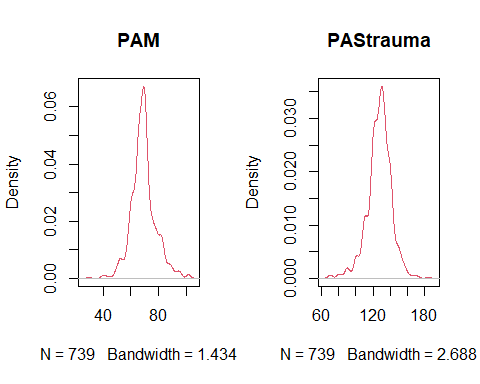


table(DBhtatx$hipot_lev)

##
## presente ausente
## 99 640

table(DBhtatx$hipot_md)

##
## presente ausente
## 63 676

table(DBhtatx$hipot_vaso)

##
## presente ausente
## 34 705

hist(DBhtatx$hipotPAM)

ggplot(DBhtatx, aes(x = hipotPAM, fill = antihta, color = antihta)) +
 geom_density(alpha = 0.8)


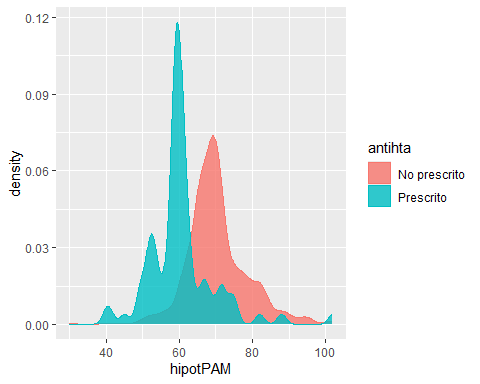


# MODELOS

# MODELOS---------------------------------------------
# Lineal 60 para estancia hospitalaria menos de 60 dias-------------------

colnames(DBhtatx
)

## [1] "nombre" "id" "sexo" "raza"
## [5] "edad" "ingreso" "egreso" "estancia"
## [9] "muerte" "pas" "pad" "hipot"
## [13] "hipotPAM" "hipot_lev" "hipot_md" "hipot_vaso"
## [17] "antihta" "clase_med" "ddd" "horario_med"
## [21] "mg_med" "dias_med" "ddp" "freccard"
## [25] "frecresp" "glasgow" "pasrts" "cRTS"
## [29] "rts" "fractura" "gustillo" "qxco"
## [33] "tiempo_qxco" "lev" "aines" "opioid"
## [37] "infx" "etev" "dolor" "insomnio"
## [41] "constipa" "ansiedad" "hb" "creat"
## [45] "peso" "fuma" "alcohol" "obeso"
## [49] "diabetes" "iam" "ecv" "Columna43"
## [53] "glasgow_coded" "pasrts_coded" "frecresp_coded" "Hipot"

quantile(DBhtatx$creat, na.rm=T)

## 0% 25% 50% 75% 100%
## 0.14 0.64 0.82 1.01 9.70

max(DBhtatx$creat)

## [1] NA

table(DBhtatx$obeso
)

##
## Ausente Presente
## 693 46

# Evaluar la variable antecedente de obesidad respecto al peso inicial reportado en la hospitalizacion
DBhtatx |> group_by(obeso) |> summarise(mean(peso))

## # A tibble: 2 × 2
## obeso `mean(peso)`
## <fct> <dbl>
## 1 Ausente 67.6
## 2 Presente 91.7

# Antihipertensivos
table(DBhtatx$antihta)

##
## No prescrito Prescrito
## 656 83

hist(DBhtatx$dias_med)


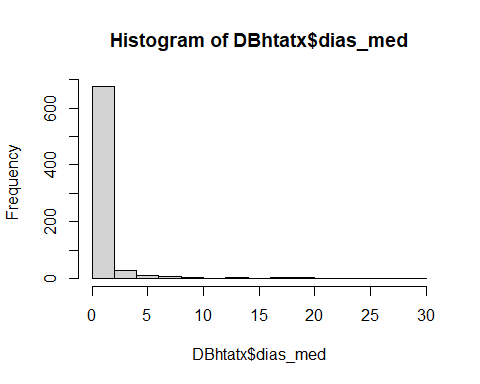


#DBhtatx$Antihipertensivos <- ifelse(
# DBhtatx$Antihipertensivos == "muerte intrahospitalaria", 1,0)

#table(DBhtatx$Muerte)

# DBhtatx_60 <- DBhtatx %>%
# filter(DBhtatx$estancia<60)

# Nueva variable permite clasificar estancia menor o mayor a 60 dias
DBhtatx <- DBhtatx %>%
 mutate(
 estancia60 = estancia <60)

# Verificamos cuantos son menores a 60 dias (solo 16 hay por encima de ese tiempo)
table(DBhtatx$estancia60)

##
## FALSE TRUE
## 16 723

# El valor de la variable es el de la estancia original y no se consideraran los outlier
DBhtatx <- DBhtatx %>%
 mutate(estancianew = ifelse(DBhtatx$estancia60==TRUE,estancia,NA))

summary(DBhtatx$estancianew)

## Min. 1st Qu. Median Mean 3rd Qu. Max. NA's
## 1.00 6.00 9.00 14.16 17.00 59.00 16

# Modelo lineal de estancia hospitalaria incluyendo las variables de confusion del DAG (opcion Poisson)
modelo2_60 <- lm (DBhtatx$estancianew ~ antihta + tiempo_qxco +
 edad + raza + peso + fuma + alcohol + obeso + diabetes + iam + ecv +
 dolor + insomnio + constipa + ansiedad + lev +
 cRTS + infx + hb
 , data = DBhtatx)

# EL coeficiente cambia de 3.6 a 1.57

# Estructura general intercepto y coeficientes
modelo2_60

##
## Call:
## lm(formula = DBhtatx$estancianew ~ antihta + tiempo_qxco + edad +
## raza + peso + fuma + alcohol + obeso + diabetes + iam + ecv +
## dolor + insomnio + constipa + ansiedad + lev + cRTS + infx +
## hb, data = DBhtatx)
##
## Coefficients:
## (Intercept) antihtaPrescrito tiempo_qxco edad
## 2.103078 1.527050 0.034229 0.014865
## razaNegro peso fumaSi alcoholConsumo
## -1.271970 -0.014308 -0.599092 -0.481691
## obesoPresente diabetesDiabetes iamIAM ecvECV
## -0.255442 1.003130 -1.174491 3.708314
## dolor insomnioPresente constipaPresente ansiedadPresente
## 0.010612 -0.214859 NA 1.074837
## lev cRTS infxPresente hb
## 0.001217 0.026080 2.139031 -0.181228

hist(DBhtatx$ddd)


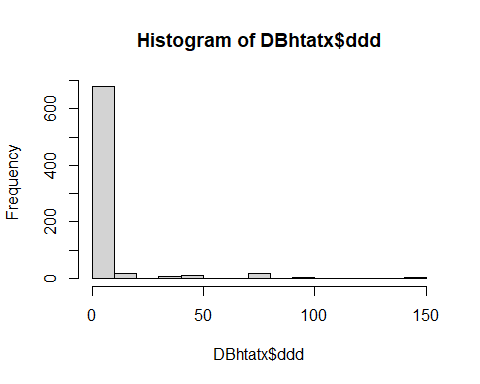


hist(DBhtatx$ddp)


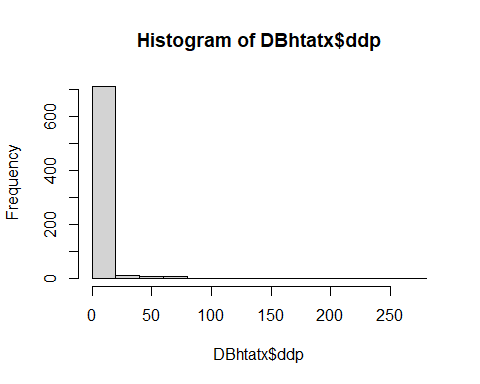


table(DBhtatx$horario_med) # hay problema

##
## noMd c/24hr c/12hr c/8hr c/6hr
## 655 26 41 15 1

hist(DBhtatx$dias_med)


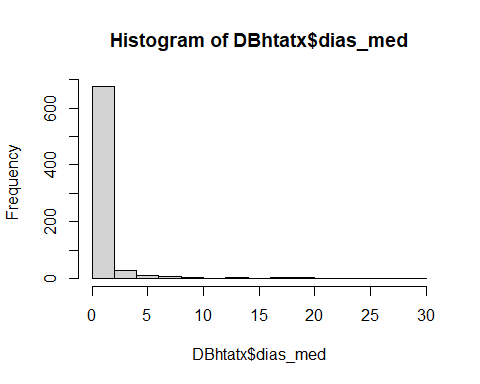


# + pad + pas + edad + + clase_med + ddd + horario_med + mg_med +
# dias_med + ddp + freccard + glasgow + pasrts + rts + fractura +
# gustillo + qxco + lev + aines + opioid + infx + etev +
# dolor + insomnio + constipa + ansiedad + hb + creat + peso +
# fuma + alcohol + obeso + diabetes + iam + ecv , data = DBhtatx)


hist(DBhtatx$ddp)


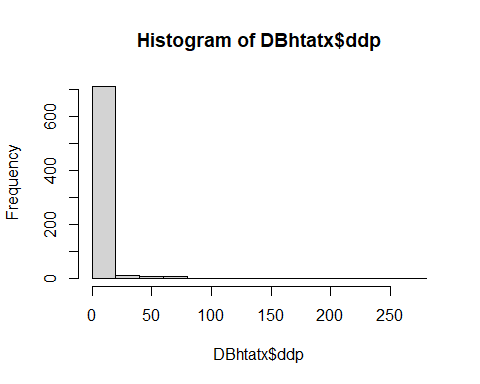


summary(DBhtatx$ddp)

## Min. 1st Qu. Median Mean 3rd Qu. Max. NA's
## 0.000 0.000 0.000 2.915 0.000 261.000 2

DBhtatx$DDP

## NULL

DBhtatx <- DBhtatx %>%
 mutate(DDP= ifelse(DBhtatx$ddp==0,NA,DBhtatx$ddp))

hist(DBhtatx$DDP)


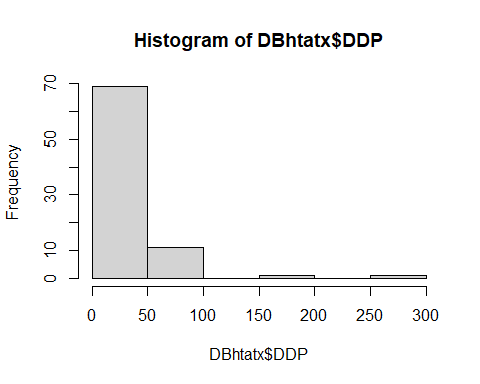


summary(DBhtatx$DDP)

## Min. 1st Qu. Median Mean 3rd Qu. Max. NA's
## 0.02 7.25 13.00 26.20 35.00 261.00 657

# Modelo lineal con ddp---------

modelo2a <- lm (DBhtatx$estancia ~ ddp + tiempo_qxco +
 edad + raza + peso + fuma + alcohol + obeso + diabetes + iam + ecv +
 dolor + insomnio + constipa + ansiedad + lev +
 rts + infx + hb
 , data = DBhtatx)

modelo2a

##
## Call:
## lm(formula = DBhtatx$estancia ~ ddp + tiempo_qxco + edad + raza +
## peso + fuma + alcohol + obeso + diabetes + iam + ecv + dolor +
## insomnio + constipa + ansiedad + lev + rts + infx + hb, data = DBhtatx)
##
## Coefficients:
## (Intercept) ddp tiempo_qxco edad
## 9.107772 0.044126 0.028572 0.003317
## razaNegro peso fumaSi alcoholConsumo
## -2.520272 -0.074973 -0.555987 0.106162
## obesoPresente diabetesDiabetes iamIAM ecvECV
## 3.329023 2.890011 -0.820684 0.859082
## dolor insomnioPresente constipaPresente ansiedadPresente
## 0.018118 -0.396351 NA 1.966280
## lev rts infxPresente hb
## 0.001324 -0.022852 3.515382 -0.235960

# Modelo lineal original con original completo incluyendo los 16 outlier---------
modelo2 <- lm (DBhtatx$estancia ~ antihta + tiempo_qxco +
 edad + raza + peso + fuma + obeso + diabetes + iam + ecv +
 dolor + insomnio + constipa + ansiedad + lev +
 rts + infx + hb
 , data = DBhtatx)

# Residuiales, qq plot, residuales std, leverage######## Graficamente
plot(modelo2)


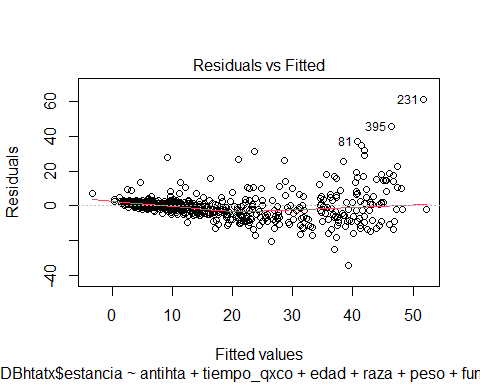

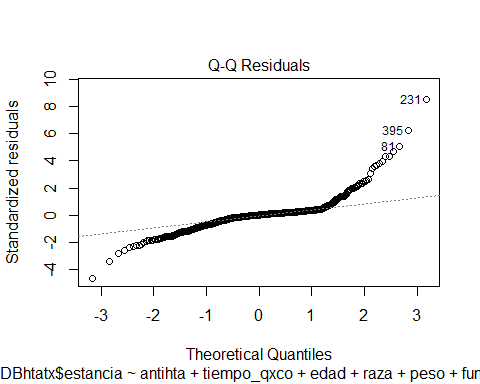

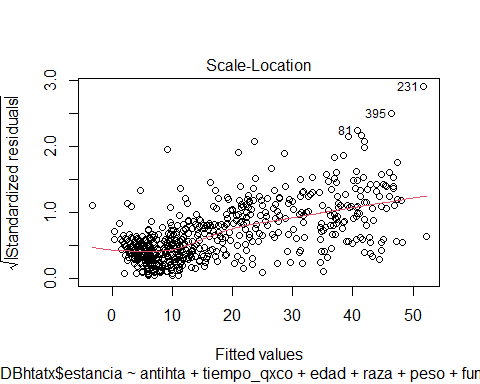

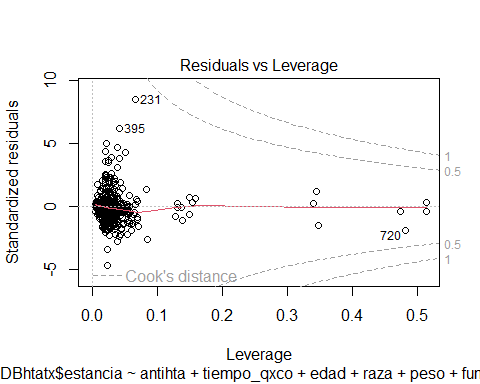


covratio(modelo2)

## 1 2 3 4 5 6 8 9
## 1.0633412 1.1024448 1.0317599 1.0595615 0.5542631 1.0392052 1.0437693 1.0653219
## 10 11 12 13 14 15 16 17
## 1.0338234 1.0154789 0.6986502 1.0507700 1.0227312 0.5483065 0.7510703 1.0420911
## 18 19 20 21 22 23 24 25
## 0.9921336 0.8753664 0.9072388 0.9495518 1.0220515 0.9164447 1.0344098 1.1596725
## 26 27 28 29 30 31 32 33
## 1.0373880 1.0459892 1.0819905 1.0199072 1.0083430 1.0518329 0.9272170 0.6224531
## 34 35 36 37 38 39 40 41
## 1.0364985 0.8967592 1.0438081 1.0396917 0.9458362 1.0470364 0.9963398 0.7082399
## 42 43 44 45 46 47 48 49
## 1.0574546 1.0803131 1.0690609 1.0391714 1.0131447 1.0341495 1.0833360 1.0535587
## 50 51 52 53 55 56 57 58
## 1.0072192 1.0241082 1.0248407 1.0420336 1.0399079 1.0389765 1.0386220 1.0391165
## 59 60 61 62 63 64 65 66
## 1.0524029 1.0230200 0.9374126 1.1853983 1.0124773 1.0438073 1.0297056 1.0043696
## 67 68 69 71 72 73 74 75
## 1.0348159 0.9315182 1.0392211 1.0262290 1.0369436 1.2138503 1.0950827 1.0544859
## 76 77 78 79 80 81 82 84
## 1.0592118 1.0594983 1.0014140 1.0734786 1.0325354 0.4914060 1.0145662 0.9539036
## 85 86 87 89 90 91 92 93
## 1.0509160 1.0447184 1.0431820 1.0403598 1.0407292 1.0399450 1.0413654 0.9921111
## 94 95 96 97 98 99 100 101
## 1.0709023 1.0524446 1.0779447 1.0556245 1.0655264 1.0522590 0.9862867 1.0361960
## 102 103 104 105 106 107 108 109
## 1.0407092 2.1069874 1.0385735 1.0581359 0.9314438 1.0446883 1.0354003 1.0237904
## 110 111 112 113 114 115 116 117
## 1.0425215 1.0465470 1.0108858 1.0461061 0.9768355 1.0644329 0.9950040 1.0573861
## 118 119 120 121 122 123 124 126
## 1.0099245 0.9215582 1.0371037 0.9861016 1.0707895 1.0662209 1.0433092 1.0542215
## 127 128 129 131 132 133 134 135
## 1.0433866 1.0475827 1.0458408 1.0515226 1.0546842 1.5031959 0.9815320 1.0550994
## 136 137 138 139 140 141 143 144
## 1.0382050 1.0400400 1.0375806 0.8647563 1.0442274 1.0443616 1.0234274 1.0398493
## 146 147 148 149 150 151 152 154
## 1.0559939 1.0496034 1.0445941 0.9836487 1.0710365 1.0153773 1.0304609 1.0448298
## 155 156 157 158 159 160 161 162
## 1.0445149 1.1520745 0.9757498 1.0440966 1.0405998 1.0454794 1.0451364 1.0452888
## 163 164 165 166 167 168 169 170
## 1.0414965 1.0284408 1.2072304 1.0499715 0.9904738 1.0421456 1.0611342 1.0516735
## 171 172 173 174 175 176 177 179
## 0.9457136 0.9616262 0.9901957 1.0426526 1.1926550 0.8091429 1.0420835 1.0754681
## 180 181 182 183 184 185 186 187
## 1.0354038 1.0392684 1.0479147 1.0484391 0.9201961 0.9397625 1.1839871 1.0435920
## 188 189 190 191 193 194 195 197
## 1.0217758 1.0396212 1.0540667 1.0572832 1.0609516 1.0109831 1.0201139 1.0071493
## 198 199 200 201 202 203 204 205
## 1.1929361 1.0459912 0.9442195 1.0688522 0.9606572 1.0395995 1.0387826 1.0425065
## 206 207 208 209 210 211 212 213
## 1.0353714 1.0384105 1.0351104 1.0355646 1.0376637 1.0441746 0.9925540 0.6675658
## 214 215 216 217 218 219 220 221
## 1.0556212 1.0560888 1.0362096 1.0639151 0.9986194 1.0712145 0.8990317 1.0332886
## 222 223 224 225 226 227 228 229
## 1.0681592 1.0430885 1.0479699 1.0468131 1.0498428 1.0455846 1.0435687 1.0543980
## 230 231 232 233 234 235 236 237
## 0.9039068 0.1155766 1.0537755 1.0342133 1.0382551 1.0159600 1.0455420 1.0589171
## 238 239 240 241 242 243 244 245
## 1.0491474 1.0398463 1.0828443 0.9700302 1.0626104 1.0610460 1.0501651 1.0272266
## 246 247 248 249 250 251 252 253
## 1.0180937 1.0687369 1.0666104 1.0538162 1.0737115 1.0817498 1.0333623 1.0488114
## 254 255 256 257 258 259 260 261
## 1.0437643 1.0445583 1.0419560 1.0897526 1.0556227 0.9974836 1.0421135 1.0378586
## 262 263 264 265 266 267 268 269
## 1.0445636 1.0414375 1.0842409 1.0409621 1.0115053 1.0448669 1.0562867 1.0681365
## 270 271 272 273 274 275 276 277
## 0.9611497 1.0551957 1.0684849 0.8787169 1.0339839 1.0589847 0.8649557 1.0483338
## 278 279 280 281 282 283 284 285
## 1.0618599 1.0427914 1.0481321 1.0455690 1.0273870 0.9839221 0.9563366 1.0442659
## 286 287 288 289 290 291 292 293
## 1.0644769 1.0582096 1.0953382 1.0483783 1.0528877 1.0436749 1.0520465 1.0893871
## 294 295 296 297 298 299 300 301
## 1.0842310 1.0130240 1.0442495 1.0600058 1.0598359 1.0522798 1.0446605 1.0781188
## 302 303 304 305 306 307 308 310
## 1.0433798 1.0414541 1.0356190 1.0610324 1.0731430 1.0524978 1.0545900 1.0523869
## 311 312 313 314 315 316 318 319
## 1.0565683 1.0584778 1.0193381 1.0478665 1.0377610 2.1069874 0.9513421 1.0393092
## 320 321 322 325 326 327 328 329
## 1.0299701 1.0592812 1.0703778 1.0445910 1.0529603 0.9920424 1.0560334 1.0390065
## 330 332 333 334 335 336 337 338
## 1.0331092 1.5585828 1.0630523 1.0320593 1.0453929 1.0376906 1.0386184 1.0479357
## 339 340 341 342 343 344 345 346
## 1.0418742 1.0445216 1.0511072 1.0396510 1.0656765 1.0403641 1.0429547 1.0381477
## 347 349 350 351 352 353 354 355
## 1.1974959 1.0535072 1.0495766 1.0395059 1.0348482 1.0430799 1.0421933 1.0504283
## 356 357 358 359 360 361 362 363
## 1.0361153 0.9399998 1.0634771 1.0888488 0.7383977 1.0405956 1.0584907 1.0470704
## 364 365 366 367 368 369 370 371
## 1.0416444 1.0381148 0.7189978 1.0237104 1.0235766 1.0446347 1.0423260 1.0387448
## 372 373 374 375 376 377 378 380
## 0.9793476 1.0445427 1.0393972 1.0383142 1.0526154 1.0439144 1.0375807 1.0390387
## 381 382 383 384 385 386 387 388
## 1.0522151 1.0400367 1.0386341 1.0161474 1.0551242 1.0370063 1.0547297 1.0495795
## 389 390 391 392 393 394 395 396
## 1.0483374 1.0252061 1.0445623 1.0392811 1.0499662 1.0585281 0.3287904 1.0402246
## 397 398 399 400 401 402 403 404
## 1.0541091 1.0445447 1.0378622 1.0793829 1.0381943 1.0370880 1.0584983 1.0748666
## 405 406 407 408 409 410 411 412
## 1.0534287 1.0615654 1.0512465 1.0683194 1.1020294 1.0455226 1.0577402 1.0521485
## 413 414 415 417 418 419 420 421
## 1.0782556 1.0426824 1.0395815 1.0494157 1.0383873 1.0550618 1.0469126 1.0492141
## 422 423 424 425 427 428 429 430
## 1.0472501 1.0378156 1.0447914 1.0451389 1.0583702 1.0468045 1.0437027 1.0315798
## 431 432 433 435 437 439 441 442
## 1.0477980 1.0429709 1.0254695 0.9125730 1.0415758 1.0351700 1.0362619 1.0389568
## 443 444 445 446 447 448 449 450
## 0.9564135 1.4827642 0.8500959 1.0376909 1.0598840 1.0195880 1.0388586 1.0400654
## 451 452 454 455 456 457 458 459
## 1.0477731 1.0479694 1.0655172 1.0462892 1.0440492 1.0455612 1.0394166 1.0375363
## 460 462 463 464 465 466 467 468
## 1.0483455 1.0329563 1.0421663 1.0661090 1.0368409 1.0372462 1.0348103 1.0504228
## 469 470 471 472 473 474 475 476
## 1.0329566 1.0388343 1.0515547 1.0357107 1.0796245 1.0418921 1.0351529 1.0613182
## 477 478 479 480 481 482 483 484
## 1.0180513 1.0231346 1.0289424 1.0391521 1.0545470 1.0424457 1.0399625 1.0564871
## 485 486 487 488 489 490 491 492
## 1.0355353 1.0616666 1.0399114 1.0361584 1.0374012 1.0365756 1.0454599 1.0930784
## 493 494 496 497 499 500 501 502
## 1.0456098 1.0747021 1.0371651 1.0523810 0.9896792 1.0053883 1.0320193 1.0394872
## 504 506 507 509 512 513 514 515
## 1.0462047 1.0437608 1.0446907 1.0377408 1.0292690 1.0496828 1.0613054 1.0451599
## 516 517 518 519 522 523 524 525
## 1.0429116 1.0276250 1.0377285 1.0426432 1.0451717 1.0633665 1.0375210 1.0389842
## 526 527 528 529 530 531 532 533
## 0.5982037 1.0486443 1.0475035 1.0594060 1.0600634 1.0486085 1.0590480 1.0437725
## 534 535 536 538 539 543 545 546
## 1.0370572 1.0484022 1.0404669 1.0374144 1.0515094 1.0527626 1.0463038 1.0343414
## 547 548 549 550 551 552 553 554
## 1.0463151 1.0310958 1.0442231 1.0501683 0.9892174 1.0410073 1.0379079 1.0566319
## 555 556 557 558 559 560 561 563
## 1.0367257 1.0382715 1.0503203 1.0520923 1.0500613 1.0429975 1.0878891 0.9267469
## 564 565 566 568 569 570 572 573
## 1.0438513 1.0381349 1.0498860 1.0355950 1.0460411 1.0427396 1.0375277 1.0517509
## 574 575 576 577 578 579 580 581
## 1.0248802 1.0569103 1.0370534 1.0463778 1.0403828 1.0487084 1.0566811 1.0484989
## 582 584 585 586 587 588 590 591
## 1.0595038 1.0268887 1.0421025 1.0378029 1.0436543 1.0597942 1.0608912 1.0353256
## 592 594 595 596 597 598 599 600
## 1.0404821 1.0422077 1.0705939 1.0600382 1.0417581 1.0419627 1.0367655 1.0506375
## 601 602 603 605 606 607 608 609
## 1.0156340 1.0590604 0.9647798 1.0473175 1.0418799 1.0313152 1.0432840 1.0397666
## 610 611 612 614 615 616 617 618
## 1.0367555 1.0459684 1.0378562 1.0357408 1.0353408 1.0316097 1.0490931 1.0146254
## 619 620 621 622 623 624 625 627
## 1.0437429 1.0371507 1.0387177 0.9950709 0.9779903 1.0383575 1.0368022 1.0441463
## 629 630 631 632 633 634 635 636
## 1.0436878 1.0467201 1.0472091 1.0014214 1.0394164 1.0437599 1.0454514 1.0447764
## 637 638 639 640 641 642 646 647
## 1.0408165 1.0375067 1.0410836 1.0388952 1.0394021 1.0351280 1.0799619 1.0319351
## 648 649 650 653 654 656 657 658
## 1.0128708 1.0476908 1.0408173 1.0392012 1.0451982 1.0483802 1.0362664 1.0368691
## 659 660 661 663 664 665 666 667
## 0.9692458 1.0880788 1.0560880 1.0384907 1.0395662 1.0388674 1.0383501 1.0713865
## 669 670 671 672 673 676 677 678
## 1.0828030 1.0443014 1.0609967 1.0547607 1.0486418 1.0528668 1.0404448 1.0572500
## 679 680 681 682 683 684 685 686
## 1.0358393 1.0426066 1.0403114 1.0369359 1.0442043 1.0492965 1.0463368 1.0359023
## 687 688 689 690 691 692 693 694
## 1.0576040 1.0395313 1.0451582 1.0568483 1.0397459 1.0759188 1.0397079 1.0404958
## 695 696 698 699 700 701 702 703
## 1.0397823 1.0413782 1.0394605 1.0522817 1.0517824 1.0111749 1.0294586 1.0621916
## 704 705 706 707 708 709 710 711
## 1.0418348 1.0870254 0.9566126 1.0484448 1.0359807 1.0549713 1.0455368 1.0399456
## 712 713 714 715 716 717 718 719
## 1.0410681 1.0506438 1.0501417 1.9463688 1.0777192 1.0192619 1.0238460 1.0518789
## 720 721 722 723 724 725 726
## 1.7802727 0.9572410 1.0772406 1.0402602 1.0566411 1.0428230 1.0347629

# coeficientes, IC95% y valor p a presentar
tbl_regression(modelo2, label = list(antihta~"Antihipertensivos",tiempo_qxco~"Tiempo a cirugía (dias)",
 raza~"Raza", edad~"Edad (años)",peso ~"Peso (Kg)", fuma~"Fumador cigarrillo tabaco o vaper", obeso~ "Antecedente Obesidad",diabetes~"Diabetes Mellitus tipo 1 o 2",
 iam~"Infarto del miocardio",ecv~ "Enfermedad cerebrovascular",dolor~"Dolor por EVA", insomnio~"Insomnio",
 constipa~"Constipación", ansiedad~"Ansiedad", hb~"Hemoglobina (gr/dL)", infx ~"Infeccion clinica",
 lev~"Líquidos endovenosos (mL)", rts~"Puntaje de Trauma (RTS)"))

## Table printed with {flextable}, not {gt}. Learn why at
## https://www.danieldsjoberg.com/gtsummary/articles/rmarkdown.html
## To suppress this message, include `message = FALSE` in the code chunk header.

| **Characteristic** | **Beta** | **95% CI**^1^ | **p-value** |
| --- | --- | --- | --- |
| Antihipertensivos |  |  |  |
| No prescrito | — | — |  |
| Prescrito | 3.7 | 1.7, 5.7 | <0.001 |
| Tiempo a cirugía (dias) | 0.03 | 0.00, 0.06 | 0.039 |
| Edad (años) | 0.00 | -0.03, 0.03 | >0.9 |
| Raza |  |  |  |
| Mestizo | — | — |  |
| Negro | -2.4 | -7.7, 2.9 | 0.4 |
| Peso (Kg) | -0.08 | -0.15, 0.00 | 0.037 |
| Fumador cigarrillo tabaco o vaper |  |  |  |
| No | — | — |  |
| Si | -0.48 | -1.7, 0.71 | 0.4 |
| Antecedente Obesidad |  |  |  |
| Ausente | — | — |  |
| Presente | 3.1 | 0.21, 6.0 | 0.035 |
| Diabetes Mellitus tipo 1 o 2 |  |  |  |
| no Diabetes | — | — |  |
| Diabetes | 2.5 | -0.30, 5.4 | 0.079 |
| Infarto del miocardio |  |  |  |
| no IAM | — | — |  |
| IAM | -1.5 | -10, 7.1 | 0.7 |
| Enfermedad cerebrovascular |  |  |  |
| no ECV | — | — |  |
| ECV | 1.7 | -8.9, 12 | 0.7 |
| Dolor por EVA | 0.01 | -1.1, 1.1 | >0.9 |
| Insomnio |  |  |  |
| Ausente | — | — |  |
| Presente | -0.33 | -1.8, 1.2 | 0.7 |
| Constipación |  |  |  |
| Ausente | — | — |  |
| Presente | -0.09 | -1.7, 1.5 | >0.9 |
| Ansiedad |  |  |  |
| Ausente | — | — |  |
| Presente | 1.4 | -0.41, 3.1 | 0.13 |
| Líquidos endovenosos (mL) | 0.00 | 0.00, 0.00 | <0.001 |
| Puntaje de Trauma (RTS) | -0.02 | -0.07, 0.03 | 0.4 |
| Infeccion clinica |  |  |  |
| Ausente | — | — |  |
| Presente | 3.4 | 1.7, 5.2 | <0.001 |
| Hemoglobina (gr/dL) | -0.22 | -0.49, 0.05 | 0.11 |
| ^1^CI = Confidence Interval | | | |

# Interceptos, medidas de ajuste (RSE, df, R2 y F stat)

summary.lm(modelo2)

##
## Call:
## lm(formula = DBhtatx$estancia ~ antihta + tiempo_qxco + edad +
## raza + peso + fuma + obeso + diabetes + iam + ecv + dolor +
## insomnio + constipa + ansiedad + lev + rts + infx + hb, data = DBhtatx)
##
## Residuals:
## Min 1Q Median 3Q Max
## -34.210 -2.616 0.195 1.753 61.322
##
## Coefficients:
## Estimate Std. Error t value Pr(>|t|)
## (Intercept) 9.073e+00 4.627e+00 1.961 0.050297 .
## antihtaPrescrito 3.689e+00 1.029e+00 3.585 0.000363 ***
## tiempo_qxco 2.959e-02 1.430e-02 2.070 0.038880 *
## edad 1.238e-03 1.698e-02 0.073 0.941906
## razaNegro -2.402e+00 2.686e+00 -0.894 0.371559
## peso -7.863e-02 3.759e-02 -2.092 0.036858 *
## fumaSi -4.842e-01 6.061e-01 -0.799 0.424665
## obesoPresente 3.093e+00 1.466e+00 2.109 0.035324 *
## diabetesDiabetes 2.548e+00 1.450e+00 1.757 0.079325 .
## iamIAM -1.476e+00 4.374e+00 -0.338 0.735849
## ecvECV 1.726e+00 5.397e+00 0.320 0.749213
## dolor 6.289e-03 5.631e-01 0.011 0.991094
## insomnioPresente -3.292e-01 7.571e-01 -0.435 0.663818
## constipaPresente -9.342e-02 8.177e-01 -0.114 0.909084
## ansiedadPresente 1.354e+00 8.991e-01 1.506 0.132478
## lev 1.304e-03 5.571e-05 23.400 < 2e-16 ***
## rts -2.027e-02 2.468e-02 -0.821 0.411832
## infxPresente 3.424e+00 8.916e-01 3.841 0.000135 ***
## hb -2.231e-01 1.379e-01 -1.618 0.106245
## ---
## Signif. codes: 0 '***' 0.001 '**' 0.01 '*' 0.05 '.' 0.1 ' ' 1
##
## Residual standard error: 7.474 on 644 degrees of freedom
## (76 observations deleted due to missingness)
## Multiple R-squared: 0.7594, Adjusted R-squared: 0.7527
## F-statistic: 112.9 on 18 and 644 DF, p-value: < 2.2e-16

print(summary.lm(modelo2))

##
## Call:
## lm(formula = DBhtatx$estancia ~ antihta + tiempo_qxco + edad +
## raza + peso + fuma + obeso + diabetes + iam + ecv + dolor +
## insomnio + constipa + ansiedad + lev + rts + infx + hb, data = DBhtatx)
##
## Residuals:
## Min 1Q Median 3Q Max
## -34.210 -2.616 0.195 1.753 61.322
##
## Coefficients:
## Estimate Std. Error t value Pr(>|t|)
## (Intercept) 9.073e+00 4.627e+00 1.961 0.050297 .
## antihtaPrescrito 3.689e+00 1.029e+00 3.585 0.000363 ***
## tiempo_qxco 2.959e-02 1.430e-02 2.070 0.038880 *
## edad 1.238e-03 1.698e-02 0.073 0.941906
## razaNegro -2.402e+00 2.686e+00 -0.894 0.371559
## peso -7.863e-02 3.759e-02 -2.092 0.036858 *
## fumaSi -4.842e-01 6.061e-01 -0.799 0.424665
## obesoPresente 3.093e+00 1.466e+00 2.109 0.035324 *
## diabetesDiabetes 2.548e+00 1.450e+00 1.757 0.079325 .
## iamIAM -1.476e+00 4.374e+00 -0.338 0.735849
## ecvECV 1.726e+00 5.397e+00 0.320 0.749213
## dolor 6.289e-03 5.631e-01 0.011 0.991094
## insomnioPresente -3.292e-01 7.571e-01 -0.435 0.663818
## constipaPresente -9.342e-02 8.177e-01 -0.114 0.909084
## ansiedadPresente 1.354e+00 8.991e-01 1.506 0.132478
## lev 1.304e-03 5.571e-05 23.400 < 2e-16 ***
## rts -2.027e-02 2.468e-02 -0.821 0.411832
## infxPresente 3.424e+00 8.916e-01 3.841 0.000135 ***
## hb -2.231e-01 1.379e-01 -1.618 0.106245
## ---
## Signif. codes: 0 '***' 0.001 '**' 0.01 '*' 0.05 '.' 0.1 ' ' 1
##
## Residual standard error: 7.474 on 644 degrees of freedom
## (76 observations deleted due to missingness)
## Multiple R-squared: 0.7594, Adjusted R-squared: 0.7527
## F-statistic: 112.9 on 18 and 644 DF, p-value: < 2.2e-16

summary(DBhtatx$estancia)

## Min. 1st Qu. Median Mean 3rd Qu. Max.
## 1.00 6.00 10.00 15.38 18.00 113.00

hist(DBhtatx$estancia)


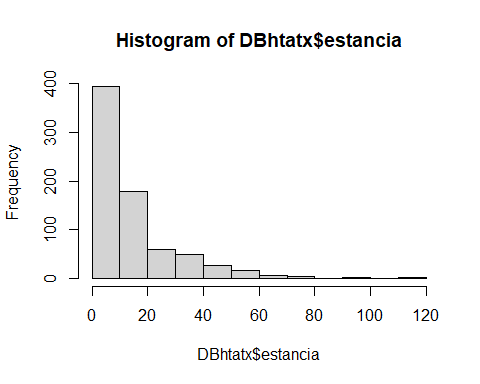


# Validacion modelo lineal----
# Linealidad
anovamod2<-anova(modelo2)
anovamod2

## Analysis of Variance Table
##
## Response: DBhtatx$estancia
## Df Sum Sq Mean Sq F value Pr(>F)
## antihta 1 27025 27025 483.7841 < 2.2e-16 ***
## tiempo_qxco 1 2943 2943 52.6745 1.137e-12 ***
## edad 1 1927 1927 34.4937 6.854e-09 ***
## raza 1 13 13 0.2309 0.6309976
## peso 1 201 201 3.5983 0.0582864 .
## fuma 1 319 319 5.7034 0.0172196 *
## obeso 1 126 126 2.2489 0.1341955
## diabetes 1 0 0 0.0007 0.9786096
## iam 1 310 310 5.5558 0.0187184 *
## ecv 1 583 583 10.4401 0.0012959 **
## dolor 1 12267 12267 219.5962 < 2.2e-16 ***
## insomnio 1 7984 7984 142.9222 < 2.2e-16 ***
## constipa 1 1472 1472 26.3436 3.789e-07 ***
## ansiedad 1 1126 1126 20.1505 8.481e-06 ***
## lev 1 56200 56200 1006.0375 < 2.2e-16 ***
## rts 1 82 82 1.4713 0.2255782
## infx 1 843 843 15.0828 0.0001135 ***
## hb 1 146 146 2.6165 0.1062453
## Residuals 644 35975 56
## ---
## Signif. codes: 0 '***' 0.001 '**' 0.01 '*' 0.05 '.' 0.1 ' ' 1

quantile(DBhtatx$estancia)

## 0% 25% 50% 75% 100%
## 1 6 10 18 113

tail(DBhtatx$estancia)

## [1] 1 1 1 3 1 5

sort(DBhtatx$estancia)

## [1] 1 1 1 1 2 2 2 2 2 2 2 3 3 3 3 3 3 3
## [19] 3 3 3 3 3 3 3 3 3 4 4 4 4 4 4 4 4 4
## [37] 4 4 4 4 4 4 4 4 4 4 4 4 4 4 4 4 4 4
## [55] 4 4 4 4 4 4 4 4 4 4 4 4 4 4 4 4 4 5
## [73] 5 5 5 5 5 5 5 5 5 5 5 5 5 5 5 5 5 5
## [91] 5 5 5 5 5 5 5 5 5 5 5 5 5 5 5 5 5 5
## [109] 5 5 5 5 5 5 5 5 5 5 5 5 5 5 5 5 5 5
## [127] 5 5 5 5 5 5 5 5 5 5 5 5 5 5 5 6 6 6
## [145] 6 6 6 6 6 6 6 6 6 6 6 6 6 6 6 6 6 6
## [163] 6 6 6 6 6 6 6 6 6 6 6 6 6 6 6 6 6 6
## [181] 6 6 6 6 6 6 6 6 6 6 6 6 6 6 6 6 6 6
## [199] 6 6 6 6 6 6 6 6 7 7 7 7 7 7 7 7 7 7
## [217] 7 7 7 7 7 7 7 7 7 7 7 7 7 7 7 7 7 7
## [235] 7 7 7 7 7 7 7 7 7 7 7 7 7 7 7 7 7 7
## [253] 7 7 7 7 7 7 7 7 7 7 7 7 7 7 7 7 7 7
## [271] 7 7 7 7 8 8 8 8 8 8 8 8 8 8 8 8 8 8
## [289] 8 8 8 8 8 8 8 8 8 8 8 8 8 8 8 8 8 8
## [307] 8 8 8 8 8 8 8 8 8 8 8 8 8 8 8 8 8 8
## [325] 9 9 9 9 9 9 9 9 9 9 9 9 9 9 9 9 9 9
## [343] 9 9 9 9 9 9 9 9 9 9 9 9 9 9 9 9 9 9
## [361] 9 9 9 9 10 10 10 10 10 10 10 10 10 10 10 10 10 10
## [379] 10 10 10 10 10 10 10 10 10 10 10 10 10 10 10 10 10 11
## [397] 11 11 11 11 11 11 11 11 11 11 11 11 11 11 11 11 11 11
## [415] 11 11 11 11 11 11 11 11 11 11 11 11 11 11 11 12 12 12
## [433] 12 12 12 12 12 12 12 12 12 12 12 12 12 12 12 12 12 12
## [451] 12 12 12 12 12 12 12 12 12 12 12 12 13 13 13 13 13 13
## [469] 13 13 13 13 13 13 13 13 13 13 13 13 13 13 13 14 14 14
## [487] 14 14 14 14 14 14 14 14 14 14 14 14 14 14 14 14 14 14
## [505] 15 15 15 15 15 15 15 15 15 15 15 15 15 15 15 15 15 15
## [523] 16 16 16 16 16 16 16 16 16 16 16 16 16 16 17 17 17 17
## [541] 17 17 17 18 18 18 18 18 18 18 18 18 18 18 18 19 19 19
## [559] 19 19 19 19 19 19 20 20 20 20 20 20 20 20 20 21 21 21
## [577] 21 21 21 21 21 21 21 21 21 22 22 22 22 22 23 23 23 23
## [595] 24 24 24 24 24 24 24 25 25 25 25 25 26 26 26 26 26 26
## [613] 27 27 28 28 28 28 28 28 28 28 28 29 29 29 30 30 30 30
## [631] 30 30 30 31 31 31 31 31 31 31 32 32 32 32 32 32 33 33
## [649] 33 33 33 33 34 34 34 34 34 34 35 35 35 35 35 35 35 35
## [667] 36 36 36 37 37 37 38 38 38 39 39 39 39 40 40 40 40 41
## [685] 41 42 42 43 43 43 43 44 44 44 45 45 45 46 46 47 47 47
## [703] 47 47 47 48 48 49 49 50 52 53 54 54 54 54 55 55 57 58
## [721] 58 59 59 60 60 60 60 61 63 64 64 65 70 71 74 76 78 92
## [739] 113

# Residuales evaluar graficamente tendencia, componentes cuadraticos o var. omitidas
residuales2<-residuals.lm(modelo2)
hist(residuales2)


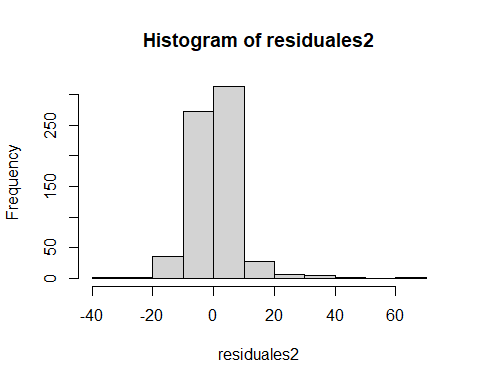


#Normalidad con test de Shapiro wilk
shapiro.test(residuales2)

##
## Shapiro-Wilk normality test
##
## data: residuales2
## W = 0.80075, p-value < 2.2e-16

# Homocedasticidad con test de Brusch Pagan
library(lmtest)

## Loading required package: zoo
##
## Attaching package: 'zoo'
##
## The following objects are masked from 'package:base':
##
## as.Date, as.Date.numeric

bptest(modelo2)

##
## studentized Breusch-Pagan test
##
## data: modelo2
## BP = 128.81, df = 18, p-value < 2.2e-16

# ## VIF
library(car)

## Loading required package: carData
##
## Attaching package: 'car'
##
## The following object is masked from 'package:psych':
##
## logit
##
## The following object is masked from 'package:dplyr':
##
## recode
##
## The following object is masked from 'package:purrr':
##
## some

vif(modelo2)

## antihta tiempo_qxco edad raza peso fuma
## 1.376714 1.034870 1.207427 1.020668 1.691564 1.084164
## obeso diabetes iam ecv dolor insomnio
## 1.648063 1.112249 1.022707 1.039845 1.312934 1.638511
## constipa ansiedad lev rts infx hb
## 1.564217 1.431274 2.673738 1.034976 1.749158 1.414618

alias(modelo2)

## Model :
## DBhtatx$estancia ~ antihta + tiempo_qxco + edad + raza + peso +
## fuma + obeso + diabetes + iam + ecv + dolor + insomnio +
## constipa + ansiedad + lev + rts + infx + hb

# Multicolinearidad
# Corrrelacion para evaluar multicolinealidad
library(corrplot)
#DBhtatx<-na.omit(DBhtatx)
# DBhtatx |>
# select(muerte,antihta,hipot,edad,estancia,pas,pad,hipotPAM,ddd,mg_med,dias_med,ddp,freccard,glasgow,pasrts,rts,lev,dolor,hb,creat,peso)|>
# cor()|> corrplot()


# Estancia y LEV
with(DBhtatx, cor(lev,estancia))

## [1] 0.864101

ggplot(data = DBhtatx, aes(x=lev, y= estancia, color= antihta, fill= antihta))+
 geom_point()+
 geom_smooth(method=lm, se=TRUE, level=0.95)+
 labs(title = "Dispersograma")+
 theme_bw()

## `geom_smooth()` using formula = 'y ~ x'


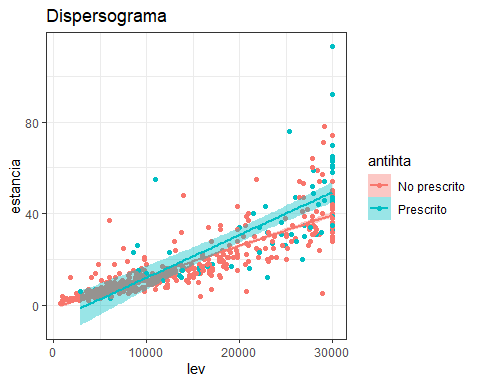


# estancia y dolor
ggplot(DBhtatx, aes(x=DBhtatx$dolor, y=DBhtatx$estancia, color= antihta, fill= antihta)) +
 geom_point() +
 geom_smooth(method=lm, se=TRUE, level=0.95)

## `geom_smooth()` using formula = 'y ~ x'


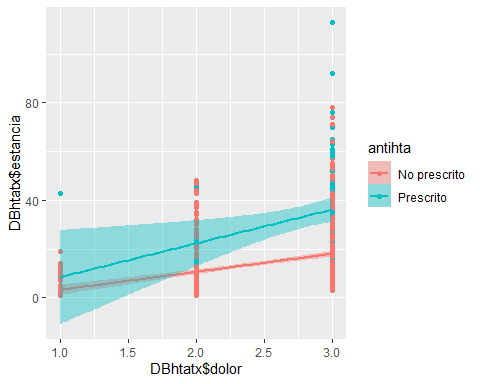


table(DBhtatx$dolor)

##
## 1 2 3
## 43 360 336

# Autocorrelacion de errores

# No se espera autocorrelacion: el diseño no es de series de tiempo, ni de clusters
dwtest(modelo2)

##
## Durbin-Watson test
##
## data: modelo2
## DW = 1.9466, p-value = 0.2354
## alternative hypothesis: true autocorrelation is greater than 0

plot(residuales2)


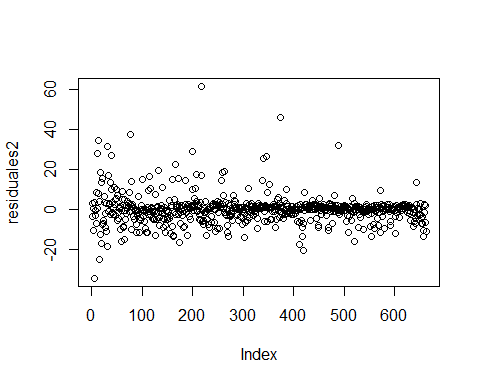


## Presentacion del modelo

#Para presentar la tabla con coeficientes de regresión
library(sjPlot)

## #refugeeswelcome

sjPlot::tab_model(modelo2,show.se=TRUE, show.stat=TRUE)

Dependent variable

Predictors

Estimates

std. Error

CI

Statistic

p

(Intercept)

9.07

4.63

-0.01 – 18.16

1.96

0.050

antihta [Prescrito]

3.69

1.03

1.67 – 5.71

3.58

<0.001

tiempo qxco

0.03

0.01

0.00 – 0.06

2.07

0.039

edad

0.00

0.02

-0.03 – 0.03

0.07

0.942

raza [Negro]

-2.40

2.69

-7.68 – 2.87

-0.89

0.372

peso

-0.08

0.04

-0.15 – -0.00

-2.09

0.037

fuma [Si]

-0.48

0.61

-1.67 – 0.71

-0.80

0.425

obeso [Presente]

3.09

1.47

0.21 – 5.97

2.11

0.035

diabetes [Diabetes]

2.55

1.45

-0.30 – 5.40

1.76

0.079

iam [IAM]

-1.48

4.37

-10.06 – 7.11

-0.34

0.736

ecv [ECV]

1.73

5.40

-8.87 – 12.32

0.32

0.749

dolor

0.01

0.56

-1.10 – 1.11

0.01

0.991

insomnio [Presente]

-0.33

0.76

-1.82 – 1.16

-0.43

0.664

constipa [Presente]

-0.09

0.82

-1.70 – 1.51

-0.11

0.909

ansiedad [Presente]

1.35

0.90

-0.41 – 3.12

1.51

0.132

lev

0.00

0.00

0.00 – 0.00

23.40

<0.001

rts

-0.02

0.02

-0.07 – 0.03

-0.82

0.412

infx [Presente]

3.42

0.89

1.67 – 5.17

3.84

<0.001

hb

-0.22

0.14

-0.49 – 0.05

-1.62

0.106

Observations

663

R2 / R2 adjusted

0.759 / 0.753

# Modelo Logistico para hipotension--------------------------
install.packages("summarytools")
library(summarytools)
colnames(DBhtatx)

## [1] "nombre" "id" "sexo" "raza"
## [5] "edad" "ingreso" "egreso" "estancia"
## [9] "muerte" "pas" "pad" "hipot"
## [13] "hipotPAM" "hipot_lev" "hipot_md" "hipot_vaso"
## [17] "antihta" "clase_med" "ddd" "horario_med"
## [21] "mg_med" "dias_med" "ddp" "freccard"
## [25] "frecresp" "glasgow" "pasrts" "cRTS"
## [29] "rts" "fractura" "gustillo" "qxco"
## [33] "tiempo_qxco" "lev" "aines" "opioid"
## [37] "infx" "etev" "dolor" "insomnio"
## [41] "constipa" "ansiedad" "hb" "creat"
## [45] "peso" "fuma" "alcohol" "obeso"
## [49] "diabetes" "iam" "ecv" "Columna43"
## [53] "glasgow_coded" "pasrts_coded" "frecresp_coded" "Hipot"
## [57] "estancia60" "estancianew" "DDP"

DBhtatx <- DBhtatx %>%
 mutate(Hipot = ifelse(DBhtatx$hipot=="presente",0,1))


DBhtatx$Hipot <- factor(DBhtatx$Hipot,
 levels = c(1,0),
 labels = c("presente", "ausente"))

# + pad + pas + edad + + clase_med + ddd + horario_med + mg_med +
# dias_med + ddp + freccard + glasgow + pasrts + rts + fractura +
# gustillo + qxco + lev + aines + opioid + infx + etev +
# dolor + insomnio + constipa + ansiedad + hb + creat + peso +
# fuma + alcohol + obeso + diabetes + iam + ecv , data = DBhtatx)

quantile(DBhtatx$tiempo_qxco, na.rm = T)

## 0% 25% 50% 75% 100%
## 0 2 3 5 368

table(DBhtatx$ecv)

##
## no ECV ECV
## 737 2

# La famosa tabla 2x2
DBhtatx$antihta <- na.omit(DBhtatx$antihta)
DBhtatx$Hipot <- na.omit(DBhtatx$Hipot)

dospordos <- ctable(DBhtatx$antihta, DBhtatx$Hipot, chisq = TRUE,OR=TRUE)
dospordos

## Cross-Tabulation, Row Proportions
## antihta * Hipot
## Data Frame: DBhtatx
##
##
## -------------- ------- ------------- ------------- --------------
## Hipot presente ausente Total
## antihta
## No prescrito 542 (82.6%) 114 (17.4%) 656 (100.0%)
## Prescrito 15 (18.1%) 68 (81.9%) 83 (100.0%)
## Total 557 (75.4%) 182 (24.6%) 739 (100.0%)
## -------------- ------- ------------- ------------- --------------
##
## ----------------------------
## Chi.squared df p.value
## ------------- ---- ---------
## 161.9227 1 0
## ----------------------------
##
## ----------------------------------
## Odds Ratio Lo - 95% Hi - 95%
## ------------ ---------- ----------
## 21.55 11.89 39.06
## ----------------------------------

# Funcion lineal generalizada: logistica para hipotension que requiera vasopresor, suspension de medicamentos y liquidos endovenosos
# hipot_vaso, hipot_md y hipot_lev (opcion Log-lineal)
modelo1<-glm(Hipot ~ antihta + raza + edad + iam + pas
 + obeso + diabetes + ecv + dolor
 + lev+ hb +creat + ansiedad + tiempo_qxco + rts + peso + fuma + insomnio + infx + constipa + gustillo
 , data = DBhtatx,
 family = binomial)


# tabla del modelo (ojo mediadores? tiempo_pxco + rts + peso + fuma + insomnio + infx + constipa + gustillo )
modelo1

##
## Call: glm(formula = Hipot ~ antihta + raza + edad + iam + pas + obeso +
## diabetes + ecv + dolor + lev + hb + creat + ansiedad + tiempo_qxco +
## rts + peso + fuma + insomnio + infx + constipa + gustillo,
## family = binomial, data = DBhtatx)
##
## Coefficients:
## (Intercept) antihtaPrescrito razaNegro edad
## 1.759e+00 2.372e+00 -1.420e+00 1.104e-02
## iamIAM pas obesoPresente diabetesDiabetes
## 2.348e-01 1.027e-02 7.227e-01 5.271e-01
## ecvECV dolor lev hb
## 8.556e-02 7.077e-02 3.942e-05 -1.838e-01
## creat ansiedadPresente tiempo_qxco rts
## 2.201e-01 8.964e-02 -1.331e-02 -1.673e-02
## peso fumaSi insomnioPresente infxPresente
## -3.186e-02 -9.536e-03 1.209e-01 5.831e-01
## constipaPresente gustilloGA1 gustilloGA2 gustilloGA3
## -4.317e-01 7.083e-01 1.922e-01 3.922e-01
##
## Degrees of Freedom: 598 Total (i.e. Null); 575 Residual
## (140 observations deleted due to missingness)
## Null Deviance: 721.9
## Residual Deviance: 521.4 AIC: 569.4

#table(DBhtatx$hipot)
library(gtsummary)

table(DBhtatx$antihta,DBhtatx$Hipot)

##
## presente ausente
## No prescrito 542 114
## Prescrito 15 68

prop.table(table(DBhtatx$antihta,DBhtatx$Hipot))

##
## presente ausente
## No prescrito 0.73342355 0.15426252
## Prescrito 0.02029770 0.09201624

# Figura con OR e IC 95%
tbl_regression(modelo1 , label = list(antihta~"Antihipertensivos",tiempo_qxco~"Tiempo a cirugía (dias)",
 raza~"Raza", edad~"Edad (años)",peso ~"Peso (Kg)", fuma~"Fumador cigarrillo, tabaco o vaper",
 obeso~ "Antecedente Obesidad",diabetes~"Diabetes Mellitus tipo 1 o 2",
 iam~"Infarto del miocardio",ecv~ "Enfermedad cerebrovascular",dolor~"Dolor por EVA", insomnio~"Insomnio",
 constipa~"Constipación", ansiedad~"Ansiedad", hb~"Hemoglobina (gr/dL)", infx ~"Infeccion clinica",
 lev~"Líquidos endovenosos (mL)", rts~"Puntaje de Trauma (RTS)"), exponentiate = T, add_estimate_to_reference_rows = T)

## Table printed with {flextable}, not {gt}. Learn why at
## https://www.danieldsjoberg.com/gtsummary/articles/rmarkdown.html
## To suppress this message, include `message = FALSE` in the code chunk header.

| **Characteristic** | **OR**^1^ | **95% CI**^1^ | **p-value** |
| --- | --- | --- | --- |
| Antihipertensivos |  |  |  |
| No prescrito | 1.00 | — |  |
| Prescrito | 10.7 | 5.15, 23.6 | <0.001 |
| Raza |  |  |  |
| Mestizo | 1.00 | — |  |
| Negro | 0.24 | 0.01, 1.74 | 0.2 |
| Edad (años) | 1.01 | 1.00, 1.02 | 0.10 |
| Infarto del miocardio |  |  |  |
| no IAM | 1.00 | — |  |
| IAM | 1.26 | 0.03, 31.1 | 0.9 |
| pas | 1.01 | 0.99, 1.03 | 0.2 |
| Antecedente Obesidad |  |  |  |
| Ausente | 1.00 | — |  |
| Presente | 2.06 | 0.72, 5.84 | 0.2 |
| Diabetes Mellitus tipo 1 o 2 |  |  |  |
| no Diabetes | 1.00 | — |  |
| Diabetes | 1.69 | 0.60, 4.54 | 0.3 |
| Enfermedad cerebrovascular |  |  |  |
| no ECV | 1.00 | — |  |
| ECV | 1.09 | 0.03, 37.2 | >0.9 |
| Dolor por EVA | 1.07 | 0.70, 1.66 | 0.7 |
| Líquidos endovenosos (mL) | 1.00 | 1.00, 1.00 | 0.057 |
| Hemoglobina (gr/dL) | 0.83 | 0.75, 0.92 | <0.001 |
| creat | 1.25 | 0.86, 1.79 | 0.2 |
| Ansiedad |  |  |  |
| Ausente | 1.00 | — |  |
| Presente | 1.09 | 0.59, 1.99 | 0.8 |
| Tiempo a cirugía (dias) | 0.99 | 0.95, 1.00 | 0.4 |
| Puntaje de Trauma (RTS) | 0.98 | 0.97, 1.00 | 0.069 |
| Peso (Kg) | 0.97 | 0.94, 1.00 | 0.023 |
| Fumador cigarrillo, tabaco o vaper |  |  |  |
| No | 1.00 | — |  |
| Si | 0.99 | 0.62, 1.57 | >0.9 |
| Insomnio |  |  |  |
| Ausente | 1.00 | — |  |
| Presente | 1.13 | 0.65, 1.95 | 0.7 |
| Infeccion clinica |  |  |  |
| Ausente | 1.00 | — |  |
| Presente | 1.79 | 0.99, 3.24 | 0.054 |
| Constipación |  |  |  |
| Ausente | 1.00 | — |  |
| Presente | 0.65 | 0.36, 1.15 | 0.15 |
| gustillo |  |  |  |
| Cerrada | 1.00 | — |  |
| GA1 | 2.03 | 1.05, 3.92 | 0.035 |
| GA2 | 1.21 | 0.62, 2.34 | 0.6 |
| GA3 | 1.48 | 0.77, 2.85 | 0.2 |
| ^1^OR = Odds Ratio, CI = Confidence Interval | | | |

# Extract coefficients
coefs <- summary(modelo1)$coefficients

# Calculate OR
OR <- exp(coefs[, "Estimate"])

# Calculate 95% CI
CI_lower <- exp(coefs[, "Estimate"] - 1.96 * coefs[, "Std. Error"])
CI_upper <- exp(coefs[, "Estimate"] + 1.96 * coefs[, "Std. Error"])

# Combine into a data frame
results <- data.frame(
 OR = OR,
 CI_lower = CI_lower,
 CI_upper = CI_upper,
 p_value = coefs[, "Pr(>|z|)"]
)

print(results)

## OR CI_lower CI_upper p_value
## (Intercept) 5.8063921 0.11484898 293.5523687 3.795176e-01
## antihtaPrescrito 10.7142864 5.02253961 22.8561528 8.504806e-10
## razaNegro 0.2416819 0.02503618 2.3330287 2.195768e-01
## edad 1.0110996 0.99778327 1.0245937 1.026970e-01
## iamIAM 1.2646937 0.03281099 48.7473974 8.997022e-01
## pas 1.0103247 0.99385489 1.0270674 2.206036e-01
## obesoPresente 2.0599430 0.72897160 5.8210295 1.727106e-01
## diabetesDiabetes 1.6939986 0.61980126 4.6299216 3.041842e-01
## ecvECV 1.0893273 0.04381748 27.0812921 9.583780e-01
## dolor 1.0733346 0.69487661 1.6579162 7.497068e-01
## lev 1.0000394 0.99999889 1.0000800 5.660691e-02
## hb 0.8321295 0.75189636 0.9209241 3.816290e-04
## creat 1.2462435 0.88584064 1.7532756 2.062371e-01
## ansiedadPresente 1.0937755 0.59573292 2.0081899 7.724685e-01
## tiempo_qxco 0.9867741 0.95328973 1.0214346 4.497028e-01
## rts 0.9834119 0.96583919 1.0013044 6.901832e-02
## peso 0.9686422 0.94243747 0.9955756 2.279235e-02
## fumaSi 0.9905097 0.62466520 1.5706164 9.676612e-01
## insomnioPresente 1.1284712 0.65241839 1.9518875 6.654947e-01
## infxPresente 1.7916020 0.99075201 3.2397994 5.369859e-02
## constipaPresente 0.6493745 0.36231515 1.1638685 1.469851e-01
## gustilloGA1 2.0304445 1.05204994 3.9187351 3.475050e-02
## gustilloGA2 1.2118990 0.62614612 2.3456173 5.683857e-01
## gustilloGA3 1.4802518 0.76907197 2.8490773 2.403823e-01

# Interceptos, medidas de ajuste (deviance y criterio de informacion) y residuales
plot (modelo1)


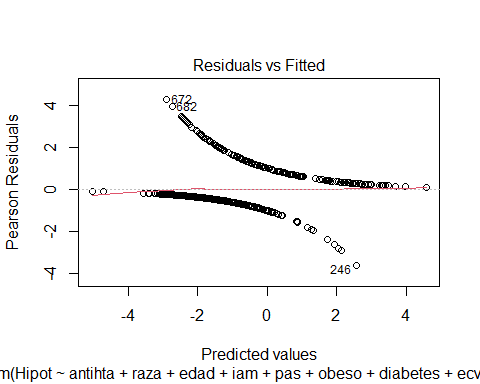

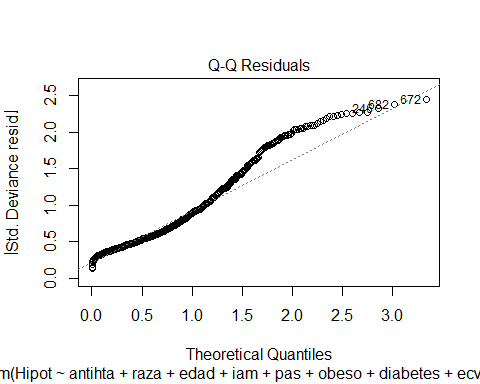

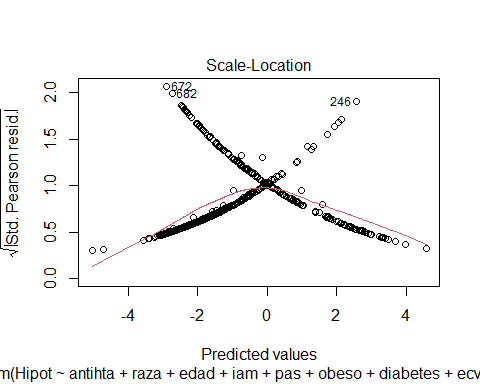

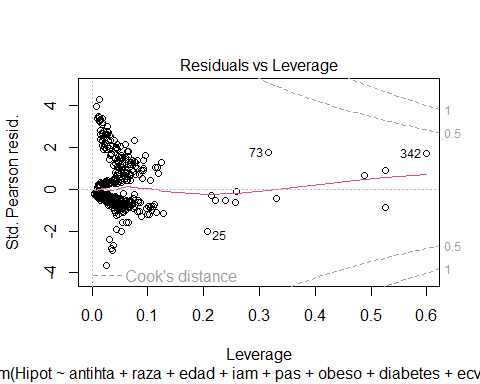


summary.glm(modelo1)

##
## Call:
## glm(formula = Hipot ~ antihta + raza + edad + iam + pas + obeso +
## diabetes + ecv + dolor + lev + hb + creat + ansiedad + tiempo_qxco +
## rts + peso + fuma + insomnio + infx + constipa + gustillo,
## family = binomial, data = DBhtatx)
##
## Coefficients:
## Estimate Std. Error z value Pr(>|z|)
## (Intercept) 1.759e+00 2.002e+00 0.879 0.379518
## antihtaPrescrito 2.372e+00 3.866e-01 6.135 8.5e-10 ***
## razaNegro -1.420e+00 1.157e+00 -1.228 0.219577
## edad 1.104e-02 6.764e-03 1.632 0.102697
## iamIAM 2.348e-01 1.863e+00 0.126 0.899702
## pas 1.027e-02 8.386e-03 1.225 0.220604
## obesoPresente 7.227e-01 5.300e-01 1.364 0.172711
## diabetesDiabetes 5.271e-01 5.130e-01 1.028 0.304184
## ecvECV 8.556e-02 1.639e+00 0.052 0.958378
## dolor 7.077e-02 2.218e-01 0.319 0.749707
## lev 3.942e-05 2.068e-05 1.906 0.056607 .
## hb -1.838e-01 5.173e-02 -3.552 0.000382 ***
## creat 2.201e-01 1.742e-01 1.264 0.206237
## ansiedadPresente 8.964e-02 3.100e-01 0.289 0.772469
## tiempo_qxco -1.331e-02 1.761e-02 -0.756 0.449703
## rts -1.673e-02 9.199e-03 -1.818 0.069018 .
## peso -3.186e-02 1.399e-02 -2.277 0.022792 *
## fumaSi -9.536e-03 2.352e-01 -0.041 0.967661
## insomnioPresente 1.209e-01 2.796e-01 0.432 0.665495
## infxPresente 5.831e-01 3.022e-01 1.929 0.053699 .
## constipaPresente -4.317e-01 2.977e-01 -1.450 0.146985
## gustilloGA1 7.083e-01 3.355e-01 2.111 0.034750 *
## gustilloGA2 1.922e-01 3.369e-01 0.570 0.568386
## gustilloGA3 3.922e-01 3.341e-01 1.174 0.240382
## ---
## Signif. codes: 0 '***' 0.001 '**' 0.01 '*' 0.05 '.' 0.1 ' ' 1
##
## (Dispersion parameter for binomial family taken to be 1)
##
## Null deviance: 721.90 on 598 degrees of freedom
## Residual deviance: 521.37 on 575 degrees of freedom
## (140 observations deleted due to missingness)
## AIC: 569.37
##
## Number of Fisher Scoring iterations: 5

print(summary.glm(modelo1))

##
## Call:
## glm(formula = Hipot ~ antihta + raza + edad + iam + pas + obeso +
## diabetes + ecv + dolor + lev + hb + creat + ansiedad + tiempo_qxco +
## rts + peso + fuma + insomnio + infx + constipa + gustillo,
## family = binomial, data = DBhtatx)
##
## Coefficients:
## Estimate Std. Error z value Pr(>|z|)
## (Intercept) 1.759e+00 2.002e+00 0.879 0.379518
## antihtaPrescrito 2.372e+00 3.866e-01 6.135 8.5e-10 ***
## razaNegro -1.420e+00 1.157e+00 -1.228 0.219577
## edad 1.104e-02 6.764e-03 1.632 0.102697
## iamIAM 2.348e-01 1.863e+00 0.126 0.899702
## pas 1.027e-02 8.386e-03 1.225 0.220604
## obesoPresente 7.227e-01 5.300e-01 1.364 0.172711
## diabetesDiabetes 5.271e-01 5.130e-01 1.028 0.304184
## ecvECV 8.556e-02 1.639e+00 0.052 0.958378
## dolor 7.077e-02 2.218e-01 0.319 0.749707
## lev 3.942e-05 2.068e-05 1.906 0.056607 .
## hb -1.838e-01 5.173e-02 -3.552 0.000382 ***
## creat 2.201e-01 1.742e-01 1.264 0.206237
## ansiedadPresente 8.964e-02 3.100e-01 0.289 0.772469
## tiempo_qxco -1.331e-02 1.761e-02 -0.756 0.449703
## rts -1.673e-02 9.199e-03 -1.818 0.069018 .
## peso -3.186e-02 1.399e-02 -2.277 0.022792 *
## fumaSi -9.536e-03 2.352e-01 -0.041 0.967661
## insomnioPresente 1.209e-01 2.796e-01 0.432 0.665495
## infxPresente 5.831e-01 3.022e-01 1.929 0.053699 .
## constipaPresente -4.317e-01 2.977e-01 -1.450 0.146985
## gustilloGA1 7.083e-01 3.355e-01 2.111 0.034750 *
## gustilloGA2 1.922e-01 3.369e-01 0.570 0.568386
## gustilloGA3 3.922e-01 3.341e-01 1.174 0.240382
## ---
## Signif. codes: 0 '***' 0.001 '**' 0.01 '*' 0.05 '.' 0.1 ' ' 1
##
## (Dispersion parameter for binomial family taken to be 1)
##
## Null deviance: 721.90 on 598 degrees of freedom
## Residual deviance: 521.37 on 575 degrees of freedom
## (140 observations deleted due to missingness)
## AIC: 569.37
##
## Number of Fisher Scoring iterations: 5

library(sjPlot)
tab_model(modelo1, show.se = TRUE, show.stat = TRUE, transform = "exp", show.aic = T, dv.labels = c("Modelo Logistico para Hipotension"),pred.labels = list(antihta~"Antihipertensivos",tiempo_qxco~"Tiempo a cirugía (dias)",
 raza~"Raza", edad~"Edad (años)",peso ~"Peso (Kg)", fuma~"Fumador cigarrillo, tabaco o vaper",
 alcohol~"Consumo de licor", obeso~ "Antecedente Obesidad",diabetes~"Diabetes Mellitus tipo 1 o 2",
 iam~"Infarto del miocardio",ecv~ "Enfermedad cerebrovascular",dolor~"Dolor por EVA", insomnio~"Insomnio",
 constipa~"Constipación", ansiedad~"Ansiedad", hb~"Hemoglobina (gr/dL)", infx ~"Infeccion clinica",
 lev~"Líquidos endovenosos (mL)", rts~"Puntaje de Trauma (RTS)"))

## Length of `pred.labels` does not equal number of predictors, no labelling applied.

Modelo Logistico para Hipotension

Predictors

Odds Ratios

std. Error

CI

Statistic

p

(Intercept)

5.81

11.62

0.12 – 303.17

0.88

0.380

antihtaPrescrito

10.71

4.14

5.15 – 23.57

6.14

<0.001

razaNegro

0.24

0.28

0.01 – 1.74

-1.23

0.220

edad

1.01

0.01

1.00 – 1.02

1.63

0.103

iamIAM

1.26

2.36

0.03 – 31.07

0.13

0.900

pas

1.01

0.01

0.99 – 1.03

1.22

0.221

obesoPresente

2.06

1.09

0.72 – 5.84

1.36

0.173

diabetesDiabetes

1.69

0.87

0.60 – 4.54

1.03

0.304

ecvECV

1.09

1.79

0.03 – 37.25

0.05

0.958

dolor

1.07

0.24

0.70 – 1.66

0.32

0.750

lev

1.00

0.00

1.00 – 1.00

1.91

0.057

hb

0.83

0.04

0.75 – 0.92

-3.55

<0.001

creat

1.25

0.22

0.86 – 1.79

1.26

0.206

ansiedadPresente

1.09

0.34

0.59 – 1.99

0.29

0.772

tiempo_qxco

0.99

0.02

0.95 – 1.00

-0.76

0.450

rts

0.98

0.01

0.97 – 1.00

-1.82

0.069

peso

0.97

0.01

0.94 – 1.00

-2.28

0.023

fumaSi

0.99

0.23

0.62 – 1.57

-0.04

0.968

insomnioPresente

1.13

0.32

0.65 – 1.95

0.43

0.665

infxPresente

1.79

0.54

0.99 – 3.24

1.93

0.054

constipaPresente

0.65

0.19

0.36 – 1.15

-1.45

0.147

gustilloGA1

2.03

0.68

1.05 – 3.92

2.11

0.035

gustilloGA2

1.21

0.41

0.62 – 2.34

0.57

0.568

gustilloGA3

1.48

0.49

0.77 – 2.85

1.17

0.240

Observations

599

R2 Tjur

0.330

AIC

569.366

# Graficar el modelo
ggplot(DBhtatx, aes(x=antihta, y=hipot)) +
 geom_point(size = 0.5, alpha = 0.5) +
 geom_smooth(method = "loess")

## `geom_smooth()` using formula = 'y ~ x'


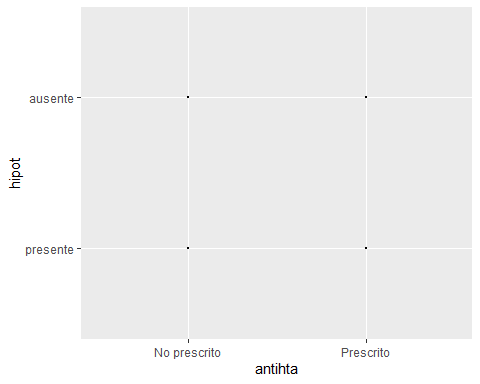


# Validacion modelo logistico----
#Evaluar supuesto de monotonicidad con residuos parciales de los modelos
library(car)
crPlots(modelo1)


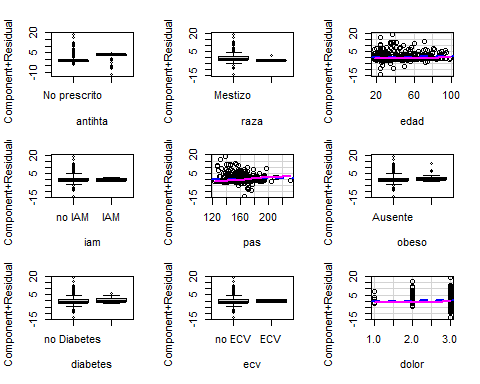

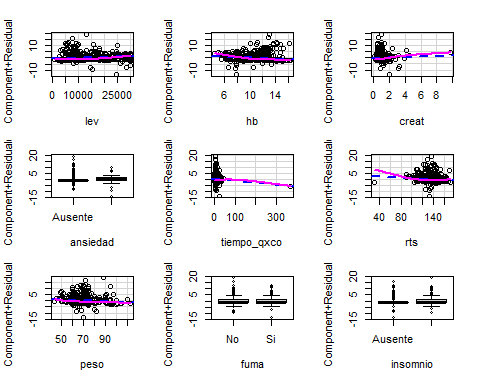

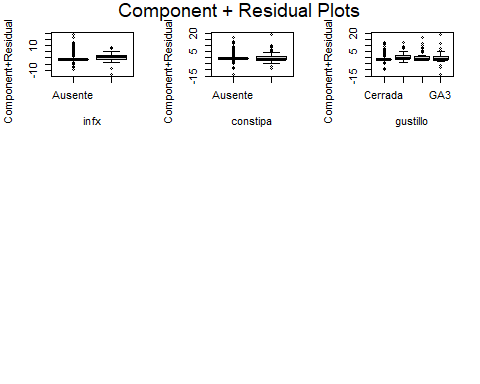


alias(modelo1)

## Model :
## Hipot ~ antihta + raza + edad + iam + pas + obeso + diabetes +
## ecv + dolor + lev + hb + creat + ansiedad + tiempo_qxco +
## rts + peso + fuma + insomnio + infx + constipa + gustillo

# Añadir constatnte positiva pequeña a predictores continuos
DBhtatx <- DBhtatx %>%
 mutate(
 edad = edad + 0.0001,
 peso = peso + 0.0001,
 hb = hb + 0.0001,
 )

# Ajuste de regresion con transformacion the Box-Tidwell para interaccion con la var continua
box_tidwell_model <- glm(
 Hipot ~ antihta + raza +
 edad * log(edad) +
 peso * log(peso) +
 fuma + alcohol + obeso + diabetes + iam + ecv +
 dolor + insomnio + constipa + ansiedad + lev +
 rts + infx + hb * log(hb) + infx,
 data = DBhtatx,
 family = binomial(link = "logit")
)

# resumen
summary(box_tidwell_model)

##
## Call:
## glm(formula = Hipot ~ antihta + raza + edad * log(edad) + peso *
## log(peso) + fuma + alcohol + obeso + diabetes + iam + ecv +
## dolor + insomnio + constipa + ansiedad + lev + rts + infx +
## hb * log(hb) + infx, family = binomial(link = "logit"), data = DBhtatx)
##
## Coefficients: (1 not defined because of singularities)
## Estimate Std. Error z value Pr(>|z|)
## (Intercept) 4.666e+02 2.332e+02 2.001 0.04544 *
## antihtaPrescrito 2.448e+00 3.802e-01 6.440 1.2e-10 ***
## razaNegro -1.439e+00 1.161e+00 -1.239 0.21518
## edad 3.942e-01 1.011e+00 0.390 0.69669
## log(edad) -5.316e+00 7.598e+00 -0.700 0.48415
## peso 1.553e+01 8.984e+00 1.729 0.08378 .
## log(peso) -1.858e+02 1.023e+02 -1.817 0.06917 .
## fumaSi -7.791e-02 2.320e-01 -0.336 0.73702
## alcoholConsumo -5.923e-01 3.094e-01 -1.914 0.05556 .
## obesoPresente 5.629e-01 6.648e-01 0.847 0.39716
## diabetesDiabetes 5.953e-01 5.170e-01 1.152 0.24947
## iamIAM 6.256e-01 1.811e+00 0.346 0.72972
## ecvECV -1.072e+00 1.617e+00 -0.663 0.50724
## dolor 2.030e-01 2.202e-01 0.922 0.35654
## insomnioPresente 2.119e-01 2.831e-01 0.749 0.45410
## constipaPresente NA NA NA NA
## ansiedadPresente 4.441e-03 3.146e-01 0.014 0.98874
## lev 5.336e-05 1.967e-05 2.712 0.00669 **
## rts -1.229e-02 8.802e-03 -1.397 0.16253
## infxPresente 8.132e-01 3.014e-01 2.698 0.00697 **
## hb 2.464e+01 1.604e+01 1.536 0.12465
## log(hb) -6.158e+01 3.656e+01 -1.684 0.09214 .
## edad:log(edad) -5.441e-02 1.732e-01 -0.314 0.75344
## peso:log(peso) -2.454e+00 1.431e+00 -1.715 0.08639 .
## hb:log(hb) -5.605e+00 3.732e+00 -1.502 0.13311
## ---
## Signif. codes: 0 '***' 0.001 '**' 0.01 '*' 0.05 '.' 0.1 ' ' 1
##
## (Dispersion parameter for binomial family taken to be 1)
##
## Null deviance: 772.63 on 664 degrees of freedom
## Residual deviance: 540.40 on 641 degrees of freedom
## (74 observations deleted due to missingness)
## AIC: 588.4
##
## Number of Fisher Scoring iterations: 5

# Medidas de ajuste (mas util en predictivo)
logisticPseudoR2s <- function(modelo1) {
 dev <- modelo1$deviance
 nullDev <- modelo1$null.deviance
 modelN <- length(modelo1$fitted.values)
 R.l <- 1 - dev / nullDev
 R.cs <- 1- exp ( -(nullDev - dev) / modelN)
 R.n <- R.cs / ( 1 - ( exp (-(nullDev / modelN))))
 cat("Pseudo R^2 for logistic regression\n")
 cat("Hosmer and Lemeshow R^2 ", round(R.l, 3), "\n")
 cat("Cox and Snell R^2 ", round(R.cs, 3), "\n")
 cat("Nagelkerke R^2 ", round(R.n, 3), "\n")
}

logisticPseudoR2s(modelo1)

## Pseudo R^2 for logistic regression
## Hosmer and Lemeshow R^2 0.278
## Cox and Snell R^2 0.285
## Nagelkerke R^2 0.406

# Submodelos de tipos de hipotension----
table(DBhtatx$antihta)

##
## No prescrito Prescrito
## 656 83

# Hipotension que requiera LIQUIDOS
table(DBhtatx$hipot_lev)

##
## presente ausente
## 99 640

DBhtatx <- DBhtatx %>%
 mutate(Hipot_lev = ifelse(DBhtatx$hipot_lev=="presente",0,1))

table(DBhtatx$Hipot_lev)

##
## 0 1
## 99 640

DBhtatx$Hipot_lev <- factor(DBhtatx$Hipot_lev,
 levels = c(1,0),
 labels = c("presente", "ausente"))

table(DBhtatx$Hipot_lev)

##
## presente ausente
## 640 99

modelo1a<-glm(Hipot_lev ~ antihta + raza + edad + iam + pas
 + alcohol + obeso + diabetes + ecv + dolor
 + lev+ hb + ansiedad + tiempo_qxco + rts + peso + fuma + insomnio + infx + constipa + gustillo
 , data = DBhtatx,
 family = binomial)

modelo1a

##
## Call: glm(formula = Hipot_lev ~ antihta + raza + edad + iam + pas +
## alcohol + obeso + diabetes + ecv + dolor + lev + hb + ansiedad +
## tiempo_qxco + rts + peso + fuma + insomnio + infx + constipa +
## gustillo, family = binomial, data = DBhtatx)
##
## Coefficients:
## (Intercept) antihtaPrescrito razaNegro edad
## -1.688e+00 3.380e+00 -1.625e+01 -1.541e-03
## iamIAM pas alcoholConsumo obesoPresente
## 1.430e+00 2.684e-02 -3.628e-01 9.474e-01
## diabetesDiabetes ecvECV dolor lev
## -3.753e-01 -1.619e+01 -5.045e-01 5.709e-05
## hb ansiedadPresente tiempo_qxco rts
## -1.634e-01 -1.908e-01 -3.693e-03 -1.021e-02
## peso fumaSi insomnioPresente infxPresente
## -3.698e-02 -1.514e-01 9.854e-01 1.018e+00
## constipaPresente gustilloGA1 gustilloGA2 gustilloGA3
## NA 5.672e-02 7.064e-02 6.691e-01
##
## Degrees of Freedom: 662 Total (i.e. Null); 640 Residual
## (76 observations deleted due to missingness)
## Null Deviance: 555.5
## Residual Deviance: 275.5 AIC: 321.5

# Hipotension que requiera suspender MDS
table(DBhtatx$hipot_md)

##
## presente ausente
## 63 676

DBhtatx <- DBhtatx %>%
 mutate(Hipot_md = ifelse(DBhtatx$hipot_md=="presente",0,1))

table(DBhtatx$Hipot_md)

##
## 0 1
## 63 676

DBhtatx$Hipot_md <- factor(DBhtatx$Hipot_md,
 levels = c(1,0),
 labels = c("presente", "ausente"))

table(DBhtatx$Hipot_md)

##
## presente ausente
## 676 63

modelo1b<-glm(Hipot_md ~ antihta + raza + edad + iam + pas
 + alcohol + obeso + diabetes + ecv + dolor
 + lev+ hb + ansiedad + tiempo_qxco + rts + peso + fuma + insomnio + infx + constipa + gustillo
 , data = DBhtatx,
 family = binomial)

modelo1b

##
## Call: glm(formula = Hipot_md ~ antihta + raza + edad + iam + pas +
## alcohol + obeso + diabetes + ecv + dolor + lev + hb + ansiedad +
## tiempo_qxco + rts + peso + fuma + insomnio + infx + constipa +
## gustillo, family = binomial, data = DBhtatx)
##
## Coefficients:
## (Intercept) antihtaPrescrito razaNegro edad
## -1.399e+00 3.359e+00 -1.493e+01 7.895e-03
## iamIAM pas alcoholConsumo obesoPresente
## 1.781e+00 1.789e-02 -3.789e-01 -1.516e-01
## diabetesDiabetes ecvECV dolor lev
## -1.161e+00 -1.545e+01 -1.994e-01 3.740e-06
## hb ansiedadPresente tiempo_qxco rts
## -1.542e-01 3.186e-01 -1.033e-02 -2.559e-02
## peso fumaSi insomnioPresente infxPresente
## -1.170e-02 5.537e-01 6.125e-02 1.382e+00
## constipaPresente gustilloGA1 gustilloGA2 gustilloGA3
## NA 4.392e-01 4.234e-01 7.678e-01
##
## Degrees of Freedom: 662 Total (i.e. Null); 640 Residual
## (76 observations deleted due to missingness)
## Null Deviance: 416.4
## Residual Deviance: 215.3 AIC: 261.3

# Hipotension que requiera VASOPRESOR
table(DBhtatx$hipot_vaso)

##
## presente ausente
## 34 705

DBhtatx <- DBhtatx %>%
 mutate(Hipot_vaso = ifelse(DBhtatx$hipot_vaso=="presente",0,1))

table(DBhtatx$Hipot_vaso)

##
## 0 1
## 34 705

DBhtatx$Hipot_vaso <- factor(DBhtatx$Hipot_vaso,
 levels = c(1,0),
 labels = c("presente", "ausente"))

table(DBhtatx$Hipot_vaso)

##
## presente ausente
## 705 34

modelo1c<-glm(Hipot_lev ~ antihta + raza + edad + iam + pas
 + alcohol + obeso + diabetes + ecv + dolor
 + lev+ hb + ansiedad + tiempo_qxco + rts + peso + fuma + insomnio + infx + constipa + gustillo
 , data = DBhtatx,
 family = binomial)

modelo1c

##
## Call: glm(formula = Hipot_lev ~ antihta + raza + edad + iam + pas +
## alcohol + obeso + diabetes + ecv + dolor + lev + hb + ansiedad +
## tiempo_qxco + rts + peso + fuma + insomnio + infx + constipa +
## gustillo, family = binomial, data = DBhtatx)
##
## Coefficients:
## (Intercept) antihtaPrescrito razaNegro edad
## -1.688e+00 3.380e+00 -1.625e+01 -1.541e-03
## iamIAM pas alcoholConsumo obesoPresente
## 1.430e+00 2.684e-02 -3.628e-01 9.474e-01
## diabetesDiabetes ecvECV dolor lev
## -3.753e-01 -1.619e+01 -5.045e-01 5.709e-05
## hb ansiedadPresente tiempo_qxco rts
## -1.634e-01 -1.908e-01 -3.693e-03 -1.021e-02
## peso fumaSi insomnioPresente infxPresente
## -3.698e-02 -1.514e-01 9.854e-01 1.018e+00
## constipaPresente gustilloGA1 gustilloGA2 gustilloGA3
## NA 5.672e-02 7.064e-02 6.691e-01
##
## Degrees of Freedom: 662 Total (i.e. Null); 640 Residual
## (76 observations deleted due to missingness)
## Null Deviance: 555.5
## Residual Deviance: 275.5 AIC: 321.5

# Competing risks para tiempo al egreso vivo-----------------------
# Load necessary libraries
library(survival)
library(car)
library(caret)

## Loading required package: lattice
##
## Attaching package: 'caret'
##
## The following object is masked from 'package:survival':
##
## cluster
##
## The following object is masked from 'package:purrr':
##
## lift

# Muerte
table(DBhtatx$muerte)

##
## sobrevivio fallecio
## 728 11

#Recodifico muerte a factor (Muerte)
DBhtatx$Muerte <- ifelse(DBhtatx$muerte == "fallecio", 1,0)

table(DBhtatx$Muerte)

##
## 0 1
## 728 11

# Crearemos el objeto de supervivencia
library(survival)

# Pero no es la muerte lo que queremos modelar, es el egreso vivo, creamos la variable

DBhtatx <- DBhtatx %>%
 mutate(altavivo = ifelse(DBhtatx$Muerte==1,0,1))

table(DBhtatx$altavivo)

##
## 0 1
## 11 728

# altavivo = 1 ~ "egreso vivo",
# Muerte = 0 ~ "egreso muerto"
# ))

# Es decir creamos la variable dependiente como la combinación de la variable tiempo (continua) con la variable desenlace ( Status dicotómica)
DBhtatx.surv <- Surv(DBhtatx$estancia, DBhtatx$altavivo) #Creando objeto tipo Surv
DBhtatx.km <- survfit(DBhtatx.surv ~ 1, data = DBhtatx)
DBhtatx.km

## Call: survfit(formula = DBhtatx.surv ~ 1, data = DBhtatx)
##
## n events median 0.95LCL 0.95UCL
## [1,] 739 728 10 9 11

summary(DBhtatx.km)

## Call: survfit(formula = DBhtatx.surv ~ 1, data = DBhtatx)
##
## time n.risk n.event survival std.err lower 95% CI upper 95% CI
## 1 739 4 0.99459 0.00270 0.989311 0.9999
## 2 735 6 0.98647 0.00425 0.978173 0.9948
## 3 728 15 0.96614 0.00666 0.953185 0.9793
## 4 712 44 0.90644 0.01072 0.885660 0.9277
## 5 668 70 0.81145 0.01441 0.783701 0.8402
## 6 598 65 0.72325 0.01648 0.691662 0.7563
## 7 533 67 0.63233 0.01776 0.598466 0.6681
## 8 465 50 0.56434 0.01827 0.529647 0.6013
## 9 415 40 0.50995 0.01842 0.475090 0.5474
## 10 375 30 0.46915 0.01839 0.434454 0.5066
## 11 344 34 0.42278 0.01821 0.388551 0.4600
## 12 310 33 0.37778 0.01788 0.344309 0.4145
## 13 277 21 0.34914 0.01758 0.316321 0.3854
## 14 256 20 0.32186 0.01723 0.289793 0.3575
## 15 235 16 0.29995 0.01691 0.268570 0.3350
## 16 217 13 0.28198 0.01661 0.251223 0.3165
## 17 203 7 0.27225 0.01644 0.241860 0.3065
## 18 196 12 0.25558 0.01613 0.225856 0.2892
## 19 184 8 0.24447 0.01590 0.215222 0.2777
## 20 175 8 0.23330 0.01565 0.204550 0.2661
## 21 166 12 0.21643 0.01526 0.188499 0.2485
## 22 154 5 0.20940 0.01508 0.181833 0.2412
## 23 149 4 0.20378 0.01494 0.176511 0.2353
## 24 145 7 0.19395 0.01467 0.167218 0.2249
## 25 138 5 0.18692 0.01447 0.160598 0.2176
## 26 133 6 0.17849 0.01422 0.152675 0.2087
## 27 127 2 0.17568 0.01414 0.150039 0.2057
## 28 125 9 0.16303 0.01373 0.138212 0.1923
## 29 116 3 0.15881 0.01359 0.134283 0.1878
## 30 113 7 0.14897 0.01325 0.125140 0.1773
## 31 106 6 0.14054 0.01294 0.117335 0.1683
## 32 99 6 0.13202 0.01261 0.109476 0.1592
## 33 93 6 0.12351 0.01227 0.101652 0.1501
## 34 87 6 0.11499 0.01191 0.093867 0.1409
## 35 81 8 0.10363 0.01139 0.083551 0.1285
## 36 73 3 0.09937 0.01118 0.079704 0.1239
## 37 70 3 0.09511 0.01097 0.075870 0.1192
## 38 67 3 0.09085 0.01075 0.072049 0.1146
## 39 64 4 0.08518 0.01045 0.066976 0.1083
## 40 60 4 0.07950 0.01013 0.061930 0.1020
## 41 56 2 0.07666 0.00996 0.059418 0.0989
## 42 54 2 0.07382 0.00980 0.056914 0.0957
## 43 52 4 0.06814 0.00944 0.051932 0.0894
## 44 48 3 0.06388 0.00917 0.048218 0.0846
## 45 45 3 0.05962 0.00888 0.044528 0.0798
## 46 42 2 0.05678 0.00868 0.042081 0.0766
## 47 40 6 0.04827 0.00805 0.034814 0.0669
## 48 34 2 0.04543 0.00782 0.032419 0.0637
## 49 32 2 0.04259 0.00758 0.030041 0.0604
## 50 30 1 0.04117 0.00746 0.028858 0.0587
## 52 29 1 0.03975 0.00734 0.027680 0.0571
## 53 28 1 0.03833 0.00721 0.026506 0.0554
## 54 27 4 0.03265 0.00668 0.021865 0.0488
## 55 23 2 0.02981 0.00639 0.019581 0.0454
## 57 21 1 0.02839 0.00624 0.018449 0.0437
## 58 20 2 0.02555 0.00593 0.016209 0.0403
## 59 18 2 0.02271 0.00560 0.014005 0.0368
## 60 16 4 0.01704 0.00487 0.009728 0.0298
## 61 12 1 0.01562 0.00467 0.008694 0.0280
## 63 11 1 0.01420 0.00445 0.007677 0.0263
## 64 10 2 0.01136 0.00399 0.005705 0.0226
## 65 8 1 0.00994 0.00373 0.004757 0.0208
## 70 7 1 0.00852 0.00346 0.003842 0.0189
## 71 6 1 0.00710 0.00316 0.002965 0.0170
## 74 5 1 0.00568 0.00283 0.002138 0.0151
## 76 4 1 0.00426 0.00245 0.001377 0.0132
## 78 3 1 0.00284 0.00200 0.000712 0.0113
## 92 2 1 0.00142 0.00142 0.000200 0.0101
## 113 1 1 0.00000 NaN NA NA

# vamos a omitir datos perdidos por ahora
# DBhtatx<-na.omit(DBhtatx)


# Curva de riesgo acumulado
#R <- DBhtatx.km %>% fortify(DBhtatx) %>% mutate(CumHaz = cumsum(n.event/n.risk))

plot(DBhtatx.km, fun = "cumhaz", conf.int = F, main = "Riesgo Acumulado", col = 1:2,
 xlab = "Tiempo (Dias)", ylab = "Riesgo Acumulado")


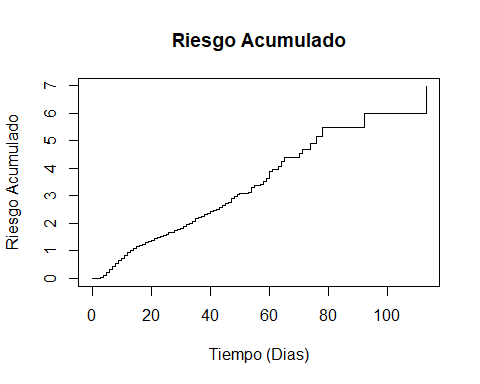


print(DBhtatx.km, print.rmean = TRUE)

## Call: survfit(formula = DBhtatx.surv ~ 1, data = DBhtatx)
##
## n events rmean* se(rmean) median 0.95LCL 0.95UCL
## [1,] 739 728 15.6 0.551 10 9 11
## * restricted mean with upper limit = 113

#quantile(DBhtatx.km, c(0.05, 0.25, 0.95))

# Supervivencia GLOBAL
library(ggplot2)
library(survminer)

## Loading required package: ggpubr
##
## Attaching package: 'survminer'
##
## The following object is masked from 'package:survival':
##
## myeloma

ggsurvplot(fit = DBhtatx.km, data = DBhtatx, conf.int = T, title = "Curva de Egreso vivo Hipertension en Trauma", color = "tomato",
 xlab = "Tiempo (en dias)", ylab = "Probabilidad de egreso vivo", legend.title = "Estimación",
 legend.labs = "Kaplan-Meier")


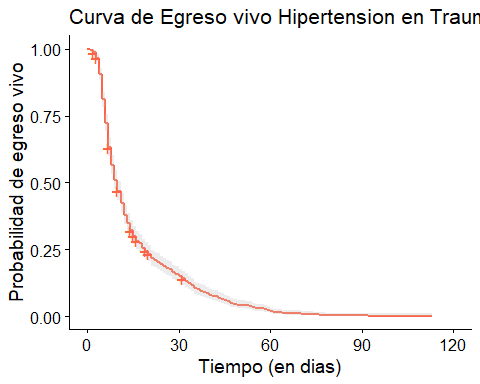


table(DBhtatx$antihta)

##
## No prescrito Prescrito
## 656 83

# Supervivencia con prescripcion de antihipertensivo y sin este
survfit(Surv(estancia, altavivo) ~ antihta, DBhtatx, conf.type = "log-log") %>%
 ggsurvplot(title = "Egreso vivo por prescripcion Antihipertensivo", xlab ="Tiempo (dias)" , ylab = "Probabilidad de Egreso vivo",
 conf.int = T, legend.title = "Antihipertensivo", legend.labs = c("No Prescrito", "Prescrito"))


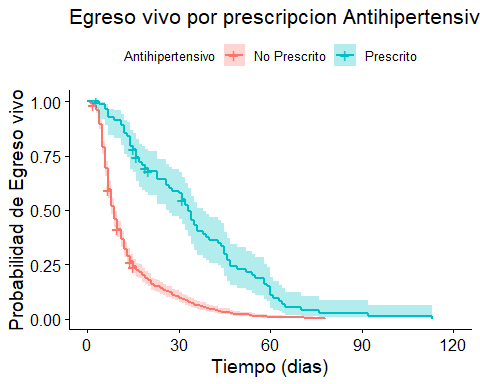


#Log rank Test
diffsfit <- survdiff(Surv(estancia, Muerte) ~ antihta, data=DBhtatx)


print(diffsfit)

## Call:
## survdiff(formula = Surv(estancia, Muerte) ~ antihta, data = DBhtatx)
##
## N Observed Expected (O-E)^2/E (O-E)^2/V
## antihta=No prescrito 656 5 8.28 1.30 5.51
## antihta=Prescrito 83 6 2.72 3.97 5.51
##
## Chisq= 5.5 on 1 degrees of freedom, p= 0.02

# MODELO DE Cox ajustado por las variables

#+ pad + pas + edad + + clase_med + ddd + horario_med + mg_med +
# dias_med + ddp + freccard + glasgow + pasrts + rts + fractura +
# gustillo + qxco + lev + aines + opioid + infx + etev +
# dolor + insomnio + constipa + ansiedad + hb + creat + peso +
# fuma + alcohol + obeso + diabetes + iam + ecv


modelo3<-coxph(DBhtatx.surv~antihta + edad + sexo + raza+ rts + tiempo_qxco + hb + infx + aines + opioid + etev
 + lev + fuma + rts + alcohol + obeso + diabetes + iam +ecv + dolor, data = DBhtatx)

modelo3

## Call:
## coxph(formula = DBhtatx.surv ~ antihta + edad + sexo + raza +
## rts + tiempo_qxco + hb + infx + aines + opioid + etev + lev +
## fuma + rts + alcohol + obeso + diabetes + iam + ecv + dolor,
## data = DBhtatx)
##
## coef exp(coef) se(coef) z p
## antihtaPrescrito -3.261e-01 7.217e-01 1.434e-01 -2.274 0.02294
## edad 1.051e-03 1.001e+00 2.262e-03 0.465 0.64213
## sexoHombre 2.678e-01 1.307e+00 1.056e-01 2.536 0.01122
## razaNegro 6.637e-01 1.942e+00 3.609e-01 1.839 0.06589
## rts 2.363e-03 1.002e+00 3.517e-03 0.672 0.50164
## tiempo_qxco -2.000e-02 9.802e-01 6.997e-03 -2.859 0.00426
## hb 3.010e-02 1.031e+00 1.928e-02 1.561 0.11844
## infxPresente -6.255e-01 5.350e-01 1.242e-01 -5.038 4.70e-07
## ainesPrescrito -1.315e+00 2.684e-01 7.195e-01 -1.828 0.06753
## opioidPrescrito 2.559e-01 1.292e+00 4.165e-01 0.614 0.53889
## etevPresente -5.531e-01 5.751e-01 4.088e-01 -1.353 0.17602
## lev -1.980e-04 9.998e-01 9.671e-06 -20.470 < 2e-16
## fumaSi 2.678e-01 1.307e+00 8.567e-02 3.126 0.00177
## alcoholConsumo 9.063e-02 1.095e+00 1.031e-01 0.879 0.37951
## obesoPresente -8.392e-02 9.195e-01 1.681e-01 -0.499 0.61759
## diabetesDiabetes -1.176e+00 3.086e-01 2.227e-01 -5.278 1.31e-07
## iamIAM 1.084e+00 2.958e+00 5.880e-01 1.844 0.06512
## ecvECV 4.423e-01 1.556e+00 7.214e-01 0.613 0.53980
## dolor -1.989e-01 8.196e-01 7.711e-02 -2.579 0.00991
##
## Likelihood ratio test=950.7 on 19 df, p=< 2.2e-16
## n= 663, number of events= 652
## (76 observations deleted due to missingness)

tbl_regression(modelo3, label = list(antihta~"Antihipertensivos",tiempo_qxco~"Tiempo a cirugía (dias)", sexo~"Sexo",
 raza~"Raza", edad~"Edad (años)", fuma~"Fumador cigarrillo, tabaco o vaper",
 alcohol~"Consumo de licor", obeso~ "Antecedente Obesidad",diabetes~"Diabetes Mellitus tipo 1 o 2",
 iam~"Infarto del miocardio",ecv~ "Enfermedad cerebrovascular",dolor~"Dolor por EVA", hb~"Hemoglobina (gr/dL)", infx ~"Infeccion clinica",
 lev~"Líquidos endovenosos (mL)", rts~"Puntaje de Trauma (RTS)", etev~"Enfermedad tromboembolica venosa"), exponentiate = T, add_estimate_to_reference_rows = T)

## Table printed with {flextable}, not {gt}. Learn why at
## https://www.danieldsjoberg.com/gtsummary/articles/rmarkdown.html
## To suppress this message, include `message = FALSE` in the code chunk header.

| **Characteristic** | **HR**^1^ | **95% CI**^1^ | **p-value** |
| --- | --- | --- | --- |
| Antihipertensivos |  |  |  |
| No prescrito | 1.00 | — |  |
| Prescrito | 0.72 | 0.54, 0.96 | 0.023 |
| Edad (años) | 1.00 | 1.00, 1.01 | 0.6 |
| Sexo |  |  |  |
| Mujer | 1.00 | — |  |
| Hombre | 1.31 | 1.06, 1.61 | 0.011 |
| Raza |  |  |  |
| Mestizo | 1.00 | — |  |
| Negro | 1.94 | 0.96, 3.94 | 0.066 |
| Puntaje de Trauma (RTS) | 1.00 | 1.00, 1.01 | 0.5 |
| Tiempo a cirugía (dias) | 0.98 | 0.97, 0.99 | 0.004 |
| Hemoglobina (gr/dL) | 1.03 | 0.99, 1.07 | 0.12 |
| Infeccion clinica |  |  |  |
| Ausente | 1.00 | — |  |
| Presente | 0.53 | 0.42, 0.68 | <0.001 |
| aines |  |  |  |
| No prescrito | 1.00 | — |  |
| Prescrito | 0.27 | 0.07, 1.10 | 0.068 |
| opioid |  |  |  |
| No prescrito | 1.00 | — |  |
| Prescrito | 1.29 | 0.57, 2.92 | 0.5 |
| Enfermedad tromboembolica venosa |  |  |  |
| Ausente | 1.00 | — |  |
| Presente | 0.58 | 0.26, 1.28 | 0.2 |
| Líquidos endovenosos (mL) | 1.00 | 1.00, 1.00 | <0.001 |
| Fumador cigarrillo, tabaco o vaper |  |  |  |
| No | 1.00 | — |  |
| Si | 1.31 | 1.11, 1.55 | 0.002 |
| Consumo de licor |  |  |  |
| No consumo | 1.00 | — |  |
| Consumo | 1.09 | 0.89, 1.34 | 0.4 |
| Antecedente Obesidad |  |  |  |
| Ausente | 1.00 | — |  |
| Presente | 0.92 | 0.66, 1.28 | 0.6 |
| Diabetes Mellitus tipo 1 o 2 |  |  |  |
| no Diabetes | 1.00 | — |  |
| Diabetes | 0.31 | 0.20, 0.48 | <0.001 |
| Infarto del miocardio |  |  |  |
| no IAM | 1.00 | — |  |
| IAM | 2.96 | 0.93, 9.36 | 0.065 |
| Enfermedad cerebrovascular |  |  |  |
| no ECV | 1.00 | — |  |
| ECV | 1.56 | 0.38, 6.40 | 0.5 |
| Dolor por EVA | 0.82 | 0.70, 0.95 | 0.010 |
| ^1^HR = Hazard Ratio, CI = Confidence Interval | | | |

tab_model(modelo3, show.se = TRUE, show.stat = TRUE, transform = "exp", show.aic = T, dv.labels = c("Modelo para Riesgo de muerte "))

Modelo para Riesgo de muerte

Predictors

Estimates

std. Error

CI

Statistic

p

antihta [Prescrito]

0.72

0.10

0.54 – 0.96

-2.27

0.023

edad

1.00

0.00

1.00 – 1.01

0.46

0.642

sexo [Hombre]

1.31

0.14

1.06 – 1.61

2.54

0.011

raza [Negro]

1.94

0.70

0.96 – 3.94

1.84

0.066

rts

1.00

0.00

1.00 – 1.01

0.67

0.502

tiempo qxco

0.98

0.01

0.97 – 0.99

-2.86

0.004

hb

1.03

0.02

0.99 – 1.07

1.56

0.118

infx [Presente]

0.53

0.07

0.42 – 0.68

-5.04

<0.001

aines [Prescrito]

0.27

0.19

0.07 – 1.10

-1.83

0.068

opioid [Prescrito]

1.29

0.54

0.57 – 2.92

0.61

0.539

etev [Presente]

0.58

0.24

0.26 – 1.28

-1.35

0.176

lev

1.00

0.00

1.00 – 1.00

-20.47

<0.001

fuma [Si]

1.31

0.11

1.11 – 1.55

3.13

0.002

alcohol [Consumo]

1.09

0.11

0.89 – 1.34

0.88

0.380

obeso [Presente]

0.92

0.15

0.66 – 1.28

-0.50

0.618

diabetes [Diabetes]

0.31

0.07

0.20 – 0.48

-5.28

<0.001

iam [IAM]

2.96

1.74

0.93 – 9.36

1.84

0.065

ecvECV

1.56

1.12

0.38 – 6.40

0.61

0.540

dolor

0.82

0.06

0.70 – 0.95

-2.58

0.010

Observations

663

R2 Nagelkerke

0.762

AIC

6261.772

library(dplyr)

# Assuming your data frame is named 'DBhtatx'
# MODELO estancia menor a 60 dias riesgos que compiten: Base de datos con los casos estancia < 60----

DBhtatx_filtered <- DBhtatx %>%
 filter(estancia < 60)

# De nuevo, creamos la variable dependiente como la combinación de la variable tiempo (continua) con la variable desenlace ( Status dicotómica)
DBhtatx.survnew<- Surv(DBhtatx_filtered$estancia, DBhtatx_filtered$altavivo) #Creando objeto tipo Surv
DBhtatx.kmnew <- survfit(DBhtatx.survnew ~ 1, data = DBhtatx_filtered)
DBhtatx.kmnew

## Call: survfit(formula = DBhtatx.survnew ~ 1, data = DBhtatx_filtered)
##
## n events median 0.95LCL 0.95UCL
## [1,] 723 712 9 9 10

summary(DBhtatx.kmnew)

## Call: survfit(formula = DBhtatx.survnew ~ 1, data = DBhtatx_filtered)
##
## time n.risk n.event survival std.err lower 95% CI upper 95% CI
## 1 723 4 0.99447 0.00276 0.989075 0.9999
## 2 719 6 0.98617 0.00434 0.977692 0.9947
## 3 712 15 0.96539 0.00680 0.952155 0.9788
## 4 696 44 0.90436 0.01095 0.883154 0.9261
## 5 652 70 0.80727 0.01469 0.778987 0.8366
## 6 582 65 0.71711 0.01677 0.684977 0.7507
## 7 517 67 0.62418 0.01804 0.589807 0.6605
## 8 449 50 0.55467 0.01851 0.519543 0.5922
## 9 399 40 0.49906 0.01863 0.463854 0.5369
## 10 359 30 0.45736 0.01856 0.422385 0.4952
## 11 328 34 0.40995 0.01833 0.375546 0.4475
## 12 294 33 0.36393 0.01794 0.330418 0.4009
## 13 261 21 0.33465 0.01760 0.301879 0.3710
## 14 240 20 0.30676 0.01720 0.274838 0.3424
## 15 219 16 0.28435 0.01683 0.253204 0.3193
## 16 201 13 0.26596 0.01650 0.235514 0.3003
## 17 187 7 0.25601 0.01630 0.225965 0.2900
## 18 180 12 0.23894 0.01594 0.209646 0.2723
## 19 168 8 0.22756 0.01568 0.198806 0.2605
## 20 159 8 0.21611 0.01541 0.187926 0.2485
## 21 150 12 0.19882 0.01496 0.171557 0.2304
## 22 138 5 0.19162 0.01476 0.164762 0.2229
## 23 133 4 0.18586 0.01460 0.159339 0.2168
## 24 129 7 0.17577 0.01429 0.149873 0.2061
## 25 122 5 0.16857 0.01407 0.143133 0.1985
## 26 117 6 0.15992 0.01378 0.135070 0.1893
## 27 111 2 0.15704 0.01368 0.132388 0.1863
## 28 109 9 0.14407 0.01322 0.120363 0.1725
## 29 100 3 0.13975 0.01305 0.116371 0.1678
## 30 97 7 0.12967 0.01266 0.107088 0.1570
## 31 90 6 0.12102 0.01230 0.099172 0.1477
## 32 83 6 0.11227 0.01191 0.091191 0.1382
## 33 77 6 0.10353 0.01151 0.083257 0.1287
## 34 71 6 0.09478 0.01108 0.075374 0.1192
## 35 65 8 0.08311 0.01045 0.064954 0.1063
## 36 57 3 0.07874 0.01020 0.061077 0.1015
## 37 54 3 0.07436 0.00994 0.057218 0.0966
## 38 51 3 0.06999 0.00967 0.053379 0.0918
## 39 48 4 0.06416 0.00930 0.048293 0.0852
## 40 44 4 0.05832 0.00890 0.043250 0.0787
## 41 40 2 0.05541 0.00869 0.040747 0.0753
## 42 38 2 0.05249 0.00847 0.038256 0.0720
## 43 36 4 0.04666 0.00802 0.033318 0.0653
## 44 32 3 0.04228 0.00765 0.029657 0.0603
## 45 29 3 0.03791 0.00727 0.026039 0.0552
## 46 26 2 0.03499 0.00699 0.023653 0.0518
## 47 24 6 0.02625 0.00609 0.016656 0.0414
## 48 18 2 0.02333 0.00575 0.014390 0.0378
## 49 16 2 0.02041 0.00539 0.012167 0.0342
## 50 14 1 0.01896 0.00520 0.011074 0.0324
## 52 13 1 0.01750 0.00500 0.009996 0.0306
## 53 12 1 0.01604 0.00479 0.008933 0.0288
## 54 11 4 0.01021 0.00383 0.004888 0.0213
## 55 7 2 0.00729 0.00325 0.003046 0.0174
## 57 5 1 0.00583 0.00291 0.002196 0.0155
## 58 4 2 0.00292 0.00206 0.000731 0.0116
## 59 2 2 0.00000 NaN NA NA

modelo3a<-coxph(DBhtatx.survnew~antihta + edad + sexo + raza+ rts + tiempo_qxco + hb + infx + aines + opioid + etev
 + lev + fuma + rts + alcohol + obeso + diabetes + iam +ecv + dolor, data = DBhtatx_filtered)

modelo3a

## Call:
## coxph(formula = DBhtatx.survnew ~ antihta + edad + sexo + raza +
## rts + tiempo_qxco + hb + infx + aines + opioid + etev + lev +
## fuma + rts + alcohol + obeso + diabetes + iam + ecv + dolor,
## data = DBhtatx_filtered)
##
## coef exp(coef) se(coef) z p
## antihtaPrescrito -3.018e-01 7.395e-01 1.490e-01 -2.026 0.042794
## edad 1.302e-05 1.000e+00 2.304e-03 0.006 0.995491
## sexoHombre 2.756e-01 1.317e+00 1.056e-01 2.609 0.009080
## razaNegro 6.213e-01 1.861e+00 3.612e-01 1.720 0.085482
## rts 2.746e-03 1.003e+00 3.638e-03 0.755 0.450254
## tiempo_qxco -2.413e-02 9.762e-01 7.189e-03 -3.357 0.000789
## hb 2.932e-02 1.030e+00 1.934e-02 1.516 0.129534
## infxPresente -5.720e-01 5.644e-01 1.217e-01 -4.700 2.60e-06
## ainesPrescrito -1.315e+00 2.686e-01 7.196e-01 -1.827 0.067740
## opioidPrescrito 2.501e-01 1.284e+00 4.165e-01 0.601 0.548127
## etevPresente -7.083e-01 4.925e-01 4.694e-01 -1.509 0.131348
## lev -1.920e-04 9.998e-01 9.631e-06 -19.936 < 2e-16
## fumaSi 3.006e-01 1.351e+00 8.631e-02 3.482 0.000497
## alcoholConsumo 9.828e-02 1.103e+00 1.047e-01 0.938 0.348003
## obesoPresente -5.580e-02 9.457e-01 1.673e-01 -0.334 0.738670
## diabetesDiabetes -1.093e+00 3.353e-01 2.221e-01 -4.920 8.65e-07
## iamIAM 1.072e+00 2.922e+00 5.880e-01 1.823 0.068269
## ecvECV 4.139e-01 1.513e+00 7.223e-01 0.573 0.566560
## dolor -2.007e-01 8.181e-01 7.710e-02 -2.604 0.009223
##
## Likelihood ratio test=858 on 19 df, p=< 2.2e-16
## n= 647, number of events= 636
## (76 observations deleted due to missingness)

tbl_regression(modelo3a, label = list(antihta~"Antihipertensivos",tiempo_qxco~"Tiempo a cirugía (dias)", sexo~"Sexo",
 raza~"Raza", edad~"Edad (años)", fuma~"Fumador cigarrillo, tabaco o vaper",
 alcohol~"Consumo de licor", obeso~ "Antecedente Obesidad",diabetes~"Diabetes Mellitus tipo 1 o 2",
 iam~"Infarto del miocardio",ecv~ "Enfermedad cerebrovascular",dolor~"Dolor por EVA", hb~"Hemoglobina (gr/dL)", infx ~"Infeccion clinica",
 lev~"Líquidos endovenosos (mL)", rts~"Puntaje de Trauma (RTS)", etev~"Enfermedad tromboembolica venosa"), exponentiate = T, add_estimate_to_reference_rows = T)

## Table printed with {flextable}, not {gt}. Learn why at
## https://www.danieldsjoberg.com/gtsummary/articles/rmarkdown.html
## To suppress this message, include `message = FALSE` in the code chunk header.

| **Characteristic** | **HR**^1^ | **95% CI**^1^ | **p-value** |
| --- | --- | --- | --- |
| Antihipertensivos |  |  |  |
| No prescrito | 1.00 | — |  |
| Prescrito | 0.74 | 0.55, 0.99 | 0.043 |
| Edad (años) | 1.00 | 1.00, 1.00 | >0.9 |
| Sexo |  |  |  |
| Mujer | 1.00 | — |  |
| Hombre | 1.32 | 1.07, 1.62 | 0.009 |
| Raza |  |  |  |
| Mestizo | 1.00 | — |  |
| Negro | 1.86 | 0.92, 3.78 | 0.085 |
| Puntaje de Trauma (RTS) | 1.00 | 1.00, 1.01 | 0.5 |
| Tiempo a cirugía (dias) | 0.98 | 0.96, 0.99 | <0.001 |
| Hemoglobina (gr/dL) | 1.03 | 0.99, 1.07 | 0.13 |
| Infeccion clinica |  |  |  |
| Ausente | 1.00 | — |  |
| Presente | 0.56 | 0.44, 0.72 | <0.001 |
| aines |  |  |  |
| No prescrito | 1.00 | — |  |
| Prescrito | 0.27 | 0.07, 1.10 | 0.068 |
| opioid |  |  |  |
| No prescrito | 1.00 | — |  |
| Prescrito | 1.28 | 0.57, 2.90 | 0.5 |
| Enfermedad tromboembolica venosa |  |  |  |
| Ausente | 1.00 | — |  |
| Presente | 0.49 | 0.20, 1.24 | 0.13 |
| Líquidos endovenosos (mL) | 1.00 | 1.00, 1.00 | <0.001 |
| Fumador cigarrillo, tabaco o vaper |  |  |  |
| No | 1.00 | — |  |
| Si | 1.35 | 1.14, 1.60 | <0.001 |
| Consumo de licor |  |  |  |
| No consumo | 1.00 | — |  |
| Consumo | 1.10 | 0.90, 1.35 | 0.3 |
| Antecedente Obesidad |  |  |  |
| Ausente | 1.00 | — |  |
| Presente | 0.95 | 0.68, 1.31 | 0.7 |
| Diabetes Mellitus tipo 1 o 2 |  |  |  |
| no Diabetes | 1.00 | — |  |
| Diabetes | 0.34 | 0.22, 0.52 | <0.001 |
| Infarto del miocardio |  |  |  |
| no IAM | 1.00 | — |  |
| IAM | 2.92 | 0.92, 9.25 | 0.068 |
| Enfermedad cerebrovascular |  |  |  |
| no ECV | 1.00 | — |  |
| ECV | 1.51 | 0.37, 6.23 | 0.6 |
| Dolor por EVA | 0.82 | 0.70, 0.95 | 0.009 |
| ^1^HR = Hazard Ratio, CI = Confidence Interval | | | |

tab_model(modelo3a, show.se = TRUE, show.stat = TRUE, transform = "exp", show.aic = T, dv.labels = c("Modelo para Riesgo de muerte "))

Modelo para Riesgo de muerte

Predictors

Estimates

std. Error

CI

Statistic

p

antihta [Prescrito]

0.74

0.11

0.55 – 0.99

-2.03

0.043

edad

1.00

0.00

1.00 – 1.00

0.01

0.995

sexo [Hombre]

1.32

0.14

1.07 – 1.62

2.61

0.009

raza [Negro]

1.86

0.67

0.92 – 3.78

1.72

0.085

rts

1.00

0.00

1.00 – 1.01

0.75

0.450

tiempo qxco

0.98

0.01

0.96 – 0.99

-3.36

0.001

hb

1.03

0.02

0.99 – 1.07

1.52

0.130

infx [Presente]

0.56

0.07

0.44 – 0.72

-4.70

<0.001

aines [Prescrito]

0.27

0.19

0.07 – 1.10

-1.83

0.068

opioid [Prescrito]

1.28

0.53

0.57 – 2.90

0.60

0.548

etev [Presente]

0.49

0.23

0.20 – 1.24

-1.51

0.131

lev

1.00

0.00

1.00 – 1.00

-19.94

<0.001

fuma [Si]

1.35

0.12

1.14 – 1.60

3.48

<0.001

alcohol [Consumo]

1.10

0.12

0.90 – 1.35

0.94

0.348

obeso [Presente]

0.95

0.16

0.68 – 1.31

-0.33

0.739

diabetes [Diabetes]

0.34

0.07

0.22 – 0.52

-4.92

<0.001

iam [IAM]

2.92

1.72

0.92 – 9.25

1.82

0.068

ecvECV

1.51

1.09

0.37 – 6.23

0.57

0.567

dolor

0.82

0.06

0.70 – 0.95

-2.60

0.009

Observations

647

R2 Nagelkerke

0.735

AIC

6148.512

survfit(Surv(estancia, altavivo) ~ antihta, DBhtatx_filtered, conf.type = "log-log") %>%
 ggsurvplot(title = "Egreso vivo por prescripcion Antihipertensivo", xlab ="Tiempo (dias)" , ylab = "Probabilidad de Egreso vivo",
 conf.int = T, legend.title = "Antihipertensivo", legend.labs = c("No Prescrito", "Prescrito"))


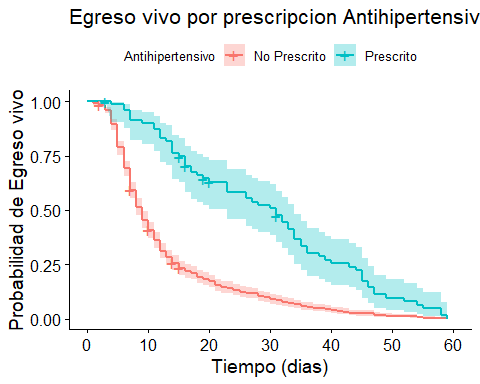


library(survminer)
#sfit <- survfit(DBhtatx.surv~antihta + edad + sexo + raza+ rts + tiempo_qxco + hb + infx + aines + opioid + etev
# + lev, data=DBhtatx)
# sfit
# ggsurvplot(sfit)

# Validacion modelo riesgos
# Chequear multicolinealidad
vif_values <- vif(modelo3)
print(vif_values)

## antihta edad sexo raza rts tiempo_qxco
## 1.180134 1.184993 1.258504 1.024204 1.090389 1.120087
## hb infx aines opioid etev lev
## 1.176594 1.109762 1.028638 1.024046 1.069383 1.570500
## fuma alcohol obeso diabetes iam ecv
## 1.154174 1.244657 1.088876 1.198469 1.019790 1.033345
## dolor
## 1.153822

# Evaluando supuesto de riesgos proporcionales
#Standardize the predictors
predictors <- c("edad", "rts" , "tiempo_qxco" , "hb"
 , "lev") # Replace with your actual predictors
DBhtatx_standardized <- DBhtatx
DBhtatx_standardized[predictors] <- scale(DBhtatx[predictors])
# #
# # # Fit the Cox model
modelo3a <- coxph(DBhtatx.surv ~ antihta + edad + sexo, data = DBhtatx_standardized)
#
tbl_regression(modelo3a, exponentiate = T)

## Table printed with {flextable}, not {gt}. Learn why at
## https://www.danieldsjoberg.com/gtsummary/articles/rmarkdown.html
## To suppress this message, include `message = FALSE` in the code chunk header.

| **Characteristic** | **HR**^1^ | **95% CI**^1^ | **p-value** |
| --- | --- | --- | --- |
| antihta |  |  |  |
| No prescrito | — | — |  |
| Prescrito | 0.31 | 0.24, 0.40 | <0.001 |
| edad | 1.07 | 0.99, 1.16 | 0.090 |
| sexo |  |  |  |
| Mujer | — | — |  |
| Hombre | 0.85 | 0.71, 1.02 | 0.075 |
| ^1^HR = Hazard Ratio, CI = Confidence Interval | | | |

# library(recipes)
# nzv <- nearZeroVar(DBhtatx, saveMetrics = TRUE)
# print(nzv)

missing_values <- sapply(DBhtatx, function(x) sum(is.na(x)))
print(missing_values)

## nombre id sexo raza edad
## 0 0 0 0 1
## ingreso egreso estancia muerte pas
## 0 0 0 0 0
## pad hipot hipotPAM hipot_lev hipot_md
## 0 0 0 0 0
## hipot_vaso antihta clase_med ddd horario_med
## 0 0 1 0 1
## mg_med dias_med ddp freccard frecresp
## 1 0 2 0 0
## glasgow pasrts cRTS rts fractura
## 0 0 0 1 1
## gustillo qxco tiempo_qxco lev aines
## 0 13 13 0 0
## opioid infx etev dolor insomnio
## 0 1 0 0 0
## constipa ansiedad hb creat peso
## 0 0 72 139 0
## fuma alcohol obeso diabetes iam
## 0 0 0 0 0
## ecv Columna43 glasgow_coded pasrts_coded frecresp_coded
## 0 0 11 1 0
## Hipot estancia60 estancianew DDP Hipot_lev
## 0 0 16 657 0
## Hipot_md Hipot_vaso Muerte altavivo
## 0 0 0 0

test.ph <- cox.zph(modelo3)
test.ph

## chisq df p
## antihta 3.03e+00 1 0.08179
## edad 1.06e+01 1 0.00111
## sexo 3.16e+01 1 1.9e-08
## raza 2.29e+00 1 0.13031
## rts 2.05e+00 1 0.15236
## tiempo_qxco 1.66e+01 1 4.7e-05
## hb 1.47e+01 1 0.00013
## infx 1.48e+00 1 0.22330
## aines 2.28e+00 1 0.13093
## opioid 2.97e+00 1 0.08487
## etev 1.81e-03 1 0.96603
## lev 2.03e+02 1 < 2e-16
## fuma 1.34e+01 1 0.00025
## alcohol 2.33e+01 1 1.4e-06
## obeso 5.89e+00 1 0.01524
## diabetes 2.13e+01 1 3.9e-06
## iam 3.46e-01 1 0.55638
## ecv 3.57e-01 1 0.55025
## dolor 3.52e+00 1 0.06081
## GLOBAL 2.39e+02 19 < 2e-16

print(test.ph)

## chisq df p
## antihta 3.03e+00 1 0.08179
## edad 1.06e+01 1 0.00111
## sexo 3.16e+01 1 1.9e-08
## raza 2.29e+00 1 0.13031
## rts 2.05e+00 1 0.15236
## tiempo_qxco 1.66e+01 1 4.7e-05
## hb 1.47e+01 1 0.00013
## infx 1.48e+00 1 0.22330
## aines 2.28e+00 1 0.13093
## opioid 2.97e+00 1 0.08487
## etev 1.81e-03 1 0.96603
## lev 2.03e+02 1 < 2e-16
## fuma 1.34e+01 1 0.00025
## alcohol 2.33e+01 1 1.4e-06
## obeso 5.89e+00 1 0.01524
## diabetes 2.13e+01 1 3.9e-06
## iam 3.46e-01 1 0.55638
## ecv 3.57e-01 1 0.55025
## dolor 3.52e+00 1 0.06081
## GLOBAL 2.39e+02 19 < 2e-16

plot(test.ph)


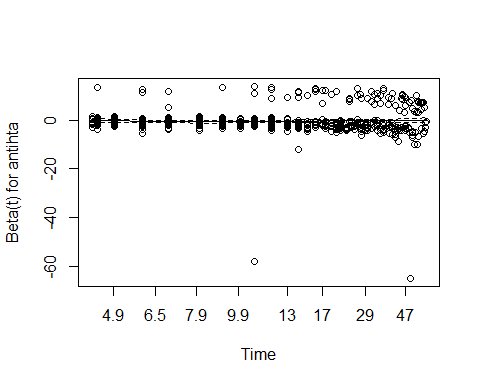

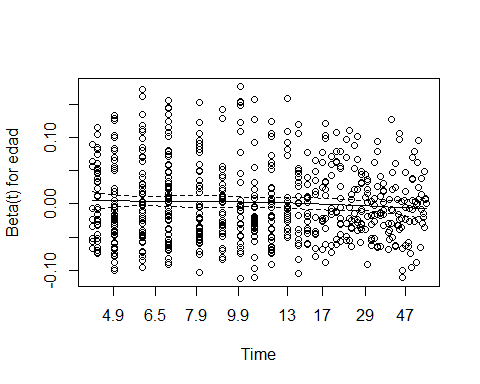

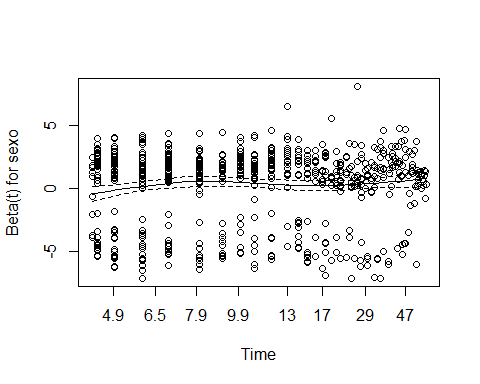

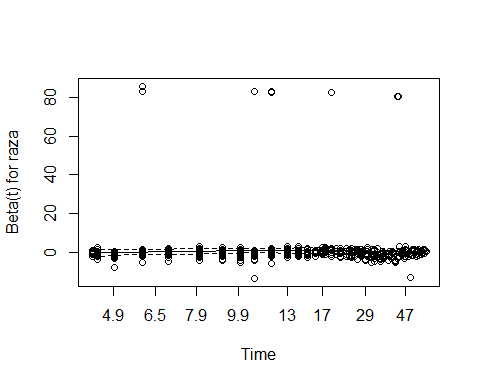

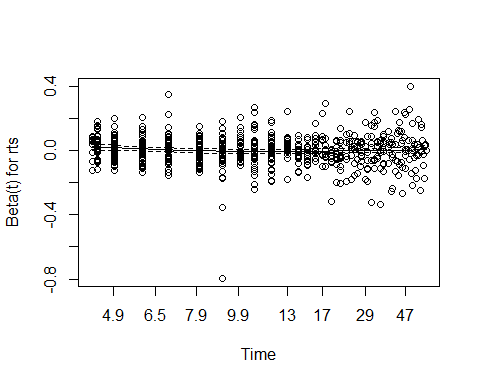

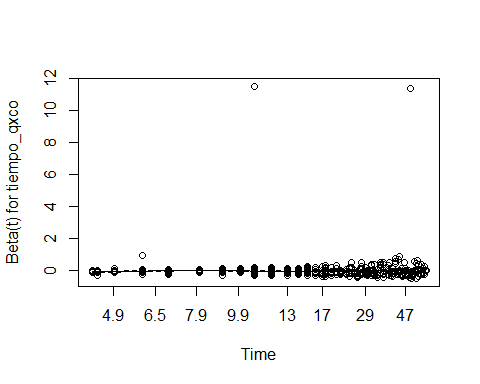

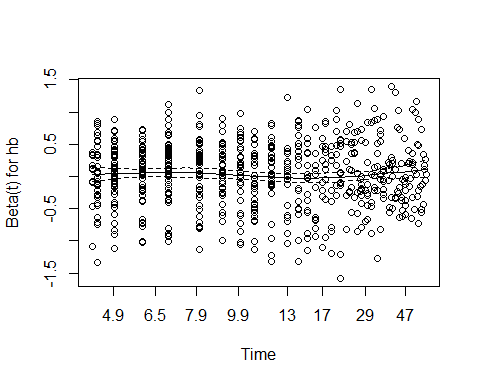

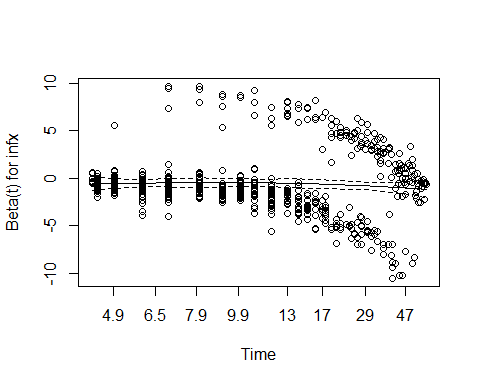

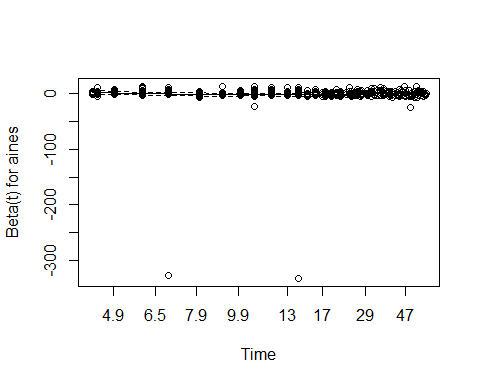

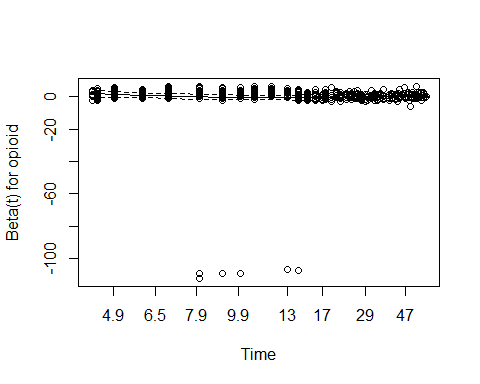

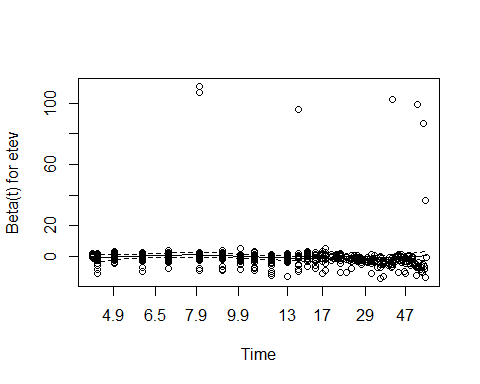

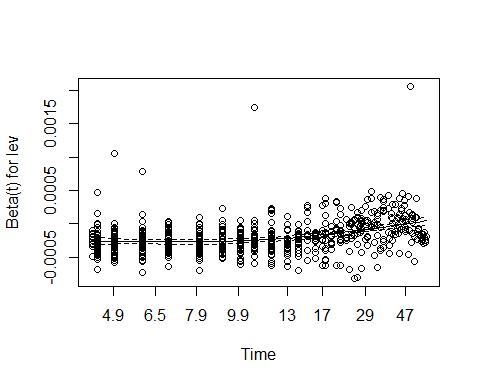

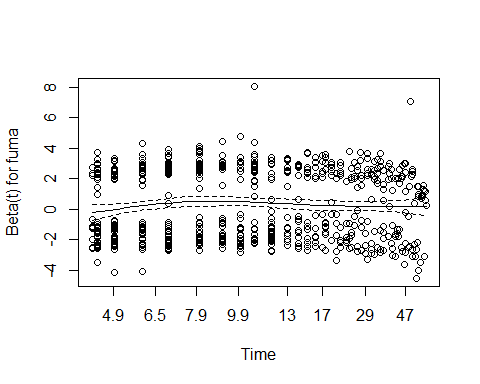

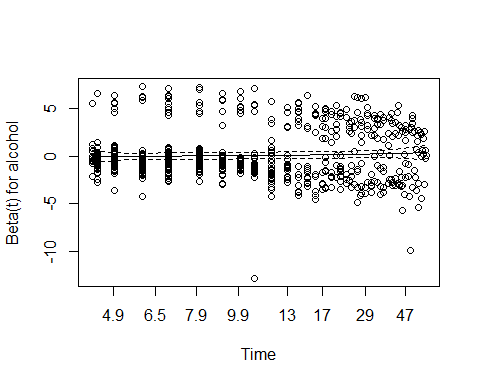

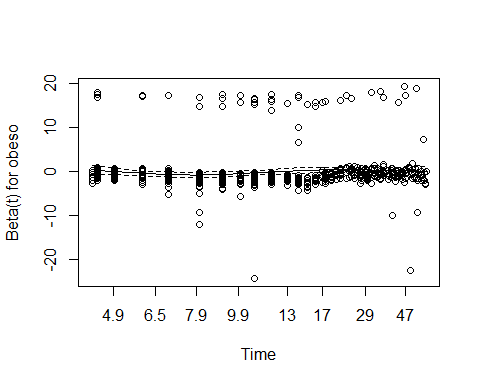

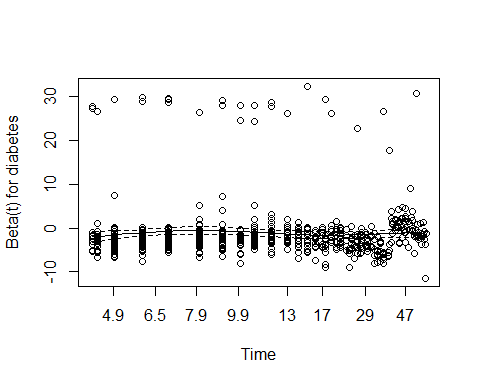

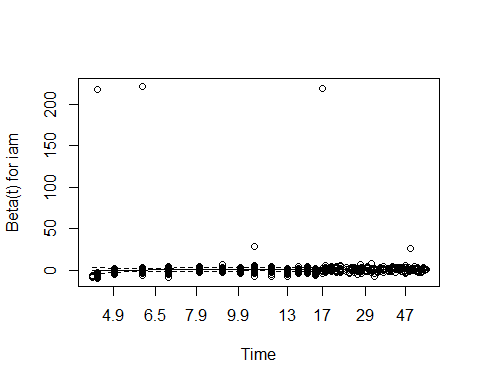

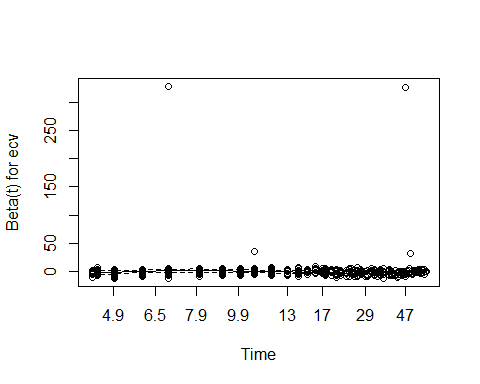

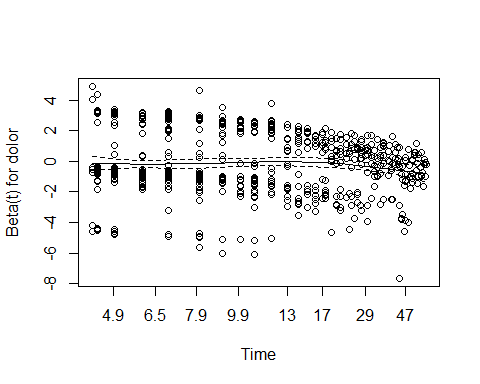


### test influencial y residuales martingala
ggcoxdiagnostics(modelo3, type = , linear.predictions = TRUE)

## `geom_smooth()` using formula = 'y ~ x'


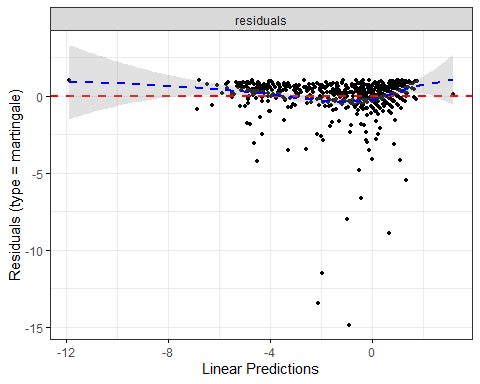


ggcoxdiagnostics(modelo3, type = "dfbeta",
 linear.predictions = FALSE, ggtheme = theme_bw())

## `geom_smooth()` using formula = 'y ~ x'


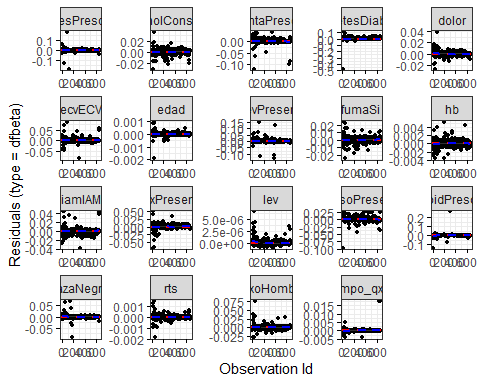


## Para ajustar un modelo de riesgo competitivo--------------------
table(DBhtatx$muerte)

##
## sobrevivio fallecio
## 728 11

table(DBhtatx$altavivo)

##
## 0 1
## 11 728

DBhtatx <- DBhtatx%>%
 mutate(compite = ifelse(muerte == 1, 1, ifelse(altavivo == 1, 2, 0)))

table(DBhtatx$compite)

##
## 0 2
## 11 728

sum(is.na(DBhtatx$compite))

## [1] 0

DBhtatx$compite <- factor(DBhtatx$compite,
 levels = c(1,2),
 labels = c("Muerte", "Alta vivo"))

# Ahora descargamos los paquetes de riesgo competitivo
install.packages("survival")
install.packages("cmprsk")

## Installing package into 'C:/Users/Usuario/AppData/Local/R/win-library/4.3'
## (as 'lib' is unspecified)

## package 'cmprsk' successfully unpacked and MD5 sums checked
##
## The downloaded binary packages are in
## C:\Users\Usuario\AppData\Local\Temp\RtmpYXro44\downloaded_packages

library(survival)
library(cmprsk)

fit <- cuminc(DBhtatx$estancia, DBhtatx$compite, DBhtatx$antihta,
 cencode= 0)

## 11 cases omitted due to missing values

cuminc_fit <- cuminc(DBhtatx$estancia, DBhtatx$compite)

## 11 cases omitted due to missing values

# Print cumulative incidence results
print(cuminc_fit)

## Estimates and Variances:
## $est
## 20 40 60 80 100
## 1 Alta vivo 0.7733516 0.9230769 0.9835165 0.9972527 0.9986264
##
## $var
## 20 40 60 80 100
## 1 Alta vivo 0.0002416313 9.845322e-05 2.317105e-05 4.853875e-06 3.100318e-06

print(fit)

## Tests:
## stat pv df
## Alta vivo 16.99104 3.755656e-05 1
## Estimates and Variances:
## $est
## 20 40 60 80 100
## No prescrito Alta vivo 0.8248848 0.9554531 0.9938556 NA NA
## Prescrito Alta vivo 0.3376623 0.6493506 0.8961039 0.974026 0.987013
##
## $var
## 20 40 60 80
## No prescrito Alta vivo 0.0002229877 6.653183e-05 1.063469e-05 NA
## Prescrito Alta vivo 0.0029517153 3.031142e-03 1.290161e-03 0.000425919
## 100
## No prescrito Alta vivo NA
## Prescrito Alta vivo 0.0002751422

# Plot cumulative incidence functions
plot(cuminc_fit, xlab("Tiempo (dias)"))


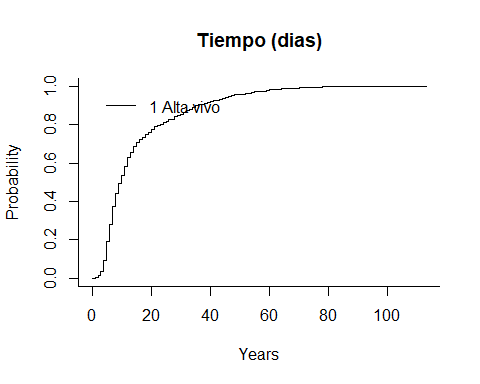


plot(fit, xlab("Incidencia acumulada"))


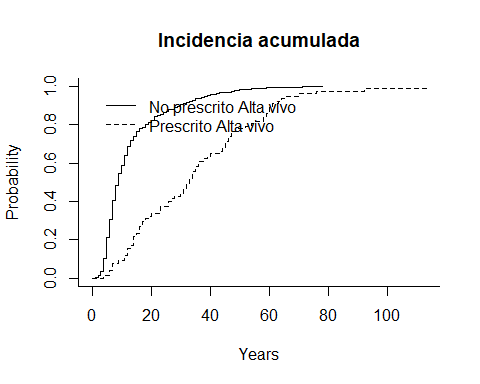


# Antes de ajustar el modelo chequeo diagnostico de datos perdidos

# sum(is.na(DBhtatx))
# sum(is.nan(DBhtatx))
# sum(is.infinite(DBhtatx))

# Remover rows with NA, NaN, or Inf values
# library(stats)
# cleanDBhtatx <- DBhtatx[complete.cases(DBhtatx) &
# !apply(DBhtatx, 1, function(row) any(is.nan(row) | is.infinite(row))), ]
#
# cleanDBhtatx$estancia <- as.numeric(cleanDBhtatx$estancia)
# cleanDBhtatx$compite <- as.numeric(cleanDBhtatx$compite)

# # Fit competing risks regression model
# c("peso","hb","creat","lev","estancia","edad","dolor","raza","pas","pasrts","infx")

# Check if 'estancia' and 'compite' columns exist
if(!all(c("estancia", "compite") %in% names(DBhtatx))) {
 stop("One or both of the columns 'estancia' and 'compite' do not exist in DBhtatx")
}

# Handle missing values (if any)
# DBhtatx <- na.omit(DBhtatx)

# Ensure columns are in correct format (e.g., numeric for time-to-event and factors for event indicators)
DBhtatx$estancia <- as.numeric(DBhtatx$estancia)
DBhtatx$compite <- as.factor(DBhtatx$compite)

# crr_fit <- crr( DBhtatx$estancia, DBhtatx$compite, cov1 = c("peso","hb","creat","lev","estancia","edad","dolor","raza","pas","pasrts","infx"), na.action = na.omit("peso","hb","creat","lev","estancia","edad","dolor","raza","pas","pasrts","infx"))
#
# # # Summary of the fitted model
# summary(fit)
# #
# tbl_regression(fit)
#
# crr


# Modelo 0 de probabilidad de muerte en pacientes expuestod vs no expuestos a antihipertensivos------

modelo0<-glm(Muerte ~ antihta + raza + edad + iam + pas
 + alcohol + obeso + diabetes + ecv + dolor
 + lev+ hb +creat + ansiedad + tiempo_qxco + cRTS + peso + fuma + insomnio + infx + constipa + gustillo
 , data = DBhtatx,
 family = binomial)

modelo0

##
## Call: glm(formula = Muerte ~ antihta + raza + edad + iam + pas + alcohol +
## obeso + diabetes + ecv + dolor + lev + hb + creat + ansiedad +
## tiempo_qxco + cRTS + peso + fuma + insomnio + infx + constipa +
## gustillo, family = binomial, data = DBhtatx)
##
## Coefficients:
## (Intercept) antihtaPrescrito razaNegro edad
## -7.748597 1.546142 -14.971336 0.044163
## iamIAM pas alcoholConsumo obesoPresente
## -16.728633 0.011207 0.887436 1.387541
## diabetesDiabetes ecvECV dolor lev
## 0.292211 -17.575545 -0.464938 -0.000098
## hb creat ansiedadPresente tiempo_qxco
## -0.129697 0.605401 1.833779 -0.096890
## cRTS peso fumaSi insomnioPresente
## 0.628517 -0.028540 0.979745 -0.582927
## infxPresente constipaPresente gustilloGA1 gustilloGA2
## 0.380019 NA -14.672538 1.479970
## gustilloGA3
## 2.379195
##
## Degrees of Freedom: 598 Total (i.e. Null); 575 Residual
## (140 observations deleted due to missingness)
## Null Deviance: 109.7
## Residual Deviance: 70.93 AIC: 118.9

# Figura con OR e IC 95%
tbl_regression(modelo0 , label = list(antihta~"Antihipertensivos",tiempo_qxco~"Tiempo a cirugía (dias)",
 raza~"Raza", edad~"Edad (años)",peso ~"Peso (Kg)", fuma~"Fumador cigarrillo, tabaco o vaper",
 alcohol~"Consumo de licor", obeso~ "Antecedente Obesidad",diabetes~"Diabetes Mellitus tipo 1 o 2",
 iam~"Infarto del miocardio",ecv~ "Enfermedad cerebrovascular",dolor~"Dolor por EVA", insomnio~"Insomnio",
 constipa~"Constipación", ansiedad~"Ansiedad", hb~"Hemoglobina (gr/dL)", infx ~"Infeccion clinica",
 lev~"Líquidos endovenosos (mL)", cRTS~"Puntaje de Trauma (RTS)"), exponentiate = T, add_estimate_to_reference_rows = T)

## Table printed with {flextable}, not {gt}. Learn why at
## https://www.danieldsjoberg.com/gtsummary/articles/rmarkdown.html
## To suppress this message, include `message = FALSE` in the code chunk header.

| **Characteristic** | **OR**^1^ | **95% CI**^1^ | **p-value** |
| --- | --- | --- | --- |
| Antihipertensivos |  |  |  |
| No prescrito | 1.00 | — |  |
| Prescrito | 4.69 | 0.74, 35.1 | 0.11 |
| Raza |  |  |  |
| Mestizo | 1.00 | — |  |
| Negro | 0.00 |  | >0.9 |
| Edad (años) | 1.05 | 1.00, 1.10 | 0.082 |
| Infarto del miocardio |  |  |  |
| no IAM | 1.00 | — |  |
| IAM | 0.00 |  | >0.9 |
| pas | 1.01 | 0.96, 1.06 | 0.7 |
| Consumo de licor |  |  |  |
| No consumo | 1.00 | — |  |
| Consumo | 2.43 | 0.28, 21.6 | 0.4 |
| Antecedente Obesidad |  |  |  |
| Ausente | 1.00 | — |  |
| Presente | 4.00 | 0.19, 74.5 | 0.4 |
| Diabetes Mellitus tipo 1 o 2 |  |  |  |
| no Diabetes | 1.00 | — |  |
| Diabetes | 1.34 | 0.14, 10.3 | 0.8 |
| Enfermedad cerebrovascular |  |  |  |
| no ECV | 1.00 | — |  |
| ECV | 0.00 |  | >0.9 |
| Dolor por EVA | 0.63 | 0.14, 2.86 | 0.5 |
| Líquidos endovenosos (mL) | 1.00 | 1.00, 1.00 | 0.2 |
| Hemoglobina (gr/dL) | 0.88 | 0.62, 1.23 | 0.5 |
| creat | 1.83 | 0.81, 3.17 | 0.038 |
| Ansiedad |  |  |  |
| Ausente | 1.00 | — |  |
| Presente | 6.26 | 1.00, 45.5 | 0.054 |
| Tiempo a cirugía (dias) | 0.91 | 0.73, 1.05 | 0.3 |
| Puntaje de Trauma (RTS) | 1.87 | 0.45, 15.9 | 0.5 |
| Peso (Kg) | 0.97 | 0.89, 1.06 | 0.5 |
| Fumador cigarrillo, tabaco o vaper |  |  |  |
| No | 1.00 | — |  |
| Si | 2.66 | 0.48, 18.2 | 0.3 |
| Insomnio |  |  |  |
| Ausente | 1.00 | — |  |
| Presente | 0.56 | 0.05, 4.89 | 0.6 |
| Infeccion clinica |  |  |  |
| Ausente | 1.00 | — |  |
| Presente | 1.46 | 0.16, 12.9 | 0.7 |
| Constipación |  |  |  |
| Ausente | 1.00 | — |  |
| Presente |  |  |  |
| gustillo |  |  |  |
| Cerrada | 1.00 | — |  |
| GA1 | 0.00 |  | >0.9 |
| GA2 | 4.39 | 0.49, 43.3 | 0.2 |
| GA3 | 10.8 | 1.16, 143 | 0.049 |
| ^1^OR = Odds Ratio, CI = Confidence Interval | | | |

logisticPseudoR2s <- function(modelo0) {
 dev <- modelo0$deviance
 nullDev <- modelo0$null.deviance
 modelN <- length(modelo0$fitted.values)
 R.l <- 1 - dev / nullDev
 R.cs <- 1- exp ( -(nullDev - dev) / modelN)
 R.n <- R.cs / ( 1 - ( exp (-(nullDev / modelN))))
 cat("Pseudo R^2 for logistic regression\n")
 cat("Hosmer and Lemeshow R^2 ", round(R.l, 3), "\n")
 cat("Cox and Snell R^2 ", round(R.cs, 3), "\n")
 cat("Nagelkerke R^2 ", round(R.n, 3), "\n")
}

logisticPseudoR2s(modelo0)

## Pseudo R^2 for logistic regression
## Hosmer and Lemeshow R^2 0.354
## Cox and Snell R^2 0.063
## Nagelkerke R^2 0.375

# Analisis Sensibilidad -------------

# Visualizamos los datos de la columna de las perdidas
DBhtatx$Columna43

## [1] "0" "0" "0" "0"
## [5] "0" "0" "0" "0"
## [9] "0" "0" "0" "0"
## [13] "0" "0" "0" "0"
## [17] "0" "0" "0" "0"
## [21] "0" "0" "0" "0"
## [25] "0" "0" "0" "0"
## [29] "0" "0" "0" "0"
## [33] "0" "0" "0" "0"
## [37] "0" "0" "0" "0"
## [41] "0" "0" "0" "0"
## [45] "0" "0" "0" "0"
## [49] "0" "0" "0" "0"
## [53] "0" "0" "0" "0"
## [57] "0" "0" "0" "0"
## [61] "0" "0" "0" "0"
## [65] "0" "0" "0" "0"
## [69] "0" "0" "0" "0"
## [73] "0" "0" "0" "0"
## [77] "0" "0" "0" "0"
## [81] "0" "0" "0" "0"
## [85] "0" "0" "0" "0"
## [89] "0" "0" "0" "0"
## [93] "0" "0" "0" "0"
## [97] "0" "0" "0" "0"
## [101] "0" "0" "0" "0"
## [105] "0" "0" "0" "0"
## [109] "0" "0" "0" "0"
## [113] "0" "0" "0" "0"
## [117] "0" "0" "0" "0"
## [121] "0" "0" "0" "0"
## [125] "0" "0" "0" "0"
## [129] "0" "0" "0" "0"
## [133] "0" "0" "0" "0"
## [137] "0" "0" "0" "0"
## [141] "0" "0" "0" "0"
## [145] "0" "0" "0" "0"
## [149] "0" "0" "0" "0"
## [153] "0" "0" "0" "0"
## [157] "0" "0" "0" "0"
## [161] "0" "0" "0" "0"
## [165] "0" "0" "0" "0"
## [169] "0" "0" "0" "0"
## [173] "0" "0" "0" "0"
## [177] "0" "0" "0" "0"
## [181] "0" "0" "0" "0"
## [185] "0" "0" "0" "0"
## [189] "0" "0" "0" "0"
## [193] "0" "0" "0" "0"
## [197] "0" "0" "0" "0"
## [201] "0" "0" "0" "0"
## [205] "0" "0" "0" "0"
## [209] "0" "0" "0" "0"
## [213] "0" "0" "0" "0"
## [217] "0" "0" "0" "0"
## [221] "0" "0" "0" "0"
## [225] "0" "0" "0" "0"
## [229] "0" "0" "0" "0"
## [233] "0" "0" "0" "0"
## [237] "0" "0" "0" "0"
## [241] "0" "0" "0" "0"
## [245] "0" "0" "0" "0"
## [249] "0" "0" "0" "0"
## [253] "0" "0" "0" "0"
## [257] "0" "0" "0" "0"
## [261] "0" "0" "0" "0"
## [265] "0" "0" "0" "0"
## [269] "0" "0" "0" "0"
## [273] "0" "0" "0" "0"
## [277] "0" "0" "0" "0"
## [281] "0" "0" "0" "0"
## [285] "0" "0" "0" "0"
## [289] "0" "0" "0" "0"
## [293] "0" "0" "0" "0"
## [297] "0" "0" "0" "0"
## [301] "0" "0" "0" "0"
## [305] "0" "0" "0" "0"
## [309] "0" "0" "0" "0"
## [313] "0" "0" "0" "0"
## [317] "0" "0" "0" "0"
## [321] "0" "0" "0" "0"
## [325] "0" "0" "0" "0"
## [329] "0" "0" "0" "0"
## [333] "0" "0" "0" "0"
## [337] "0" "0" "0" "0"
## [341] "0" "0" "0" "0"
## [345] "0" "0" "0" "0"
## [349] "0" "0" "0" "0"
## [353] "0" "0" "0" "0"
## [357] "0" "0" "0" "0"
## [361] "0" "0" "0" "0"
## [365] "0" "0" "0" "0"
## [369] "0" "0" "0" "0"
## [373] "0" "0" "0" "0"
## [377] "0" "0" "0" "0"
## [381] "0" "0" "0" "0"
## [385] "0" "0" "0" "0"
## [389] "0" "0" "0" "0"
## [393] "0" "0" "0" "0"
## [397] "0" "0" "0" "0"
## [401] "0" "0" "0" "0"
## [405] "0" "0" "0" "0"
## [409] "0" "0" "0" "0"
## [413] "0" "0" "0" "0"
## [417] "0" "0" "0" "0"
## [421] "0" "0" "0" "0"
## [425] "0" "0" "0" "0"
## [429] "0" "0" "0" "0"
## [433] "0" "0" "0" "0"
## [437] "0" "0" "0" "0"
## [441] "0" "0" "0" "0"
## [445] "0" "0" "0" "0"
## [449] "0" "0" "0" "0"
## [453] "0" "0" "0" "0"
## [457] "0" "0" "0" "0"
## [461] "0" "0" "0" "0"
## [465] "0" "0" "0" "0"
## [469] "0" "0" "0" "0"
## [473] "0" "0" "0" "0"
## [477] "0" "0" "0" "0"
## [481] "0" "0" "0" "0"
## [485] "0" "0" "0" "0"
## [489] "0" "0" "0" "0"
## [493] "0" "0" "0" "0"
## [497] "0" "0" "0" "0"
## [501] "0" "0" "0" "0"
## [505] "0" "0" "0" "0"
## [509] "0" "0" "0" "0"
## [513] "0" "0" "0" "0"
## [517] "0" "0" "0" "0"
## [521] "0" "0" "0" "0"
## [525] "0" "0" "0" "0"
## [529] "0" "0" "0" "0"
## [533] "0" "0" "0" "0"
## [537] "0" "0" "0" "0"
## [541] "0" "0" "0" "0"
## [545] "0" "0" "0" "0"
## [549] "0" "0" "0" "0"
## [553] "0" "0" "0" "0"
## [557] "0" "0" "0" "0"
## [561] "0" "0" "0" "0"
## [565] "0" "0" "0" "0"
## [569] "0" "0" "0" "0"
## [573] "0" "0" "0" "0"
## [577] "0" "0" "0" "0"
## [581] "0" "0" "0" "0"
## [585] "0" "0" "0" "0"
## [589] "0" "0" "0" "0"
## [593] "0" "0" "0" "0"
## [597] "0" "0" "0" "0"
## [601] "0" "0" "0" "0"
## [605] "0" "0" "0" "0"
## [609] "0" "0" "0" "0"
## [613] "0" "0" "0" "0"
## [617] "0" "0" "0" "0"
## [621] "0" "0" "0" "0"
## [625] "0" "0" "0" "0"
## [629] "0" "0" "0" "0"
## [633] "0" "0" "0" "0"
## [637] "0" "0" "0" "0"
## [641] "0" "0" "0" "0"
## [645] "0" "0" "0" "0"
## [649] "0" "0" "0" "0"
## [653] "0" "0" "0" "0"
## [657] "0" "0" "0" "0"
## [661] "0" "0" "0" "0"
## [665] "0" "0" "0" "0"
## [669] "0" "0" "0" "0"
## [673] "0" "0" "0" "0"
## [677] "0" "0" "0" "0"
## [681] "0" "0" "0" "0"
## [685] "0" "0" "0" "0"
## [689] "0" "0" "0" "0"
## [693] "0" "0" "0" "0"
## [697] "0" "0" "0" "0"
## [701] "0" "0" "0" "0"
## [705] "0" "0" "0" "0"
## [709] "0" "0" "0" "0"
## [713] "0" "0" "0" "0"
## [717] "0" "0" "0" "0"
## [721] "0" "0" "0" "0"
## [725] "0" "0" "Alta voluntaria" "Remision"
## [729] "Remision" "Remision" "Alta voluntaria" "Alta voluntaria"
## [733] "Remision" "Alta voluntaria" "Alta voluntaria" "Alta voluntaria"
## [737] "Remision" "Remision" "Remision"

# Renombramos como perdidos
DBhtatx <- DBhtatx %>%
 rename("perdidos" = 52)

# visualizamos nuevamente la variable
table(DBhtatx$perdidos)

##
## 0 Alta voluntaria Remision
## 726 6 7

# cambiar a factor 0: sin perdidas 1: perdidas
DBhtatx$perdidos <- factor(ifelse(DBhtatx$perdidos == "0", "0", "1"))

# Visualizamois nueva variable factor
table(DBhtatx$perdidos)

##
## 0 1
## 726 13

# Creamos una nueva base de datos de peor escenario (todos los perdidos hacen hipotension o mueren)

table(DBhtatx$Muerte)

##
## 0 1
## 728 11

table(DBhtatx$Hipot)

##
## presente ausente
## 557 182

peorDB <- DBhtatx %>%
 mutate(Muerte = ifelse(perdidos == "1", 1, Muerte),
 Hipot = ifelse(perdidos == "1", 1, Hipot))

table(peorDB$Muerte)

##
## 0 1
## 715 24

table(peorDB$Hipot)

##
## 1 2
## 557 182

peorDB$Hipot <- factor(ifelse(peorDB$Hipot == "2", "1", "0"))

# Creamos otra nueva base de datos de mejor escenario (todos los perdidos NO hacen hipotension NI mueren)
mejorDB <- DBhtatx %>%
 mutate(Muerte = ifelse(perdidos == "1", 0, Muerte),
 Hipot = ifelse(perdidos == "1", 1, Hipot)) # Hipotension esta codificada como 1 y 2 (no como 0,1)

table(mejorDB$Muerte)

##
## 0 1
## 728 11

table(mejorDB$Hipot) # Hipotension esta codificada como 1 y 2 (no como 0,1)

##
## 1 2
## 557 182

mejorDB$Hipot <- factor(ifelse(mejorDB$Hipot == "2", "1", "0"))

table(mejorDB$Hipot)

##
## 0 1
## 557 182

summary(DBhtatx$estancia) # q1 y q3 para estancia...? pero aqui SI tenemos el dato de estancia

## Min. 1st Qu. Median Mean 3rd Qu. Max.
## 1.00 6.00 10.00 15.38 18.00 113.00

# Restaurar the original dataframe from the backup despues de erroro codificando-------------

# df_backup <- df_original # Crea un respaldo
#ERROR codificando!
# df_original <- df_backup # Restaura la base de datos original despues de codificacion fallida


# Modelo logistico Ade Sensibilidad----
modelo1AS<-glm(Hipot ~ antihta + raza + edad + iam + pas
 + alcohol + obeso + diabetes + ecv + dolor
 + lev+ hb +creat + ansiedad + tiempo_qxco + rts + peso + fuma + insomnio + infx + constipa + gustillo
 , data = peorDB,
 family = binomial)


# tabla del modelo
modelo1AS

##
## Call: glm(formula = Hipot ~ antihta + raza + edad + iam + pas + alcohol +
## obeso + diabetes + ecv + dolor + lev + hb + creat + ansiedad +
## tiempo_qxco + rts + peso + fuma + insomnio + infx + constipa +
## gustillo, family = binomial, data = peorDB)
##
## Coefficients:
## (Intercept) antihtaPrescrito razaNegro edad
## 1.759e+00 2.372e+00 -1.420e+00 1.104e-02
## iamIAM pas alcoholConsumo obesoPresente
## 2.348e-01 1.027e-02 -4.317e-01 7.227e-01
## diabetesDiabetes ecvECV dolor lev
## 5.271e-01 8.556e-02 7.077e-02 3.942e-05
## hb creat ansiedadPresente tiempo_qxco
## -1.838e-01 2.201e-01 8.964e-02 -1.331e-02
## rts peso fumaSi insomnioPresente
## -1.673e-02 -3.186e-02 -9.536e-03 1.209e-01
## infxPresente constipaPresente gustilloGA1 gustilloGA2
## 5.831e-01 NA 7.083e-01 1.922e-01
## gustilloGA3
## 3.922e-01
##
## Degrees of Freedom: 598 Total (i.e. Null); 575 Residual
## (140 observations deleted due to missingness)
## Null Deviance: 721.9
## Residual Deviance: 521.4 AIC: 569.4

# Figura con OR e IC 95%
tbl_regression(modelo1AS , label = list(antihta~"Antihipertensivos",tiempo_qxco~"Tiempo a cirugía (dias)",
 raza~"Raza", edad~"Edad (años)",peso ~"Peso (Kg)", fuma~"Fumador cigarrillo, tabaco o vaper",
 obeso~ "Antecedente Obesidad",diabetes~"Diabetes Mellitus tipo 1 o 2",
 iam~"Infarto del miocardio",ecv~ "Enfermedad cerebrovascular",dolor~"Dolor por EVA", insomnio~"Insomnio",
 constipa~"Constipación", ansiedad~"Ansiedad", hb~"Hemoglobina (gr/dL)", infx ~"Infeccion clinica",
 lev~"Líquidos endovenosos (mL)", rts~"Puntaje de Trauma (RTS)"), exponentiate = T, add_estimate_to_reference_rows = T)

## Table printed with {flextable}, not {gt}. Learn why at
## https://www.danieldsjoberg.com/gtsummary/articles/rmarkdown.html
## To suppress this message, include `message = FALSE` in the code chunk header.

| **Characteristic** | **OR**^1^ | **95% CI**^1^ | **p-value** |
| --- | --- | --- | --- |
| Antihipertensivos |  |  |  |
| No prescrito | 1.00 | — |  |
| Prescrito | 10.7 | 5.15, 23.6 | <0.001 |
| Raza |  |  |  |
| Mestizo | 1.00 | — |  |
| Negro | 0.24 | 0.01, 1.74 | 0.2 |
| Edad (años) | 1.01 | 1.00, 1.02 | 0.10 |
| Infarto del miocardio |  |  |  |
| no IAM | 1.00 | — |  |
| IAM | 1.26 | 0.03, 31.1 | 0.9 |
| pas | 1.01 | 0.99, 1.03 | 0.2 |
| alcohol |  |  |  |
| No consumo | 1.00 | — |  |
| Consumo | 0.65 | 0.36, 1.15 | 0.15 |
| Antecedente Obesidad |  |  |  |
| Ausente | 1.00 | — |  |
| Presente | 2.06 | 0.72, 5.84 | 0.2 |
| Diabetes Mellitus tipo 1 o 2 |  |  |  |
| no Diabetes | 1.00 | — |  |
| Diabetes | 1.69 | 0.60, 4.54 | 0.3 |
| Enfermedad cerebrovascular |  |  |  |
| no ECV | 1.00 | — |  |
| ECV | 1.09 | 0.03, 37.2 | >0.9 |
| Dolor por EVA | 1.07 | 0.70, 1.66 | 0.7 |
| Líquidos endovenosos (mL) | 1.00 | 1.00, 1.00 | 0.057 |
| Hemoglobina (gr/dL) | 0.83 | 0.75, 0.92 | <0.001 |
| creat | 1.25 | 0.86, 1.79 | 0.2 |
| Ansiedad |  |  |  |
| Ausente | 1.00 | — |  |
| Presente | 1.09 | 0.59, 1.99 | 0.8 |
| Tiempo a cirugía (dias) | 0.99 | 0.95, 1.00 | 0.4 |
| Puntaje de Trauma (RTS) | 0.98 | 0.97, 1.00 | 0.069 |
| Peso (Kg) | 0.97 | 0.94, 1.00 | 0.023 |
| Fumador cigarrillo, tabaco o vaper |  |  |  |
| No | 1.00 | — |  |
| Si | 0.99 | 0.62, 1.57 | >0.9 |
| Insomnio |  |  |  |
| Ausente | 1.00 | — |  |
| Presente | 1.13 | 0.65, 1.95 | 0.7 |
| Infeccion clinica |  |  |  |
| Ausente | 1.00 | — |  |
| Presente | 1.79 | 0.99, 3.24 | 0.054 |
| Constipación |  |  |  |
| Ausente | 1.00 | — |  |
| Presente |  |  |  |
| gustillo |  |  |  |
| Cerrada | 1.00 | — |  |
| GA1 | 2.03 | 1.05, 3.92 | 0.035 |
| GA2 | 1.21 | 0.62, 2.34 | 0.6 |
| GA3 | 1.48 | 0.77, 2.85 | 0.2 |
| ^1^OR = Odds Ratio, CI = Confidence Interval | | | |

# Modelo riesgo compite de alta vivo A. de sensibilidad----
modelo3as<-coxph(DBhtatx.survnew~antihta + edad + sexo + raza+ rts + tiempo_qxco + hb + infx + aines + opioid + etev
 + lev + fuma + rts + alcohol + obeso + diabetes + iam +ecv + dolor, data = DBhtatx_filtered)
# tabla del modelo
modelo3as

## Call:
## coxph(formula = DBhtatx.survnew ~ antihta + edad + sexo + raza +
## rts + tiempo_qxco + hb + infx + aines + opioid + etev + lev +
## fuma + rts + alcohol + obeso + diabetes + iam + ecv + dolor,
## data = DBhtatx_filtered)
##
## coef exp(coef) se(coef) z p
## antihtaPrescrito -3.018e-01 7.395e-01 1.490e-01 -2.026 0.042794
## edad 1.302e-05 1.000e+00 2.304e-03 0.006 0.995491
## sexoHombre 2.756e-01 1.317e+00 1.056e-01 2.609 0.009080
## razaNegro 6.213e-01 1.861e+00 3.612e-01 1.720 0.085482
## rts 2.746e-03 1.003e+00 3.638e-03 0.755 0.450254
## tiempo_qxco -2.413e-02 9.762e-01 7.189e-03 -3.357 0.000789
## hb 2.932e-02 1.030e+00 1.934e-02 1.516 0.129534
## infxPresente -5.720e-01 5.644e-01 1.217e-01 -4.700 2.60e-06
## ainesPrescrito -1.315e+00 2.686e-01 7.196e-01 -1.827 0.067740
## opioidPrescrito 2.501e-01 1.284e+00 4.165e-01 0.601 0.548127
## etevPresente -7.083e-01 4.925e-01 4.694e-01 -1.509 0.131348
## lev -1.920e-04 9.998e-01 9.631e-06 -19.936 < 2e-16
## fumaSi 3.006e-01 1.351e+00 8.631e-02 3.482 0.000497
## alcoholConsumo 9.828e-02 1.103e+00 1.047e-01 0.938 0.348003
## obesoPresente -5.580e-02 9.457e-01 1.673e-01 -0.334 0.738670
## diabetesDiabetes -1.093e+00 3.353e-01 2.221e-01 -4.920 8.65e-07
## iamIAM 1.072e+00 2.922e+00 5.880e-01 1.823 0.068269
## ecvECV 4.139e-01 1.513e+00 7.223e-01 0.573 0.566560
## dolor -2.007e-01 8.181e-01 7.710e-02 -2.604 0.009223
##
## Likelihood ratio test=858 on 19 df, p=< 2.2e-16
## n= 647, number of events= 636
## (76 observations deleted due to missingness)

tbl_regression(modelo3as, label = list(antihta~"Antihipertensivos",tiempo_qxco~"Tiempo a cirugía (dias)", sexo~"Sexo",
 raza~"Raza", edad~"Edad (años)", fuma~"Fumador cigarrillo, tabaco o vaper",
 alcohol~"Consumo de licor", obeso~ "Antecedente Obesidad",diabetes~"Diabetes Mellitus tipo 1 o 2",
 iam~"Infarto del miocardio",ecv~ "Enfermedad cerebrovascular",dolor~"Dolor por EVA", hb~"Hemoglobina (gr/dL)", infx ~"Infeccion clinica",
 lev~"Líquidos endovenosos (mL)", rts~"Puntaje de Trauma (RTS)", etev~"Enfermedad tromboembolica venosa"), exponentiate = T, add_estimate_to_reference_rows = T)

## Table printed with {flextable}, not {gt}. Learn why at
## https://www.danieldsjoberg.com/gtsummary/articles/rmarkdown.html
## To suppress this message, include `message = FALSE` in the code chunk header.

| **Characteristic** | **HR**^1^ | **95% CI**^1^ | **p-value** |
| --- | --- | --- | --- |
| Antihipertensivos |  |  |  |
| No prescrito | 1.00 | — |  |
| Prescrito | 0.74 | 0.55, 0.99 | 0.043 |
| Edad (años) | 1.00 | 1.00, 1.00 | >0.9 |
| Sexo |  |  |  |
| Mujer | 1.00 | — |  |
| Hombre | 1.32 | 1.07, 1.62 | 0.009 |
| Raza |  |  |  |
| Mestizo | 1.00 | — |  |
| Negro | 1.86 | 0.92, 3.78 | 0.085 |
| Puntaje de Trauma (RTS) | 1.00 | 1.00, 1.01 | 0.5 |
| Tiempo a cirugía (dias) | 0.98 | 0.96, 0.99 | <0.001 |
| Hemoglobina (gr/dL) | 1.03 | 0.99, 1.07 | 0.13 |
| Infeccion clinica |  |  |  |
| Ausente | 1.00 | — |  |
| Presente | 0.56 | 0.44, 0.72 | <0.001 |
| aines |  |  |  |
| No prescrito | 1.00 | — |  |
| Prescrito | 0.27 | 0.07, 1.10 | 0.068 |
| opioid |  |  |  |
| No prescrito | 1.00 | — |  |
| Prescrito | 1.28 | 0.57, 2.90 | 0.5 |
| Enfermedad tromboembolica venosa |  |  |  |
| Ausente | 1.00 | — |  |
| Presente | 0.49 | 0.20, 1.24 | 0.13 |
| Líquidos endovenosos (mL) | 1.00 | 1.00, 1.00 | <0.001 |
| Fumador cigarrillo, tabaco o vaper |  |  |  |
| No | 1.00 | — |  |
| Si | 1.35 | 1.14, 1.60 | <0.001 |
| Consumo de licor |  |  |  |
| No consumo | 1.00 | — |  |
| Consumo | 1.10 | 0.90, 1.35 | 0.3 |
| Antecedente Obesidad |  |  |  |
| Ausente | 1.00 | — |  |
| Presente | 0.95 | 0.68, 1.31 | 0.7 |
| Diabetes Mellitus tipo 1 o 2 |  |  |  |
| no Diabetes | 1.00 | — |  |
| Diabetes | 0.34 | 0.22, 0.52 | <0.001 |
| Infarto del miocardio |  |  |  |
| no IAM | 1.00 | — |  |
| IAM | 2.92 | 0.92, 9.25 | 0.068 |
| Enfermedad cerebrovascular |  |  |  |
| no ECV | 1.00 | — |  |
| ECV | 1.51 | 0.37, 6.23 | 0.6 |
| Dolor por EVA | 0.82 | 0.70, 0.95 | 0.009 |
| ^1^HR = Hazard Ratio, CI = Confidence Interval | | | |

# Modelo logistico de muerte A de sensibilidad----
modelo0AS<-glm(Muerte ~ antihta + raza + edad + iam + pas
 + alcohol + obeso + diabetes + ecv + dolor
 + lev+ hb +creat + ansiedad + tiempo_qxco + cRTS + peso + fuma + insomnio + infx + constipa + gustillo
 , data = peorDB,
 family = binomial)
# tabla del modelo
modelo0AS

##
## Call: glm(formula = Muerte ~ antihta + raza + edad + iam + pas + alcohol +
## obeso + diabetes + ecv + dolor + lev + hb + creat + ansiedad +
## tiempo_qxco + cRTS + peso + fuma + insomnio + infx + constipa +
## gustillo, family = binomial, data = peorDB)
##
## Coefficients:
## (Intercept) antihtaPrescrito razaNegro edad
## -7.748597 1.546142 -14.971336 0.044163
## iamIAM pas alcoholConsumo obesoPresente
## -16.728633 0.011207 0.887436 1.387541
## diabetesDiabetes ecvECV dolor lev
## 0.292211 -17.575545 -0.464938 -0.000098
## hb creat ansiedadPresente tiempo_qxco
## -0.129697 0.605401 1.833779 -0.096890
## cRTS peso fumaSi insomnioPresente
## 0.628517 -0.028540 0.979745 -0.582927
## infxPresente constipaPresente gustilloGA1 gustilloGA2
## 0.380019 NA -14.672538 1.479970
## gustilloGA3
## 2.379195
##
## Degrees of Freedom: 598 Total (i.e. Null); 575 Residual
## (140 observations deleted due to missingness)
## Null Deviance: 109.7
## Residual Deviance: 70.93 AIC: 118.9

# Figura con OR e IC 95%
tbl_regression(modelo0AS , label = list(antihta~"Antihipertensivos",tiempo_qxco~"Tiempo a cirugía (dias)",
 raza~"Raza", edad~"Edad (años)",peso ~"Peso (Kg)", fuma~"Fumador cigarrillo, tabaco o vaper",
 alcohol~"Consumo de licor", obeso~ "Antecedente Obesidad",diabetes~"Diabetes Mellitus tipo 1 o 2",
 iam~"Infarto del miocardio",ecv~ "Enfermedad cerebrovascular",dolor~"Dolor por EVA", insomnio~"Insomnio",
 constipa~"Constipación", ansiedad~"Ansiedad", hb~"Hemoglobina (gr/dL)", infx ~"Infeccion clinica",
 lev~"Líquidos endovenosos (mL)", cRTS~"Puntaje de Trauma (RTS)"), exponentiate = T, add_estimate_to_reference_rows = T)

## Table printed with {flextable}, not {gt}. Learn why at
## https://www.danieldsjoberg.com/gtsummary/articles/rmarkdown.html
## To suppress this message, include `message = FALSE` in the code chunk header.

| **Characteristic** | **OR**^1^ | **95% CI**^1^ | **p-value** |
| --- | --- | --- | --- |
| Antihipertensivos |  |  |  |
| No prescrito | 1.00 | — |  |
| Prescrito | 4.69 | 0.74, 35.1 | 0.11 |
| Raza |  |  |  |
| Mestizo | 1.00 | — |  |
| Negro | 0.00 |  | >0.9 |
| Edad (años) | 1.05 | 1.00, 1.10 | 0.082 |
| Infarto del miocardio |  |  |  |
| no IAM | 1.00 | — |  |
| IAM | 0.00 |  | >0.9 |
| pas | 1.01 | 0.96, 1.06 | 0.7 |
| Consumo de licor |  |  |  |
| No consumo | 1.00 | — |  |
| Consumo | 2.43 | 0.28, 21.6 | 0.4 |
| Antecedente Obesidad |  |  |  |
| Ausente | 1.00 | — |  |
| Presente | 4.00 | 0.19, 74.5 | 0.4 |
| Diabetes Mellitus tipo 1 o 2 |  |  |  |
| no Diabetes | 1.00 | — |  |
| Diabetes | 1.34 | 0.14, 10.3 | 0.8 |
| Enfermedad cerebrovascular |  |  |  |
| no ECV | 1.00 | — |  |
| ECV | 0.00 |  | >0.9 |
| Dolor por EVA | 0.63 | 0.14, 2.86 | 0.5 |
| Líquidos endovenosos (mL) | 1.00 | 1.00, 1.00 | 0.2 |
| Hemoglobina (gr/dL) | 0.88 | 0.62, 1.23 | 0.5 |
| creat | 1.83 | 0.81, 3.17 | 0.038 |
| Ansiedad |  |  |  |
| Ausente | 1.00 | — |  |
| Presente | 6.26 | 1.00, 45.5 | 0.054 |
| Tiempo a cirugía (dias) | 0.91 | 0.73, 1.05 | 0.3 |
| Puntaje de Trauma (RTS) | 1.87 | 0.45, 15.9 | 0.5 |
| Peso (Kg) | 0.97 | 0.89, 1.06 | 0.5 |
| Fumador cigarrillo, tabaco o vaper |  |  |  |
| No | 1.00 | — |  |
| Si | 2.66 | 0.48, 18.2 | 0.3 |
| Insomnio |  |  |  |
| Ausente | 1.00 | — |  |
| Presente | 0.56 | 0.05, 4.89 | 0.6 |
| Infeccion clinica |  |  |  |
| Ausente | 1.00 | — |  |
| Presente | 1.46 | 0.16, 12.9 | 0.7 |
| Constipación |  |  |  |
| Ausente | 1.00 | — |  |
| Presente |  |  |  |
| gustillo |  |  |  |
| Cerrada | 1.00 | — |  |
| GA1 | 0.00 |  | >0.9 |
| GA2 | 4.39 | 0.49, 43.3 | 0.2 |
| GA3 | 10.8 | 1.16, 143 | 0.049 |
| ^1^OR = Odds Ratio, CI = Confidence Interval | | | |

# Tabla 1 Analisis sensibilidad--------
missDB <- DBhtatx |> slice(727:739)

View(missDB)

missDB %>% dplyr:::select (
 !c(
 nombre,
 id,
 ingreso,
 egreso,
 muerte,
 hipot,
 Hipot,
 estancia,
 hipotPAM,
 hipot_lev,
 hipot_md,
 hipot_vaso,
 qxco,
 Hipot_lev,
 Hipot_md,
 Hipot_vaso,
 Muerte,
 altavivo,
 compite,
 clase_med,
 ddd,
 horario_med,
 mg_med,
 ddp,
 clase_med,
 dias_med,
 dias_med,
 pas,
 pad,
 lev,
 aines,
 opioid,
 infx,
 etev,
 dolor,
 insomnio,
 constipa,
 ansiedad,
 hb ,
 creat,
 tiempo_qxco,
 rts,
 glasgow_coded,
 pasrts_coded,
 frecresp_coded,
 freccard
 )
) %>% tbl_summary(
 by = antihta,
 label = list(
 sexo ~ "Sexo",
 raza ~ "Raza",
 edad ~ "Edad (años)",
 cRTS ~
 "Puntaje de Trauma (RTS)",
 fractura ~ "Hueso fracturado",
 gustillo ~ "Clasificacion Gustillo Anderson (GA)",
 peso ~
 "Peso (Kg)",
 fuma ~ "Fumador cigarrillo, tabaco o vaper",
 alcohol ~ "Consumo de licor",
 obeso ~ "Antecedente Obesidad",
 iam ~ "Infarto del miocardio",
 diabetes ~
 "Diabetes Mellitus tipo 1 o 2",
 ecv ~ "Enfermedad cerebrovascular",
 frecresp ~ "Frecuencia respiratoria al ingreso (rpm)",
 glasgow ~ "Escala coma Glasgow (pts.)",
 pasrts ~
 "PAS al ingreso (mmHg)"
 ),
 value = list(
 sexo ~ "Hombre",
 raza ~ "Mestizo",
 fuma ~
 "Si",
 alcohol ~ "Consumo",
 obeso ~ "Presente",
 iam ~ "IAM",
 diabetes ~
 "Diabetes",
 ecv ~ "ECV"
 ),
 missing = "always"
) %>% add_overall()

## Table printed with {flextable}, not {gt}. Learn why at
## https://www.danieldsjoberg.com/gtsummary/articles/rmarkdown.html
## To suppress this message, include `message = FALSE` in the code chunk header.

| **Characteristic** | **Overall**, N = 13^1^ | **No prescrito**, N = 13^1^ | **Prescrito**, N = 0^1^ |
| --- | --- | --- | --- |
| Sexo | 10 (77%) | 10 (77%) | 0 (NA%) |
| Unknown | 0 | 0 | 0 |
| Raza | 13 (100%) | 13 (100%) | 0 (NA%) |
| Unknown | 0 | 0 | 0 |
| Edad (años) | 28 (24, 36) | 28 (24, 36) | NA (NA, NA) |
| Unknown | 0 | 0 | 0 |
| Frecuencia respiratoria al ingreso (rpm) |  |  |  |
| 18 | 1 (7.7%) | 1 (7.7%) | 0 (NA%) |
| 19.25 | 1 (7.7%) | 1 (7.7%) | 0 (NA%) |
| 19.5 | 1 (7.7%) | 1 (7.7%) | 0 (NA%) |
| 20 | 1 (7.7%) | 1 (7.7%) | 0 (NA%) |
| 22 | 1 (7.7%) | 1 (7.7%) | 0 (NA%) |
| 22.25 | 2 (15%) | 2 (15%) | 0 (NA%) |
| 22.5 | 2 (15%) | 2 (15%) | 0 (NA%) |
| 23 | 3 (23%) | 3 (23%) | 0 (NA%) |
| 26.25 | 1 (7.7%) | 1 (7.7%) | 0 (NA%) |
| Unknown | 0 | 0 | 0 |
| Escala coma Glasgow (pts.) |  |  |  |
| 15 | 13 (100%) | 13 (100%) | 0 (NA%) |
| Unknown | 0 | 0 | 0 |
| PAS al ingreso (mmHg) | 119.0 (118.0, 122.0) | 119.0 (118.0, 122.0) | NA (NA, NA) |
| Unknown | 0 | 0 | 0 |
| Puntaje de Trauma (RTS) |  |  |  |
| 3.6874 | 13 (100%) | 13 (100%) | 0 (NA%) |
| Unknown | 0 | 0 | 0 |
| Hueso fracturado |  |  |  |
| Clavicula | 0 (0%) | 0 (0%) | 0 (NA%) |
| Hombro | 0 (0%) | 0 (0%) | 0 (NA%) |
| Humero | 0 (0%) | 0 (0%) | 0 (NA%) |
| Codo | 0 (0%) | 0 (0%) | 0 (NA%) |
| Radio o cubito | 5 (38%) | 5 (38%) | 0 (NA%) |
| Muneca | 0 (0%) | 0 (0%) | 0 (NA%) |
| Mano | 0 (0%) | 0 (0%) | 0 (NA%) |
| Pelvis | 0 (0%) | 0 (0%) | 0 (NA%) |
| Cadera | 1 (7.7%) | 1 (7.7%) | 0 (NA%) |
| Femur | 2 (15%) | 2 (15%) | 0 (NA%) |
| Rodilla | 0 (0%) | 0 (0%) | 0 (NA%) |
| Tibia - perone | 3 (23%) | 3 (23%) | 0 (NA%) |
| Tobillo | 1 (7.7%) | 1 (7.7%) | 0 (NA%) |
| Pie | 1 (7.7%) | 1 (7.7%) | 0 (NA%) |
| Unknown | 0 | 0 | 0 |
| Clasificacion Gustillo Anderson (GA) |  |  |  |
| Cerrada | 10 (77%) | 10 (77%) | 0 (NA%) |
| GA1 | 2 (15%) | 2 (15%) | 0 (NA%) |
| GA2 | 1 (7.7%) | 1 (7.7%) | 0 (NA%) |
| GA3 | 0 (0%) | 0 (0%) | 0 (NA%) |
| Unknown | 0 | 0 | 0 |
| Peso (Kg) |  |  |  |
| 57.0001 | 1 (7.7%) | 1 (7.7%) | 0 (NA%) |
| 60.0001 | 1 (7.7%) | 1 (7.7%) | 0 (NA%) |
| 61.0001 | 1 (7.7%) | 1 (7.7%) | 0 (NA%) |
| 67.0001 | 1 (7.7%) | 1 (7.7%) | 0 (NA%) |
| 69.0001 | 1 (7.7%) | 1 (7.7%) | 0 (NA%) |
| 69.1234859397418 | 7 (54%) | 7 (54%) | 0 (NA%) |
| 78.0001 | 1 (7.7%) | 1 (7.7%) | 0 (NA%) |
| Unknown | 0 | 0 | 0 |
| Fumador cigarrillo, tabaco o vaper | 6 (46%) | 6 (46%) | 0 (NA%) |
| Unknown | 0 | 0 | 0 |
| Consumo de licor | 0 (0%) | 0 (0%) | 0 (NA%) |
| Unknown | 0 | 0 | 0 |
| Antecedente Obesidad | 0 (0%) | 0 (0%) | 0 (NA%) |
| Unknown | 0 | 0 | 0 |
| Diabetes Mellitus tipo 1 o 2 | 0 (0%) | 0 (0%) | 0 (NA%) |
| Unknown | 0 | 0 | 0 |
| Infarto del miocardio | 0 (0%) | 0 (0%) | 0 (NA%) |
| Unknown | 0 | 0 | 0 |
| Enfermedad cerebrovascular | 0 (0%) | 0 (0%) | 0 (NA%) |
| Unknown | 0 | 0 | 0 |
| perdidos |  |  |  |
| 0 | 0 (0%) | 0 (0%) | 0 (NA%) |
| 1 | 13 (100%) | 13 (100%) | 0 (NA%) |
| Unknown | 0 | 0 | 0 |
| estancia60 | 13 (100%) | 13 (100%) | 0 (NA%) |
| Unknown | 0 | 0 | 0 |
| estancianew |  |  |  |
| 1 | 4 (31%) | 4 (31%) | 0 (NA%) |
| 2 | 5 (38%) | 5 (38%) | 0 (NA%) |
| 3 | 2 (15%) | 2 (15%) | 0 (NA%) |
| 5 | 1 (7.7%) | 1 (7.7%) | 0 (NA%) |
| 6 | 1 (7.7%) | 1 (7.7%) | 0 (NA%) |
| Unknown | 0 | 0 | 0 |
| DDP | NA (NA, NA) | NA (NA, NA) | NA (NA, NA) |
| Unknown | 13 | 13 | 0 |
| ^1^n (%); Median (IQR) | | | |

##------------
missDB%>% select (
 !c(sexo, raza, edad, rts,peso, glasgow,pasrts,freccard,
 nombre,fuma,diabetes,iam,obeso,ecv,alcohol,
 id,Hipot_lev, Hipot_md,Hipot_vaso, Muerte, altavivo, compite,
 ingreso,
 egreso,
 muerte,
 hipot, Hipot,
 estancia,
 hipotPAM,
 hipot_lev,
 hipot_md,
 hipot_vaso,
 qxco,
 ddd,
 mg_med,
 ddp,
 fractura,
 gustillo,glasgow_coded,pasrts_coded,frecresp,frecresp_coded,cRTS
 )
) %>% tbl_summary(
 by = antihta,
 label = list(
 clase_med~ "Medicamento antihipertensivo",
 horario_med~ "Horario administracion",
 dias_med ~ "Dias de medicamento prescrito",
 pas ~ "Presión arterial sistólica (mmHg)",
 pad ~ "Presión arterial diastólica (mmHg)",
 tiempo_qxco ~ "Tiempo a cirugía (dias)",
 lev ~
 "Líquidos endovenosos durante hospitalización (mL)",
 aines ~ "Antinflamatorios",
 opioid ~ "Opioides",
 infx ~ "Infeccion clinica",
 etev ~ "Enfermedad tromboembolica",
 dolor ~
 "Dolor por EVA",
 insomnio ~ "Insomnio",
 constipa ~ "Constipacion",
 ansiedad ~ "Ansiedad",
 hb ~ "Hemoglobina (gr/dL)",
 creat ~ "Creatinina (mg/dL)"
 ),
 value = list(
 opioid ~ "Prescrito",
 infx ~ "Presente",
 etev ~ "Presente",
 insomnio ~
 "Presente",
 constipa ~ "Presente",
 ansiedad ~ "Presente",
 aines ~
 "Prescrito"
 ),
 missing = "always"
) %>% add_overall()

## Table printed with {flextable}, not {gt}. Learn why at
## https://www.danieldsjoberg.com/gtsummary/articles/rmarkdown.html
## To suppress this message, include `message = FALSE` in the code chunk header.

| **Characteristic** | **Overall**, N = 13^1^ | **No prescrito**, N = 13^1^ | **Prescrito**, N = 0^1^ |
| --- | --- | --- | --- |
| Presión arterial sistólica (mmHg) |  |  |  |
| 122 | 1 (7.7%) | 1 (7.7%) | 0 (NA%) |
| 126 | 1 (7.7%) | 1 (7.7%) | 0 (NA%) |
| 128 | 4 (31%) | 4 (31%) | 0 (NA%) |
| 129 | 1 (7.7%) | 1 (7.7%) | 0 (NA%) |
| 130 | 2 (15%) | 2 (15%) | 0 (NA%) |
| 132 | 2 (15%) | 2 (15%) | 0 (NA%) |
| 138 | 1 (7.7%) | 1 (7.7%) | 0 (NA%) |
| 139 | 1 (7.7%) | 1 (7.7%) | 0 (NA%) |
| Unknown | 0 | 0 | 0 |
| Presión arterial diastólica (mmHg) |  |  |  |
| 79 | 5 (38%) | 5 (38%) | 0 (NA%) |
| 80 | 1 (7.7%) | 1 (7.7%) | 0 (NA%) |
| 83 | 2 (15%) | 2 (15%) | 0 (NA%) |
| 87 | 1 (7.7%) | 1 (7.7%) | 0 (NA%) |
| 90 | 1 (7.7%) | 1 (7.7%) | 0 (NA%) |
| 92 | 2 (15%) | 2 (15%) | 0 (NA%) |
| 96 | 1 (7.7%) | 1 (7.7%) | 0 (NA%) |
| Unknown | 0 | 0 | 0 |
| Medicamento antihipertensivo |  |  |  |
| noMd | 13 (100%) | 13 (100%) | 0 (NA%) |
| Losartan | 0 (0%) | 0 (0%) | 0 (NA%) |
| Enalapril | 0 (0%) | 0 (0%) | 0 (NA%) |
| Amlodipino | 0 (0%) | 0 (0%) | 0 (NA%) |
| Metoprolol | 0 (0%) | 0 (0%) | 0 (NA%) |
| Hidroclorotiazida | 0 (0%) | 0 (0%) | 0 (NA%) |
| Prazosin | 0 (0%) | 0 (0%) | 0 (NA%) |
| Clonidina | 0 (0%) | 0 (0%) | 0 (NA%) |
| Unknown | 0 | 0 | 0 |
| Horario administracion |  |  |  |
| noMd | 13 (100%) | 13 (100%) | 0 (NA%) |
| c/24hr | 0 (0%) | 0 (0%) | 0 (NA%) |
| c/12hr | 0 (0%) | 0 (0%) | 0 (NA%) |
| c/8hr | 0 (0%) | 0 (0%) | 0 (NA%) |
| c/6hr | 0 (0%) | 0 (0%) | 0 (NA%) |
| Unknown | 0 | 0 | 0 |
| Dias de medicamento prescrito | 0 (0%) | 0 (0%) | 0 (NA%) |
| Unknown | 0 | 0 | 0 |
| Tiempo a cirugía (dias) | NA (NA, NA) | NA (NA, NA) | NA (NA, NA) |
| Unknown | 13 | 13 | 0 |
| Líquidos endovenosos durante hospitalización (mL) | 1,100 (900, 2,000) | 1,100 (900, 2,000) | NA (NA, NA) |
| Unknown | 0 | 0 | 0 |
| Antinflamatorios | 13 (100%) | 13 (100%) | 0 (NA%) |
| Unknown | 0 | 0 | 0 |
| Opioides | 13 (100%) | 13 (100%) | 0 (NA%) |
| Unknown | 0 | 0 | 0 |
| Infeccion clinica | 0 (0%) | 0 (0%) | 0 (NA%) |
| Unknown | 0 | 0 | 0 |
| Enfermedad tromboembolica | 0 (0%) | 0 (0%) | 0 (NA%) |
| Unknown | 0 | 0 | 0 |
| Dolor por EVA |  |  |  |
| 1 | 5 (38%) | 5 (38%) | 0 (NA%) |
| 2 | 7 (54%) | 7 (54%) | 0 (NA%) |
| 3 | 1 (7.7%) | 1 (7.7%) | 0 (NA%) |
| Unknown | 0 | 0 | 0 |
| Insomnio | 1 (7.7%) | 1 (7.7%) | 0 (NA%) |
| Unknown | 0 | 0 | 0 |
| Constipacion | 0 (0%) | 0 (0%) | 0 (NA%) |
| Unknown | 0 | 0 | 0 |
| Ansiedad | 0 (0%) | 0 (0%) | 0 (NA%) |
| Unknown | 0 | 0 | 0 |
| Hemoglobina (gr/dL) |  |  |  |
| 12.8001 | 1 (50%) | 1 (50%) | 0 (NA%) |
| 15.0001 | 1 (50%) | 1 (50%) | 0 (NA%) |
| Unknown | 11 | 11 | 0 |
| Creatinina (mg/dL) | NA (NA, NA) | NA (NA, NA) | NA (NA, NA) |
| Unknown | 13 | 13 | 0 |
| perdidos |  |  |  |
| 0 | 0 (0%) | 0 (0%) | 0 (NA%) |
| 1 | 13 (100%) | 13 (100%) | 0 (NA%) |
| Unknown | 0 | 0 | 0 |
| estancia60 | 13 (100%) | 13 (100%) | 0 (NA%) |
| Unknown | 0 | 0 | 0 |
| estancianew |  |  |  |
| 1 | 4 (31%) | 4 (31%) | 0 (NA%) |
| 2 | 5 (38%) | 5 (38%) | 0 (NA%) |
| 3 | 2 (15%) | 2 (15%) | 0 (NA%) |
| 5 | 1 (7.7%) | 1 (7.7%) | 0 (NA%) |
| 6 | 1 (7.7%) | 1 (7.7%) | 0 (NA%) |
| Unknown | 0 | 0 | 0 |
| DDP | NA (NA, NA) | NA (NA, NA) | NA (NA, NA) |
| Unknown | 13 | 13 | 0 |
| ^1^n (%); Median (IQR) | | | |

## Numero de tomas de PAS y PAD--------
library(readxl)
Investigacion_DOC_2_ <- read_excel("C:/Users/Usuario/Downloads/Investigacion DOC (2).xlsx")

## New names:
## • `Hora Sistolica` -> `Hora Sistolica...8`
## • `Hora Sistolica` -> `Hora Sistolica...12`

View(Investigacion_DOC_2_)
datatomas<-Investigacion_DOC_2_

library(plotly)

library(janitor)

##
## Attaching package: 'janitor'
##
## The following objects are masked from 'package:stats':
##
## chisq.test, fisher.test

datatomas<-datatomas %>%
 clean_names()

str(datatomas)

## tibble [2,771 × 15] (S3: tbl_df/tbl/data.frame)
## $ codigo : chr [1:2771] "S320" "S823" "S270" "S271" ...
## $ episodio : chr [1:2771] "3336477" "3339321" "3340831" "3343902" ...
## $ cedula : chr [1:2771] "1000636729" "8457571" "8314764" "8246495" ...
## $ nombre : chr [1:2771] "TABARES_JIMENEZ_ANDRES_FELIPE" "SALDARRIAGA_GARCIA_RAMON_ANTONIO" "CATAÑO_ZAPATA_LUIS_BERNARDO" "QUIROZ_ARANGO_EDILBERTO_ANTONIO" ...
## $ edad : num [1:2771] 22 67 77 80 61 29 74 70 47 70 ...
## $ sexo : num [1:2771] 1 1 1 1 0 1 1 0 1 1 ...
## $ fecha_sistolica : POSIXct[1:2771], format: "2021-01-12" "2020-11-08" ...
## $ hora_sistolica_8 : POSIXct[1:2771], format: "1899-12-31 13:16:21" "1899-12-31 01:23:55" ...
## $ sistolica_140 : num [1:2771] 140 148 167 171 140 141 153 159 152 140 ...
## $ cantidad_sistolica : num [1:2771] 52 3 410 1135 1 ...
## $ fecha_diastolica : POSIXct[1:2771], format: "2021-01-14" "2020-11-07" ...
## $ hora_sistolica_12 : POSIXct[1:2771], format: "1899-12-31 13:17:50" "1899-12-31 19:45:43" ...
## $ diastolica_90 : num [1:2771] 97 102 98 97 96 NA 93 90 95 96 ...
## $ cantidad_diastolica: num [1:2771] 7 2 118 81 1 NA 123 10 373 20 ...
## $ doc_historia_enfer : chr [1:2771] "60000000000027508450" "60000000000027526784" "60000000000027544808" "60000000000027563589" ...

summary(datatomas$sistolica_140)

## Min. 1st Qu. Median Mean 3rd Qu. Max. NA's
## 140.0 142.0 146.0 148.8 153.0 225.0 1194

summary(datatomas$diastolica_90)

## Min. 1st Qu. Median Mean 3rd Qu. Max. NA's
## 90.00 91.00 93.00 95.11 97.00 159.00 1479

min(datatomas$sistolica_140 , na.rm = T)-
 max(datatomas$sistolica_140 , na.rm = T)

## [1] -85

range(datatomas$sistolica_140 , na.rm = T)

## [1] 140 225

min(datatomas$diastolica_90 , na.rm = T)-
 max(datatomas$diastolica_90 , na.rm = T)

## [1] -69

range(datatomas$diastolica_90 , na.rm = T)

## [1] 90 159

sum(is.na(datatomas$diastolica_90))

## [1] 1479

hist(datatomas$sistolica_140)


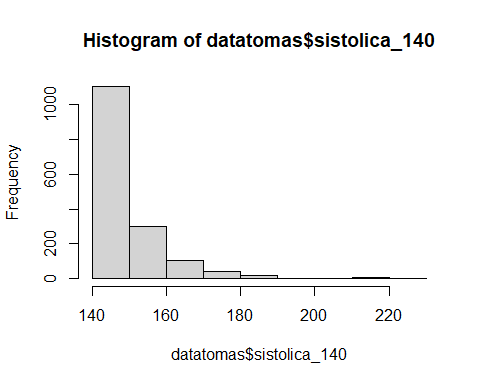


hist(datatomas$diastolica_90)


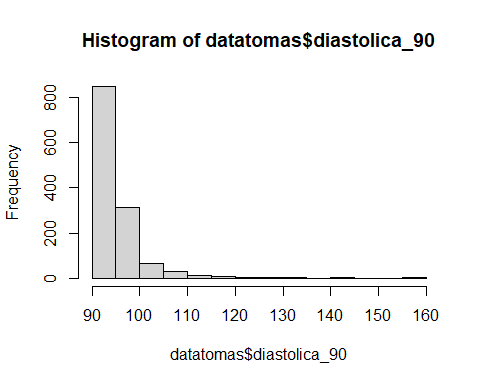


# Equal-width binning for sistolica_140
datatomas$sistolica_140_cat <- cut(
 datatomas$sistolica_140,
 breaks = 5,
 labels = c("140-157", "158-174", "175-191", "192-208", ">208")
)

# Equal-width binning for diastolica_90
datatomas$diastolica_90_cat <- cut(
 datatomas$diastolica_90,
 breaks = 5,
 labels = c("90-104", "105-119", "120-134", "135-149", ">150")
)

table(datatomas$sistolica_140_cat)

##
## 140-157 158-174 175-191 192-208 >208
## 1339 198 32 3 5

table(datatomas$diastolica_90_cat)

##
## 90-104 105-119 120-134 135-149 >150
## 1211 63 10 4 4

# Display the first few rows of the transformed datatomas
head(datatomas)

## # A tibble: 6 × 17
## codigo episodio cedula nombre edad sexo fecha_sistolica
## <chr> <chr> <chr> <chr> <dbl> <dbl> <dttm>
## 1 S320 3336477 1000636729 TABARES_JIMENEZ_AN… 22 1 2021-01-12 00:00:00
## 2 S823 3339321 8457571 SALDARRIAGA_GARCIA… 67 1 2020-11-08 00:00:00
## 3 S270 3340831 8314764 CATAÑO_ZAPATA_LUIS… 77 1 2021-03-04 00:00:00
## 4 S271 3343902 8246495 QUIROZ_ARANGO_EDIL… 80 1 2021-01-28 00:00:00
## 5 S211 3351918 70576778 CALLE_VALENCIA_ORL… 61 0 2020-11-23 00:00:00
## 6 S211 3352152 1017223711 OSORNO_VELASQUEZ_S… 29 1 2020-11-22 00:00:00
## # ℹ 10 more variables: hora_sistolica_8 <dttm>, sistolica_140 <dbl>,
## # cantidad_sistolica <dbl>, fecha_diastolica <dttm>,
## # hora_sistolica_12 <dttm>, diastolica_90 <dbl>, cantidad_diastolica <dbl>,
## # doc_historia_enfer <chr>, sistolica_140_cat <fct>, diastolica_90_cat <fct>

plot(datatomas$sistolica_140, xlab = datatomas$cantidad_sistolica)


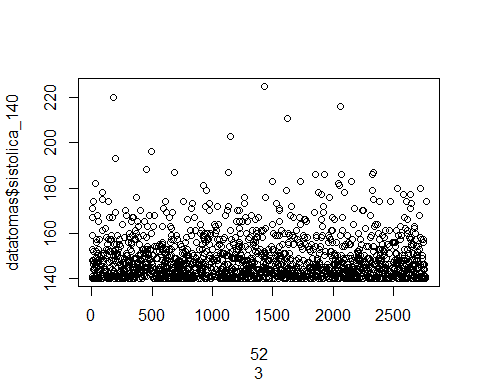


plot(datatomas$cantidad_diastolica, xlab = datatomas$diastolica_90_cat)


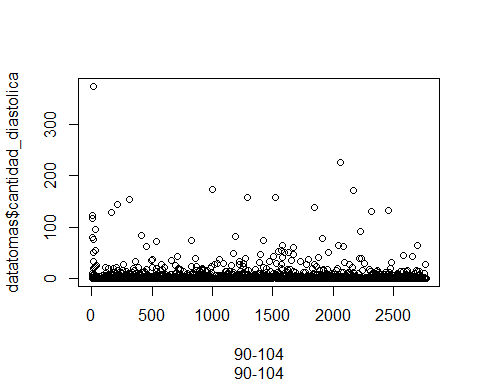


# Interactivo con cajas y bighotes y datos puntuales
sist <- ggplot(data= datatomas, mapping = aes(x= sistolica_140_cat, y=cantidad_sistolica, color= "blue")) + geom_boxplot()

ggplotly(sist)


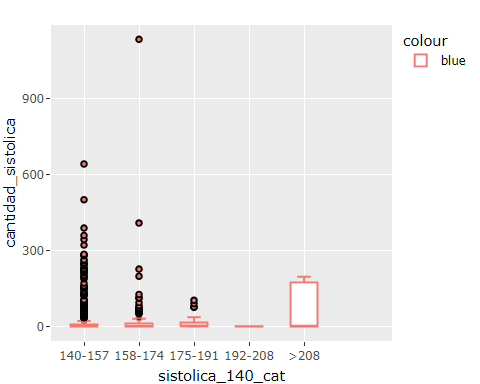


diast <- ggplot(data= datatomas, mapping = aes(x= diastolica_90_cat, y=cantidad_diastolica, color= "blue")) + geom_boxplot()

ggplotly(diast)


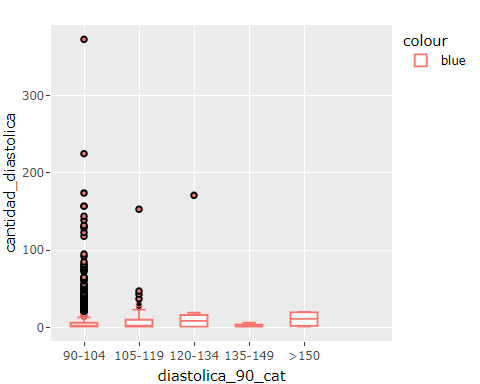


library(ggplot2)
library(plotly)

# Violin plot for 'sistolica_140_cat' vs 'cantidad_sistolica'
ggplot(data=datatomas, mapping=aes(x=sistolica_140_cat, y=cantidad_sistolica)) +
 geom_violin(fill="blue", color="black") +
 geom_jitter(width=0.1, color="red") +
 theme_minimal() +
 labs(title="Violin Plot for Systolic Blood Pressure", x="Systolic BP Category", y="Systolic BP Quantity")


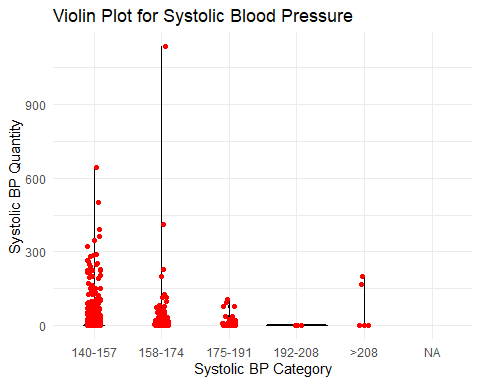


# Violin plot for 'diastolica_90_cat' vs 'cantidad_diastolica'
 ggplot(data=datatomas, mapping=aes(x=diastolica_90_cat, y=cantidad_diastolica)) +
 geom_violin(fill="blue", color="black") +
 geom_jitter(width=0.1, color="red") +
 theme_minimal() +
 labs(title="Violin Plot for Diastolic Blood Pressure", x="Diastolic BP Category", y="Diastolic BP Quantity")


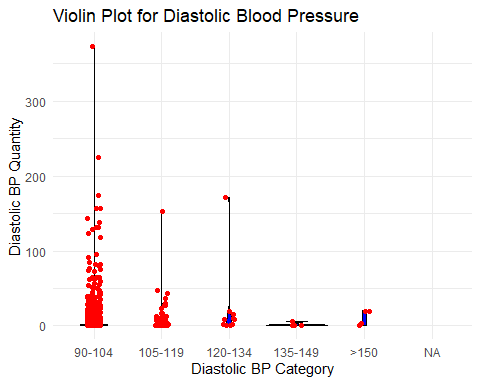


# TABLAS 1

# Tabla 1--------------------------------------------
library(gtsummary)

# Para efectoos de presentacion debemos etiquetar con un nombre correcto las variables
#(correr este codigo al final solo despues de los modelos) SOLO para efectos de presentacion.
#var_label(DBhtatx)<- list(edad= "Edad en años cumplidos", sexo="Género del participante")


# Con la funcion tbl_summary se construye y se excluye la identificacion
#Caracteristicas BASALES de ptes hombres jovenes con trauma y fx de extremidades (se complementara con visualizaciones)---------
DBhtatx %>% dplyr:::select (!c(nombre,id,ingreso,egreso,muerte,hipot, Hipot,estancia,hipotPAM,hipot_lev,hipot_md,hipot_vaso,qxco,Hipot_lev, Hipot_md,Hipot_vaso, Muerte, altavivo, compite,clase_med,ddd,horario_med,mg_med,ddp,clase_med,dias_med,dias_med, pas, pad, lev, aines, opioid, infx, etev, dolor, insomnio,constipa,ansiedad, hb ,creat, tiempo_qxco,rts,glasgow_coded, pasrts_coded,frecresp_coded,freccard)) %>% tbl_summary(by= antihta,
 label = list(sexo~"Sexo", raza~"Raza", edad~"Edad (años)",
 cRTS~"Puntaje de Trauma (RTS)", fractura~ "Hueso fracturado", gustillo~ "Clasificacion Gustillo Anderson (GA)",
 peso ~"Peso (Kg)", fuma~"Fumador cigarrillo, tabaco o vaper", alcohol~"Consumo de licor", obeso~ "Antecedente Obesidad", iam~"Infarto del miocardio",
 diabetes~"Diabetes Mellitus tipo 1 o 2", ecv~ "Enfermedad cerebrovascular", frecresp~"Frecuencia respiratoria al ingreso (rpm)", glasgow~"Escala coma Glasgow (pts.)",
 pasrts~"PAS al ingreso (mmHg)"), value = list(sexo~"Hombre", raza~"Mestizo",
 fuma~"Si", alcohol~"Consumo", obeso~ "Presente", iam~"IAM",
 diabetes~"Diabetes", ecv~ "ECV"
 ),missing = "always") %>% add_overall()

## Table printed with {flextable}, not {gt}. Learn why at
## https://www.danieldsjoberg.com/gtsummary/articles/rmarkdown.html
## To suppress this message, include `message = FALSE` in the code chunk header.

| **Characteristic** | **Overall**, N = 739^1^ | **No prescrito**, N = 656^1^ | **Prescrito**, N = 83^1^ |
| --- | --- | --- | --- |
| Sexo | 569 (77%) | 508 (77%) | 61 (73%) |
| Unknown | 0 | 0 | 0 |
| Raza | 731 (99%) | 649 (99%) | 82 (99%) |
| Unknown | 0 | 0 | 0 |
| Edad (años) | 35 (26, 52) | 35 (26, 52) | 35 (28, 52) |
| Unknown | 1 | 1 | 0 |
| Frecuencia respiratoria al ingreso (rpm) | 22.3 (20.3, 24.5) | 22.3 (20.3, 24.3) | 25.0 (21.5, 28.0) |
| Unknown | 0 | 0 | 0 |
| Escala coma Glasgow (pts.) | 15.00 (15.00, 15.00) | 15.00 (15.00, 15.00) | 15.00 (11.50, 15.00) |
| Unknown | 0 | 0 | 0 |
| PAS al ingreso (mmHg) | 128 (120, 135) | 128 (120, 135) | 130 (118, 140) |
| Unknown | 0 | 0 | 0 |
| Puntaje de Trauma (RTS) | 3.69 (3.69, 3.69) | 3.69 (3.69, 3.69) | 3.69 (3.33, 3.69) |
| Unknown | 0 | 0 | 0 |
| Hueso fracturado |  |  |  |
| Clavicula | 13 (1.8%) | 11 (1.7%) | 2 (2.4%) |
| Hombro | 4 (0.5%) | 4 (0.6%) | 0 (0%) |
| Humero | 45 (6.1%) | 41 (6.3%) | 4 (4.8%) |
| Codo | 7 (0.9%) | 6 (0.9%) | 1 (1.2%) |
| Radio o cubito | 57 (7.7%) | 51 (7.8%) | 6 (7.2%) |
| Muneca | 5 (0.7%) | 5 (0.8%) | 0 (0%) |
| Mano | 9 (1.2%) | 9 (1.4%) | 0 (0%) |
| Pelvis | 38 (5.1%) | 31 (4.7%) | 7 (8.4%) |
| Cadera | 41 (5.6%) | 37 (5.6%) | 4 (4.8%) |
| Femur | 186 (25%) | 155 (24%) | 31 (37%) |
| Rodilla | 23 (3.1%) | 20 (3.1%) | 3 (3.6%) |
| Tibia - perone | 252 (34%) | 228 (35%) | 24 (29%) |
| Tobillo | 24 (3.3%) | 23 (3.5%) | 1 (1.2%) |
| Pie | 34 (4.6%) | 34 (5.2%) | 0 (0%) |
| Unknown | 1 | 1 | 0 |
| Clasificacion Gustillo Anderson (GA) |  |  |  |
| Cerrada | 298 (40%) | 271 (41%) | 27 (33%) |
| GA1 | 136 (18%) | 124 (19%) | 12 (14%) |
| GA2 | 150 (20%) | 133 (20%) | 17 (20%) |
| GA3 | 155 (21%) | 128 (20%) | 27 (33%) |
| Unknown | 0 | 0 | 0 |
| Peso (Kg) | 69 (62, 72) | 68 (62, 72) | 70 (66, 80) |
| Unknown | 0 | 0 | 0 |
| Fumador cigarrillo, tabaco o vaper | 347 (47%) | 312 (48%) | 35 (42%) |
| Unknown | 0 | 0 | 0 |
| Consumo de licor | 186 (25%) | 135 (21%) | 51 (61%) |
| Unknown | 0 | 0 | 0 |
| Antecedente Obesidad | 46 (6.2%) | 34 (5.2%) | 12 (14%) |
| Unknown | 0 | 0 | 0 |
| Diabetes Mellitus tipo 1 o 2 | 35 (4.7%) | 28 (4.3%) | 7 (8.4%) |
| Unknown | 0 | 0 | 0 |
| Infarto del miocardio | 3 (0.4%) | 2 (0.3%) | 1 (1.2%) |
| Unknown | 0 | 0 | 0 |
| Enfermedad cerebrovascular | 2 (0.3%) | 2 (0.3%) | 0 (0%) |
| Unknown | 0 | 0 | 0 |
| perdidos |  |  |  |
| 0 | 726 (98%) | 643 (98%) | 83 (100%) |
| 1 | 13 (1.8%) | 13 (2.0%) | 0 (0%) |
| Unknown | 0 | 0 | 0 |
| estancia60 | 723 (98%) | 651 (99%) | 72 (87%) |
| Unknown | 0 | 0 | 0 |
| estancianew | 9 (6, 17) | 9 (6, 15) | 27 (15, 38) |
| Unknown | 16 | 5 | 11 |
| DDP | 13 (7, 35) | NA (NA, NA) | 13 (7, 35) |
| Unknown | 657 | 656 | 1 |
| ^1^n (%); Median (IQR) | | | |

DBhtatx %>% select (
 !c(sexo, raza, edad, rts,peso, glasgow,pasrts,freccard,
 nombre,fuma,diabetes,iam,obeso,ecv,alcohol,
 id,Hipot_lev, Hipot_md,Hipot_vaso, Muerte, altavivo, compite,
 ingreso,
 egreso,
 muerte,
 hipot, Hipot,
 estancia,
 hipotPAM,
 hipot_lev,
 hipot_md,
 hipot_vaso,
 qxco,
 ddd,
 mg_med,
 ddp,
 fractura,
 gustillo,glasgow_coded,pasrts_coded,frecresp,frecresp_coded,cRTS
 )
) %>% tbl_summary(
 by = antihta,
 label = list(
 clase_med~ "Medicamento antihipertensivo",
 horario_med~ "Horario administracion",
 dias_med ~ "Dias de medicamento prescrito",
 pas ~ "Presión arterial sistólica (mmHg)",
 pad ~ "Presión arterial diastólica (mmHg)",
 tiempo_qxco ~ "Tiempo a cirugía (dias)",
 lev ~
 "Líquidos endovenosos durante hospitalización (mL)",
 aines ~ "Antinflamatorios",
 opioid ~ "Opioides",
 infx ~ "Infeccion clinica",
 etev ~ "Enfermedad tromboembolica",
 dolor ~
 "Dolor por EVA",
 insomnio ~ "Insomnio",
 constipa ~ "Constipacion",
 ansiedad ~ "Ansiedad",
 hb ~ "Hemoglobina (gr/dL)",
 creat ~ "Creatinina (mg/dL)"
 ),
 value = list(
 opioid ~ "Prescrito",
 infx ~ "Presente",
 etev ~ "Presente",
 insomnio ~
 "Presente",
 constipa ~ "Presente",
 ansiedad ~ "Presente",
 aines ~
 "Prescrito"
 ),
 missing = "always"
) %>% add_overall()

## Table printed with {flextable}, not {gt}. Learn why at
## https://www.danieldsjoberg.com/gtsummary/articles/rmarkdown.html
## To suppress this message, include `message = FALSE` in the code chunk header.

| **Characteristic** | **Overall**, N = 739^1^ | **No prescrito**, N = 656^1^ | **Prescrito**, N = 83^1^ |
| --- | --- | --- | --- |
| Presión arterial sistólica (mmHg) | 152 (143, 162) | 151 (141, 159) | 168 (161, 177) |
| Unknown | 0 | 0 | 0 |
| Presión arterial diastólica (mmHg) | 91 (88, 97) | 91 (88, 96) | 98 (92, 102) |
| Unknown | 0 | 0 | 0 |
| Medicamento antihipertensivo |  |  |  |
| noMd | 656 (89%) | 656 (100%) | 0 (0%) |
| Losartan | 9 (1.2%) | 0 (0%) | 9 (11%) |
| Enalapril | 5 (0.7%) | 0 (0%) | 5 (6.1%) |
| Amlodipino | 8 (1.1%) | 0 (0%) | 8 (9.8%) |
| Metoprolol | 5 (0.7%) | 0 (0%) | 5 (6.1%) |
| Hidroclorotiazida | 26 (3.5%) | 0 (0%) | 26 (32%) |
| Prazosin | 1 (0.1%) | 0 (0%) | 1 (1.2%) |
| Clonidina | 28 (3.8%) | 0 (0%) | 28 (34%) |
| Unknown | 1 | 0 | 1 |
| Horario administracion |  |  |  |
| noMd | 655 (89%) | 655 (100%) | 0 (0%) |
| c/24hr | 26 (3.5%) | 0 (0%) | 26 (31%) |
| c/12hr | 41 (5.6%) | 0 (0%) | 41 (49%) |
| c/8hr | 15 (2.0%) | 0 (0%) | 15 (18%) |
| c/6hr | 1 (0.1%) | 0 (0%) | 1 (1.2%) |
| Unknown | 1 | 1 | 0 |
| Dias de medicamento prescrito | 0.00 (0.00, 0.00) | 0.00 (0.00, 0.00) | 4.00 (2.50, 7.00) |
| Unknown | 0 | 0 | 0 |
| Tiempo a cirugía (dias) | 3.0 (2.0, 5.0) | 3.0 (2.0, 5.0) | 5.0 (2.0, 11.5) |
| Unknown | 13 | 13 | 0 |
| Líquidos endovenosos durante hospitalización (mL) | 8,500 (5,300, 16,800) | 7,800 (5,100, 13,800) | 24,500 (13,850, 30,000) |
| Unknown | 0 | 0 | 0 |
| Antinflamatorios | 735 (99%) | 653 (100%) | 82 (99%) |
| Unknown | 0 | 0 | 0 |
| Opioides | 732 (99%) | 650 (99%) | 82 (99%) |
| Unknown | 0 | 0 | 0 |
| Infeccion clinica | 165 (22%) | 117 (18%) | 48 (58%) |
| Unknown | 1 | 1 | 0 |
| Enfermedad tromboembolica | 7 (0.9%) | 4 (0.6%) | 3 (3.6%) |
| Unknown | 0 | 0 | 0 |
| Dolor por EVA |  |  |  |
| 1 | 43 (5.8%) | 42 (6.4%) | 1 (1.2%) |
| 2 | 360 (49%) | 345 (53%) | 15 (18%) |
| 3 | 336 (45%) | 269 (41%) | 67 (81%) |
| Unknown | 0 | 0 | 0 |
| Insomnio | 276 (37%) | 216 (33%) | 60 (72%) |
| Unknown | 0 | 0 | 0 |
| Constipacion | 186 (25%) | 135 (21%) | 51 (61%) |
| Unknown | 0 | 0 | 0 |
| Ansiedad | 123 (17%) | 74 (11%) | 49 (59%) |
| Unknown | 0 | 0 | 0 |
| Hemoglobina (gr/dL) | 11.80 (9.50, 13.10) | 12.00 (9.90, 13.23) | 8.40 (7.25, 11.45) |
| Unknown | 72 | 72 | 0 |
| Creatinina (mg/dL) | 0.82 (0.64, 1.01) | 0.80 (0.62, 0.97) | 1.02 (0.80, 1.52) |
| Unknown | 139 | 139 | 0 |
| perdidos |  |  |  |
| 0 | 726 (98%) | 643 (98%) | 83 (100%) |
| 1 | 13 (1.8%) | 13 (2.0%) | 0 (0%) |
| Unknown | 0 | 0 | 0 |
| estancia60 | 723 (98%) | 651 (99%) | 72 (87%) |
| Unknown | 0 | 0 | 0 |
| estancianew | 9 (6, 17) | 9 (6, 15) | 27 (15, 38) |
| Unknown | 16 | 5 | 11 |
| DDP | 13 (7, 35) | NA (NA, NA) | 13 (7, 35) |
| Unknown | 657 | 656 | 1 |
| ^1^Median (IQR); n (%) | | | |

# Para seguir con el orden descriptivo de Desenlaces Hipotension - Muerte - Estancia :
# cambiaremos el orden en la base de datos


DBhtatx <- DBhtatx |>
 relocate(hipot, .after = ecv)

DBhtatx <- DBhtatx |>
 relocate(hipot_vaso, .after = hipot)

DBhtatx <- DBhtatx |>
 relocate(hipot_lev, .after = hipot)

DBhtatx <- DBhtatx |>
 relocate(hipot_md, .after = hipot)

DBhtatx <- DBhtatx |>
 relocate(muerte, .after = hipot_vaso)

DBhtatx <- DBhtatx |>
 relocate(estancia, .after = muerte)

# Tabla 2 Desenlaces sin ajustes segun prescripcion de antihipertensivos
DBhtatx %>% select (!c(nombre,id,ingreso,egreso, sexo,raza, edad, pas, pad, rts, Hipot, lev, aines, opioid, infx , etev,dolor, insomnio, constipa, ansiedad, hb, creat, peso , hipotPAM, Hipot_lev, Hipot_md,Hipot_vaso, Muerte, altavivo, compite,
 fuma, alcohol, obeso, iam,diabetes, ecv, freccard, glasgow,pasrts, fractura,gustillo,qxco, tiempo_qxco,dias_med,ddp,mg_med,ddd,clase_med,horario_med,frecresp,frecresp_coded,pasrts_coded,glasgow_coded, cRTS)) %>% tbl_summary(by= antihta,
 label = list(muerte~ "Muerte",hipot ~ "Hipotensión (PAM <65mmHg)",
 hipot_lev ~ "Hipotensión que requiere liquidos endovenosos",hipot_md~ "Hipotensión que requiere suspender medicamentos",
 hipot_vaso ~"Hipotensión que requiere vasopresor",estancia~ "Estancia (dias)"), value = list(hipot~"presente",hipot_lev~"presente",hipot_md~"presente",hipot_vaso~"presente"), missing = "always") %>% add_p() %>% add_overall()

## There was an error in 'add_p()/add_difference()' for variable 'DDP', p-value omitted:
## Error in wilcox.test.formula(as.numeric(DDP) ~ as.factor(antihta), data = structure(list(: grouping factor must have exactly 2 levels
## Table printed with {flextable}, not {gt}. Learn why at
## https://www.danieldsjoberg.com/gtsummary/articles/rmarkdown.html
## To suppress this message, include `message = FALSE` in the code chunk header.

| **Characteristic** | **Overall**, N = 739^1^ | **No prescrito**, N = 656^1^ | **Prescrito**, N = 83^1^ | **p-value**^2^ |
| --- | --- | --- | --- | --- |
| Hipotensión (PAM <65mmHg) | 182 (25%) | 114 (17%) | 68 (82%) | <0.001 |
| Unknown | 0 | 0 | 0 |  |
| Hipotensión que requiere suspender medicamentos | 63 (8.5%) | 16 (2.4%) | 47 (57%) | <0.001 |
| Unknown | 0 | 0 | 0 |  |
| Hipotensión que requiere liquidos endovenosos | 99 (13%) | 36 (5.5%) | 63 (76%) | <0.001 |
| Unknown | 0 | 0 | 0 |  |
| Hipotensión que requiere vasopresor | 34 (4.6%) | 12 (1.8%) | 22 (27%) | <0.001 |
| Unknown | 0 | 0 | 0 |  |
| Muerte |  |  |  | <0.001 |
| sobrevivio | 728 (99%) | 651 (99%) | 77 (93%) |  |
| fallecio | 11 (1.5%) | 5 (0.8%) | 6 (7.2%) |  |
| Unknown | 0 | 0 | 0 |  |
| Estancia (dias) | 10 (6, 18) | 9 (6, 15) | 31 (16, 46) | <0.001 |
| Unknown | 0 | 0 | 0 |  |
| perdidos |  |  |  | 0.4 |
| 0 | 726 (98%) | 643 (98%) | 83 (100%) |  |
| 1 | 13 (1.8%) | 13 (2.0%) | 0 (0%) |  |
| Unknown | 0 | 0 | 0 |  |
| estancia60 | 723 (98%) | 651 (99%) | 72 (87%) | <0.001 |
| Unknown | 0 | 0 | 0 |  |
| estancianew | 9 (6, 17) | 9 (6, 15) | 27 (15, 38) | <0.001 |
| Unknown | 16 | 5 | 11 |  |
| DDP | 13 (7, 35) | NA (NA, NA) | 13 (7, 35) |  |
| Unknown | 657 | 656 | 1 |  |
| ^1^n (%); Median (IQR) | | | | |
| ^2^Pearson's Chi-squared test; Fisher's exact test; Wilcoxon rank sum test | | | | |

# infIndexPlot()
# leveragePlot()

# Ajuste para multiples desenlaces-------

 # Suppose these are the p-values from your four tests
 p_values <- c(0.01, 0.094, 0.066)
 #p_valueshipot <-c(0.001, 0.001,0.037)

 # Number of tests
 m <- length(p_values)

 # Sort the p-values and get their indices
 sorted_p_values <- sort(p_values)
 sorted_indices <- order(p_values)

 # Calculate Holm-Bonferroni adjusted alpha levels
 adjusted_alpha <- 0.05 / (m:1)

 # Compare p-values to adjusted alpha levels
 rejected <- sorted_p_values <= adjusted_alpha

 # Print results
 data.frame(
 Test = sorted_indices,
 p_value = sorted_p_values,
 adjusted_alpha = adjusted_alpha,
 rejected = rejected
 )

## Test p_value adjusted_alpha rejected
## 1 1 0.010 0.01666667 TRUE
## 2 3 0.066 0.02500000 FALSE
## 3 2 0.094 0.05000000 FALSE

# Ajuste para multiples desenlaces de hipotension-------

 # Suppose these are the p-values from your four tests

p_valueshipot <-c(0.001, 0.001,0.037)

 # Number of tests
 mh <- length(p_valueshipot)

 # Sort the p-values and get their indices
 sorted_p_valuesh <- sort(p_valueshipot)
 sorted_indicesh <- order(p_valueshipot)

 # Calculate Holm-Bonferroni adjusted alpha levels
 adjusted_alphah <- 0.05 / (mh:1)

 # Compare p-values to adjusted alpha levels
 rejectedh <- sorted_p_valuesh <= adjusted_alphah

 # Print results
 data.frame(
 Test = sorted_indicesh,
 p_value = sorted_p_valuesh,
 adjusted_alphah = adjusted_alphah,
 rejectedh = rejectedh
 )

## Test p_value adjusted_alphah rejectedh
## 1 1 0.001 0.01666667 TRUE
## 2 2 0.001 0.02500000 TRUE
## 3 3 0.037 0.05000000 TRUE
